# Single Tri-Epitopic Antibodies (TeAbs) to Botulinum Neurotoxin Serotypes B, E, and F Recapitulate the Full Potency of a Combination of Three Monoclonal Antibodies in Toxin Neutralization

**DOI:** 10.3390/toxins17060281

**Published:** 2025-06-04

**Authors:** Jianlong Lou, Wei Hua Wen, Fraser Conrad, Christina C. Tam, Consuelo Garcia-Rodriguez, Shauna Farr-Jones, James D. Marks

**Affiliations:** 1 Department of Anesthesia and Perioperative Care, University of California, Zuckerberg San Francisco General Hospital, Box 1327, 2540 23rd St, San Francisco, CA 94143, USAshauna.farr-jones@ucsf.edu (S.F.-J.); 2 Foodborne Toxin Detection and Prevention Research Unit, Western Regional Research Center, Agricultural Research Services, United States Department of Agriculture, 800 Buchanan Street, Albany, CA 94710, USA

**Keywords:** tri-epitopic antibody, recombinant monoclonal antibody, botulinum neurotoxin, antitoxin, botulism, serotypes B, E, F, mouse neutralization assay, kinetic exclusion analysis

## Abstract

Recombinant monoclonal antibody (mAb) botulinum neurotoxin (BoNT) antitoxins, consisting of three mAbs that bind non-overlapping epitopes, are highly potent. However, the three-mAb mixtures pose unique development and manufacturing challenges. Combining even more mAbs to create multivalent antitoxin drugs multiplies those challenges. We previously reported that a single tri-epitopic IgG1-based mAb (TeAb) containing the variable domains of the three parental BoNT/A mAbs and an Fc was as potent as the combination of three IgGs in the mouse neutralization assay (MNA). Here, we extended the tri-epitopic strategy to three other BoNT serotypes. Each TeAb (TeAb-B for BoNT/B, TeAb-E for BoNT/E, and TeAb-F for BoNT/F) binding was measured using fluorescence-activated cell sorting and flow fluorimetry, and the potency was tested in the MNA. The three TeAbs displayed binding affinities that were the same within error of the parental IgGs for each epitope, and all had higher avidity to each serotype of BoNT than that of the parental mAbs. The potency of the BoNT/B, BoNT/E, and BoNT/F TeAbs was similar to the combinations of the three parental IgGs binding BoNT/B, BoNT/E, and BoNT/F in the MNA. We now have four examples of a single TeAb recapitulating the affinity and in vivo potency of a three-mAb antitoxin. The tri-epitopic strategy could be applied to streamline the production and bioanalytics of antibody drugs where three-mAb binding is required for activity.

## 1. Introduction

Botulism is caused by botulinum neurotoxin (BoNT) produced by *Clostridia* species. Botulism occurs globally in infants [1] and adults [2,3,4,5]. The currently FDA-approved treatment for botulism is equine antitoxin [6,7] for adults and human immune globulin (BabyBIG^®^) for infants [8,9,10,11]. The FDA-approved treatments—equine-derived heptavalent antitoxin (HBAT) for adults and human-derived immunoglobulin (BabyBIG^®^) for infants—reduce mortality but are limited by non-renewable production via equine or human immunization, manufacturing challenges, hypersensitivity risks, and supply constraints, particularly for BabyBIG^®^ due to the lack of an FDA-approved BoNT vaccine [7,12,13,14].

For the above reasons, we have been developing monoclonal antibody (mAb) combinations to prevent and treat botulism. Our initial studies with mAbs to BoNT showed that single mAbs neutralized at most 10–20 LD_50_ per 50 μg of mAb, regardless of affinity or epitope [15]. Combining two mAbs that bind non-overlapping epitopes increased the potency 75-fold, and adding a third mAb, which binds a non-overlapping epitope, increased the potency a further 15-fold, making a three-mAb combination more than 1000-fold more potent than any individual mAb. We have published similar results for other serotypes including BoNT/E where a three-mAb combination was 40 times more potent than the most potent individual mAb, and adding a third mAb to the combination increased the potency a further 20-fold, making the three-mAb combination more than 800 times more potent than an individual mAb [16]. Similar increases in potency were observed for three mAb combinations to BoNT/B and BoNT/F (Unpublished data).

Recombinant human or humanized mAb combinations of antitoxins are in clinical development, including antitoxin for BoNT/A [17], for BoNT/B [18], and for BoNT/E [19] and divalent antitoxins for BoNT/C and/or BoNT/D [20] and for BoNT/A and BoNT/B (NCT05348993). The antitoxin for monovalent BoNT/F [21] is in pre-clinical development.

Multi-mAb antitoxins targeting all BoNT serotypes (A–G) face complex manufacturing, bioanalytical, and regulatory challenges [22,23]. While cross-reactive mAbs for BoNT/C, BoNT/D, BoNT/E, and BoNT/F reduce the number of IgGs needed, a heptavalent product may still require up to 16 IgGs.

We developed tri-epitopic antibodies (TeAbs), single IgG1 molecules with three binding domains, to match the potency of three-mAb combinations. Our BoNT/A TeAb recapitulated this potency in the mouse neutralization assay (MNA) [24].

Others have recognized the need and challenge of developing a combination and have taken different and promising approaches. Lu et al. reported a biepitopic antibody that binds two different epitopes on BoNT/A; the biepitopic antibody was 20-fold more potent than a single antibody in the MNA [25]. Bispecific binding via VHH has also been reported. VHHs offer several advantages over IgGs since they can be easily fused to construct multi-valent binders [26,27]. They are thermally stable and easy to manufacture and can be produced in microbial systems but must be fused to Fc to achieve a sufficiently long half-life and for the immune complexes to be cleared by the liver via FcgR2 [27]. Another advantage is that VHH can be encoded in mRNA for in vivo expression [28]. Furthermore, VHH can be delivered intracellularly when fused to internalizing proteins, presenting the potential for post-exposure treatment [29].

Here, we report that the design strategy used for the tri-epitopic BoNT/A antibody is generalizable to BoNT serotypes B, E, and F. These four serotypes are responsible for almost all botulism cases [30]. Each tri-epitopic mAb combines variable regions of high-affinity antibodies specific to the BoNT serotypes that have demonstrated high potency [21,31]. This work opens the possibility of a heptavalent antitoxin consisting of seven or fewer tri-epitopic antibodies and may be generalizable for targeting other toxins [32,33,34].

## 2. Results

### TeAb Constructs

**Figure 1:**
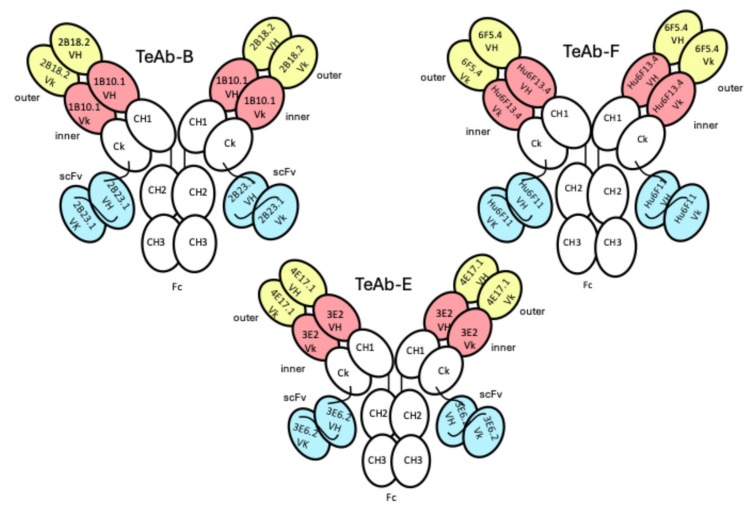
Cartoon of TeAb-B, TeAb-E, and TeAb-F constructs containing three variable domains numbered VH1 and Vk1 (inner, coral), VH2 and Vk2 (outer, yellow), and VH3 and Vk3 (scFv, blue). For BoNT serotype B, variable domain 1 (inner, coral) = mAb 1B10.1, variable domain 2 (outer, yellow) = mAb 2B18.2, and variable domain 3 (scFv, blue) = mAb 2B23.1 [36]. For BoNT serotype E, variable domain 1 (inner, coral) = mAb 3E2, variable domain 2 (outer, yellow) = mAb 4E17.1, and variable domain 3 (scFv, blue) = mAb 3E6.2 [16]. For BoNT serotype F, variable domain 1 (inner, coral) = mAb hu6F13.4, variable domain 2 (outer, yellow) = mAb 6F5.4, and variable domain 3 (scFv, blue) = mAb hu6F11 [35].

Tri-epitopic antibodies were constructed using variable domains from antibodies previously found to provide potent protection in the mouse neutralization assay (MNA) [16,35,36]. The design of the TeAb construct is illustrated in Figure 1. It is designed based on the dual variable domain [37] with a single-chain Fv (scFv) fused to the C-terminus of the kappa constant domain with a peptide linker (VH3 and Vk3 in Figure 1) [24]. The choice of the location of the various binding domains was based on our ability to express the TeAb in adequate yield and purity for characterization.

We initially designed the TeAb-F to have 6F5.4 in the outer position and Hu6F11 in the inner position, while Hu6F13.4 was the Ck-linked scFv domain. However, this TeAb-F construct had extremely poor expression. Therefore, we swapped the arrangement of the domains so that hu6F13.4 was in the inner position and hu6F11 was in the scFv position. We also changed the linker between the two Vk domains by removing the last residue (D) from the linker (TVAAPSVFIFPPSD) that was used for TeAb-A, TeAb-B, and TeAb-E [24] because the first residue of the Framework 1 sequence of hu6F13.4 was an aspartic acid. Using the new construct for TeAb-F (Figure 1), we were able to produce TeAb-F in adequate yield and purity for characterization.

**Figure 2:**
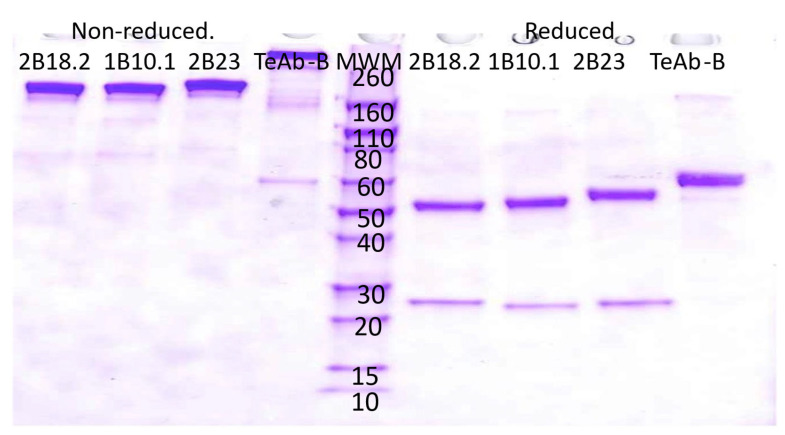
Electrophoretic analysis of anti-BoNT B IgG and TeAb-B construct run on a SDS-PAGE 4–20% acrylamide gel. The loaded samples (5 µg/lane) were as follows: lanes 1–4, 2B18.2 IgG, 1B10.1 IgG, 2B23 IgG, and TeAb-B under non-reducing conditions; lanes 6–9, 2B18.2 IgG, 1B10.1 IgG, 2B23 IgG, and TeAb-B under reducing conditions. Lane 5, Novex Sharp Pre-stained Molecular Weight Marker standards (Thermo Fisher Scientific, LC5800, Grand Island, NY 14072, USA).

**Figure 3:**
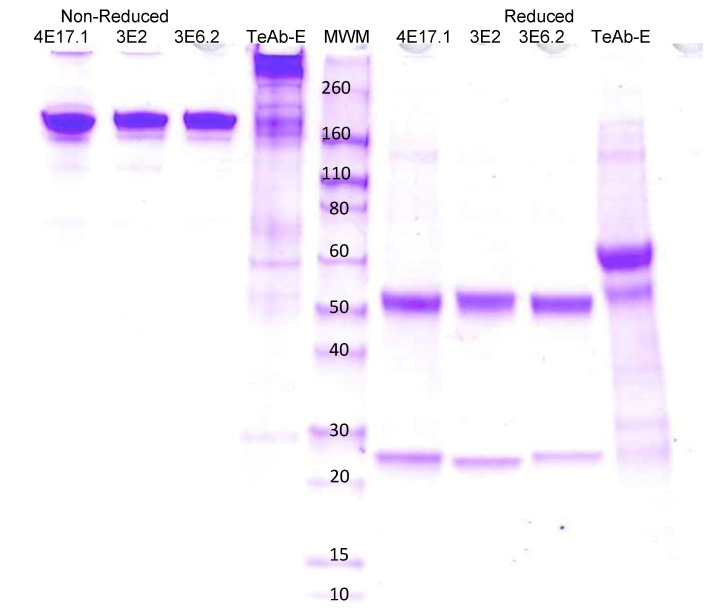
Electrophoretic analysis of anti-BoNT E IgGs and TeAb-E construct produced at 32 °C on SDS PAGE gel 4–20% acrylamide. 4E17.1, 3E2, 3E6.2 IgG [16], and TeAb-E produced at 32 °C. The loaded samples (5 µg/lane) were as follows: lanes 1–4, 4E17.1, 3E2, 3E6.2 IgG, and TeAb-E non-reduced; lanes 6–9, 4E17.1, 3E2, 3E6.2 IgG, and TeAb-E reduced (2 µg/lane). Lane 5, Novex Sharp Pre-stained Molecular Weight Marker.

HEK293 cells were used for transient expression of TeAb-B, TeAb-E, or TeAb-F, while CHO cells with methotrexate amplification after G418 selection were used for stable expression. Cell lines producing TeAb-B at 5–10 mg/L with purity over 90% were generated. However, cell lines for TeAb-E had a lower yield of 2 mg/L and were prone to degradation when expressed at 37 °C. We therefore expressed TeAb-E at 32 °C to reduce degradation of the antibody. TeAb-B was over 90% pure as depicted by SDS-PAGE analysis with reducing SDS-PAGE showing a single band of approximately 60 kDa, as expected, as the scFv fusion to Ck resulted in the heavy and light chains having the same approximate MW (Figure 2). The purity of the TeAb-E was lower and estimated at 80–90% by SDS-PAGE analysis with the reducing gel showing a degradation product at approximately 50 kDa, consistent with one domain of the scFv being proteolyzed, likely at the linker between the VH and Vk domains (Figure 3).

In vivo protection assays against subtype 1 of BoNT/B (BoNT/B1) or subtype 3 of BoNT/E (BoNT/E3) toxin were performed using TeAb-B or TeAb-E from stable cell lines.

**Figure 4:**
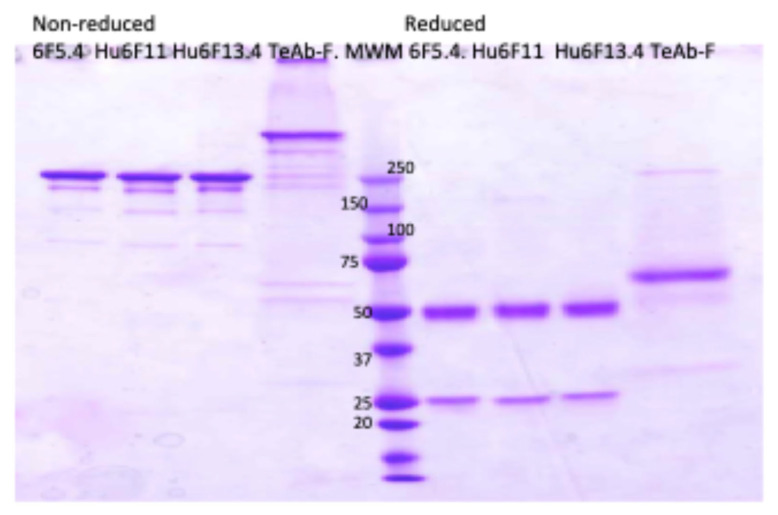
Electrophoretic analysis of anti-BoNT F IgG and TeAb-F construct produced at 32 °C on SDS PAGE gel, 4–20% acrylamide. 6F5.4, Hu6F11, Hu6F13.4 IgG, and TeAb-F were produced at 32 °C. The loaded samples (2 µg/lane) were as follows: lanes 1–4, non-reduced; lanes 6–9, reduced. Lane 5, Novex sharp Pre-stained Molecular Weight Marker.

TeAb-F was produced in HEK293 cells with a purity of approximately 80–90% as determined using SDS PAGE, shown in Figure 4. The HEK293 expressed TeAb-F was used for the MNA studies.

We measured the affinity of TeAb-B, TeAb-E, and TeAb-F using KinExA for each of the three epitopes of BoNT/B1 (Table 1), BoNT/E3 (Table 2), and BoNT/F (Table 3) using purified BoNT domains and holotoxin specific for each epitope.

TeAb-B bound the 1B10.1, 2B18.2, and 2B23 epitopes on their respective recombinant BoNT domains with very high affinity, ranging from 0.965 pM (1B10.1 epitope) to 20.53 pM (2B18.2 epitope) (Table 1). These affinities were within two- to four-fold of the parental variable domains in the IgG construct.

The affinity of TeAb-B for BoNT/B1 holotoxin ranged from 0.254 pM (2B23.1 epitope) to 92.84 pM (2B18.2 epitope). The affinity was the same as parental BoNT/B IgGs (within error) for the 1B10.1 and 2B18.2 epitopes, while the TeAb-B affinity was over 100 times higher for the 2B23.1 epitope than the parental BoNT/B IgG, reflecting a huge avidity effect at the 2B23.1 epitope for reasons that are unclear.

TeAb-E bound the 3E2, 4E17.1, and 3E6.2 epitopes on their respective recombinant BoNT domains with very high affinity, ranging from 7.48 pM (3E2 epitope) to 18.15 pM (4E17.1 epitope) (Table 2). These affinities were within two- to six-fold of the domains in the parental IgG construct.

The affinity of TeAb-E for BoNT/E3 holotoxin ranged from 1.18 pM (3E2 epitope) to 0.428 pM (4E17.1 epitope). TeAb-E bound to the 3E2 epitope with the same affinity as the parental IgG (within error) and at the 3E6.2 epitope with 10-fold higher affinity. There was a huge avidity effect for the TeAb at the 4E17.1 epitope, which bound BoNT/E3 holotoxin with a 558-fold higher affinity than the parental BoNT/E IgG for reasons that are unclear.

TeAb-F bound the 6F5.4, Hu6F11, and Hu6F13.4 epitopes on their respective recombinant BoNT domains with very high affinity, ranging from 4.4 to 1790 pM (Table 3). The affinity for the different BoNT/F epitopes ranged from the same (within error for the 6F5.4 epitope) to 5-fold (Hu6F11 epitope) to 66-fold (Hu6F13.4 epitope) lower compared with the parental IgG affinity.

The affinity of TeAb-F for BoNT/F holotoxin ranged from 15.5 pM (hu6F13.4 epitope) to 26.6 pM (6F5.4 epitope). The affinity of TeAb-F was 5.2-fold higher for the hu6F13.4 epitope, 7.1-fold higher for the 6F5.4 epitope, and 68-fold lower for the hu6F11 epitope compared with the parental BoNT/F IgGs.

**Figure 5:**
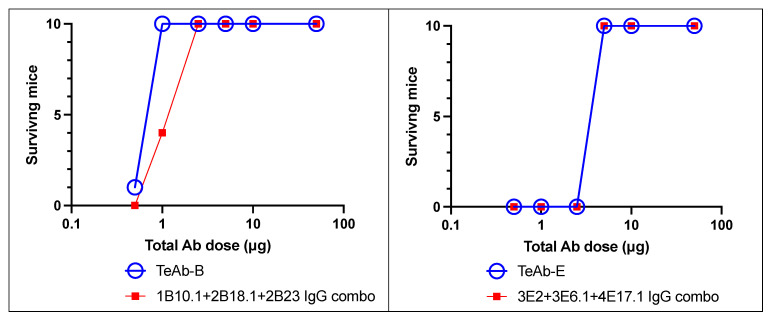
TeAb-B and TeAb-E potently protected mice against BoNT intoxication. Mouse neutralization assays with 40,000 LD_50_ of BoNT/B or BoNT/E by TeAb-B, TeAb-E, or combinations of parental IgG antibodies were tested. BoNT was injected intraperitoneally into cohorts of 10 mice. TeAb or IgG combo were combined with BoNT holotoxin for 30 min in phosphate gelatin buffer before injection. The number of mice surviving after 5 days was scored.

We then tested the potency of TeAb-B and TeAb-E in the MNA, using up to 40,000 mouse LD_50_. As shown in Figure 5, TeAb-B and TeAb-E were equipotent when compared with the combination of their three parental IgG. One µg of TeAb or IgG combination/mouse protected mice against 40,000 mouse LD_50_ of BoNT/B1, and 6.25 µg/mouse protected mice against 40,000 mouse LD_50_ of BoNT/E3.

We then tested TeAb-F in the MNA, using up to 40,000 mouse LD_50_ of serotype 1 of BoNT/F (BoNT/F1). As shown in Table 4, TeAb-F was highly potent, with 50 µg/mouse protecting against 40,000 mouse LD_50_ of BoNT/F1. We did not have, nor could we obtain, sufficient BoNT/F1 holotoxin to repeat these experiments to find the lowest concentration of antibodies that provided protection. However, the TeAb-F exhibited highly potent BoNT/F neutralization with a calculated potency of 80 IU/mg, where 1 IU = an amount of antitoxin neutralizing 10,000 mouse LD_50_ of BoNT/F.

## 3. Discussion

We previously showed that combining three mAbs that bind non-overlapping epitopes leads to highly potent botulinum neurotoxin type (BoNT) neutralization. Combinations of three mAbs to BoNT/A [15], BoNT/B [36], BoNT/E [16], BoNT C/D [38], and BoNT/F [21] that bind non-overlapping epitopes demonstrated potent toxin neutralization in mouse models of botulism. No serious adverse events were seen when these were tested in phase I clinical trials [17,18,19,20]. However, an oligoclonal antibody product, especially one where all antibodies bind the same protein, poses development challenges. For example, characterization of the product with respect to the ratio of each antibody, either in the product or in samples from pharmacokinetic studies, required the creation of unique assay reagents. For manufacturing, all the antibody components must be co-formulated and stable. To streamline the development of BoNT antitoxins, we sought to achieve the potency of multiple mAb combinations in a single IgG-based molecule with a long serum half-life.

We designed, expressed, and purified three TeAbs to BoNT/B, BoNT/E, and BoNT/F with good expression and high purity. The choice of location within the TeAb of the three mAb binding domains was relatively arbitrary but, in some instances, needed to be changed based on low expression yields or low purity of the TeAb. With respect to the affinity of the individual binding domains, we did observe a variable and usually minor impact on affinity based on the location of the variable domains. Affinity of the inner binding domains was either unchanged (TeAb-B) or decreased three- (TeAb-E) to five-fold (TeAb-F) compared with the parental IgG. Where a relatively minor decrease in affinity was observed, this could be due to steric hindrance by the outer binding domain. In the case of the outer binding domain, affinity was two- to six-fold lower for the TeAb compared with the parental IgG for reasons that are unclear. In the case of the scFv binding domain, the impact of location resulted in minor two-fold lower (TeAb-B) to two-fold higher (TeAb-E) affinities compared with the parental IgG. However, the TeAB-F scFv had a 66-fold lower affinity due to both a reduction in the on rate and an increase in the off rate. It has been observed that some scFvs are less stable than their IgG due to lower V-domain affinity, and this may explain the reduction we observed [39]. Affinity for holotoxin is generally higher in the TeAb constructs than the IgG when measured by flow fluorimetry in KinExA due to the ability of the TeAbs to bind multiple epitopes, resulting in avidity. How this compares to affinity in vivo when the three IgGs are combined is unclear as avidity due to cross-linking of multiple BoNTs can occur.

TeAb-B, TeAb-E, and TeAb-F had comparable potency to three mAb combinations against BoNT/B, BoNT/E, and BoNT/F, extending the results we previously reported for a TeAb to BoNT/A, thus demonstrating the generalizability of this approach.

## 4. Conclusions

The tri-epitopic antibodies reported here present an alternative to the three-IgG co-formulations used in the investigational botulinum antitoxin therapeutics. A single molecule in place of three IgGs simplifies bioanalytics and may simplify manufacturing the drug product. Further efforts will require optimizing the manufacturing and formulation of these molecules.

## 5. Materials and Methods

### 5.1. Design, Cloning, Expression, and Purification of TeAbs

Based on experience with the TeAb-K production for BoNT/A, we designed and constructed three plasmids for TeAb-B, TeAb-E, or TeAb-F mammalian cell expression. We used our human IgG1 production vector N5KG1Val-Lark as the starting vector backbone and inserted sequentially, as detailed below, two tandem VH, two tandem VK, and the third scFv format domain V genes into this vector. All the V genes encoded the same amino acid as those in the three parental toxin binders for each serotype. However, DNA was code-optimized for mammalian cell expression and with specific restriction enzyme sites engineered during gene synthesis. For the plasmids used here, the tandem light and heavy chains and scFv domain genes of each TeAb were driven by separate human CMV-promoters in the same plasmid.

For TeAb-B production, the V domain genes corresponding to the parental B binders were 1B10.1, 2B18.2, and 2B23.1 [36]. The parental BoNT/E binders for TeAb-E production were 3E2, 4E17.1, and 3E6.2 [16]. And the parental F binders for TeAb-F production were 6F5.4, Hu6F11, and Hu6F13.4 [35]. A cartoon of the construct is shown in Figure 1.

The tandem 2VH or tandem 2VK or scFv V genes together with CK1 domain genes were optimized for mammalian cell expression and synthesized by GenScript with unique franking subcloning sites, matching those sites in the N5KG1Val-Lark vector, i.e., Mlu I/NheI for 2VH fragment insertion, Dra III/BsiWI for 2VK fragment insertion, and BsiWI/EcoRI for scFv V genes together with CK1 domain gene fragment insertion. At the protein level, two V domains for each BoNT serotype were connected by VH or Vk linker sequences: a 14 amino acid linker1 with sequence TVAAPSVFIFPPSD for Vk before connection to the light chain constant domain or linker2 with the amino acid sequence ASTKGPSVFPLAPS [37] for VH before connection to the first heavy chain constant domain. In the case of TeAb-F, the amino acid linker1 had the sequence TVAAPSVFIFPPS. The third V domain in the format of scFv from the original pYD2 or pYD4 vector was linked to the CK1-C terminal end using a 20 amino acid linker3 SGGSTSGSGKPGSGEGSSGS [37].

For each TeAb expression plasmid construction, using routine T4 ligation and *E. coli* DH5 α competent cell transformation, the tandem 2VH genes were inserted into the empty vector N5KG1Val-Lark first, then the 2VK genes were inserted into the plasmid with correct 2VH genes in this vector after sequence verification, then the third scFv V genes together with CK1 domain genes were subcloned into the plasmid containing the correct tandem 2VH and tandem 2Vk genes. Plasmids used for sequencing were prepared from transformed *E. coli* clones at each step using a MiniPrep Kit (Qiagen, Valencia, CA, USA).

All V genes and each linker gene sequence in the TeAb expression vectors were confirmed to be as designed and in frame with the leader sequence and with the constant domain gene. Sequencing of the inserted fragments in the vector plasmid was performed from upstream of the leader sequence, the middle of the linker genes, and downstream of the constant domain genes with multiple primers for each TeAb plasmid. After final sequencing verification, clones with all the correct V genes, linkers, and leader sequences were selected for plasmid preparation using Qiagen MaxiPrep or MegaPrep Kit, and plasmids were used to transform either HEK293 cells for transient TeAb expression or CHO DG44 cells for stable TeAb production. Serum-free chemically defined media similar to those previously reported for human IgG1 production was used [40].

Stably transfected CHO DG44 cells were selected using the G418 resistant marker as previously described for IgG1 production [15]. The transfected cells were cultured and expanded into a 1L spinner flask for TeAb production. Supernatant from the cell cultures was collected and filtered with 0.22 µm filters before loading onto HiTrap Protein G HP columns (GE Healthcare Marlborough, MA 01752, USA) for protein purification as described [41,42]. Purified TeAbs were quantified via SDS-PAGE and absorbance at 280 nm and used immediately or stored at −80 °C prior to further characterization.

### 5.2. Protein Expression

The TeAb-B, TeAb-E, and TeAb-F were expressed transiently in HEK293 cells for small quantities and stably in CHO DG44 cells for larger quantities. Plasmids containing each of the correct TeAb genes in the N5KG1Val-Lark vector were used to transfect HEK293 cells via Lipofectamine 2000 in the Invitrogen^TM^ FreeStyle293 system (ThermoFisher, Waltham, MA, USA) according to the manufacturer’s instructions, and supernatant from the transformed cells was harvested three days after transfection. HEK293 cells were maintained in FreeStyle 293 Growth Medium before transfection and changed into FreeStyle 293 expression media for TeAb production. Small quantities of TeAb purified from supernatant via Protein G affinity column were used for in vitro characterization before TeAb production in CHO cell lines. The transiently produced TeAb was tested for BoNT binding by FACS, for molecular size by SDS-PAGE gel analysis, and for aggregation by visible inspection. TeAbs meeting binding and purity requirements were expressed in CHO DG44 cells by electroporating plasmid into cells [24].

CHO cells were maintained in serum-free suspension culture in CHO S SFM II medium (Gibco, Carlsbad, CA, USA), and TeAb-expressing cells as a pool were selected using G418 containing media after electroporation for 2~6 months. Methotrexate (MTX) amplification was used for the stable cell lines to generate sufficient material for mouse studies. The production level of TeAb-B from the stable cell line was 5–10 mg/L with a purity of over 90%. The yield for TeAb-E and TeAb-F was lower at 0.5–1 mg/L, when using MTX amplification.

### 5.3. Binding Affinity Determinations Using KinExA Analysis

Equilibrium dissociation constants (K_D_) and association and dissociation rate constants (*k_on_*, *k_off_*) of TeAbs for holotoxin and recombinant BoNT domains were measured using a KinExA 3200 flow fluorimeter (Sapidyne Instruments Inc., Boise, ID, USA) as described in [24,42,43]. Binding affinity assays were performed in duplicate; equilibrium titration data were fit to a 1:1 reversible binding model using KinExA Pro Software (version 4.2.12; Sapidyne Instruments) to determine the K_D_ values. The exponential decrease in the concentration of free antigen as a function of time was fit to a standard bimolecular rate equation using the KinExA Pro software to determine the *k_on_* [24]. The *k_off_* was calculated from the product of *k_on_* × *K*_D_.

For K_D_ measurements of TeAbs, each of the three individual IgGs were coupled to beads in three separate experiments, measuring the amount of free holotoxin (BoNT/B1, BoNT/E3, or BoNT/F1 from Metabiologics Inc., Madison, WI, USA) present for each specific epitope and calculating a K_D_ value for that epitope on the toxin. The resulting K_D_ reflected the avidity measured for that epitope on the holotoxin by the specific epitope being measured, as well as through the other epitopes present on the TeAb. Please see reference [24] for details.

It was not possible to measure K_D_ values with holotoxin coupled to the beads, due to the large amounts of toxin that would have been required for bead coupling (>200 μg) and the fact that coupling significantly damaged the holotoxin epitopes.

### 5.4. Mouse Neutralization Assay (MNA) of BoNT/B1, BoNT/E3, or BoNT/F1 by IgG or TeAb

Animal use protocol for BoNT mouse bioassays (Protocol #15-8) was approved by the Western Regional Research Center Institutional Animal Care and Use Committee (WRRC-IACUC) on 27 July 2015, and is renewed every three years. The most recent protocol, #24-8, was approved 11 July 2024.

TeAb; individual IgGs 1B10.1, 2B18.2, 2B23.1 or 3E2, 4E17.1, 3E6.2 or hu6F11, 6F5.4, and hu6F13.4; or a combination of two or three IgGs (1–50 μg total antibody per mouse) were added to an indicated number of mouse LD_50_ of BoNT/B1, BoNT/E3, or BoNT/F1 holotoxins (Metabiologics Inc. Madison, WI, USA) in gelatin phosphate buffer (pH 6.5) and incubated at room temperature for 30 min. For IgG, antibodies were added in equimolar ratios. The mixtures were injected intraperitoneally (i.p.) into groups of five or ten female CFW, Swiss Webster mice. Mice were observed at 4–8 h intervals after toxin challenge. Mouse survival was recorded for 5 days after injection.

## Figures and Tables

**Table 1:** Affinities and kinetic constants of 1B10.1, 2B18.2, 2B23.1, and TeAb-B for BoNT/B1 as measured by flow fluorimetry in KinExA.

	BoNT/B1 Domain			BoNT/B1 Holotoxin		
mAb	K_D_ (pM) (95% Confidence Interval)	*k_on_* (10^6^ M^−1^s^−1^) (95% Confidence Interval)	*k_off_* (10^−6^ s^−1^)	K_D_ (pM) (95% Confidence Interval)	*k_on_* (10^6^ M^−1^ s^−1^) (95% Confidence Interval)	*k_off_* (10^−6^ s^−1^)
LC-H_N_ (1B10.1 epitope)						
1B10.1	0.473 (0.906–0.202)	2.324 (4.040–1.078)	1.099	0.331 (1.180–0.038)	1.903 (2.026–1.788)	0.630
TeAb-B	0.965 (1.960–0.449)	0.414 (0.437–0.392)	0.399	0.402 (0.846–0.143)	0.875 (0.940–0.811)	0.352
LC-H_N_ (2B18.2 epitope)						
2B18.2	5.12 (8.05–3.22)	3.036 (4.149–2.101)	15.54	56.88 (69.68–45.94)	1.267 (1.368–1.174)	72.09
TeAb-B	20.53 (24.56–16.52)	0.691 (0.749–0.636)	14.186	92.84 (214.16–20.05)	1.146 (1.217–1.078)	106.4
LC-H_N_ (2B23.1 epitope)						
2B23.1	38.45 (48.60–30.91)	1.783 (2.270–1.348)	68.54	38.07 (52.12–24.62)	0.679 (0.707–0.651)	25.83
TeAb-B	18.46 (23.22 –14.40)	0.505 (0.525–0.485)	9.325	0.254 (0.628–0.049)	1.458 (1.621–1.313)	0.370

Variable domain 1 (inner) = mAb 1B10.1, variable domain 2 (outer) = mAb 2B18.2, and variable domain 3 (scFv) = mAb 2B23.1. Raw data for K_D_ and kinetics calculations are presented in Figure S1.

**Table 2:** Affinities and kinetic constants of 3E2, 4E17.1, 3E6.2, and TeAb-E for BoNT/E3, as measured by flow fluorimetry in KinExA.

	BoNT/E3 Domain			BoNT/E3 Holotoxin		
mAb	K_D_ (pM) (95% Confidence Interval)	*k_on_* (M^−1^s^−1^) (95% Confidence Interval)	*k_off_* (s^−1^)	K_D_ (pM) (95% Confidence Interval)	*k_on_* (M^−1^s^−1^) (95% Confidence Interval)	*k_off_* (s^−1^)
	LC-H_N_ (3E2 epitope)					
3E2	22.09 (30.26–14.20)	3.954 (6.450–2.429)	87.35	2.28 (2.71–1.89)	1.080 (1.253–0.927)	2.461
TeAb-E	7.48 (21.60–3.82)	0.186 (0.257–0.109)	1.391	1.18 (2.99–0.256)	0.3195 (0.3469–0.2937)	0.377
	LC-H_N_ (4E17.1 epitope)					
4E17.1	115.63 (136.72–88.91)	5.737 (8.527–4.364)	663.4	239.58 (281.13–181.42)	0.797 (0.886–0.715	190.9
TeAb-E	18.15 (51.22–9.53)	0.083 (0.089–0.077)	1.506	0.428 (1.21–0.042)	0.283 (0.299–0.268)	0.121
	LC-H_N_ (3E6.2 epitope)					
3E6.2	6.71 (9.28–4.62)	8.314 (11.58–6.083)	55.79	8.55 (14.32–4.5)	2.136 (2.705–1.687)	18.27
TeAb-E	13.87 (42.39–7.52)	0.089 (0.094–0.086)	1.246	0.851 (1.58–0.439)	0.329 (0.339–0.319)	0.28

Variable domain 1 (inner) = mAb 3E2, variable domain 2 (outer) = mAb 4E17.1, and variable domain 3 (scFv) = mAb 3E6.2. Raw data for K_D_ and kinetics calculations are presented in Figure S2.

**Table 3:** Affinities and kinetic constants of Hu6F13.4, 6F5.4, and hu6F11 and TeAb-F for BoNT/F1 as measured by flow fluorimetry in KinExA.

	BoNT/F1 Domain			BoNT/F1 Holotoxin		
mAb	K_D_ (pM) (95% Confidence Interval)	*k_on_* (M^−1^s^−1^) (95% Confidence Interval)	*k_off_* (s^−1^)	K_D_ (pM) (95% Confidence Interval)	*k_on_* (M^−1^s^−1^) (95% Confidence Interval)	*k_off_* (s^−1^)
LC-H_N_ (Hu6F13.4 epitope)						
Hu6F13.4	27.01 (49.76–14.96)	4.062 (5.443–2.896)	109.7	80.47 (139.24–13.40)	4.129 (11.4–1.438)	332.2
TeAb-F	1790 (2500–755.38)	0.729 (0.963–0.579)	1305	15.5 (35.52–3.48)	52.86 (110.3 –47.65)	819.3
LC-H_N_ (6F5.4 epitope)						
6F5.4	2.40 (3.52–1.48)	18.37 (23.14–13.51)	44.09	189.63 (497.81–99.31)	0.973 (1.717–0.425)	184.5
TeAb-F	4.41 (9.86–1.46)	13.9 (59.7–3.82)	61.31	26.6 (68.91–5.44)	0.963 (1.141–0.806)	25.61
LC-H_N_ (Hu6F11 epitope)						
Hu6F11	25.1 (41.25–12.66)	1.154 (1.693–0.711)	28.95	0.348 (0.874–0.022)	1.035 (1.068–1.003)	0.360
TeAb-F	125.07 (181.2–80.1)	2.373 (3.300–1.706)	296.7	23.3 (65.15–3.01)	0.952 (0.998–0.907)	22.19

Variable domain 1 (inner) = mAb hu6F13.4, variable domain 2 (outer) = mAb 6F5.4, and variable domain 3 (scFv) = mAb hu6F11. Raw data from K_D_ and kinetics calculations are presented in Figure S3.

**Table 4:** Mouse neutralization assay of BoNT/F1 with TeAb-F or parental three-IgG mixture.

Mabs Administered (50 µg/Mouse)	Amount of BoNT/F1 Administered (Mouse LD_50_)	Number of Mice Surviving/Number of Mice Treated
3 IgG: 6F5.4, hu6F11, hu6F13.4	20,000	10/10
3 IgG:6F5.4, hu6F11, hu6F13.4	40,000	5/5
TeAb-F	40,000	5/5
TeAb-F	20,000	5/5
TeAb-F	10,000	5/5

## Data Availability

The original contributions presented in this study are included in the article/Supplementary Materials. Further inquiries can be directed to the corresponding author(s).

## Supplementary Materials

The following supporting information can be downloaded at https://www.mdpi.com/article/10.3390/toxins17060281/s1: Figure S1: Raw data for K_D_ calculations of BoNT/B binders. Figure S2: Raw data for K_D_ calculations of BoNT/E binders. Figure S3: Raw data for K_D_ calculations of BoNT/F binders.
