# Supplementary material for: Single Tri-Epitopic Antibodies (TeAbs) to Botulinum Neurotoxin Serotypes B, E, and F Recapitulate the Full Potency of a Combination of Three Monoclonal Antibodies in Toxin Neutralization
Source: Toxins (Basel). 2025 Jun 4;17(6):281. doi: 10.3390/toxins17060281 (PMC12197607; doi:10.3390/toxins17060281)

Experiment(x)

**Experiment Name:**	KD 1B10.1 vs 1B10.1 domain 092811	**Start Time:**	Tue Sep 27 10:38:48 2011
**Experiment Type:**	Equilibrium	**End Time:**	Wed Sep 28 11:15:34 2011
**Constant Binding Partner (CBP)**		**Buffer:**	pbs/bsa
**Molecular Concentration:**	5.00pM	**Label:**	aSV5-647
**Valency:**	1	**Label Conc:**	800.00ng/ml
**Binding Site Concentration:**	5.00pM		

Comments(x)

1B10.1 beads 9/15/11

1B10.1 domain 9/12/11

1B10.1 IgG 6/4/08

aSV5-647

meter: 1.1822

Timing(x)

Bead Handling (Custom Beads)

Draw Source	Time (sec)	Volume (uL)	Rate (mL/min)	Stir
Backflush	30	0	0.0000	
Buffer	20	500	1.5000	✓
Particle Reservoir	32	533	1.0000	✓
Buffer	40	333	0.5000	
Waste	5	25	0.3000	
Buffer	2	10	0.3000	
Buffer	20	0	0.0000	
Buffer	9	150	1.0000	

Sample Timing

Draw Source	Time (sec)	Volume (uL)	Rate (mL/min)	Time Stamp
Sample Set 1-13	2880	12000	0.2500	
Buffer	30	125	0.2500	
Inject	120	500	0.2500	
Buffer	30	125	0.2500	
Buffer	120	2000	1.0000	

Analysis(x)

Baseline / Endpoints:
to (sec) from beginning
to (sec) from end

Ignore	Binding Signal (V)	Concentration
	0.2662	100.00pM
✓	0.4097	50.00pM
✓	0.5414	25.00pM
	0.2425	12.50pM
	0.4137	6.25pM
	0.7890	3.13pM
	1.0216	1.56pM
	1.1300	781.25fM
	1.2185	390.63fM
	1.2729	195.31fM
	1.2886	97.66fM
	1.3030	48.83fM
	1.3274	24.41fM
	0.1874	100.00pM
	0.2065	50.00pM
	0.2208	25.00pM
	0.2817	12.50pM
	0.3947	6.25pM
	0.6654	3.13pM
	0.9750	1.56pM
	1.1202	781.25fM
	1.2149	390.63fM
	1.2674	195.31fM
✓	1.1864	97.66fM
	1.2587	48.83fM
	1.2958	24.41fM

**Kd:**	473.38fM
**Active CBP:**	4.93pM
**CBP % Activity:**	98.63
**Ratio:**	10.4175
**Sig 100%:**	1.30
**NSB:**	0.21
**%Error:**	2.33

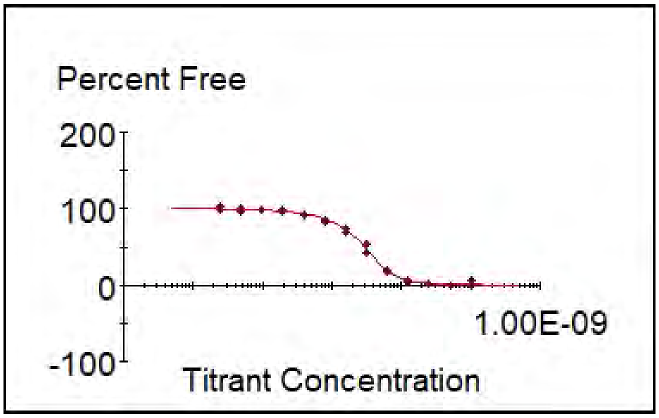

**Kd:**	473.38fM
**95% confidence interval**	
**Kd High:**	905.58fM
**Kd Low:**	201.86fM

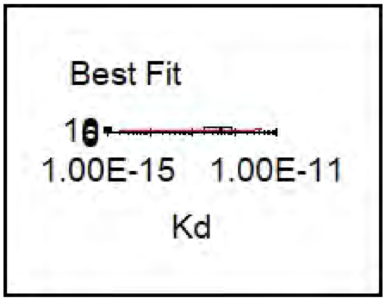

**Active CBP:**	4.93pM
**CBP %Activity:**	98.63
**95% confidence interval**	
**CBP High:**	6.10pM
**%Activity:**	122.02
**CBP Low:**	3.58pM
**%Activity:**	71.54

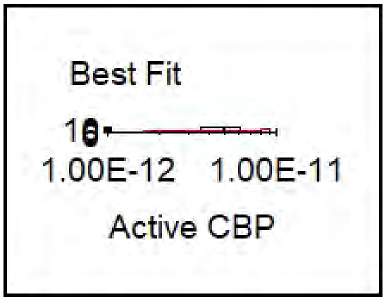

Data Traces(x)

Cycles: 2
Incubation delay (min): 0
Mix Time:

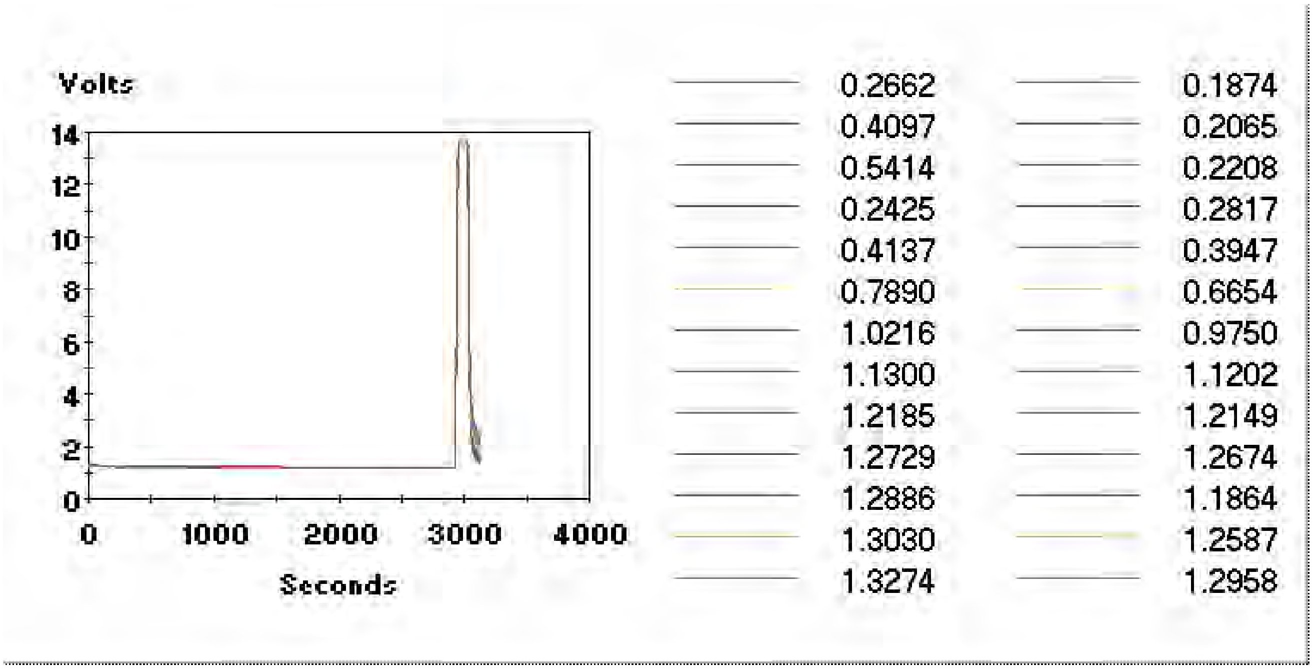

Experiment(x)

**Experiment Name:**	KD NXB10 CTR vs B1 020310	**Start Time:**	Mon Feb 1 17:49:05 2010
**Experiment Type:**	Equilibrium	**End Time:**	Tue Feb 2 18:27:54 2010
**Constant Binding Partner (CBP)**		**Buffer:**	pbs/bsa
**Molecular Concentration:**	4.00pM	**Label:**	B6.1-647
**Valency:**	1	**Label Conc:**	0
**Binding Site Concentration:**	4.00pM		

Comments(x)

1B10.1 beads 1/25/10 0.02% tween

B6.1-647

BoNT B1 100064 1/11/10

NXB10 IgG Control

meter: 0.9817

Timing(x)

Bead Handling (Custom Beads)

Draw Source	Time (sec)	Volume (uL)	Rate (mL/min)	Stir
Backflush	30	0	0.0000	
Buffer	20	500	1.5000	✓
Particle Reservoir	22	367	1.0000	✓
Buffer	40	333	0.5000	
Waste	5	25	0.3000	
Buffer	2	10	0.3000	
Buffer	20	0	0.0000	
Buffer	36	150	0.2500	

Sample Timing

Draw Source	Time (sec)	Volume (uL)	Rate (mL/min)	Time Stamp
Sample Set 1-13	2880	12000	0.2500	
Buffer	30	125	0.2500	
Inject	120	500	0.2500	
Buffer	30	125	0.2500	
Buffer	120	2000	1.0000	
Buffer	0	0	0.0000	

Analysis(x)

Baseline / Endpoints:
to (sec) from beginning
to (sec) from end

Ignore	Binding Signal (V)	Concentration
✓	0.1300	150.00pM
	0.0905	75.00pM
	0.1078	37.50pM
	0.0829	18.75pM
	0.1068	9.38pM
	0.2201	4.69pM
	0.3512	2.34pM
	0.4377	1.17pM
	0.5089	585.94fM
	0.5566	292.97fM
	0.5394	146.48fM
	0.5402	73.24fM
	0.5606	36.62fM
	0.0645	150.00pM
	0.0720	75.00pM
	0.0821	37.50pM
	0.0736	18.75pM
	0.0788	9.38pM
	0.1469	4.69pM
	0.3118	2.34pM
	0.4236	1.17pM
	0.4983	585.94fM
	0.5192	292.97fM
	0.5401	146.48fM
	0.5842	73.24fM
	0.5588	36.62fM

**Kd:**	331.11fM
**CBP:**	4.00pM
**Ratio:**	12.0806
**Titrant % Activity:**	94.3270
**Sig 100%:**	0.56
**NSB:**	0.07
**%Error:**	3.56

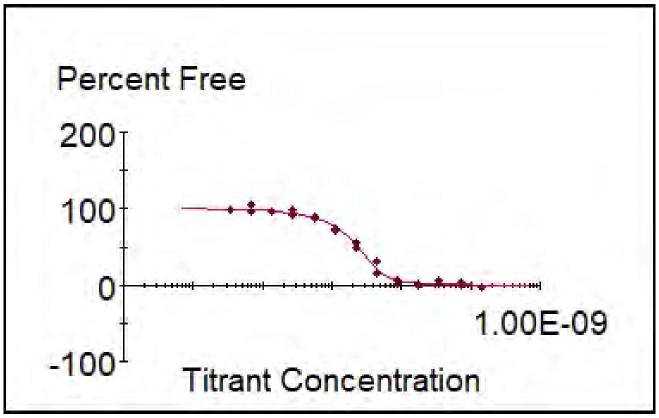

Kd: 331.11fM
95% confidence interval
Kd High: 1.18pM
Kd Low: 38.43fM

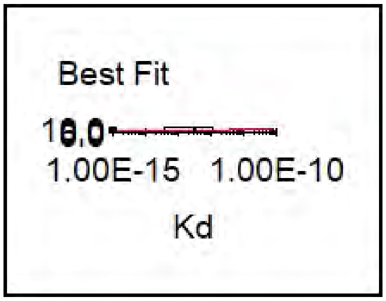

Titrant %Activity: 94.3270
95% confidence interval
Titrant %Activity High: 162.7931
Titrant %Activity Low: 68.0584

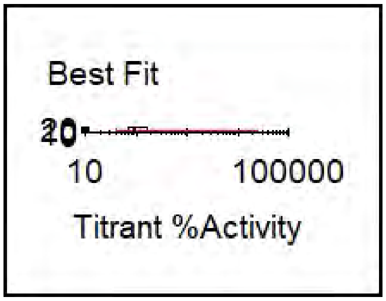

Data Traces(x)

**Cycles:**	2
**Incubation delay (min):**	0
**Mix Time:**	

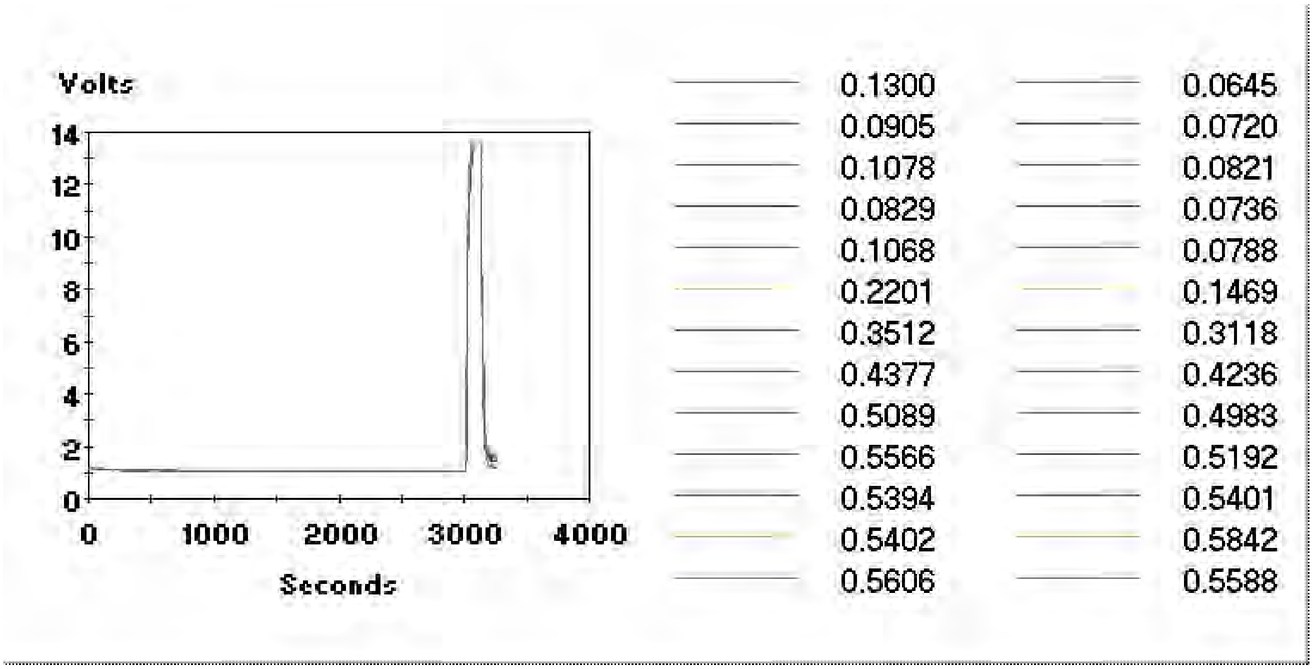

Experiment(x)

**Experiment Name:**	KD NXB18.1 vs B1 2009	**Start Time:**	Mon Feb 2 18:23:44 2009
**Experiment Type:**	Equilibrium	**End Time:**	Tue Feb 3 14:18:52 2009
**Constant Binding Partner (CBP)**		**Buffer:**	pbs/bsa
**Molecular Concentration:**	25.00pM	**Label:**	B6.1-647
**Valency:**	1	**Label Conc:**	800.00ng/ml
**Binding Site Concentration:**	25.00pM		

Comments(x)

2B18.3 beads 2/2/09

BoNT B1 2/2/09

NXB18 CB081208.01

B6.1-647 1:1000

meter: 0.6535

Timing(x)

Bead Handling (Custom Beads)

Draw Source	Time (sec)	Volume (uL)	Rate (mL/min)	Stir
Backflush	30	0	0.0000	
Buffer	20	500	1.5000	✓
Particle Reservoir	14	233	1.0000	✓
Buffer	40	333	0.5000	
Waste	5	25	0.3000	
Buffer	2	10	0.3000	
Buffer	20	0	0.0000	
Buffer	9	150	1.0000	

Sample Timing

Draw Source	Time (sec)	Volume (uL)	Rate (mL/min)	Time Stamp
Sample Set 1-13	1440	6000	0.2500	
Buffer	30	125	0.2500	
Inject	120	500	0.2500	
Buffer	30	125	0.2500	
Buffer	120	2000	1.0000	

Analysis(x)

Baseline / Endpoints:

to (sec) from beginning

to (sec) from end

Ignore	Binding Signal (V)	Concentration
	0.0854	5.00nM
	0.0968	2.50nM
	0.1250	1.25nM
	0.1891	625.00pM
	0.2720	312.50pM
	0.4339	156.25pM
	0.6537	78.13pM
	0.9367	39.06pM
	1.0508	19.53pM
	1.1686	9.77pM
	1.1802	4.88pM
	1.2792	2.44pM
	1.2834	1.22pM
	0.0858	5.00nM
	0.0959	2.50nM
	0.1308	1.25nM
	0.1748	625.00pM
	0.2813	312.50pM
	0.4499	156.25pM
	0.6694	78.13pM
	0.9067	39.06pM
	1.1029	19.53pM
	1.2067	9.77pM
	1.2922	4.88pM
	1.3224	2.44pM
	1.3433	1.22pM

**Kd:**	56.88pM
**Active CBP:**	37.61pM
**CBP %Activity:**	150.46
**Ratio:**	0.6613
**Sig 100%:**	1.24
**Drift (%/run):**	-0.4400
**NSB:**	0.08
**Drift (mV/run):**	988.8560
**%Error:**	1.32

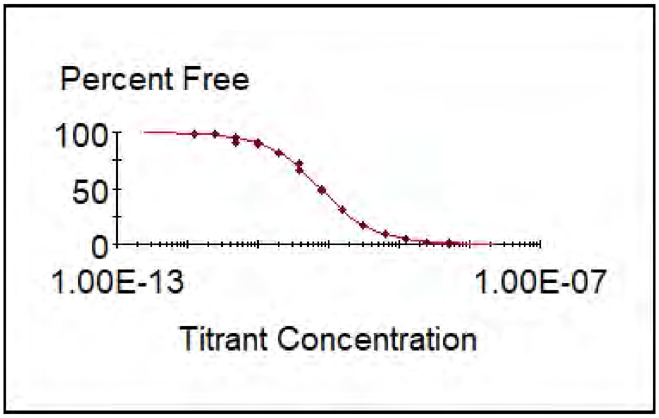

**Kd:**	56.88pM
**95% confidence interval**	
**Kd High:**	69.68pM
**Kd Low:**	45.94pM

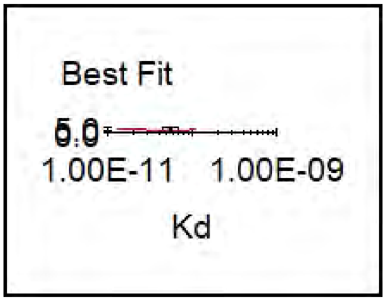

**Active CBP:**	37.61pM
**CBP %Activity:**	150.46
**95% confidence interval**	
**CBP High:**	66.14pM
**%Activity:**	264.54
**CBP Low:**	Less than 12.22pM
**%Activity:**	Less than 48.86

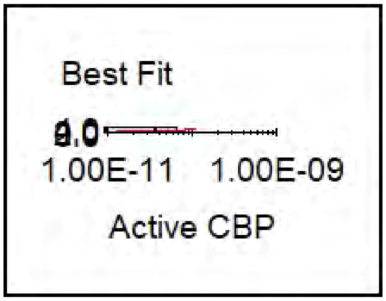

Data Traces(x)

Cycles: 2
Incubation delay (min): 360
Mix Time:

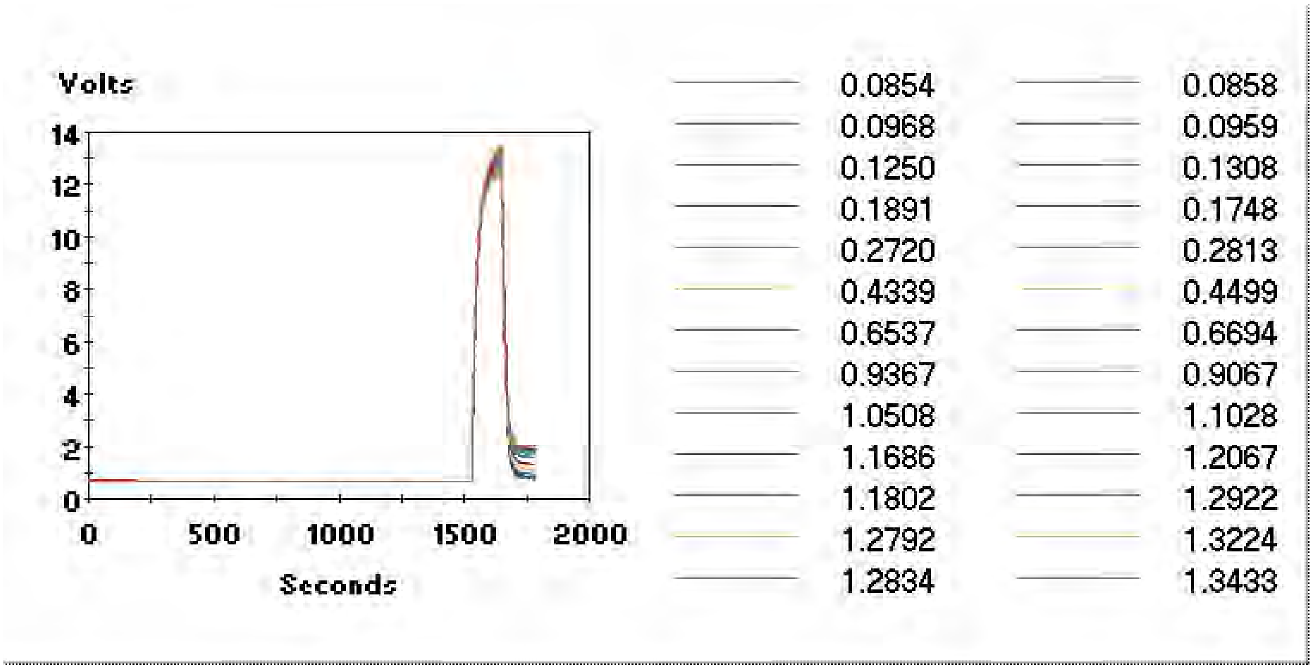

Experiment(x)

**Experiment Name:**	KD 2B18.2 & NXB18 domain 082424{2}	**Start Time:**	Sat Aug 24 21:31:41 2024
**Experiment Type:**	Equilibrium	**End Time:**	Sun Aug 25 09:16:59 2024
**Constant Binding Partner (CBP)**		**Buffer:**	PBS/BSA
**Molecular Concentration:**	20.00pM	**Label:**	Anti-SV5-647
**Valency:**	1	**Label Conc:**	0
**Binding Site Concentration:**	20.00pM		

Comments(x)

beads: 2B18.2 IgG 100ug on 1ml Sepharose 4B

sample volume: 7 ml

detection: Anti-His-647 (prepared by Lou 082424)

CBP: 20 pM NXB18 domain 082324

titrant: 2B18.2 IgG1

titration: 2 cycles, 15 samples: 4 nM - 977fM (1:2) plus NBS ctr and 20pM NXB18 domain CBPctr

samples:

3-15) titration

1): NBS

2): CBP NXB18 domain (20pM) only

3-15) 2B18.2 IgG serial dilution from 4 nM to 0.977 pM in CBP

beads: 2B18.2 IgG 100ug on 1ml Sepharose 4B

sample volume: 7 ml

detection: Anti-His-647 (prepared by Lou 082424)

CBP: 20 pM NXB18 domain 082324

titrant: 2B18.2 IgG1

titration: 2 cycles, 15 samples: 4 nM - 977fM (1:2) plus NBS ctr and 20pM NXB18 domain CBPctr

samples:

3-15) titration

1): NBS

2): CBP NXB18 domain (20pM) only

3-15) 2B18.2 IgG serial dilution from 4 nM to 0.977 pM in CBP

Timing(x)

Bead Handling (Custom Beads)

Draw Source	Time (sec)	Volume (uL)	Rate (mL/min)	Stir
Backflush	20	0	0.0000	
Buffer	20	500	1.5000	✓
Particle Reservoir 1	22	360	1.0000	✓
Buffer	30	500	1.0000	
Waste	2	8	0.2500	
Buffer	20	0	0.0000	
Buffer	9	150	1.0000	

Sample Timing

Draw Source	Time (sec)	Volume (uL)	Rate (mL/min)	Time Stamp
Sample Set 201-215	720	3000	0.2500	
Buffer	30	125	0.2500	
Rack 2: Tube 60	120	500	0.2500	
Buffer	30	125	0.2500	
Buffer	180	3000	1.0000	

Analysis(x)

Baseline / Endpoints:

to (sec) from beginning

to (sec) from end

Ignore	Binding Signal (V)	Concentration
	0.0311	NSB
✓	0.3345	0
	0.0313	4.00nM
	0.0334	2.00nM
✓	0.0406	1.00nM
	0.0327	500.00pM
	0.0525	250.00pM
	0.0358	125.00pM
	0.1082	62.50pM
	0.0891	31.25pM
	0.1261	15.63pM
	0.1683	7.81pM
	0.2000	3.91pM
✓	0.1924	1.95pM
	0.3295	976.56fM
	0.0164	NSB
✓	0.4496	0
	0.0163	4.00nM
	0.0178	2.00nM
✓	0.1123	1.00nM
	0.0196	500.00pM
	0.0269	250.00pM
	0.0363	125.00pM
	0.0450	62.50pM
	0.0722	31.25pM
	0.1160	15.63pM
	0.1584	7.81pM
	0.1756	3.91pM
✓	0.1840	1.95pM
	0.3054	976.56fM

**Kd:**	5.12pM
**Active CBP:**	5.12fM
**CBP %Activity:**	0.03
**Ratio:**	0.0010
**Sig 100%:**	0.37
**Drift (%/run):**	0.2531
**NSB:**	0.03
**Drift (mV/run):**	-0.8896
**TR NSB:**	9.74e-08
**%Error:**	3.89

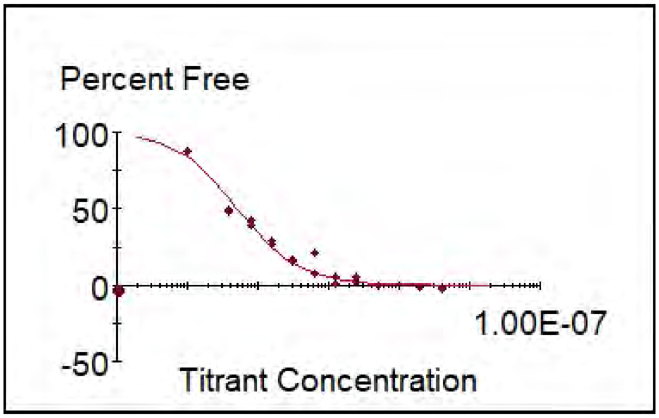

**Kd:**	5.12pM
**95% confidence interval**	
**Kd High:**	8.05pM
**Kd Low:**	3.22pM

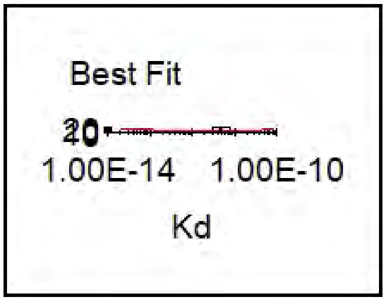

**Active CBP:**	5.12fM
**CBP %Activity:**	0.03
**95% confidence interval**	
**CBP High:**	Greater than 1.42pM
**%Activity:**	Greater than 7.09
**CBP Low:**	Less than 18.50aM
**%Activity:**	Less than 0.00

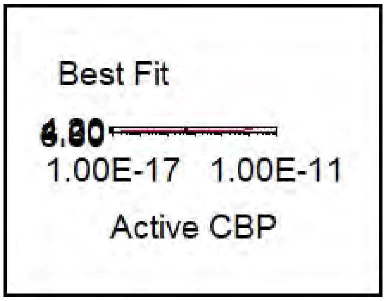

Data Traces(x)

Cycles: 2
Incubation delay (min): 0
Mix Time:

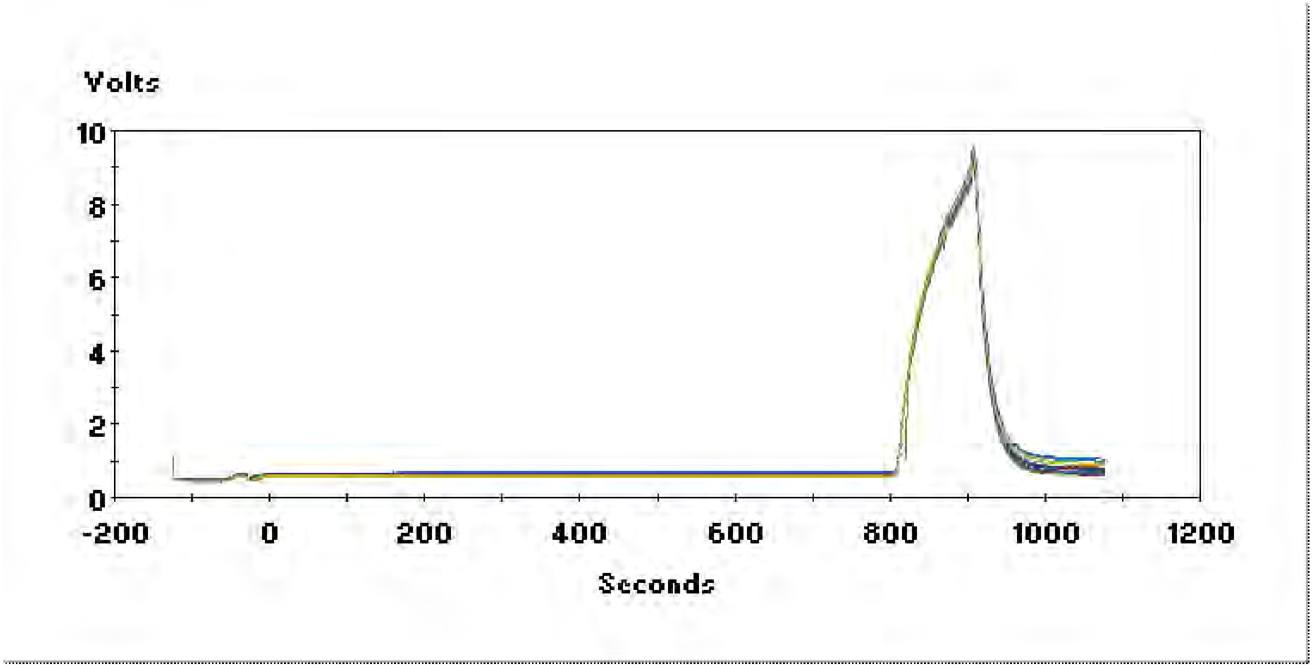

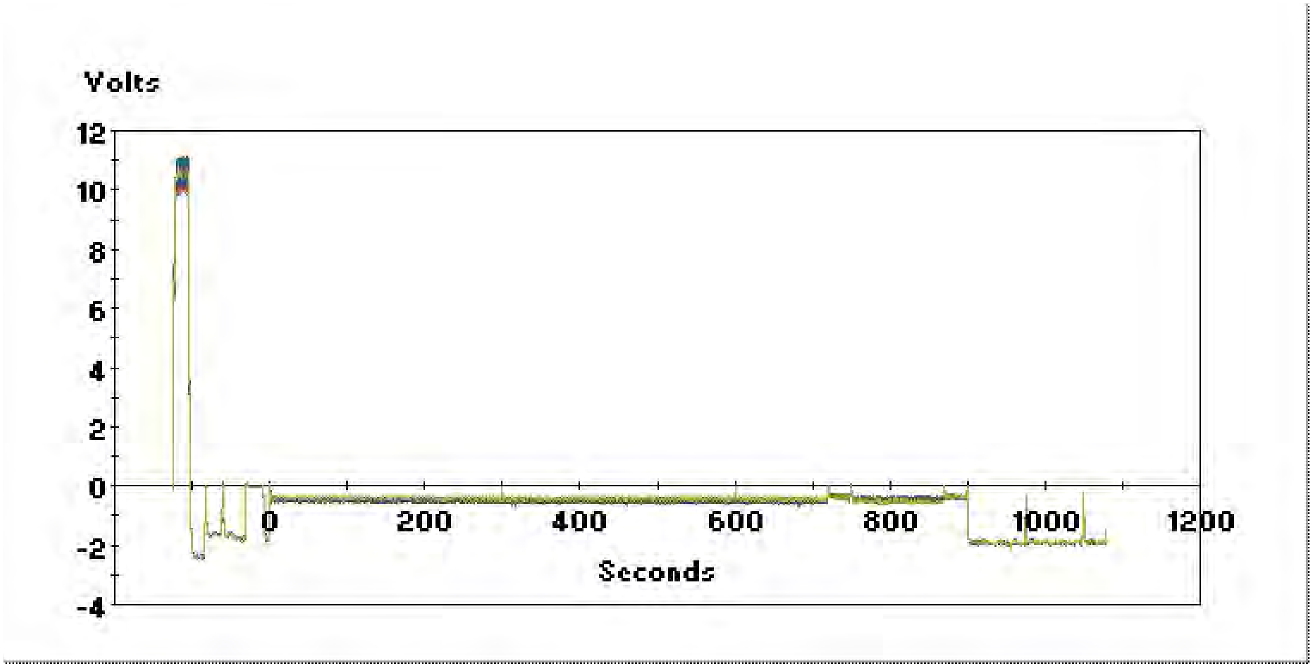

Experiment(x)

**Experiment Name:**	KD NXB23gly vs B1 121409	**Start Time:**	Fri Dec 11 17:30:59 2009
**Experiment Type:**	Equilibrium	**End Time:**	Sat Dec 12 02:15:36 2009
**Constant Binding Partner (CBP)**		**Buffer:**	pbs/bsa
**Molecular Concentration:**	30.00pM	**Label:**	B6.1-647
**Valency:**	1	**Label Conc:**	800.00ng/ml
**Binding Site Concentration:**	30.00pM		

Comments(x)

2B23 beads 12/10/09

BoNT B1 100064 11/3/09

NXB23 glycosylated IgG 5557566-135.1

B6.1-647

meter: 1.0222

Timing(x)

Bead Handling (Custom Beads)

Draw Source	Time (sec)	Volume (uL)	Rate (mL/min)	Stir
Backflush	30	0	0.0000	
Buffer	20	500	1.5000	✓
Particle Reservoir	22	367	1.0000	✓
Buffer	40	333	0.5000	
Waste	5	25	0.3000	
Buffer	2	10	0.3000	
Buffer	20	0	0.0000	
Buffer	9	150	1.0000	

Sample Timing

Draw Source	Time (sec)	Volume (uL)	Rate (mL/min)	Time Stamp
Sample Set 1-13	720	3000	0.2500	
Buffer	30	125	0.2500	
Inject	120	500	0.2500	
Buffer	30	125	0.2500	
Buffer	120	2000	1.0000	

Analysis(x)

Baseline / Endpoints:
to (sec) from beginning
to (sec) from end

	Binding	
Ignore	Signal (V)	Concentration
	0.1431	2.00nM
	0.1424	1.00nM
	0.1752	500.00pM
	0.2500	250.00pM
	0.4767	125.00pM
	0.5781	62.50pM
	0.8309	31.25pM
	1.0497	15.63pM
	1.1771	7.81pM
	1.2620	3.90pM
	1.2819	1.95pM
	1.3846	976.56fM
	1.3238	488.28fM
	0.0924	2.00nM
	0.1495	1.00nM
	0.1569	500.00pM
	0.2425	250.00pM
	0.3920	125.00pM
	0.5960	62.50pM
	0.8574	31.25pM
	1.0492	15.63pM
	1.2008	7.81pM
	1.3251	3.90pM
	1.3601	1.95pM
	1.4147	976.56fM
	1.3849	488.28fM

Kd: 38.07pM
Active CBP: 7.64pM
CBP %Activity: 25.47
Ratio: 0.2007
Sig 100%: 1.39
NSB: 0.09
%Error: 2.21

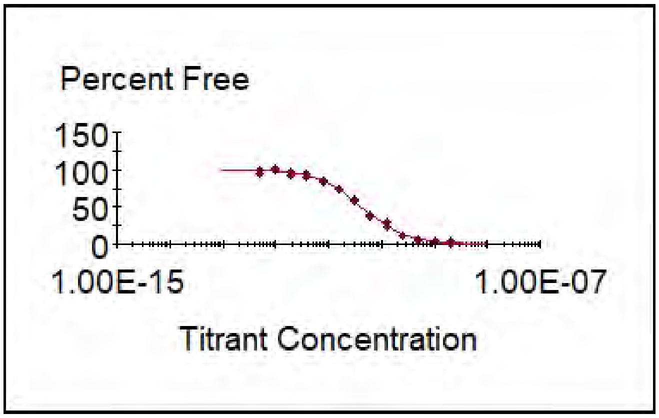

Kd: 38.07pM
95% confidence interval
Kd High: 48.28pM
Kd Low: 25.82pM

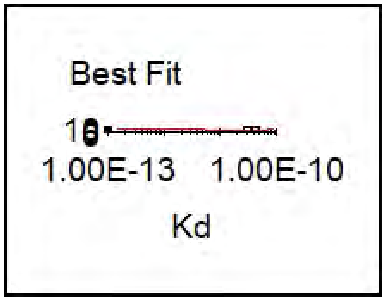

Active CBP: 7.64pM
CBP %Activity: 25.47
95% confidence interval
CBP High: 37.26pM
%Activity: 124.21
CBP Low: Less than 27.60fM
%Activity: Less than 0.09

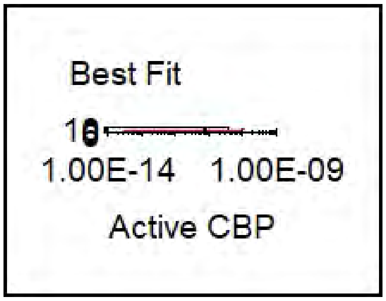

Data Traces(x)

Cycles: 2
Incubation delay (min): 0
Mix Time:

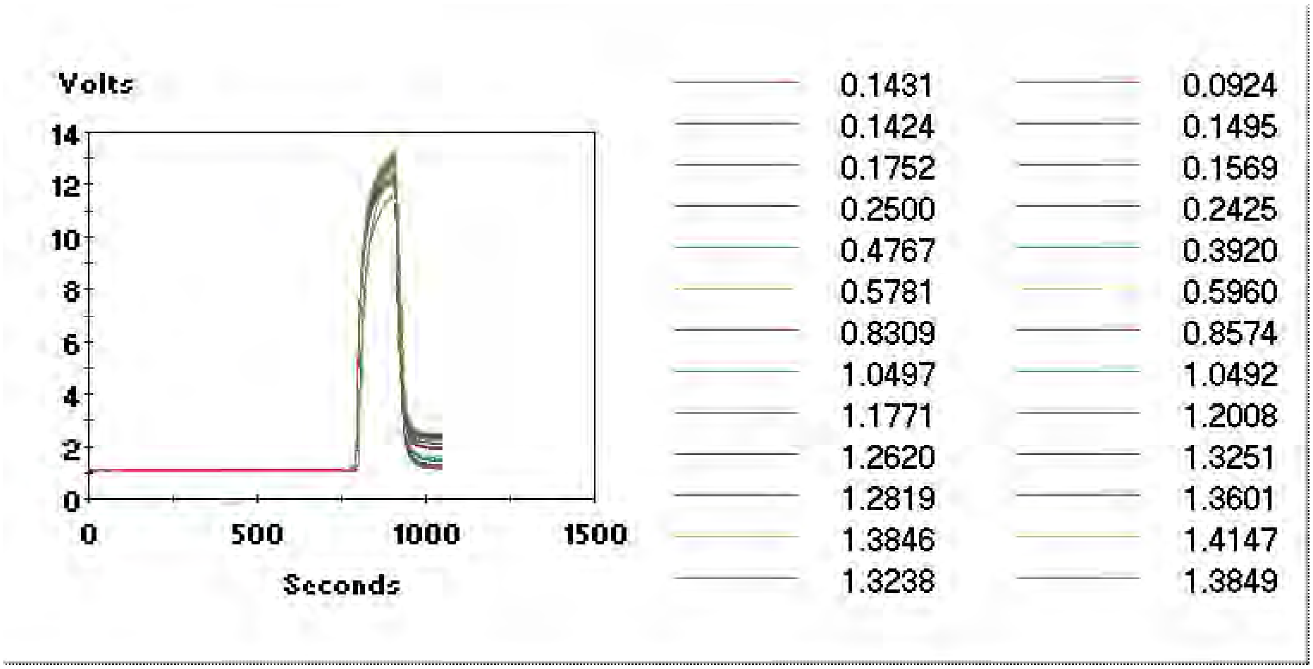

Experiment(x)

**Experiment Name:**	Kd 2B23 vs 2B23 domain 091611	**Start Time:**	Thu Sep 15 17:36:16 2011
**Experiment Type:**	Equilibrium	**End Time:**	Fri Sep 16 02:31:37 2011
**Constant Binding Partner (CBP)**		**Buffer:**	pbs/bsa
**Molecular Concentration:**	15.00pM	**Label:**	aSV5-647
**Valency:**	1	**Label Conc:**	800.00ng/ml
**Binding Site Concentration:**	15.00pM		

Comments(x)

2B23 beads 5/26/11

2B23 domain 9/12/11

2B23 IgG 2/5/08

aSV5-647

meter: 1.1755

Timing(x)

Bead Handling (Custom Beads)

Draw Source	Time (sec)	Volume (uL)	Rate (mL/min)	Stir
Backflush	30	0	0.0000	
Buffer	20	500	1.5000	✓
Particle Reservoir	34	567	1.0000	✓
Buffer	40	333	0.5000	
Waste	5	25	0.3000	
Buffer	2	10	0.3000	
Buffer	20	0	0.0000	
Buffer	9	150	1.0000	

Sample Timing

Draw Source	Time (sec)	Volume (uL)	Rate (mL/min)	Time Stamp
Sample Set 1-13	720	3000	0.2500	
Buffer	30	125	0.2500	
Inject	120	500	0.2500	
Buffer	30	125	0.2500	
Buffer	120	2000	1.0000	

Analysis(x)

Baseline / Endpoints:

to (sec) from beginning

to (sec) from end

Ignore	Binding Signal (V)	Concentration
	0.1099	2.00nM
	0.1224	1.00nM
	0.1448	500.00pM
	0.1749	250.00pM
	0.2408	125.00pM
	0.3091	62.50pM
	0.4104	31.25pM
	0.4826	15.63pM
	0.5188	7.81pM
	0.5608	3.90pM
	0.5700	1.95pM
	0.5738	976.56fM
	0.5535	488.28fM
	0.1250	2.00nM
	0.1334	1.00nM
	0.1515	500.00pM
	0.1747	250.00pM
	0.2262	125.00pM
	0.2936	62.50pM
	0.3592	31.25pM
	0.4137	15.63pM
✓	0.4049	7.81pM
	0.4824	3.90pM
	0.4747	1.95pM
	0.4743	976.56fM
	0.4624	488.28fM

**Kd:**	38.45pM
**Active CBP:**	11.86pM
**CBP %Activity:**	79.08
**Ratio:**	0.3085
**Sig 100%:**	0.57
**Drift (%/run):**	2.0367
**NSB:**	0.12
**Drift (mV/run):**	1.4040
**TR NSB:**	4.92e-07
**%Error:**	1.31

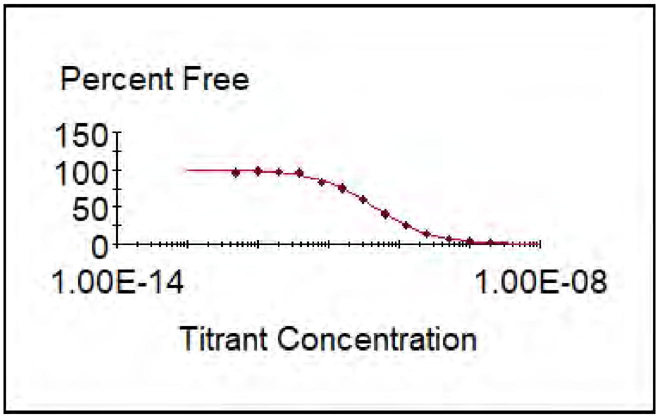

**Kd:**	38.45pM
**95% confidence interval**	
**Kd High:**	48.60pM
**Kd Low:**	30.91pM

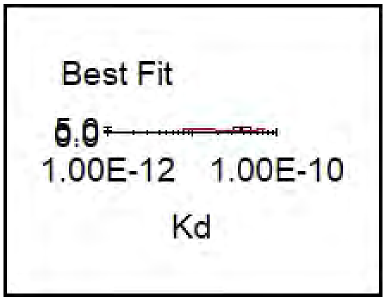

**Active CBP:**	11.86pM
**CBP %Activity:**	79.08
**95% confidence interval**	
**CBP High:**	30.99pM
**%Activity:**	206.57
**CBP Low:**	Less than 42.86fM
**%Activity:**	Less than 0.29

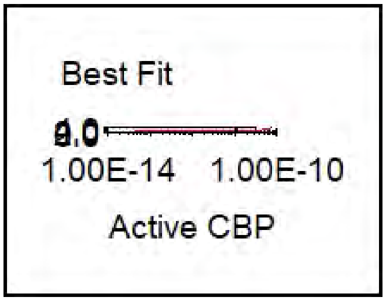

Data Traces(x)

Cycles: 2
Incubation delay (min): 0
Mix Time:

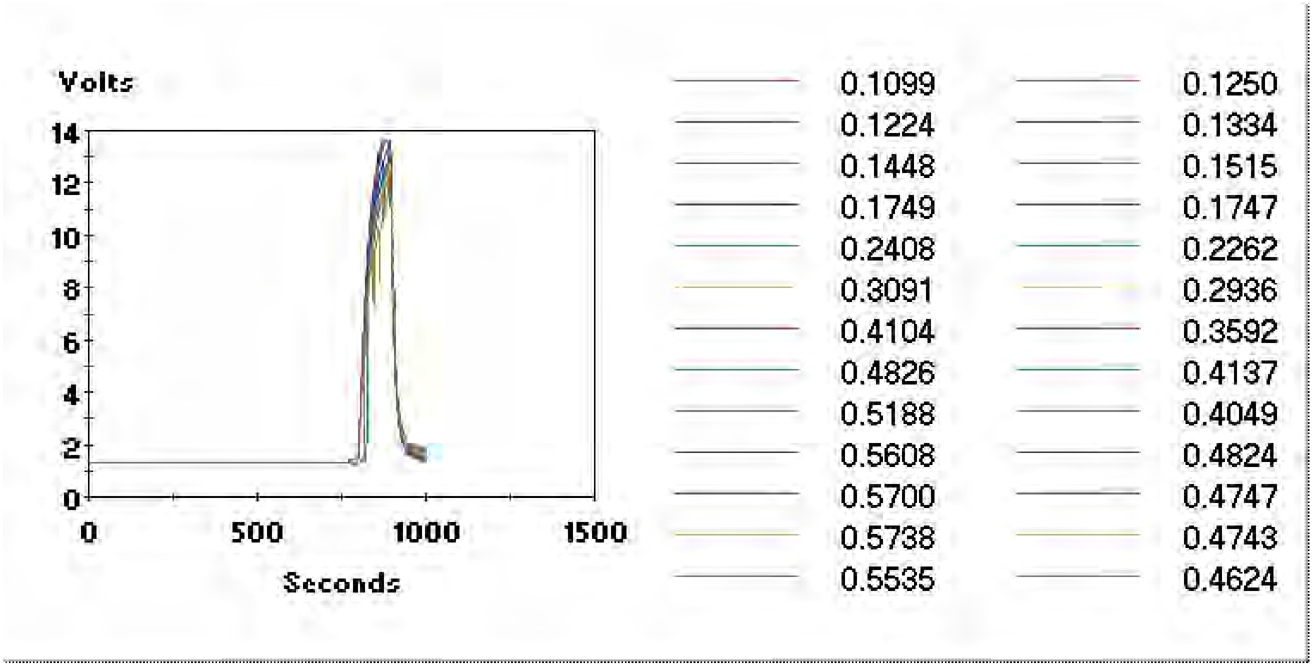

Experiment(x)

**Experiment Name:**	KD TsAb-B(1B10.1) vs B1 040915	**Start Time:**	Wed Apr 8 18:48:06 2015
**Experiment Type:**	Equilibrium	**End Time:**	Thu Apr 9 20:13:03 2015
**Constant Binding Partner (CBP)**		**Buffer:**	PBS/BSA
**Molecular Concentration:**	5.00pM	**Label:**	B6.1-647
**Valency:**	1	**Label Conc:**	0
**Binding Site Concentration:**	5.00pM		

Comments(x)

beads: XB10 4/8/15

sample volume: 10 ml

detection: B6.1-647

CBP: 5 pM BoNT B1 100065 3/20/15

titrant: TsAb-B 3/18/15 (260 kDa, 0.3 mg/ml, 1.154 uM)

titration: 13 samples: 200 pM - 48 fM (1:2); + B1 only

samples:

1) NSB

2) 100% (B1 only)

3-15) titration of TsAb-B

Timing(x)

Bead Handling (Custom Beads)

Draw Source	Time (sec)	Volume (uL)	Rate (mL/min)	Stir
Backflush	20	0	0.0000	
Buffer	20	500	1.5000	✓
Particle Reservoir 1	24	400	1.0000	✓
Buffer	30	500	1.0000	
Waste	2	8	0.2500	
Buffer	20	0	0.0000	
Buffer	9	150	1.0000	

Sample Timing

Draw Source	Time (sec)	Volume (uL)	Rate (mL/min)	Time Stamp
Sample Set 1,101-114	2400	10000	0.2500	
Buffer	30	125	0.2500	
Standards: Tube 3	120	500	0.2500	
Buffer	30	125	0.2500	
Buffer	90	1500	1.0000	

Analysis (x)

Baseline / Endpoints:

to (sec) from beginning

to (sec) from end

Ignore	Binding Signal (V)	Concentration
✓	0.1254	0
	0.6581	0
	0.1041	200.00pM
	0.1157	100.00pM
	0.1300	50.00pM
	0.1424	25.00pM
	0.1575	12.50pM
	0.2902	6.25pM
	0.4521	3.13pM
	0.5682	1.56pM
	0.6195	781.25fM
	0.6472	390.63fM
	0.6564	195.31fM
	0.6694	97.66fM
	0.6719	48.83fM
✓	0.1223	0
	0.6704	0
	0.1184	200.00pM
	0.1207	100.00pM
	0.1158	50.00pM
	0.1392	25.00pM
	0.1522	12.50pM
	0.2810	6.25pM
	0.4355	3.13pM
	0.5785	1.56pM
	0.6380	781.25fM
	0.6799	390.63fM
	0.6763	195.31fM
	0.6817	97.66fM
	0.6886	48.83fM

**Kd:**	402.02fM
**CBP:**	5.00pM
**Ratio:**	12.4371
**Titrant % Activity:**	72.2281
**Sig 100%:**	0.68
**NSB:**	0.11
**%Error:**	1.87

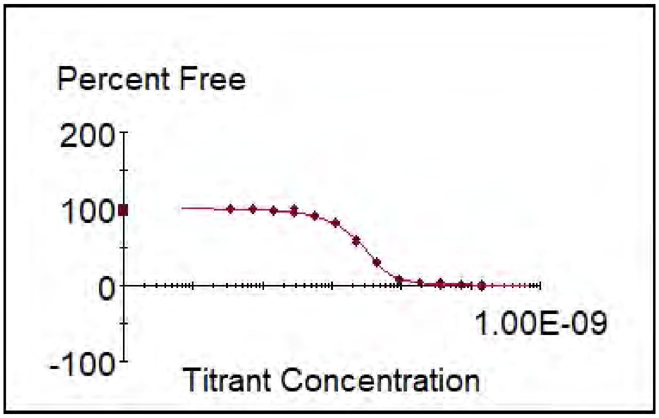

**Kd:**	402.02fM
**95% confidence interval**	
**Kd High:**	845.83fM
**Kd Low:**	143.00fM

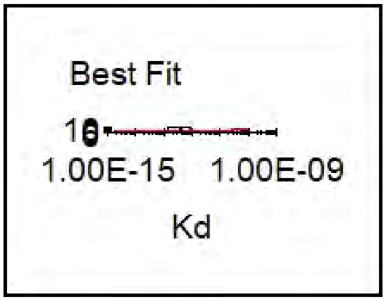

**Titrant %Activity:**	72.2281
**95% confidence interval**	
**Titrant %Activity High:**	91.7295
**Titrant %Activity Low:**	60.5837

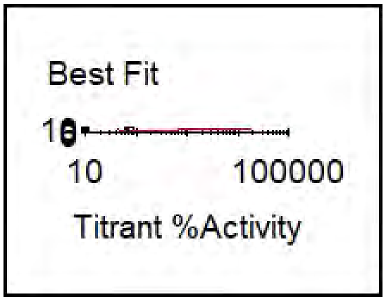

Data Traces(x)

Cycles: 2
Incubation delay (min): 0
Mix Time:

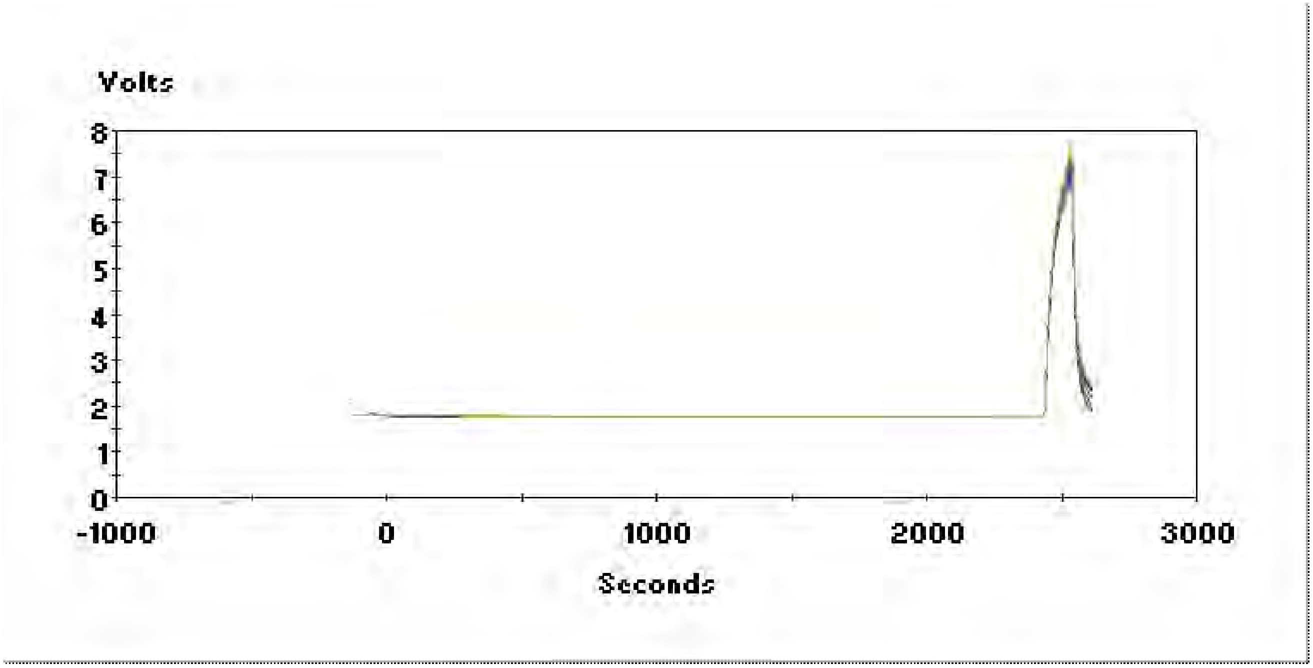

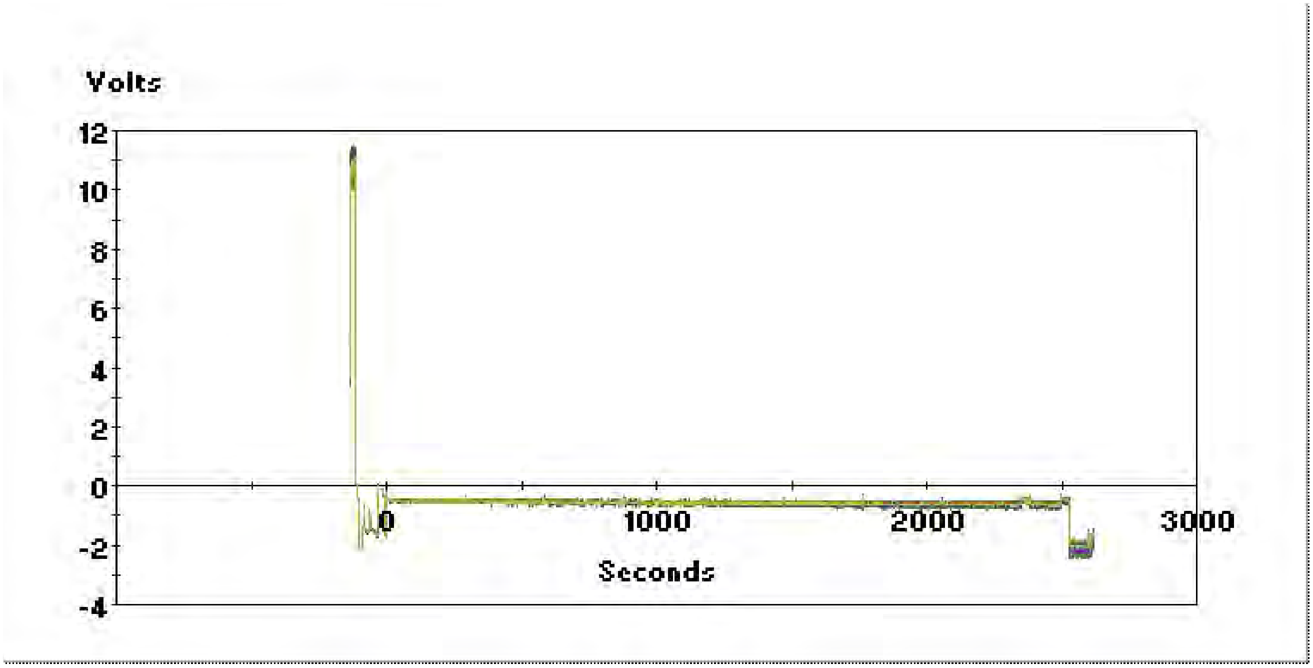

Experiment(x)

**Experiment Name:**	KD TsAb-B(1B10.1) vs LCHN-B10 080515	**Start Time:**	Tue Aug 4 16:10:41 2015
**Experiment Type:**	Equilibrium	**End Time:**	Wed Aug 5 04:47:06 2015
**Constant Binding Partner (CBP)**		**Buffer:**	PBS/BSA
**Molecular Concentration:**	7.00pM	**Label:**	aSV5-647
**Valency:**	1	**Label Conc:**	0
**Binding Site Concentration:**	7.00pM		

Comments(x)

beads: XB10 8/4/15

sample volume: 3 ml

detection: aSV5-647

CBP: 7 pM BoNT LCHN-B10 16907945-137 4/27/15

titrant: TsAb-B 5/5/15 (260 kDa, 1 mg/ml, 3.846 uM)

titration: 15 samples: 1 nM - 61 fM (1:2); + CBP only

samples:

1) NSB

2) 100% (CBP only)

3-15) titration

Timing(x)

Bead Handling (Custom Beads)

Draw Source	Time (sec)	Volume (uL)	Rate (mL/min)	Stir
Backflush	20	0	0.0000	
Buffer	20	500	1.5000	✓
Particle Reservoir 1	16	267	1.0000	✓
Buffer	30	500	1.0000	
Waste	2	8	0.2500	
Buffer	20	0	0.0000	
Buffer	9	150	1.0000	

Sample Timing

Draw Source	Time (sec)	Volume (uL)	Rate (mL/min)	Time Stamp
Sample Set 1,201-216	720	3000	0.2500	
Buffer	30	125	0.2500	
Standards: Tube 3	120	500	0.2500	
Buffer	30	125	0.2500	
Buffer	90	1500	1.0000	

Analysis(x)

Baseline / Endpoints:

to (sec) from beginning

to (sec) from end

Ignore	Binding Signal (V)	Concentration
✓	0.2933	0
	1.2040	0
	0.3002	1.00nM
	0.2989	500.00pM
	0.3140	250.00pM
	0.3279	125.00pM
	0.4474	62.50pM
	0.7149	31.25pM
	0.9043	15.63pM
	1.0467	7.81pM
	1.1262	3.91pM
	1.1762	1.95pM
	1.2132	976.56fM
	1.2157	488.28fM
✓	1.2876	244.14fM
	1.2221	122.07fM
	1.2435	61.04fM
✓	0.3144	0
	1.2204	0
	0.2988	1.00nM
	0.3197	500.00pM
	0.3301	250.00pM
	0.3137	125.00pM
	0.4242	62.50pM
	0.6890	31.25pM
	0.8906	15.63pM
	1.0371	7.81pM
	1.1241	3.91pM
	1.1612	1.95pM
	1.1767	976.56fM
	1.2010	488.28fM
	1.2047	244.14fM
	1.2138	122.07fM
	1.2182	61.04fM

**Kd:**	964.59fM
**CBP:**	7.00pM
**Ratio:**	7.2570
**Titrant % Activity:**	17.3650
**Sig 100%:**	1.21
**NSB:**	0.29
**%Error:**	1.66

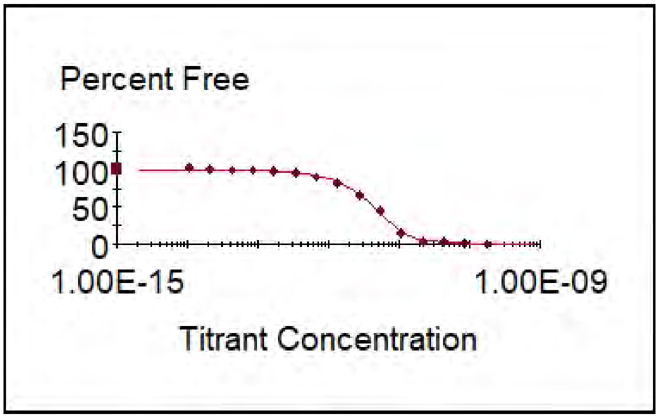

**Kd:**	964.59fM
**95% confidence interval**	
**Kd High:**	1.96pM
**Kd Low:**	448.86fM

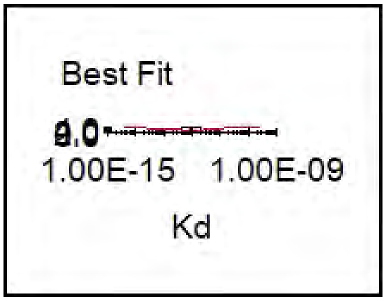

**Titrant %Activity:**	17.3650
**95% confidence interval**	
**Titrant %Activity High:**	23.8237
**Titrant %Activity Low:**	14.1384

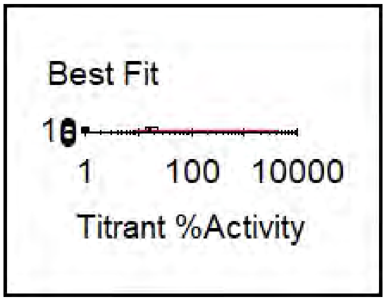

Data Traces(x)

Cycles: 2
Incubation delay (min): 0
Mix Time:

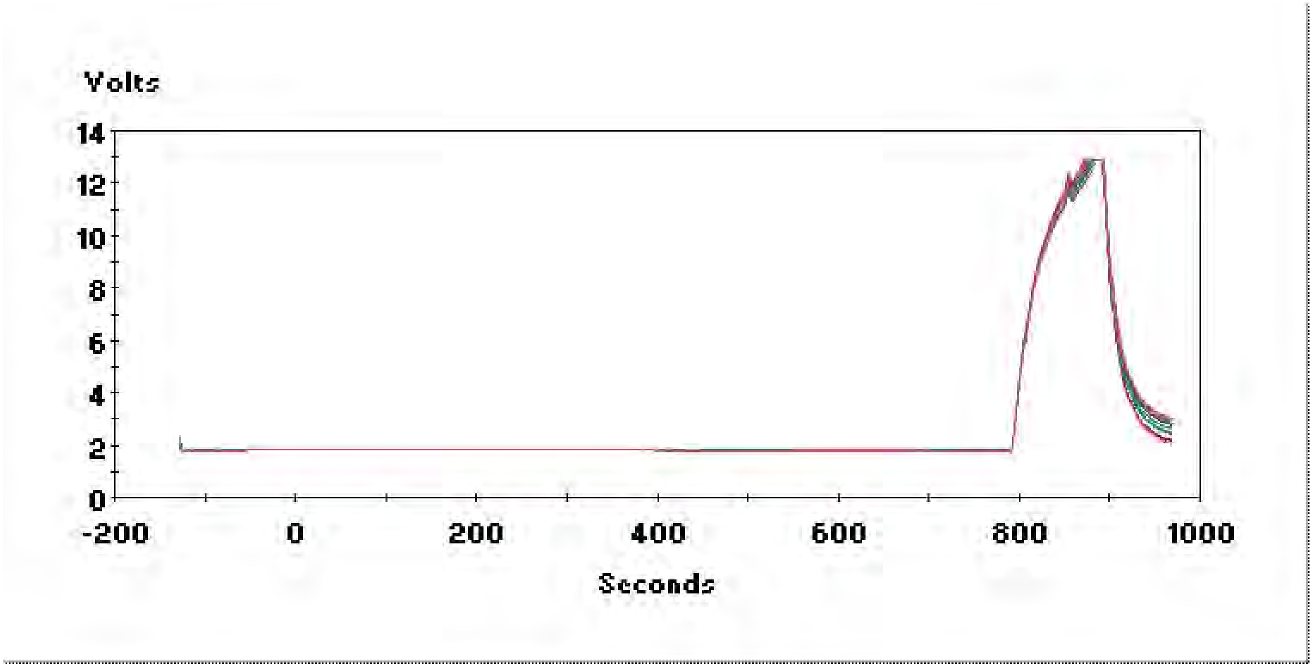

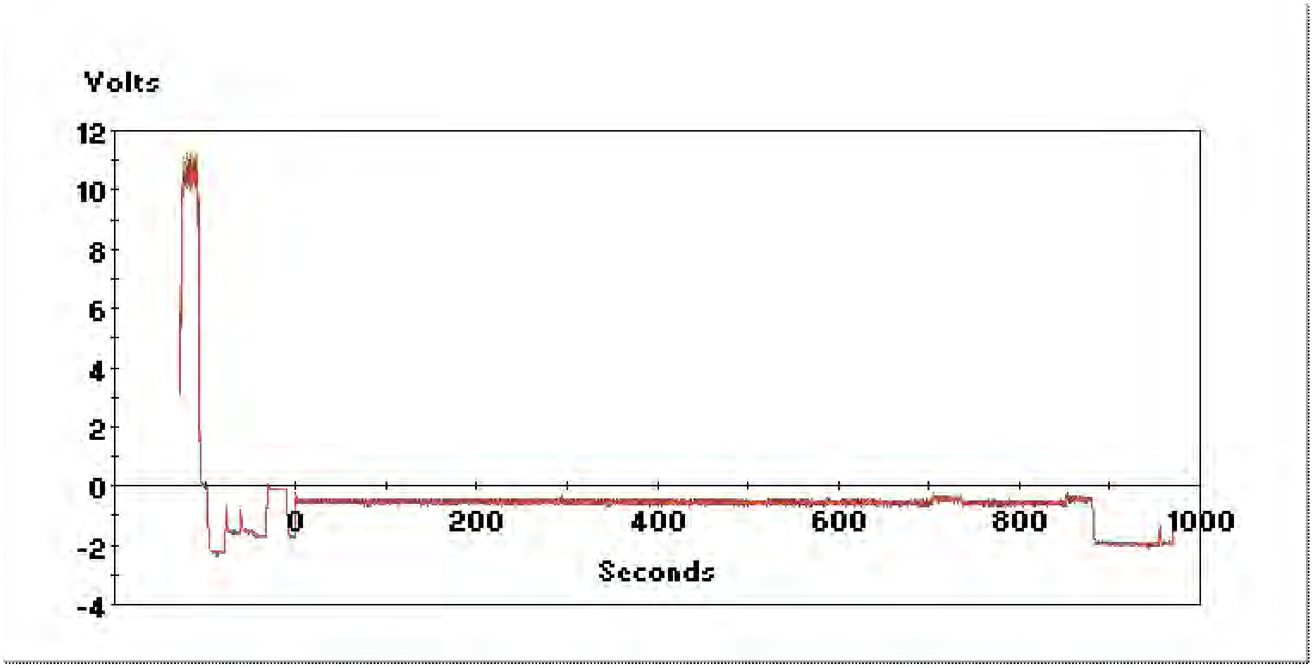

Experiment(x)

**Experiment Name:**	KD TeAb-B(2B18.2 epitope) vs B1 012925	**Start Time:**	Wed Jan 29 15:08:18 2025
**Experiment Type:**	Equilibrium	**End Time:**	Wed Jan 29 21:20:51 2025
**Constant Binding Partner (CBP)**		**Buffer:**	PBS/BSA
**Molecular Concentration:**	500.00fM	**Label:**	anti-BoBT/B-647
**Valency:**	1	**Label Conc:**	0
**Binding Site Concentration:**	500.00fM		

Comments(x)

beads: 2B18.2 IgG coated 01/24/25

sample volume: 3 ml

detection: anti-BoNT/B(1B22)-647 (1:500)

CBP: 2 pM BoNT B1

titrant: TeAb-B (Batch 05/5/15 (259 kDa, 0.81 mg/ml, 3.125 uM)

titration: 1-15 samples: 400pM - 24.41 fM (1:2); + NSB & B1 Toxin only

samples:

1-15) CBP + 400pM - 24.41 fM (1:2) TeAb-B

16) 100% CBP (2pM B1 toxin only)

beads: 2B18.2 IgG coated 01/24/25

sample volume: 3 ml

detection: anti-BoNT/B(1B22)-647 (1:500)

CBP: 0.5 pM BoNT B1

titrant: TeAb-B (Batch 05/5/15 (259 kDa, 0.81 mg/ml, 3.125 uM)

titration: 1-15 samples: 100pM - 6.1 fM (1:2); + NSB & B1 Toxin only

samples:

1-15) CBP + 100pM - 6.1 fM (1:2) TeAb-B

16) 100% CBP (0.5 pM B1 toxin only)

Timing(x)

Bead Handling (Custom Beads)

Draw Source	Time (sec)	Volume (uL)	Rate (mL/min)	Stir
Backflush	20	0	0.0000	
Buffer	20	500	1.5000	✓
Particle Reservoir 1	24	400	1.0000	✓
Buffer	30	500	1.0000	
Waste	2	8	0.2500	
Buffer	20	0	0.0000	
Buffer	9	150	1.0000	

Sample Timing

Draw Source	Time (sec)	Volume (uL)	Rate (mL/min)	Time Stamp
Sample Set 1,201-216	720	3000	0.2500	
Buffer	30	125	0.2500	
Rack 1: Tube 21	120	500	0.2500	
Buffer	30	125	0.2500	
Buffer	90	1500	1.0000	

Analysis(x)

Baseline / Endpoints:

to (sec) from beginning

to (sec) from end

Binding

Ignore	Signal (V)	Concentration
✓	0.1386	NSB
	0.1835	100.00pM
	0.2590	50.00pM
	0.2893	25.00pM
	0.3094	12.50pM
	0.3227	6.25pM
	0.3401	3.12pM
	0.3531	1.56pM
	0.3544	781.25fM
	0.3578	390.62fM
	0.3643	195.31fM
	0.3594	97.66fM
	0.3605	48.83fM
	0.3611	24.41fM
	0.3605	12.21fM
	0.3593	6.10fM
	0.3501	0

Kd: 92.84pM
Active CBP: 86.71fM
CBP %Activity: 17.34
Ratio: 0.0009
Sig 100%: 0.36
NSB: 0.03
%Error: 2.07

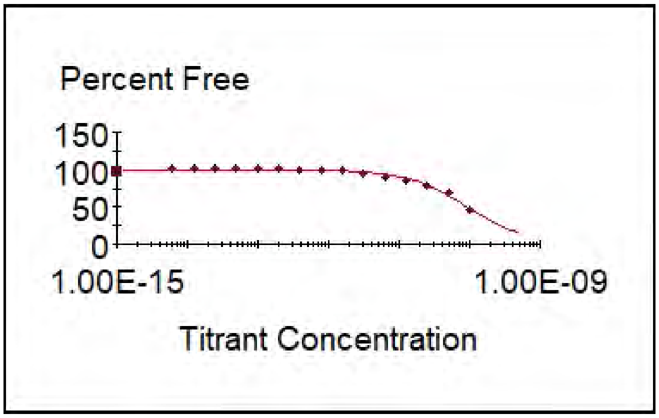

Kd: 92.84pM
95% confidence interval
Kd High: 214.16pM
Kd Low: 20.05pM

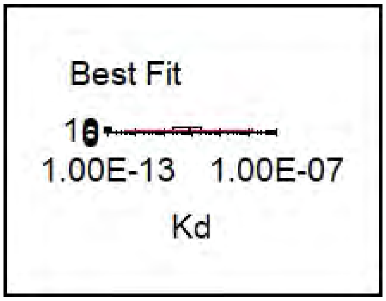

Active CBP: 86.71fM
CBP %Activity: 17.34
95% confidence interval
CBP High: Greater than 24.00pM
%Activity: Greater than 4800.32
CBP Low: Less than 313.26aM
%Activity: Less than 0.06

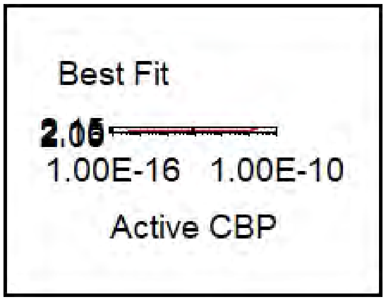

Data Traces(x)

Cycles: 1
Incubation delay (min): 30
Mix Time:

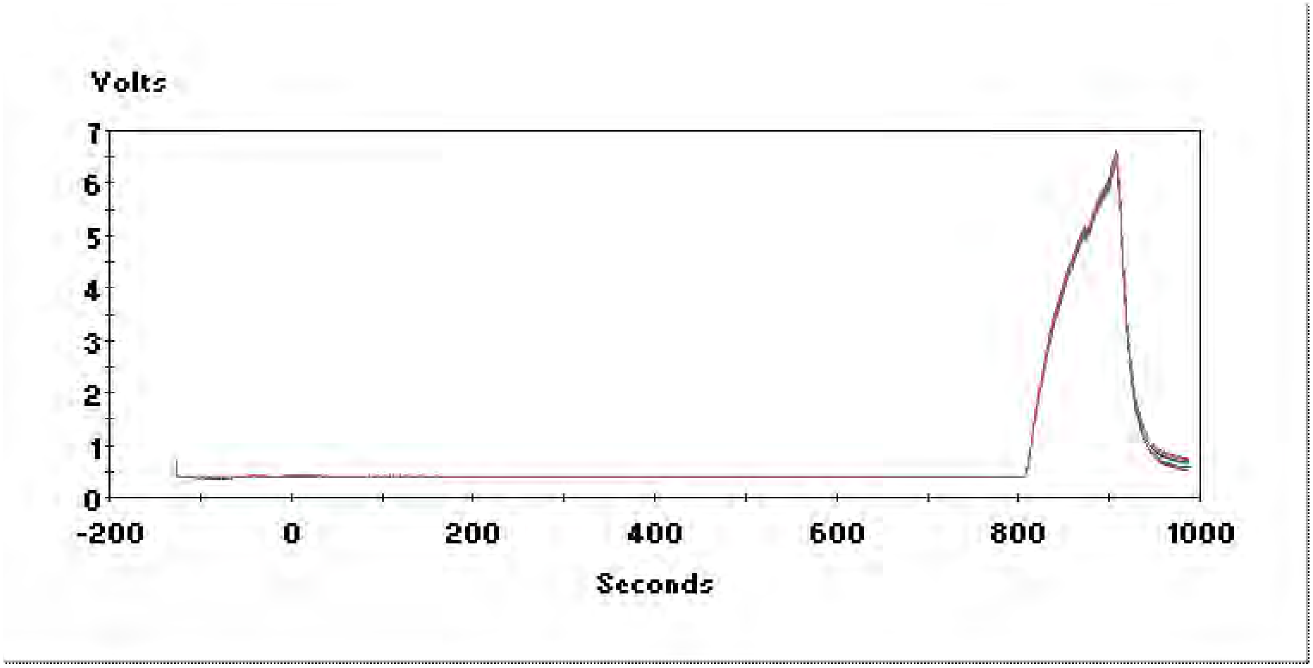

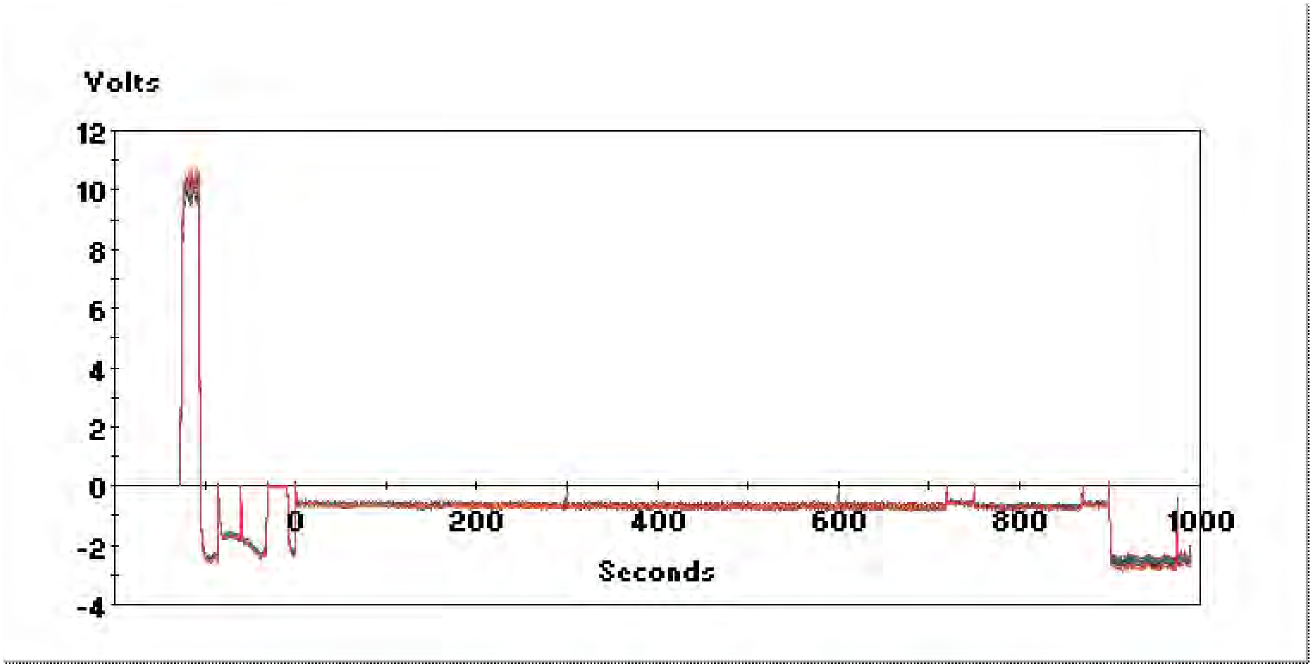

Experiment(x)

**Experiment Name:**	KD TsAb-B(2B18.2) vs LCHN-B18 2015	**Start Time:**	Mon Aug 3 12:56:49 2015
**Experiment Type:**	Equilibrium	**End Time:**	Tue Aug 4 01:33:12 2015
**Constant Binding Partner (CBP)**		**Buffer:**	PBS/BSA
**Molecular Concentration:**	7.00pM	**Label:**	aSV5-647
**Valency:**	1	**Label Conc:**	0
**Binding Site Concentration:**	7.00pM		

Comments(x)

beads: XB18 8/3/15

sample volume: 3 ml

detection: aSV5-647

CBP: 7 pM BoNT LCHN-B18 23421972-1 4/27/15

titrant: TsAb-B 5/5/15 (260 kDa, 1 mg/ml, 3.846 uM)

titration: 15 samples: 1 nM - 61 fM (1:2); + CBP only

samples:

1) NSB

2) 100% (CBP only)

3-15) titration
titration: 15 samples: 4 nM - 244 fM (1:2); + CBP only

Timing(x)

Bead Handling (Custom Beads)

Draw Source	Time (sec)	Volume (uL)	Rate (mL/min)	Stir
Backflush	20	0	0.0000	
Buffer	20	500	1.5000	✓
Particle Reservoir 1	16	267	1.0000	✓
Buffer	30	500	1.0000	
Waste	2	8	0.2500	
Buffer	20	0	0.0000	
Buffer	9	150	1.0000	

Sample Timing

Draw Source	Time (sec)	Volume (uL)	Rate (mL/min)	Time Stamp
Sample Set 1,201-216	720	3000	0.2500	
Buffer	30	125	0.2500	
Standards: Tube 3	120	500	0.2500	
Buffer	30	125	0.2500	
Buffer	90	1500	1.0000	

Analysis(x)

Baseline / Endpoints:

to (sec) from beginning

to (sec) from end

Ignore	Binding Signal (V)	Concentration
✓	0.3066	0
	0.9846	0
	0.3095	4.00nM
	0.3176	2.00nM
	0.3213	1.00nM
	0.3238	500.00pM
	0.3381	250.00pM
	0.4016	125.00pM
	0.4756	62.50pM
	0.5972	31.25pM
	0.7092	15.63pM
	0.8162	7.81pM
	0.8957	3.91pM
	0.9311	1.95pM
	0.9576	976.56fM
	0.9726	488.28fM
	0.9732	244.14fM
✓	0.3011	0
	0.9791	0
	0.2854	4.00nM
	0.3334	2.00nM
	0.3236	1.00nM
	0.3369	500.00pM
	0.3596	250.00pM
	0.3886	125.00pM
	0.4820	62.50pM
	0.5963	31.25pM
	0.6996	15.63pM
	0.8048	7.81pM
	0.8626	3.91pM
	0.8985	1.95pM
	0.9383	976.56fM
	0.9550	488.28fM
	0.9556	244.14fM

**Kd:**	20.53pM
**Active CBP:**	4.92pM
**CBP %Activity:**	70.27
**Ratio:**	0.2397
**Sig 100%:**	0.98
**Drift (%/run):**	0.1886
**NSB:**	0.30
**Drift (mV/run):**	0.2078
**%Error:**	1.31

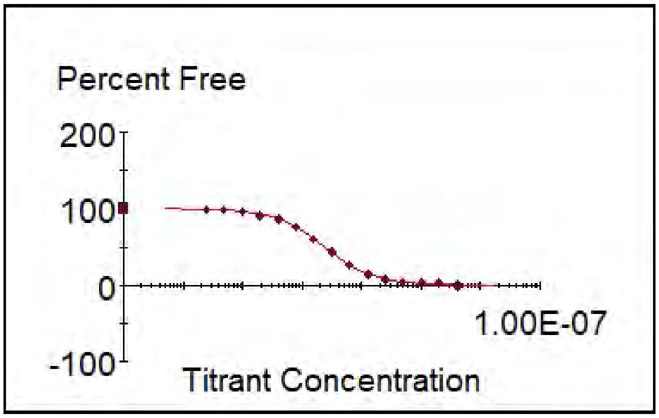

**Kd:**	20.53pM
**95% confidence interval**	
**Kd High:**	24.56pM
**Kd Low:**	16.52pM

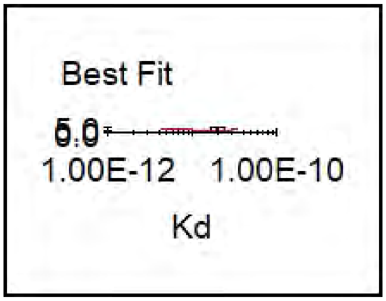

**Active CBP:**	4.92pM
**CBP %Activity:**	70.27
**95% confidence interval**	
**CBP High:**	15.22pM
**%Activity:**	217.48
**CBP Low:**	Less than 17.77fM
**%Activity:**	Less than 0.25

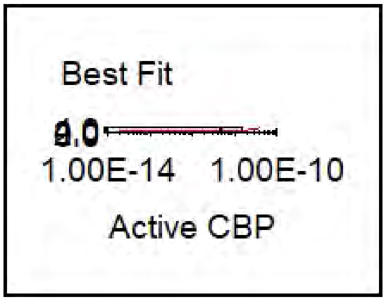

Data Traces(x)

Cycles: 2
Incubation delay (min): 0
Mix Time:

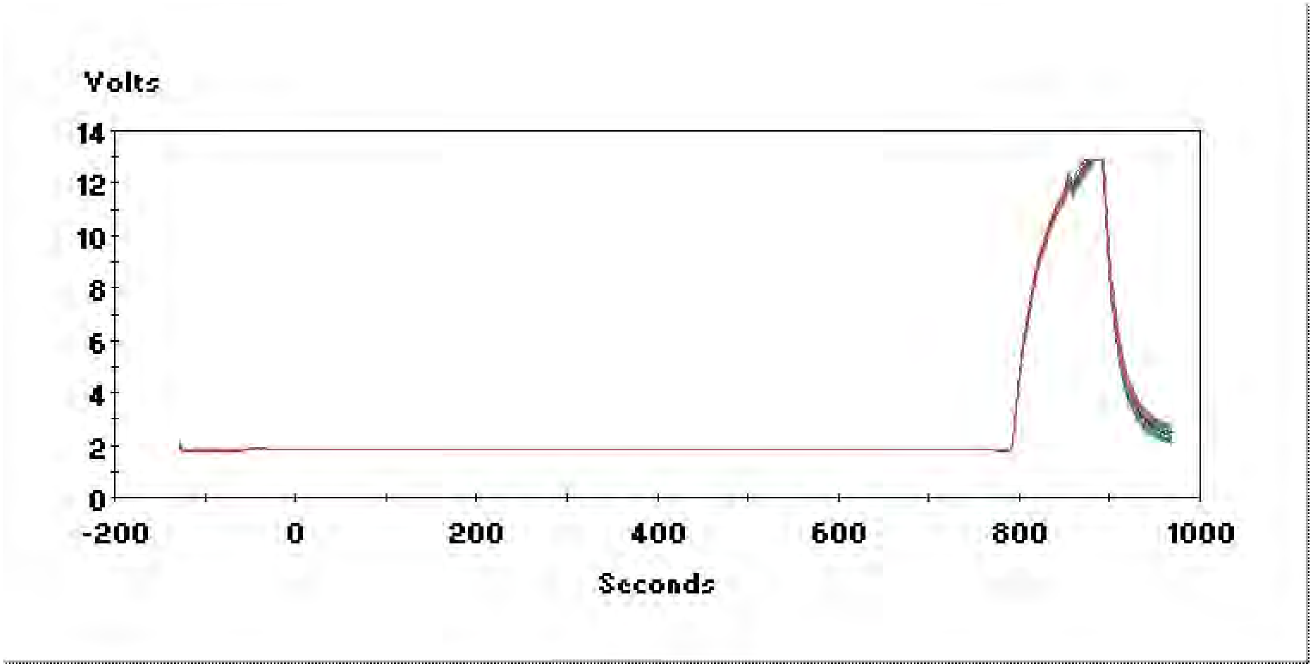

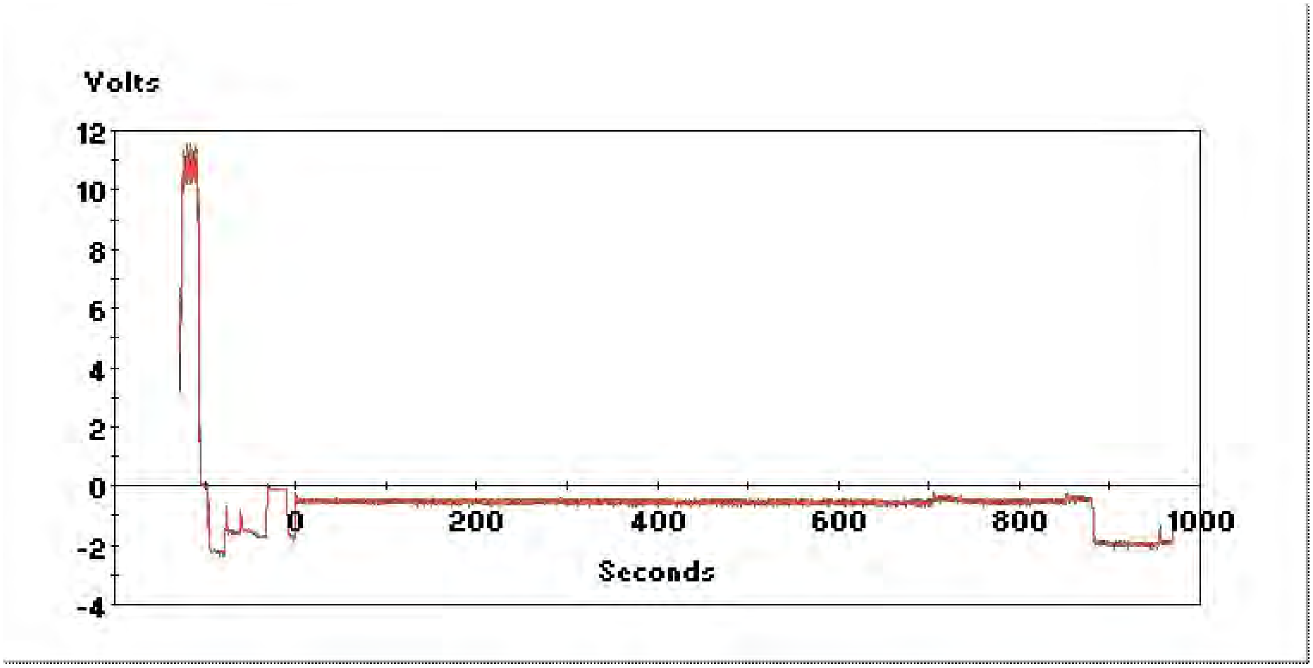

Experiment(x)

**Experiment Name:**	KD TsAb-B(2B23.1) vs LCHN-B23	**Start Time:**	Thu Aug 6 16:08:06 2015
**Experiment Type:**	Equilibrium	**End Time:**	Fri Aug 7 04:44:33 2015
**Constant Binding Partner (CBP)**		**Buffer:**	PBS/BSA
**Molecular Concentration:**	7.00pM	**Label:**	aSV5-647
**Valency:**	1	**Label Conc:**	0
**Binding Site Concentration:**	7.00pM		

Comments(x)

beads: XB23 8/6/15

sample volume: 3 ml

detection: aSV5-647

CBP: 7 pM BoNT LCHN-B23 23421972-6 4/27/15

titrant: TsAb-B 5/5/15 (260 kDa, 1 mg/ml, 3.846 uM)

titration: 15 samples: 2 nM - 122 fM (1:2); + CBP only

samples:

1) NSB

2) 100% (CBP only)

3-15) titration

Timing(x)

Bead Handling (Custom Beads)

Draw Source	Time (sec)	Volume (uL)	Rate (mL/min)	Stir
Backflush	20	0	0.0000	
Buffer	20	500	1.5000	✓
Particle Reservoir 1	16	267	1.0000	✓
Buffer	30	500	1.0000	
Waste	2	8	0.2500	
Buffer	20	0	0.0000	
Buffer	9	150	1.0000	

Sample Timing

Draw Source	Time (sec)	Volume (uL)	Rate (mL/min)	Time Stamp
Sample Set 1,201-216	720	3000	0.2500	
Buffer	30	125	0.2500	
Standards: Tube 3	120	500	0.2500	
Buffer	30	125	0.2500	
Buffer	90	1500	1.0000	

Analysis(x)

Baseline / Endpoints:
to (sec) from beginning
to (sec) from end

Ignore	Binding Signal (V)	Concentration
✓	0.3530	0
	1.2330	0
	0.3676	2.00nM
	0.3677	1.00nM
	0.4141	500.00pM
	0.4195	250.00pM
	0.5067	125.00pM
	0.6027	62.50pM
	0.7657	31.25pM
✓	1.0458	15.63pM
	1.0654	7.81pM
	1.1741	3.91pM
	1.2298	1.95pM
	1.2377	976.56fM
	1.2389	488.28fM
	1.2643	244.14fM
	1.2688	122.07fM
✓	0.3582	0
	1.2530	0
	0.3743	2.00nM
	0.3827	1.00nM
	0.4070	500.00pM
	0.4444	250.00pM
	0.4958	125.00pM
	0.5882	62.50pM
	0.7340	31.25pM
	0.9163	15.63pM
	1.0569	7.81pM
	1.1585	3.91pM
	1.2015	1.95pM
	1.2127	976.56fM
	1.2235	488.28fM
	1.2391	244.14fM
	1.2395	122.07fM

Kd: 18.46pM
Active CBP: 12.83pM
CBP %Activity: 183.35
Ratio: 0.6953
Sig 100%: 1.26
Drift (%/run): 0.0724
NSB: 0.37
Drift (mV/run): 0.0019
%Error: 1.30

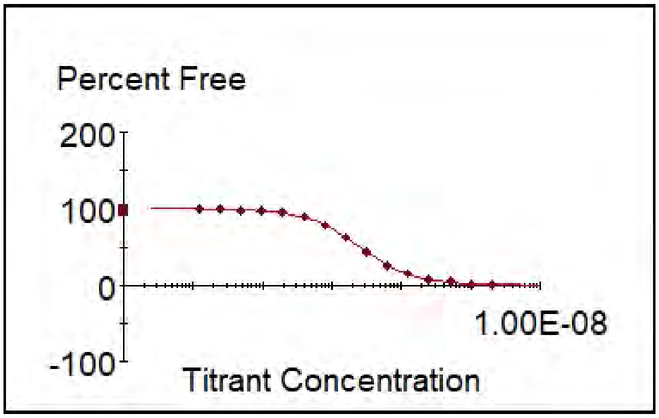

Kd: 18.46pM
95% confidence interval
Kd High: 23.22pM
Kd Low: 14.40pM

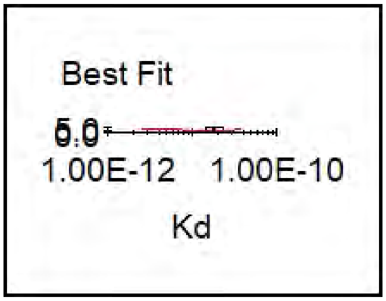

Active CBP: 12.83pM
CBP %Activity: 183.35
95% confidence interval
CBP High: 23.22pM
%Activity: 331.73
CBP Low: 1.97pM
%Activity: 28.12

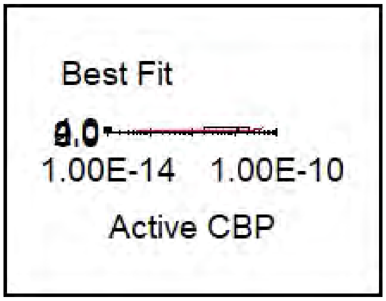

Data Traces (x)

Cycles: 2
Incubation delay (min): 0
Mix Time:

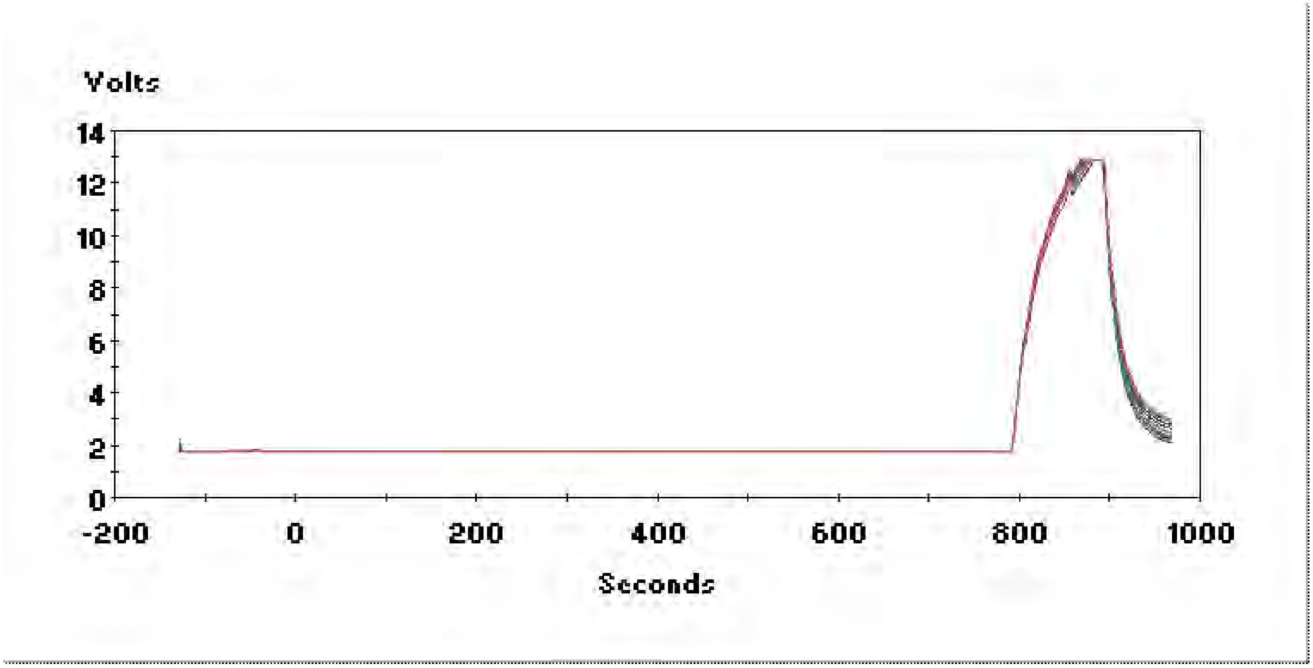

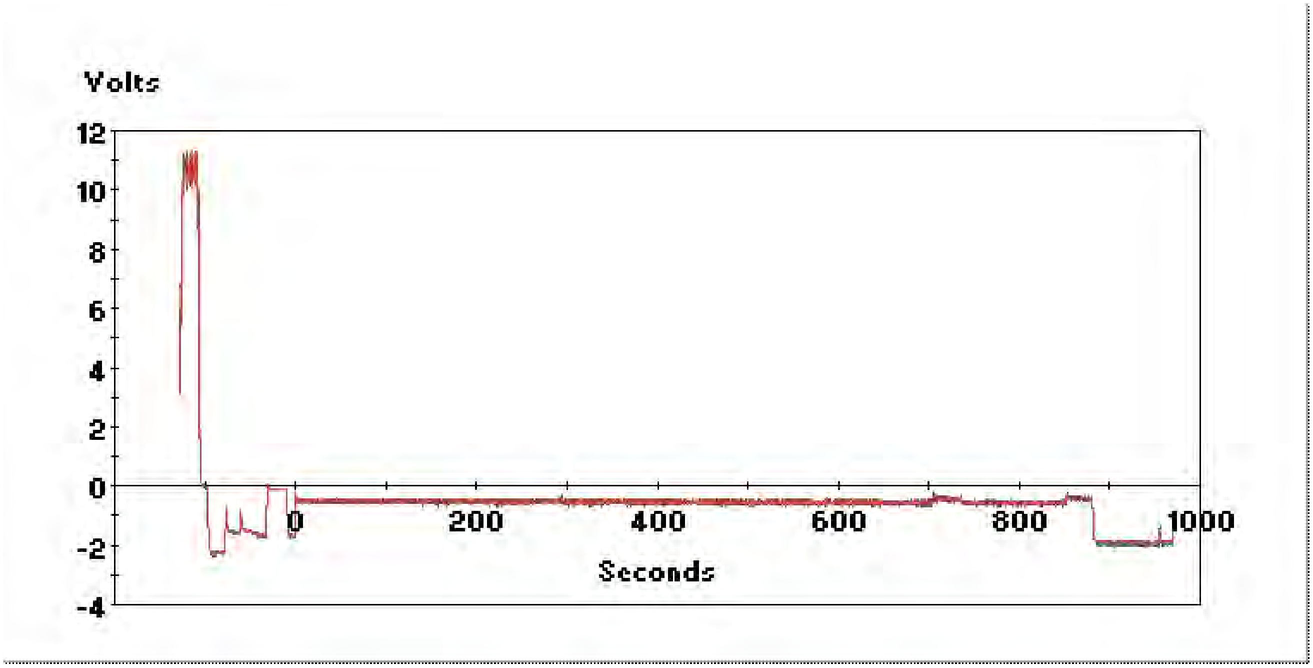

Experiment(x)

**Experiment Name:**	KD TsAb-B(2B23.1) vs B1{2}	**Start Time:**	Tue Apr 14 10:32:57 2015
**Experiment Type:**	Equilibrium	**End Time:**	Wed Apr 15 09:07:34 2015
**Constant Binding Partner (CBP)**		**Buffer:**	PBS/BSA
**Molecular Concentration:**	5.00pM	**Label:**	B6.1-647
**Valency:**	1	**Label Conc:**	0
**Binding Site Concentration:**	5.00pM		

Comments(x)

re-run after flow cell failure

beads: XB23 4/10/15

sample volume: 10 ml

detection: B6.1-647

CBP: 3 pM BoNT B1 100065 3/20/15

titrant: TsAb-B 3/18/15 (260 kDa, 0.3 mg/ml, 1.154 uM)

titration: 13 samples: 200 pM - 48 fM (1:2); + B1 only

samples:

1) NSB

2) 100% (B1 only)

3-15) titration of TsAb-B

Timing(x)

Bead Handling (Custom Beads)

Draw Source	Time (sec)	Volume (uL)	Rate (mL/min)	Stir
Backflush	20	0	0.0000	
Buffer	20	500	1.5000	✓
Particle Reservoir 2	20	333	1.0000	✓
Buffer	30	500	1.0000	
Waste	2	8	0.2500	
Buffer	20	0	0.0000	
Buffer	9	150	1.0000	

Sample Timing

Draw Source	Time (sec)	Volume (uL)	Rate (mL/min)	Time Stamp
Sample Set 1,201-214	2400	10000	0.2500	
Buffer	30	125	0.2500	
Standards: Tube 3	120	500	0.2500	
Buffer	30	125	0.2500	
Buffer	90	1500	1.0000	

Analysis(x)

Baseline / Endpoints:
to (sec) from beginning
to (sec) from end

Ignore	Binding Signal (V)	Concentration
✓	0.1221	0
	0.4419	0
	0.1627	200.00pM
	0.1412	100.00pM
	0.1396	50.00pM
	0.1216	25.00pM
	0.1279	12.50pM
	0.1494	6.25pM
	0.2347	3.13pM
	0.3168	1.56pM
	0.3780	781.25fM
	0.4053	390.63fM
	0.4020	195.31fM
	0.3832	97.66fM
	0.4165	48.83fM
✓	0.1128	0
	0.4341	0
	0.1561	200.00pM
	0.1398	100.00pM
	0.1294	50.00pM
	0.1231	25.00pM
	0.1233	12.50pM
	0.1476	6.25pM
✓	0.0850	3.13pM
	0.3126	1.56pM
✓	-0.0054	781.25fM
✓	0.0000	0

**Kd:**	253.53fM
**Active CBP:**	4.33pM
**CBP %Activity:**	86.66
**Ratio:**	17.0907
**Sig 100%:**	0.42
**Drift (%/run):**	0.3678
**NSB:**	0.12
**Drift (mV/run):**	-0.2073
**TR NSB:**	2.14e+08
**%Error:**	2.56

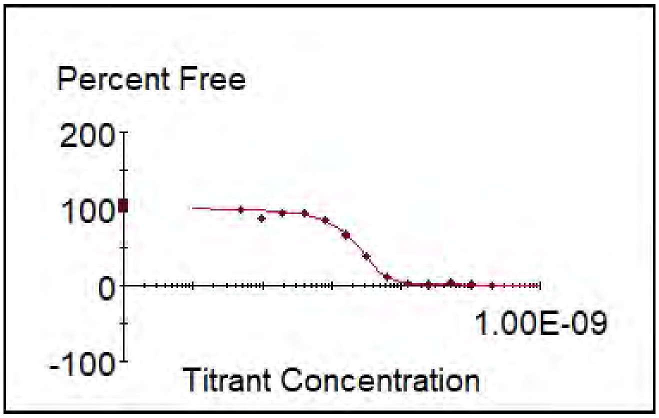

**Kd:**	253.53fM
**95% confidence interval**	
**Kd High:**	627.67fM
**Kd Low:**	49.64fM

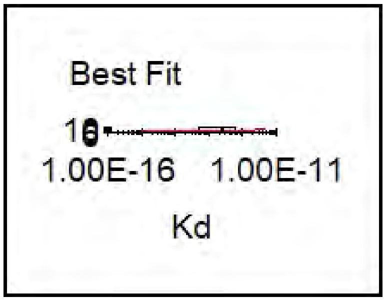

**Active CBP:**	4.33pM
**CBP %Activity:**	86.66
**95% confidence interval**	
**CBP High:**	5.37pM
**%Activity:**	107.44
**CBP Low:**	3.14pM
**%Activity:**	62.76

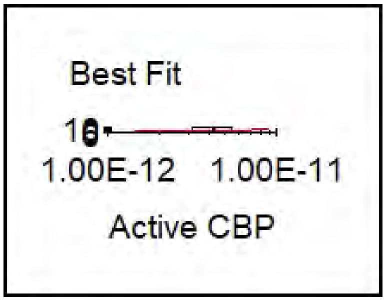

Data Traces(x)

Cycles: 2
Incubation delay (min): 0
Mix Time:

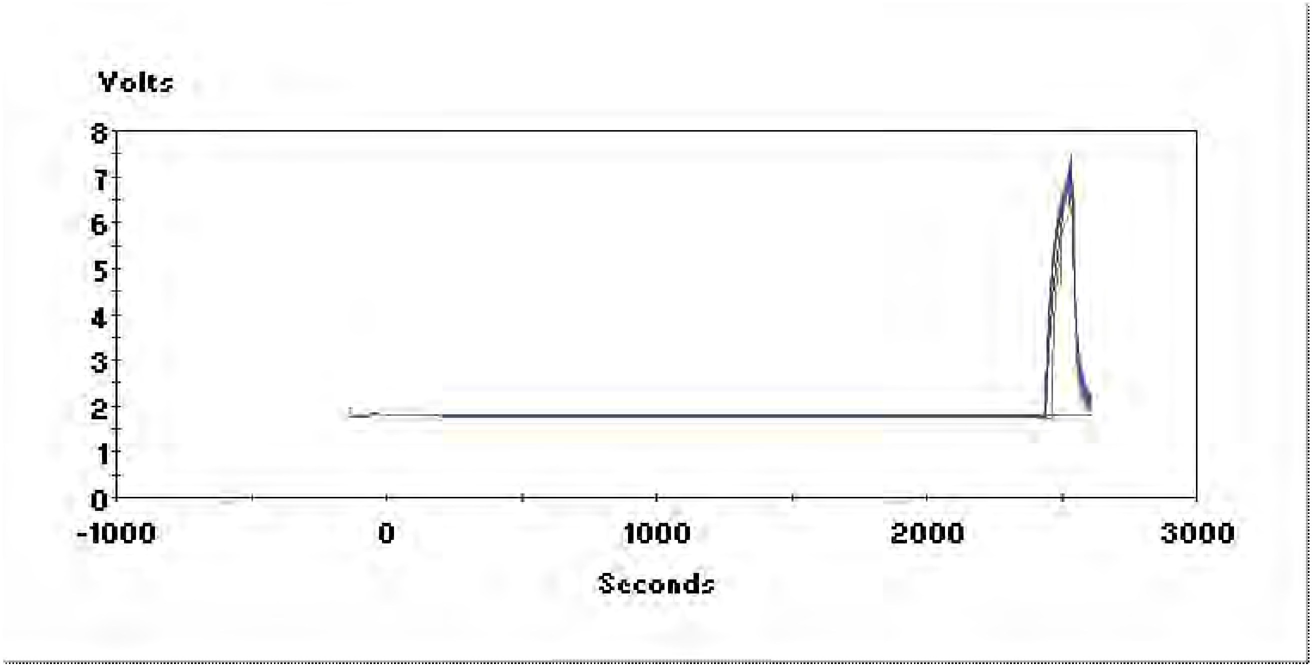

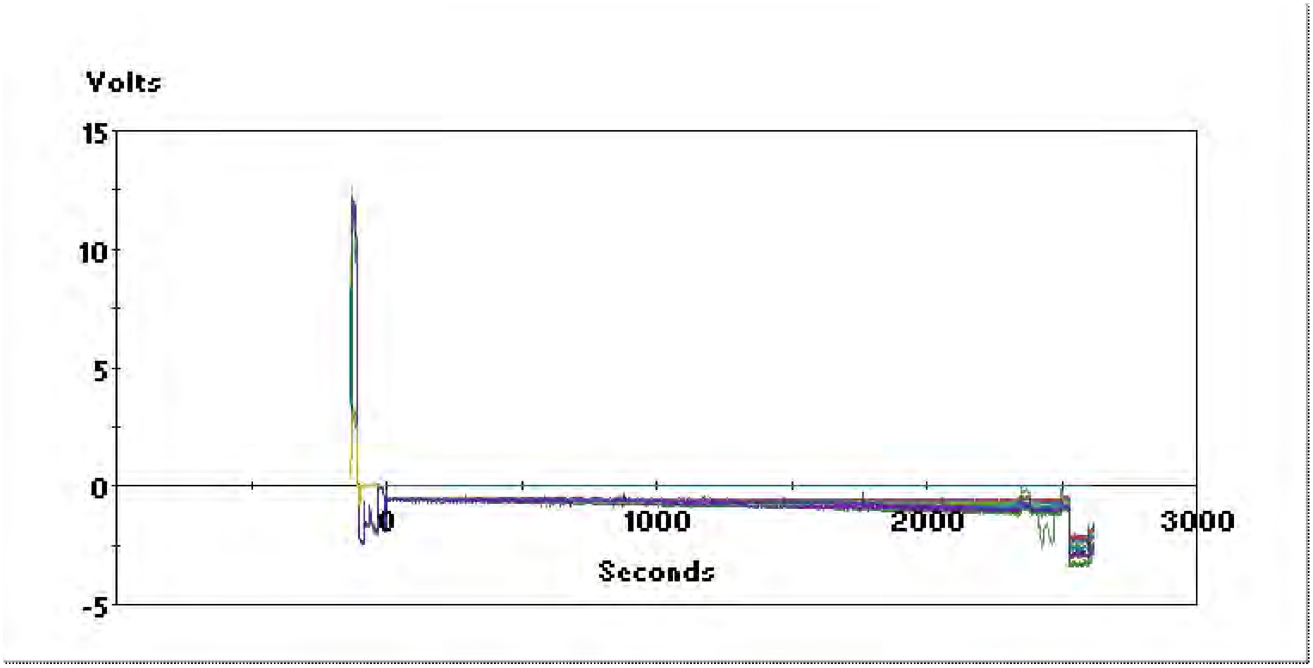

Experiment(x)

**Experiment Name:**	Kinetics Direct 1B10.1(beads) -BoNTB1{4}	**Start Time:**	Mon Sep 17 11:24:14 2007
**Experiment Type:**	Kinetics, Direct	**End Time:**	Mon Sep 17 13:26:33 2007
**Binding Site Concentration:**	298.00pM	**Buffer:**	PBS/BSA
**Kd:**	331.00fM	**Label:**	B12.1-647
**Titrant:**	700.00pM	**Label Conc:**	800.00ng/ml

Comments(x)

1B10.1beads 9/11/07 fc

1B10.1 IgG 7/25/07

BoNT B1 8/2/07

B12.1-647

meter: 1.2600

Timing(x)

Bead Handling (Soft Beads)

Draw Source	Time (sec)	Volume (uL)	Rate (mL/min)	Stir
Backflush	20	0	0.0000	
Buffer	20	500	1.5000	✓
Particle Reservoir	20	333	1.0000	✓
Buffer	40	333	0.5000	
Waste	5	25	0.3000	
Buffer	2	10	0.3000	
Buffer	20	0	0.0000	
Buffer	9	150	1.0000	

Sample Timing

Draw Source	Time (sec)	Volume (uL)	Rate (mL/min)	Time Stamp
Line 1	120	500	0.2500	
Buffer	30	125	0.2500	
Inject	120	500	0.2500	
Buffer	30	125	0.2500	
Buffer	90	1500	1.0000	

Analysis(x)

Baseline / Endpoints:
to (sec) from beginning
to (sec) from end

Binding

Ignore	Signal (V)	Time
	0.7360	378
	0.4674	942
	0.3346	1507
	0.2547	2072
	0.2115	2637
	0.1831	3201
	0.1617	3765
	0.1433	4330
	0.1429	4895
	0.1377	5459
	0.1598	6023
	0.1296	6588
	0.1313	7152

**kon:**	1.903e+06/Ms
**koff:**	6.299e-07/s
**Sig 100%:**	1.09
**NSB:**	0.13
**%Error:**	0.74
**Kd:**	331.00fM
**CBP:**	298.00pM
**Titrant:**	700.00pM

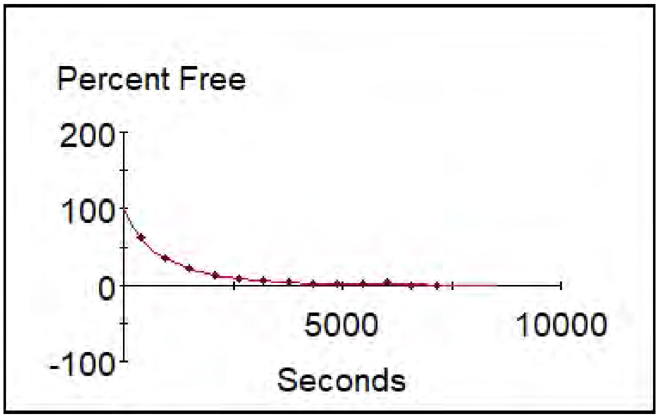

**kon:**	1.903e+06/Ms
**95% confidence interval**	
**kon High:**	2.026e+06/Ms
**kon Low:**	1.788e+06/Ms

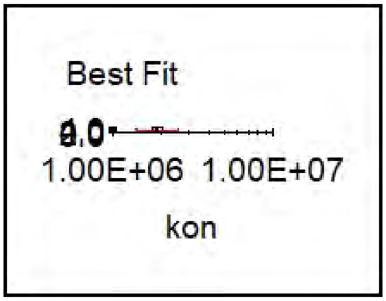

Data Traces(x)

**Cycles:**	13
**Incubation delay (min):**	0
**Mix Time:**	Mon Sep 17 11:21:31 2007

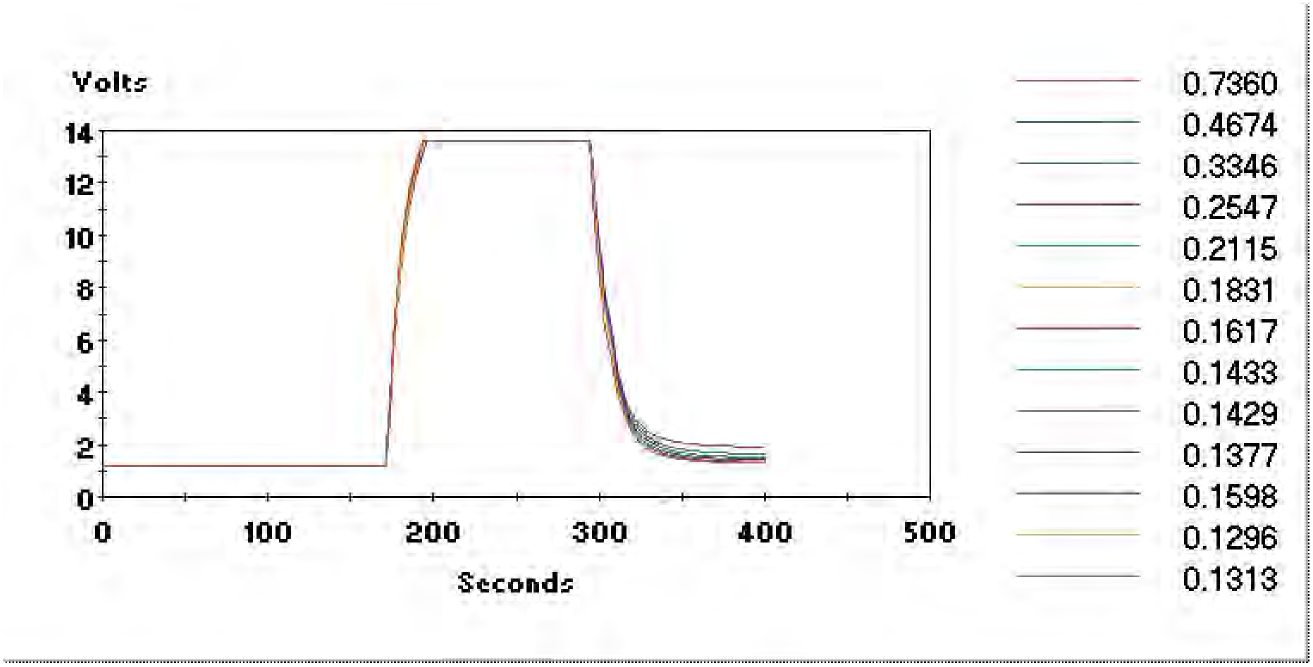

Experiment(x)

**Experiment Name:**	KinDir 1B10.1 IgG1 vs NXB10 domain Kon 081124{2}	**Start Time:**	Sun Aug 11 17:13:13 2024
**Experiment Type:**	Kinetics, Direct	**End Time:**	Sun Aug 11 19:33:12 2024
**Binding Site Concentration:**	100.00pM	**Buffer:**	PBS/BSA
**Kd:**	473.00fM	**Label:**	Anti-SV5-647
**Titrant:**	200.00pM	**Label Conc:**	0

Comments(x)

beads: 1B10.1 IgG1 coated 07/28/24

sample volume: 500 ul

detection: Anti-SV5 -647 (1:2000)

CBP: 100 pM [final] NXB10 Domain

titrant: 200 pM [final] 1B10.1 IgG 08/11/24

beads: 1B10.1 IgG1 coated 07/28/24

sample volume: 500 ul

detection: Anti-SV5 -647 (1:2000)

CBP: 100 pM [final] NXB10 Domain

titrant: 200 pM [final] 1B10.1 IgG 08/11/24

Timing(x)

Bead Handling (Custom Beads)

Draw Source	Time (sec)	Volume (uL)	Rate (mL/min)	Stir
Backflush	20	0	0.0000	
Buffer	20	500	1.5000	✓
Particle Reservoir 1	25	420	1.0000	✓
Buffer	30	500	1.0000	
Waste	2	8	0.2500	
Buffer	20	0	0.0000	
Buffer	9	150	1.0000	

Sample Timing

Draw Source	Time (sec)	Volume (uL)	Rate (mL/min)	Time Stamp
Rack 2: Tube 1	120	500	0.2500	
Buffer	30	125	0.2500	
Rack 2: Tube 60	120	500	0.2500	
Buffer	30	125	0.2500	
Buffer	90	1500	1.0000	

Analysis(x)

Baseline / Endpoints:
to (sec) from beginning
to (sec) from end

Binding

**Ignore**	**Signal (V)**	**Time**
	0.2429	334.5
	0.1820	1033
	0.1310	1731.5
	0.1178	2430.5
✓	0.0814	3129.5
	0.1137	3828.5
	0.0983	4527.5
	0.0934	5227.5
	0.0553	5926.5
✓	0.0782	6626.5
	0.0519	7326
	0.0410	8026.5

**kon:**	2.324e+06/Ms
**koff:**	1.099e-06/s
**Sig 100%:**	0.27
**NSB:**	0.03
**%Error:**	5.33
**Kd:**	473.00fM
**CBP:**	100.00pM
**Titrant:**	200.00pM

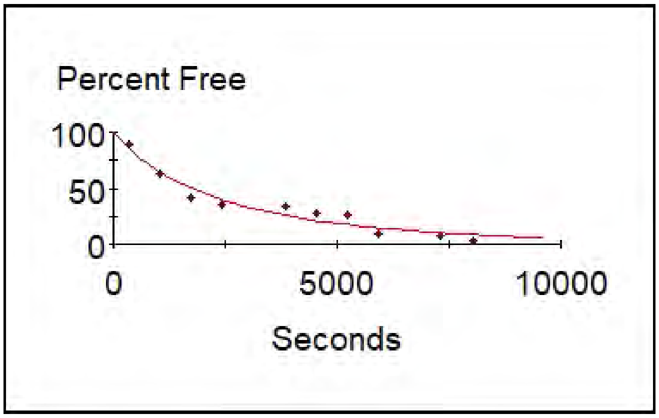

kon: 2.324e+06/Ms
95% confidence interval
kon High: 4.040e+06/Ms
kon Low: 1.078e+06/Ms

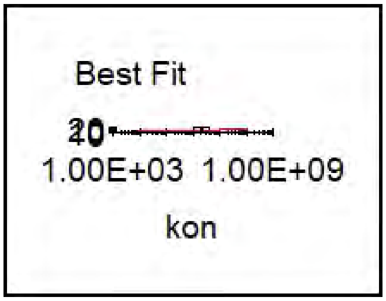

Data Traces(x)

**Cycles:**	12
**Incubation delay (min):**	0
**Mix Time:**	Sun Aug 11 17:12:37 2024

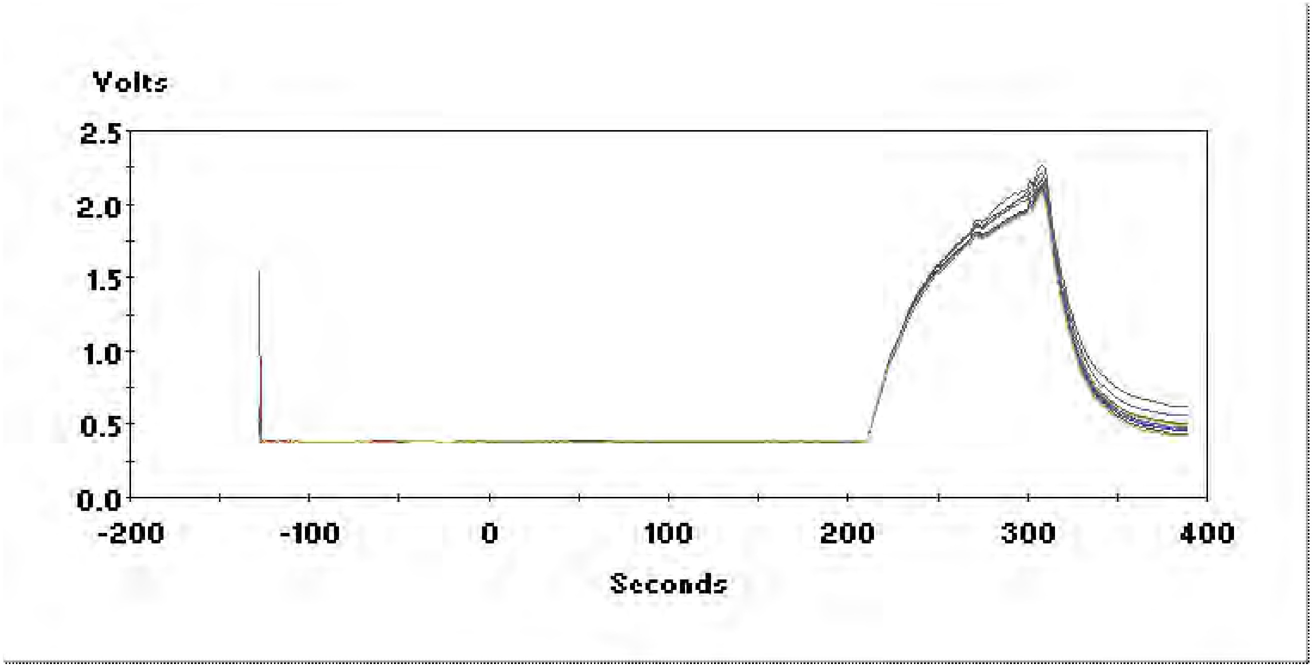

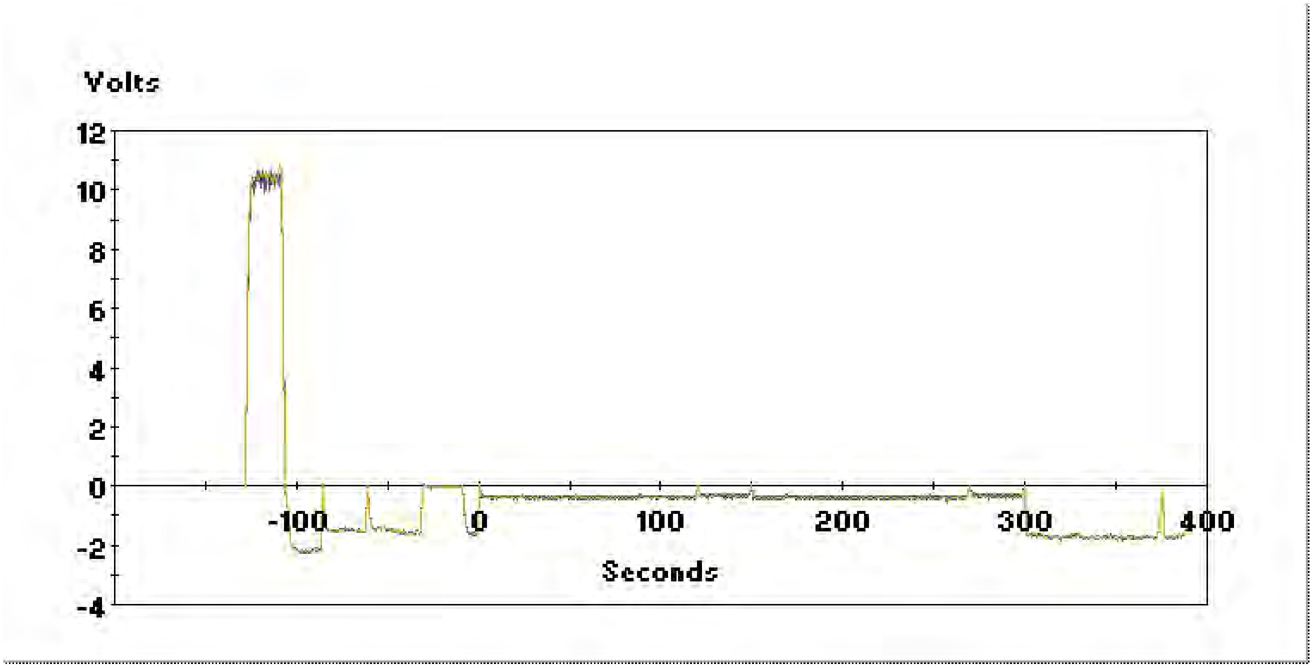

Experiment(x)

**Experiment Name:**	KinDir 2B18.1 IgG vs B1 toxin 030813	**Start Time:**	Fri Mar 8 12:19:02 2013
**Experiment Type:**	Kinetics, Direct	**End Time:**	Fri Mar 8 14:43:29 2013
**Binding Site Concentration:**	200.00pM	**Buffer:**	PBS
**Kd:**	56.88pM	**Label:**	B6.1-647
**Titrant:**	700.00pM	**Label Conc:**	0

Comments(x)

XB18 beads 2/25/13

BoNT B1 3/7/13

2B18.1 IgG 7/7/10

B6.1-647

meter: 1.2648

Timing(x)

Bead Handling (Soft Beads)

Draw Source	Time (sec)	Volume (uL)	Rate (mL/min)	Stir
Backflush	20	0	0.0000	
Buffer	20	500	1.5000	✓
Particle Reservoir	26	433	1.0000	✓
Buffer	40	333	0.5000	
Waste	5	25	0.3000	
Buffer	2	10	0.3000	
Buffer	20	0	0.0000	
Buffer	20	83	0.2500	
Waste	5	25	0.3000	
Buffer	2	10	0.3000	
Buffer	20	0	0.0000	
Buffer	36	150	0.2500	

Sample Timing

Draw Source	Time (sec)	Volume (uL)	Rate (mL/min)	Time Stamp
Line 1	120	500	0.2500	
Buffer	30	125	0.2500	
Inject	120	500	0.2500	
Buffer	30	125	0.2500	
Buffer	90	1500	1.0000	

Analysis(x)

Baseline / Endpoints:
to (sec) from beginning
to (sec) from end

	Binding	
**Ignore**	**Signal (V)**	**Time**
	0.7160	468.5
	0.5244	1135
	0.4411	1802
	0.3697	2468.5
	0.3504	3135.5
	0.3282	3802
	0.3071	4469
	0.3042	5136
	0.3106	5803
	0.2936	6469
	0.3073	7136
	0.2881	7803
	0.3026	8469

**kon:**	1.267e+06/Ms
**koff:**	7.209e-05/s
**Sig 100%:**	0.95
**NSB:**	0.23
**%Error:**	0.92
**Kd:**	56.88pM
**CBP:**	200.00pM
**Titrant:**	700.00pM

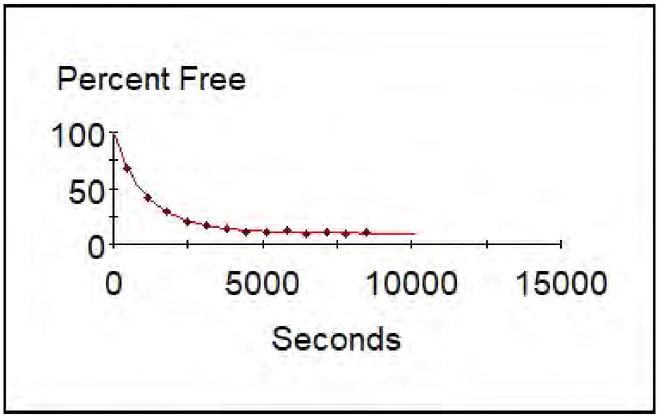

**kon:**	1.267e+06/Ms
**95% confidence interval**	
**kon High:**	1.368e+06/Ms
**kon Low:**	1.174e+06/Ms

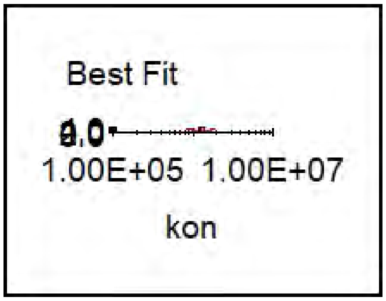

Data Traces(x)

**Cycles:**	13
**Incubation delay (min):**	0
**Mix Time:**	Fri Mar 8 12:16:25 2013

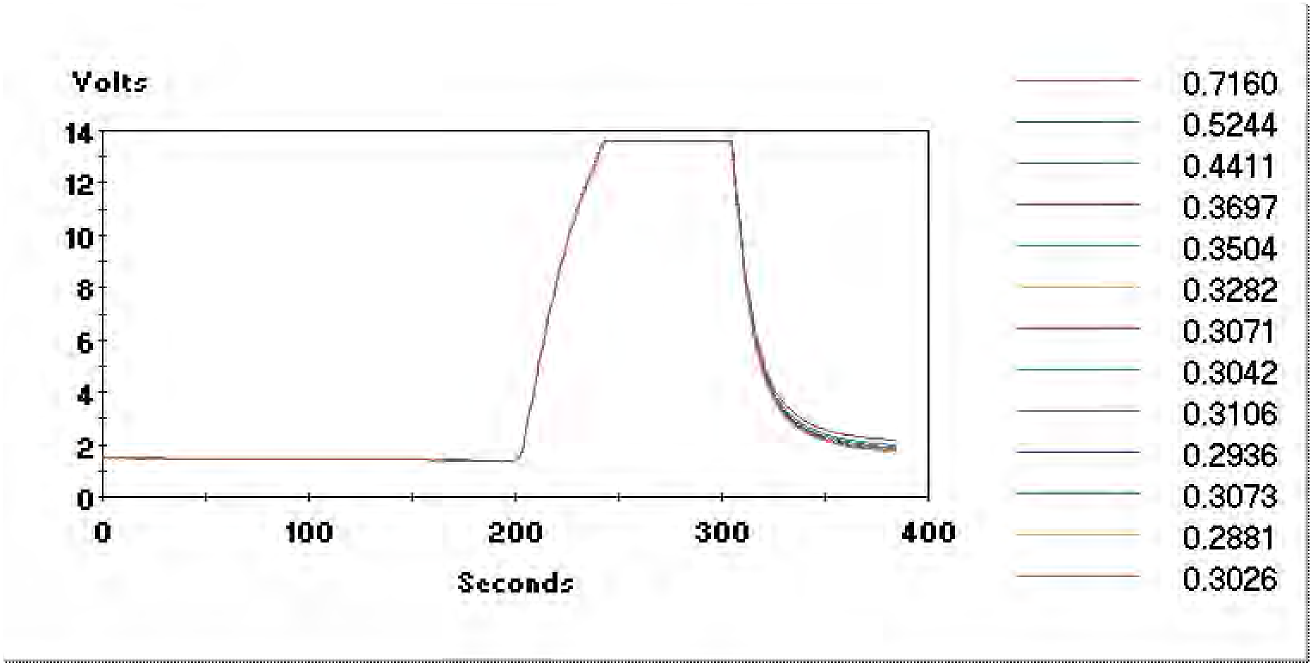

Experiment(x)

**Experiment Name:**	KinDir 2B18.1 IgG1 vs NXB18 domain Kon 081324	**Start Time:**	Tue Aug 13 16:25:32 2024
**Experiment Type:**	Kinetics, Direct	**End Time:**	Tue Aug 13 18:21:58 2024
**Binding Site Concentration:**	100.00pM	**Buffer:**	PBS/BSA
**Kd:**	5.12pM	**Label:**	Anti-SV5-647
**Titrant:**	200.00pM	**Label Conc:**	0

Comments(x)

beads: 2B18.1 IgG1 coated 08/12/24

sample volume: 500 ul

detection: Anti-SV5 -647 (1:1000)

CBP: 100 pM [final] NXB18 Domain

titrant: 200 pM [final] 2B18.1 IgG 08/13/24

beads: 2B18.1 IgG1 coated 08/12/24

sample volume: 500 ul

detection: Anti-SV5 -647 (1:1000)

CBP: 100 pM [final] NXB18 Domain

titrant: 200 pM [final] 2B18.2 IgG 08/12/24

Timing(x)

Bead Handling (Custom Beads)

Draw Source	Time (sec)	Volume (uL)	Rate (mL/min)	Stir
Backflush	20	0	0.0000	
Buffer	20	500	1.5000	✓
Particle Reservoir 1	24	400	1.0000	✓
Buffer	30	500	1.0000	
Waste	2	8	0.2500	
Buffer	20	0	0.0000	
Buffer	9	150	1.0000	

Sample Timing

Draw Source	Time (sec)	Volume (uL)	Rate (mL/min)	Time Stamp
Rack 2: Tube 1	120	500	0.2500	
Buffer	30	125	0.2500	
Rack 2: Tube 60	120	500	0.2500	
Buffer	30	125	0.2500	
Buffer	90	1500	1.0000	

Analysis(x)

Baseline / Endpoints:

to (sec) from beginning

to (sec) from end

Binding

Ignore	Signal (V)	Time
	0.2768	297.5
	0.2098	995
	0.1875	1692
	0.1341	2389.5
✓	0.1150	3086.5
	0.1236	3784.5
	0.1245	4482.5
	0.0941	5181
	0.0903	5879
	0.0974	6577.5

**kon:**	3.036e+06/Ms
**koff:**	1.554e-05/s
**Sig 100%:**	0.32
**NSB:**	0.07
**%Error:**	3.59
**Kd:**	5.12pM
**CBP:**	100.00pM
**Titrant:**	200.00pM

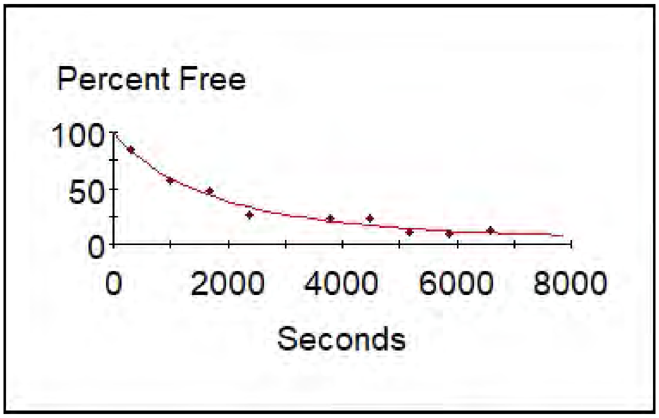

**kon:**	3.036e+06/Ms
**95% confidence interval**	
**kon High:**	4.149e+06/Ms
**kon Low:**	2.101e+06/Ms

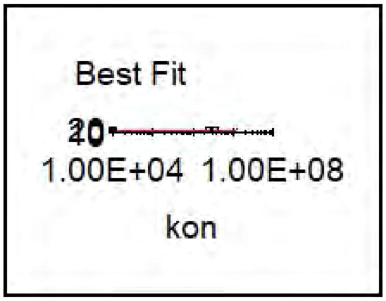

Data Traces(x)

Cycles: 10
Incubation delay (min): 0
Mix Time:

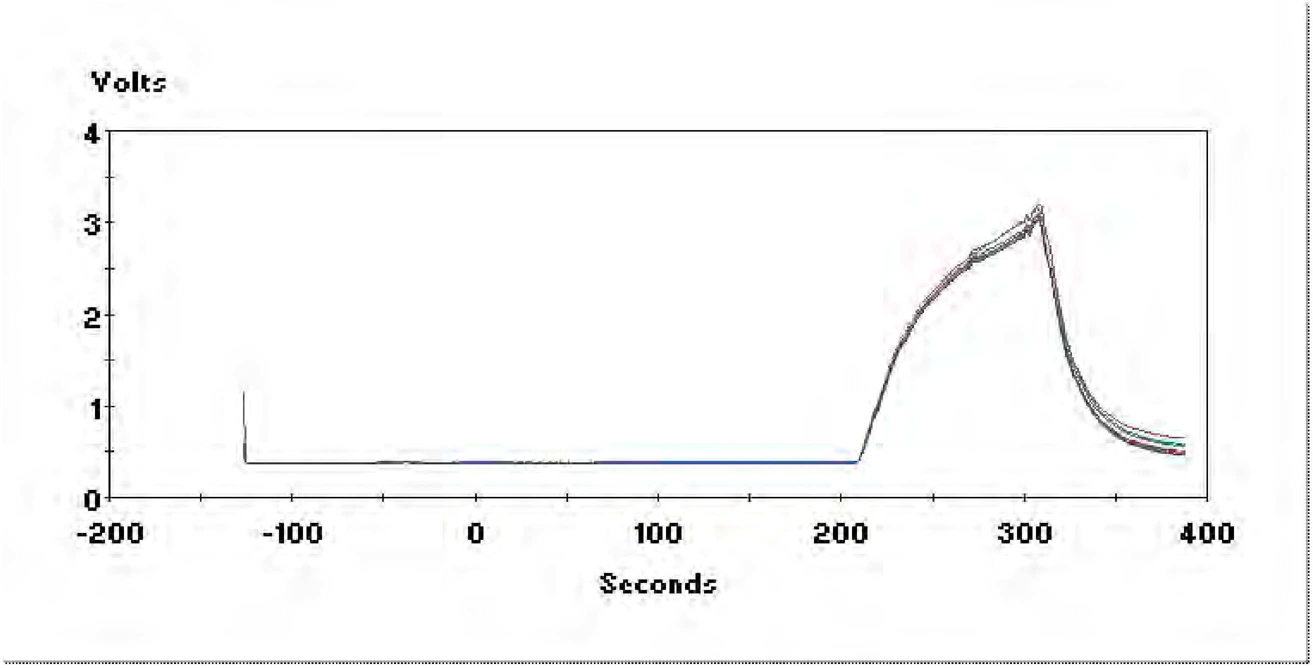

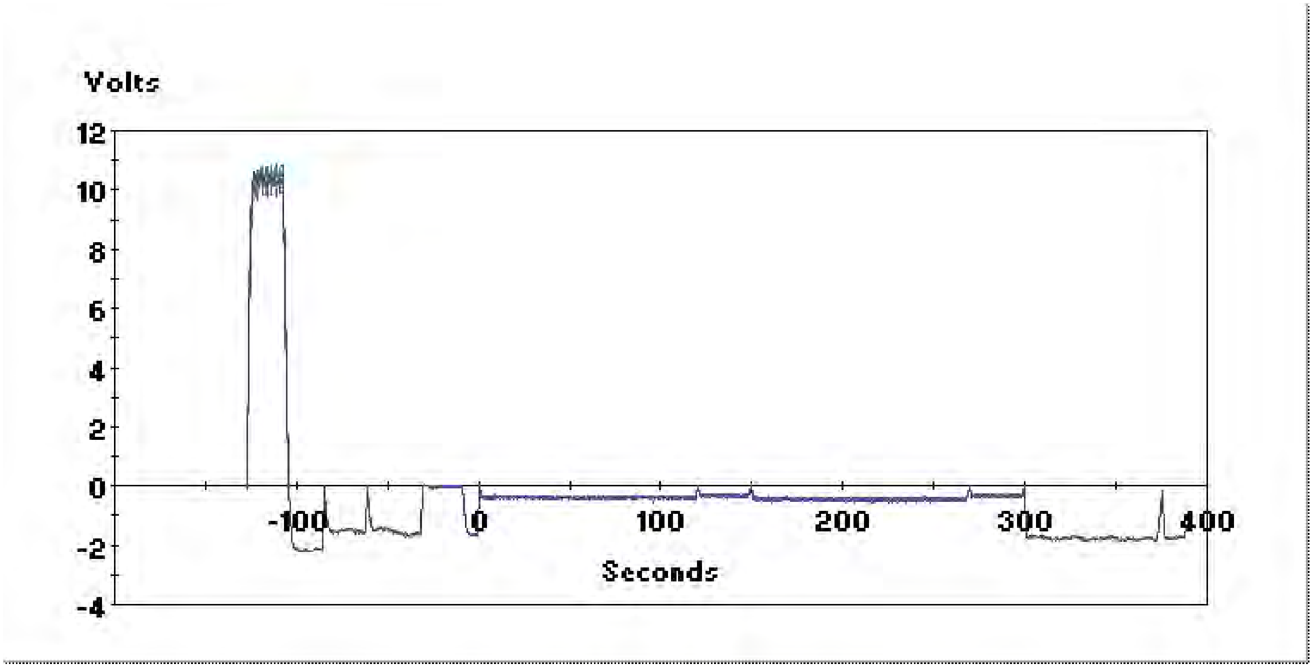

Experiment(x)

**Experiment Name:**	KinDir 2B23 vs B1 052311	**Start Time:**	Mon May 23 16:59:54 2011
**Experiment Type:**	Kinetics, Direct	**End Time:**	Mon May 23 19:04:01 2011
**Binding Site Concentration:**	70.00pM	**Buffer:**	PBS/BSA
**Kd:**	38.07pM	**Label:**	B6.1-647
**Titrant:**	800.00pM	**Label Conc:**	0

Comments(x)

2B23 beads 5/23/11

BoNT B1 100065 5/13/11

2B23 IgG 2/5/08

B6.1-647

meter: 1.5076

Timing(x)

Bead Handling (Soft Beads)

Draw Source	Time (sec)	Volume (uL)	Rate (mL/min)	Stir
Backflush	20	0	0.0000	
Buffer	20	500	1.5000	✓
Particle Reservoir	18	300	1.0000	✓
Buffer	40	333	0.5000	
Waste	5	25	0.3000	
Buffer	2	10	0.3000	
Buffer	20	0	0.0000	
Buffer	9	150	1.0000	

Sample Timing

Draw Source	Time (sec)	Volume (uL)	Rate (mL/min)	Time Stamp
Line 1	120	500	0.2500	
Buffer	30	125	0.2500	
Inject	120	500	0.2500	
Buffer	30	125	0.2500	
Buffer	90	1500	1.0000	

Analysis(x)

Baseline / Endpoints:

to (sec) from beginning

to (sec) from end

	**Binding**	
**Ignore**	**Signal (V)**	**Time**
	0.9994	388.5
	0.7933	961.5
	0.6631	1534
	0.5330	2106.5
	0.4738	2679.5
	0.4204	3252.5
✓	0.0129	3825
	0.3510	4398
	0.3234	4970.5
	0.3057	5543.5
	0.2896	6116.5
	0.2848	6689.5
	0.2819	7262.5

**kon:**	6.785e+05/Ms
**koff:**	2.583e-05/s
**Sig 100%:**	1.18
**NSB:**	0.21
**%Error:**	0.60
**Kd:**	38.07pM
**CBP:**	70.00pM
**Titrant:**	800.00pM

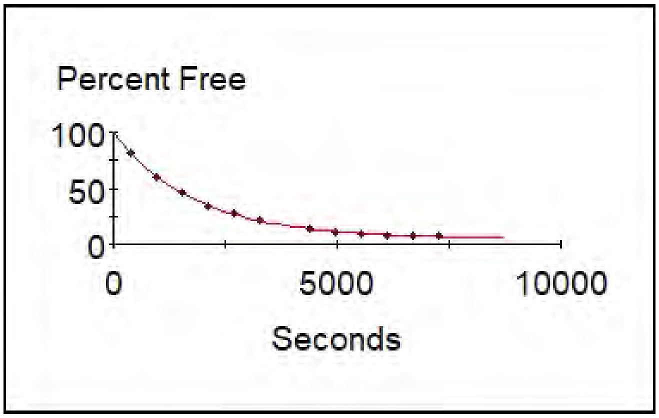

kon: 6.785e+05/Ms

95% confidence interval

kon High: 7.072e+05/Ms

kon Low: 6.509e+05/Ms

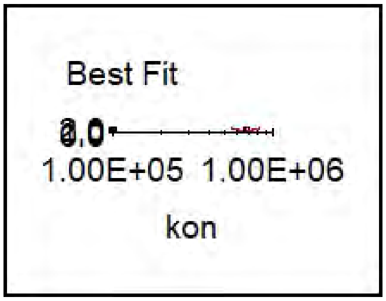

Data Traces(x)

Cycles: 13
Incubation delay (min): 0
Mix Time: Mon May 23 16:57:05 2011

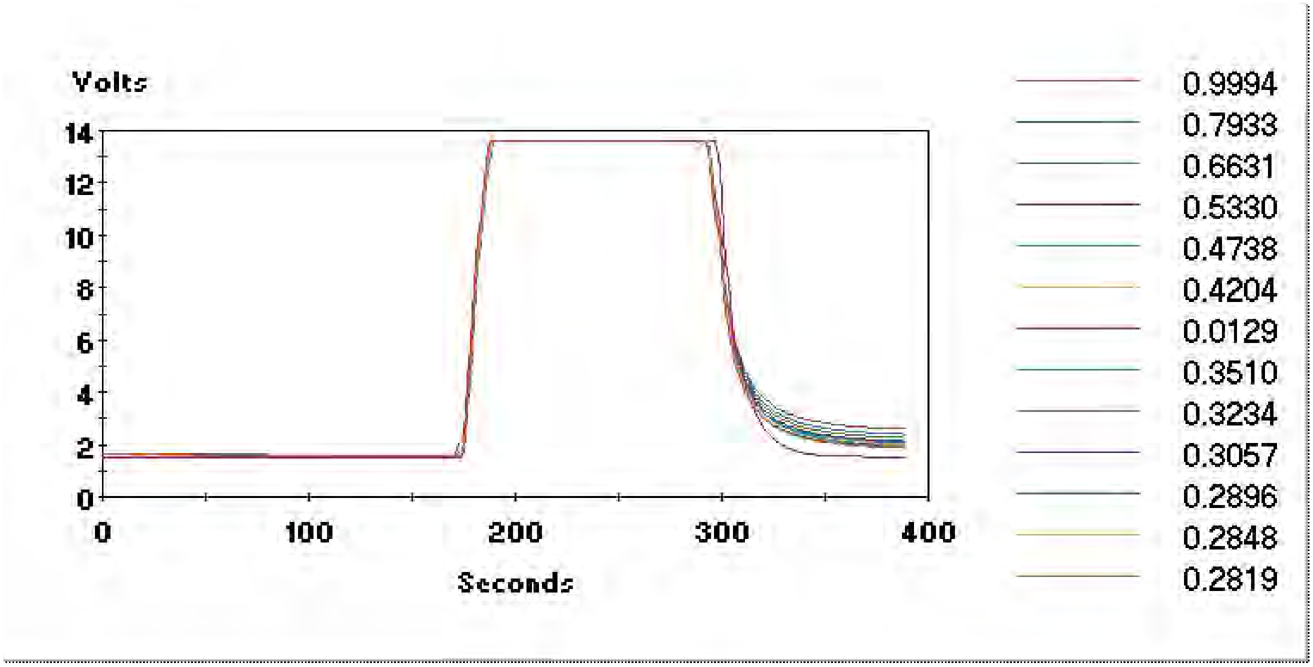

Experiment(x)

**Experiment Name:**	KinDir 2B23 IgG1 vs NXB23 domain Kon 081224	**Start Time:**	Mon Aug 12 11:36:18 2024
**Experiment Type:**	Kinetics, Direct	**End Time:**	Mon Aug 12 13:33:10 2024
**Binding Site Concentration:**	100.00pM	**Buffer:**	PBS/BSA
**Kd:**	38.45pM	**Label:**	Anti-SV5-647
**Titrant:**	200.00pM	**Label Conc:**	0

Comments(x)

beads: 2B23 IgG1 coated 07/26/24

sample volume: 500 ul

detection: Anti-SV5 -647 (1:1000)

CBP: 100 pM [final] NXB23 Domain

titrant: 200 pM [final] 2B23 IgG 08/12/24

beads: 2B23 IgG1 coated 07/26/24

sample volume: 500 ul

detection: Anti-SV5 -647 (1:1000)

CBP: 100 pM [final] NXB23 Domain

titrant: 200 pM [final] 2B23 IgG 08/12/24

Timing(x)

Bead Handling (Custom Beads)

Draw Source	Time (sec)	Volume (uL)	Rate (mL/min)	Stir
Backflush	20	0	0.0000	
Buffer	20	500	1.5000	✓
Particle Reservoir 1	26	440	1.0000	✓
Buffer	30	500	1.0000	
Waste	2	8	0.2500	
Buffer	20	0	0.0000	
Buffer	9	150	1.0000	

Sample Timing

Draw Source	Time (sec)	Volume (uL)	Rate (mL/min)	Time Stamp
Rack 2: Tube 1	120	500	0.2500	
Buffer	30	125	0.2500	
Rack 2: Tube 60	120	500	0.2500	
Buffer	30	125	0.2500	
Buffer	90	1500	1.0000	

Analysis(x)

Baseline / Endpoints:

to (sec) from beginning

to (sec) from end

Binding

**Ignore**	**Signal (V)**	**Time**
	0.4469	334.5
	0.3969	1033.5
	0.2991	1734
	0.2556	2434
	0.2400	3134.5
	0.1900	3834.5
✓	0.2179	4535.5
	0.1842	5236
	0.1652	5937.5
	0.1538	6638

**kon:**	1.783e+06/Ms
**koff:**	6.854e-05/s
**Sig 100%:**	0.51
**NSB:**	0.00
**%Error:**	2.37
**Kd:**	38.45pM
**CBP:**	100.00pM
**Titrant:**	200.00pM

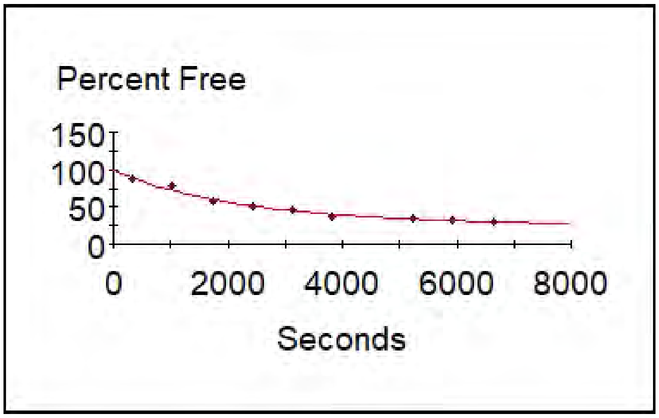

kon: 1.783e+06/Ms

95% confidence interval

kon High: 2.270e+06/Ms

kon Low: 1.348e+06/Ms

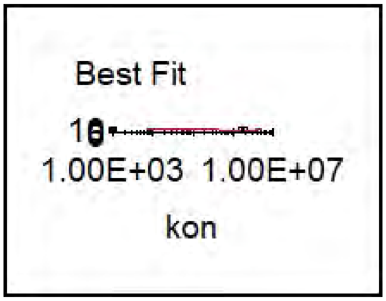

Data Traces(x)

**Cycles:**	10
**Incubation delay (min):**	0
**Mix Time:**	Mon Aug 12 11:35:44 2024

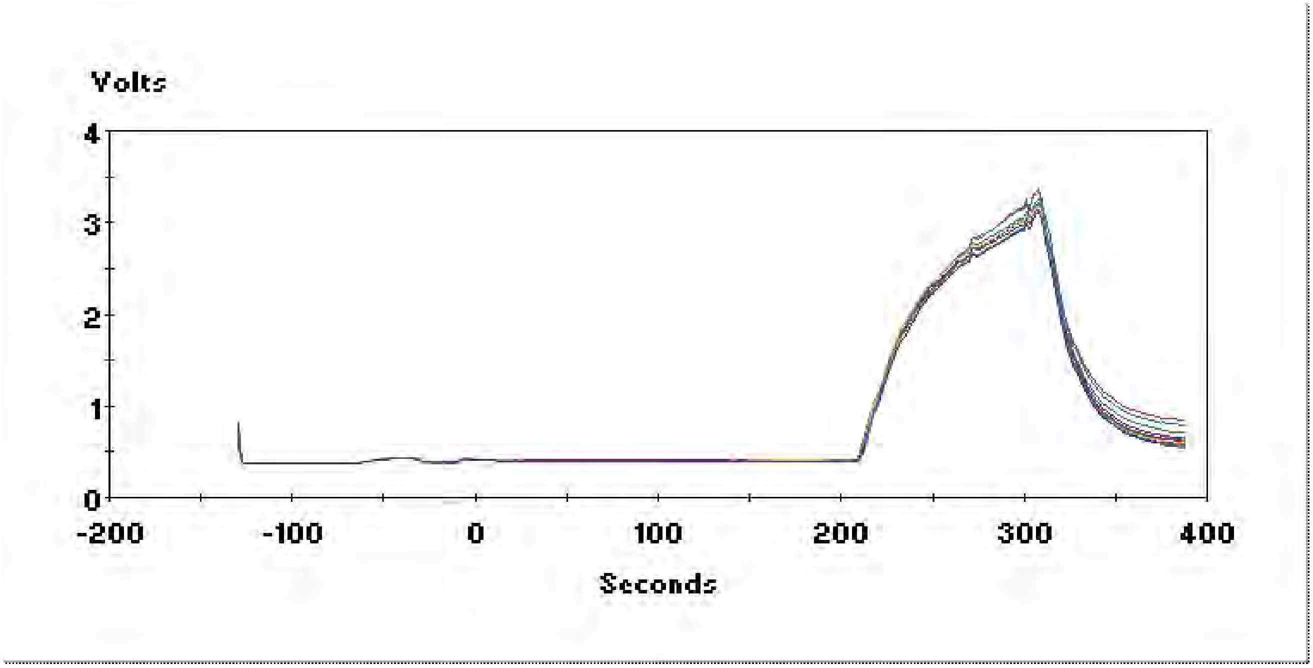

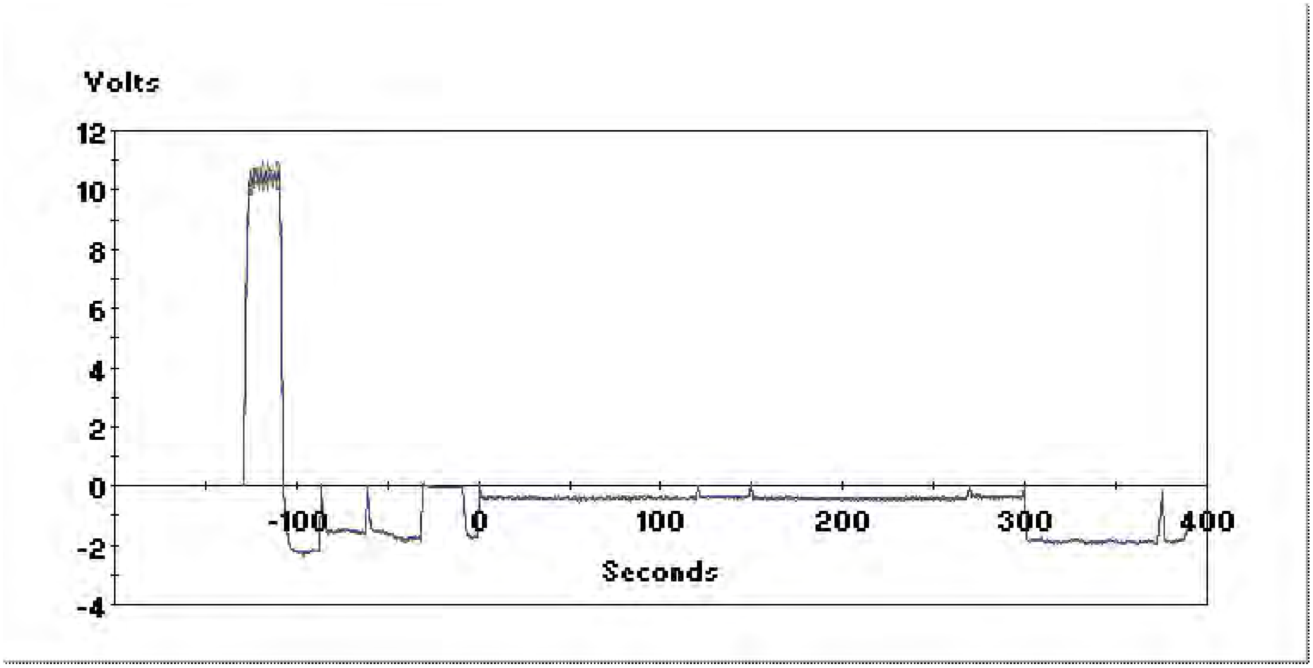

Experiment(x)

**Experiment Name:**	KinDir TsAb-B(1B10.1) vs BoNT B1{2}	**Start Time:**	Thu Mar 26 12:25:47 2015
**Experiment Type:**	Kinetics, Direct	**End Time:**	Thu Mar 26 14:26:18 2015
**Binding Site Concentration:**	200.00pM	**Buffer:**	PBS/BSA
**Kd:**	402.00fM	**Label:**	B6.1-647
**Titrant:**	800.00pM	**Label Conc:**	0

Comments(x)

beads: XB10 3/20/15

sample volume: 500 ul

detection: B6.1-647

CBP: 200 pM [final] BoNT B1 100065 3/26/15

titrant: 800 pM [final] TsAb-B 3/18/15 (260 kDa, 0.3 mg/ml, 1.154 uM

Timing(x)

Bead Handling (Custom Beads)

Draw Source	Time (sec)	Volume (uL)	Rate (mL/min)	Stir
Backflush	20	0	0.0000	
Buffer	20	500	1.5000	✓
Particle Reservoir 1	20	333	1.0000	✓
Buffer	30	500	1.0000	
Waste	2	8	0.2500	
Buffer	20	0	0.0000	
Buffer	9	150	1.0000	

Sample Timing

Draw Source	Time (sec)	Volume (uL)	Rate (mL/min)	Time Stamp
Rack 2: Tube 1	120	500	0.2500	
Buffer	30	125	0.2500	
Rack 2: Tube 60	120	500	0.2500	
Buffer	30	125	0.2500	
Buffer	90	1500	1.0000	

Analysis(x)

Baseline / Endpoints:
to (sec) from beginning
to (sec) from end

Binding		
**Ignore**	**Signal (V)**	**Time**
	0.8258	79
	0.5730	801
	0.4419	1523
	0.3698	2245
	0.3177	2967
	0.2865	3689.5
	0.2539	4412
	0.2375	5135
	0.2195	5857.5
	0.2034	6580.5

**kon:**	8.745e+05/Ms
**koff:**	3.516e-07/s
**Sig 100%:**	0.85
**NSB:**	0.20
**%Error:**	1.29
**Kd:**	402.00fM
**CBP:**	200.00pM
**Titrant:**	800.00pM

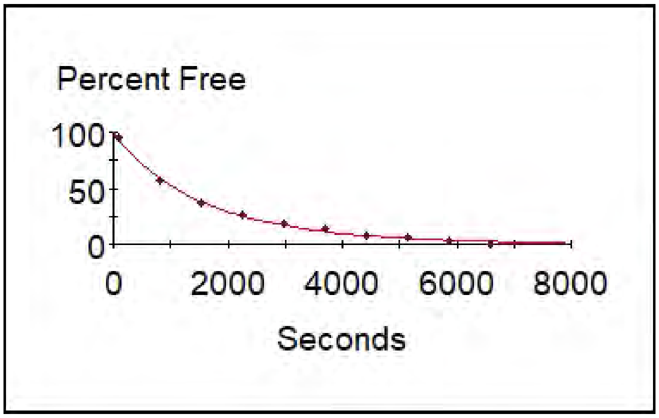

**kon:**	8.745e+05/Ms
**95% confidence interval**	
**kon High:**	9.403e+05/Ms
**kon Low:**	8.109e+05/Ms

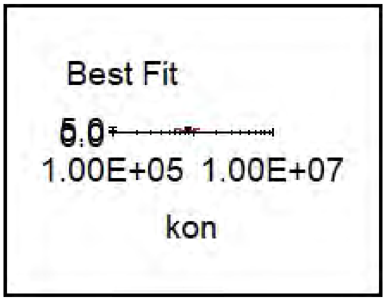

Data Traces(x)

**Cycles:**	10
**Incubation delay (min):**	0
**Mix Time:**	Thu Mar 26 12:29:48 2015

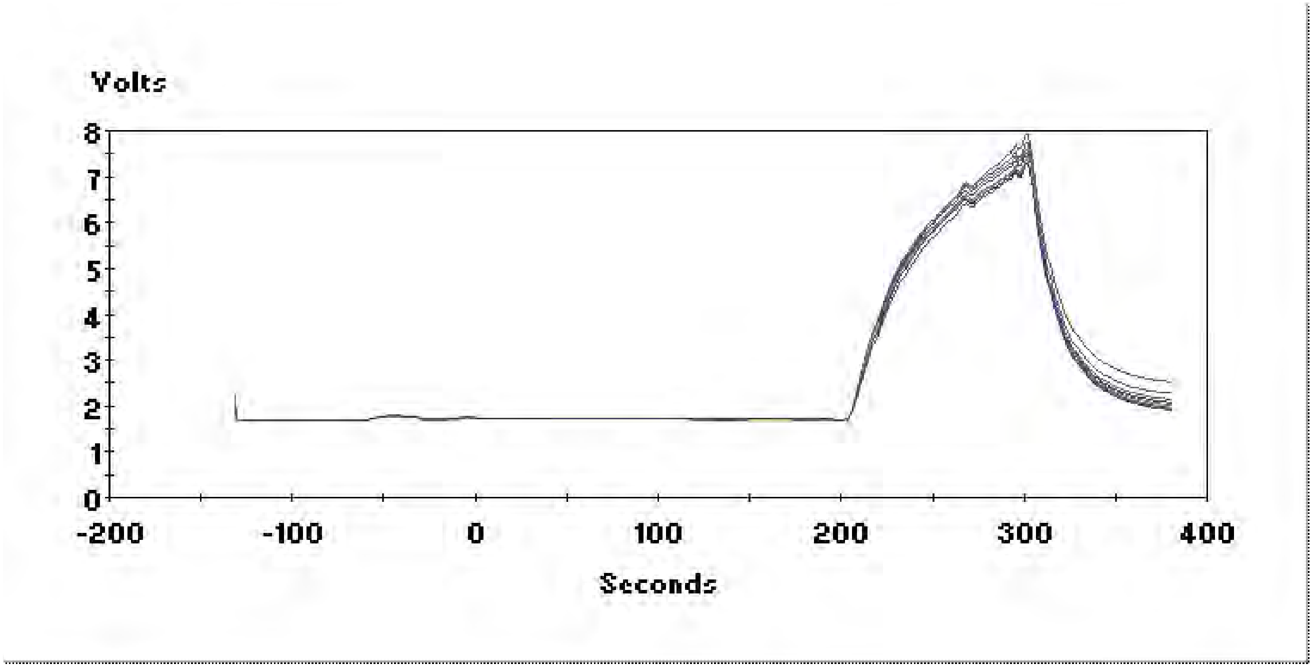

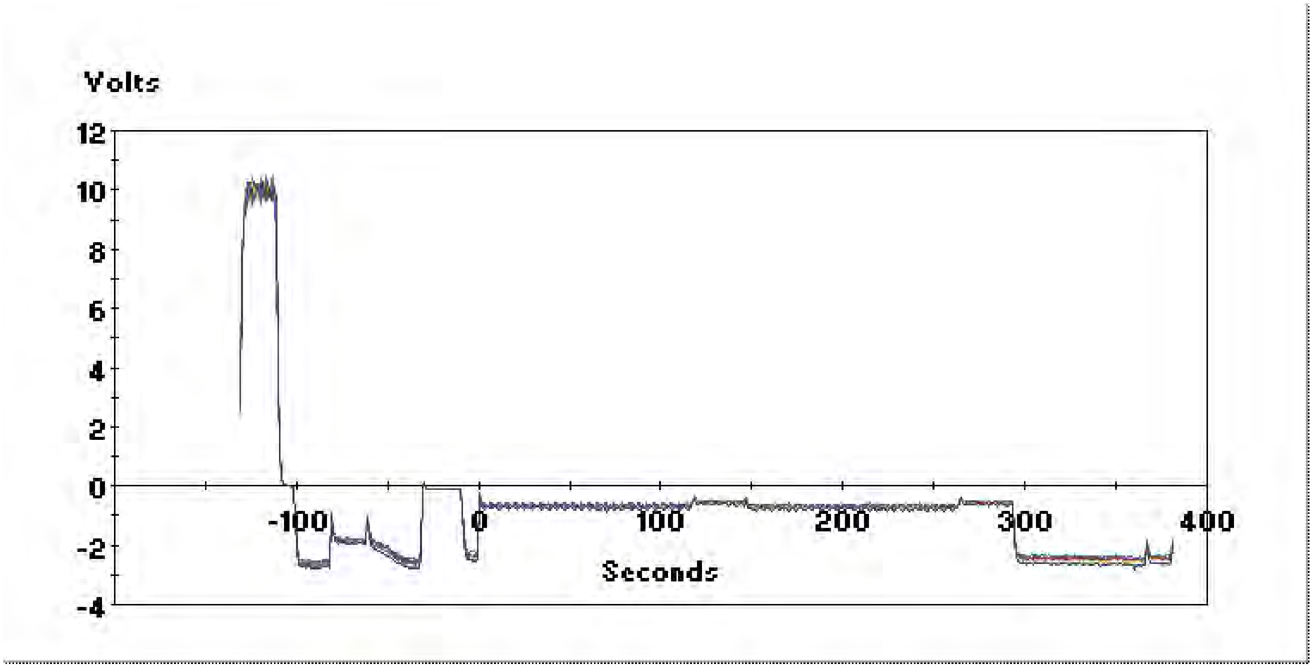

Experiment(x)

**Experiment Name:**	KinDir TsAb-B(1B10.1) vs LCHN-B10	**Start Time:**	Thu Jun 11 15:11:12 2015
**Experiment Type:**	Kinetics, Direct	**End Time:**	Thu Jun 11 17:11:42 2015
**Binding Site Concentration:**	50.00pM	**Buffer:**	PBS/BSA
**Kd:**	965.00fM	**Label:**	aSV5-647
**Titrant:**	1.00nM	**Label Conc:**	0

Comments(x)

beads: XB10 5/1/15

CBP: 100 pM [final] BoNT LCHN-B10 16907945-137 4/27/15

titrant: 1 nM [final] TsAb-B 3/18/15 (260 kDa, 0.3 mg/ml, 1.154 uM)

detection: aSV5-647

sample volume: 500 ul

Timing(x)

Bead Handling (Custom Beads)

Draw Source	Time (sec)	Volume (uL)	Rate (mL/min)	Stir
Backflush	20	0	0.0000	
Buffer	20	500	1.5000	✓
Particle Reservoir 1	18	300	1.0000	✓
Buffer	30	500	1.0000	
Waste	2	8	0.2500	
Buffer	20	0	0.0000	
Buffer	9	150	1.0000	

Sample Timing

Draw Source	Time (sec)	Volume (uL)	Rate (mL/min)	Time Stamp
Rack 2: Tube 1	120	500	0.2500	
Buffer	30	125	0.2500	
Rack 2: Tube 60	120	500	0.2500	
Buffer	30	125	0.2500	
Buffer	90	1500	1.0000	

Analysis(x)

Baseline / Endpoints:
to (sec) from beginning
to (sec) from end

Binding

Ignore	Signal (V)	Time
	1.3457	82
	1.0578	803.5
	0.8447	1525.5
	0.7202	2248
	0.6096	2970
	0.5293	3692
	0.4522	4414.5
	0.4190	5137
	0.3683	5859.5
	0.3438	6582

**kon:**	4.144e+05/Ms
**koff:**	3.999e-07/s
**Sig 100%:**	1.38
**NSB:**	0.27
**%Error:**	0.82
**Kd:**	965.00fM
**CBP:**	50.00pM
**Titrant:**	1.00nM

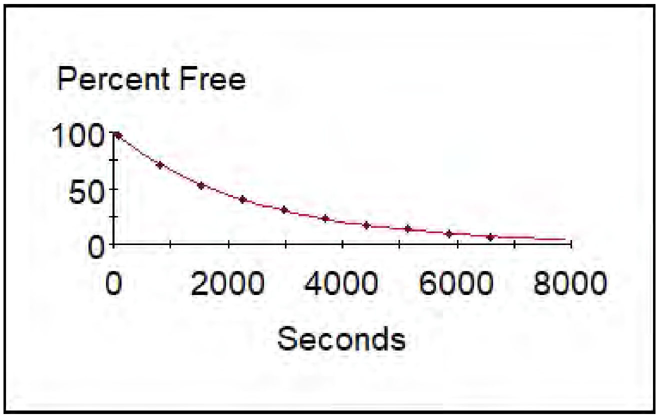

**kon:**	4.144e+05/Ms
**95% confidence interval**	
**kon High:**	4.365e+05/Ms
**kon Low:**	3.923e+05/Ms

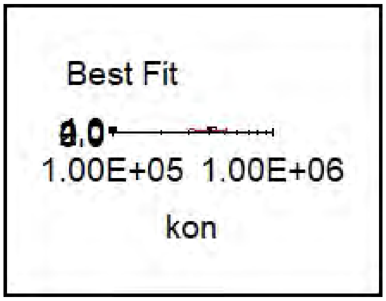

Data Traces(x)

**Cycles:**	10
**Incubation delay (min):**	0
**Mix Time:**	Thu Jun 11 15:15:09 2015

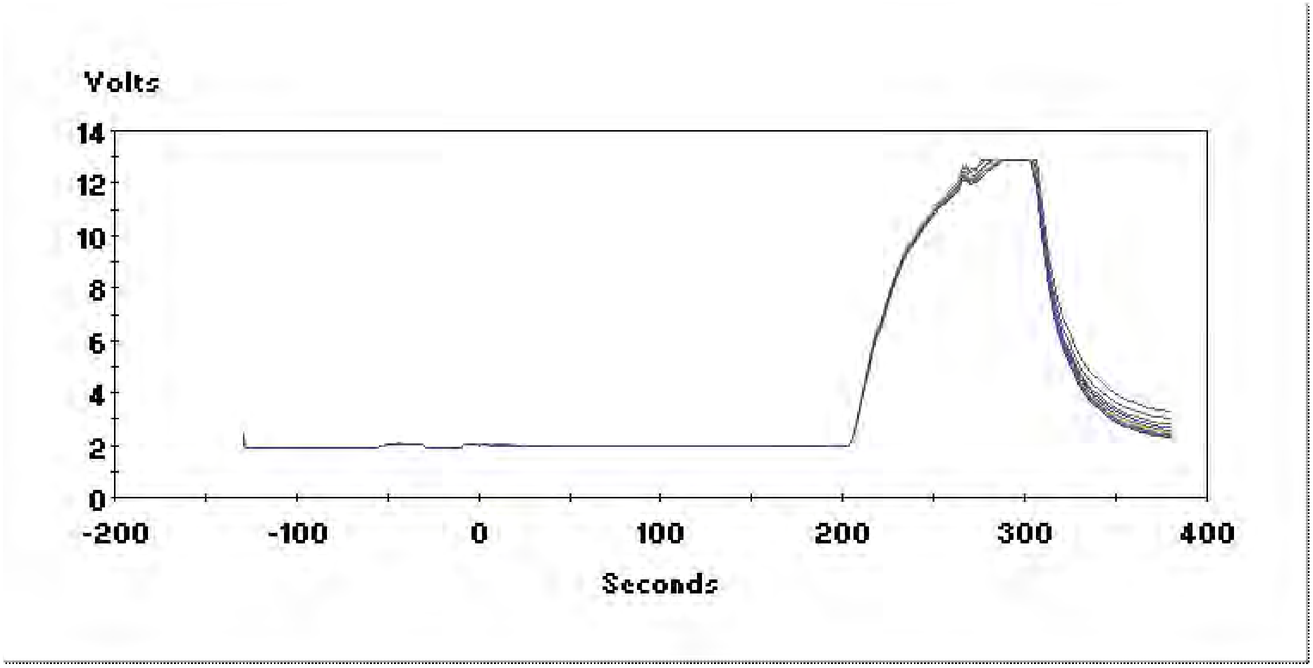

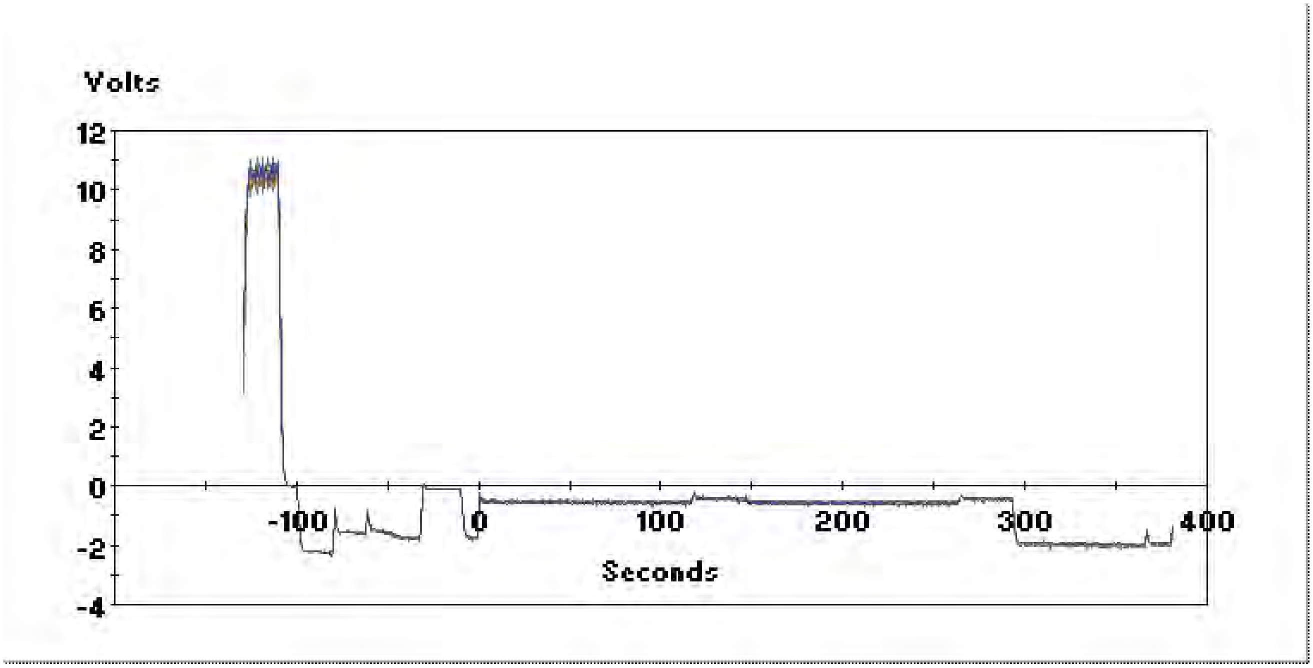

Experiment(x)

**Experiment Name:**	KinDir TsAb-B(2B18.2) vs BoNT B1	**Start Time:**	Thu Mar 26 15:02:31 2015
**Experiment Type:**	Kinetics, Direct	**End Time:**	Thu Mar 26 17:04:28 2015
**Binding Site Concentration:**	400.00pM	**Buffer:**	PBS/BSA
**Kd:**	92.84pM	**Label:**	B6.1-647
**Titrant:**	800.00pM	**Label Conc:**	0

Comments(x)

beads: XB18 3/20/15

sample volume: 500 ul

detection: B6.1-647

CBP: 400 pM [final] BoNT B1 100065 3/26/15

titrant: 800 pM [final] TsAb-B 3/18/15 (260 kDa, 0.3 mg/ml, 1.154 uM)

Timing(x)

Bead Handling (Custom Beads)

Draw Source	Time (sec)	Volume (uL)	Rate (mL/min)	Stir
Backflush	20	0	0.0000	
Buffer	20	500	1.5000	✓
Particle Reservoir 1	28	467	1.0000	✓
Buffer	30	500	1.0000	
Waste	2	8	0.2500	
Buffer	20	0	0.0000	
Buffer	9	150	1.0000	

Sample Timing

Draw Source	Time (sec)	Volume (uL)	Rate (mL/min)	Time Stamp
Rack 2: Tube 1	120	500	0.2500	
Buffer	30	125	0.2500	
Rack 2: Tube 60	120	500	0.2500	
Buffer	30	125	0.2500	
Buffer	90	1500	1.0000	

Analysis(x)

Baseline / Endpoints:

to (sec) from beginning

to (sec) from end

Binding

Ignore	Signal (V)	Time
	0.9027	80.5
	0.4884	811
	0.3153	1542
	0.2307	2272.5
	0.1871	3003
	0.1578	3734
	0.1471	4465
	0.1164	5196
	0.1068	5927.5
	0.1000	6659

**kon:**	1.146e+06/Ms
**koff:**	1.064e-04/s
**Sig 100%:**	0.97
**NSB:**	-0.06
**%Error:**	0.92
**Kd:**	92.84pM
**CBP:**	400.00pM
**Titrant:**	800.00pM

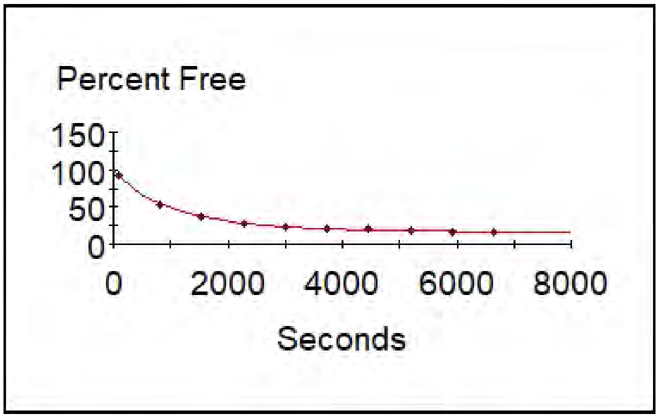

**kon:**	1.146e+06/Ms
**95% confidence interval**	
**kon High:**	1.217e+06/Ms
**kon Low:**	1.078e+06/Ms

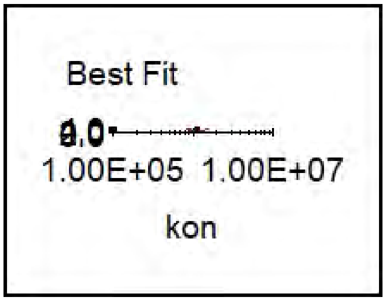

Data Traces(x)

**Cycles:**	10
**Incubation delay (min):**	0
**Mix Time:**	Thu Mar 26 15:06:39 2015

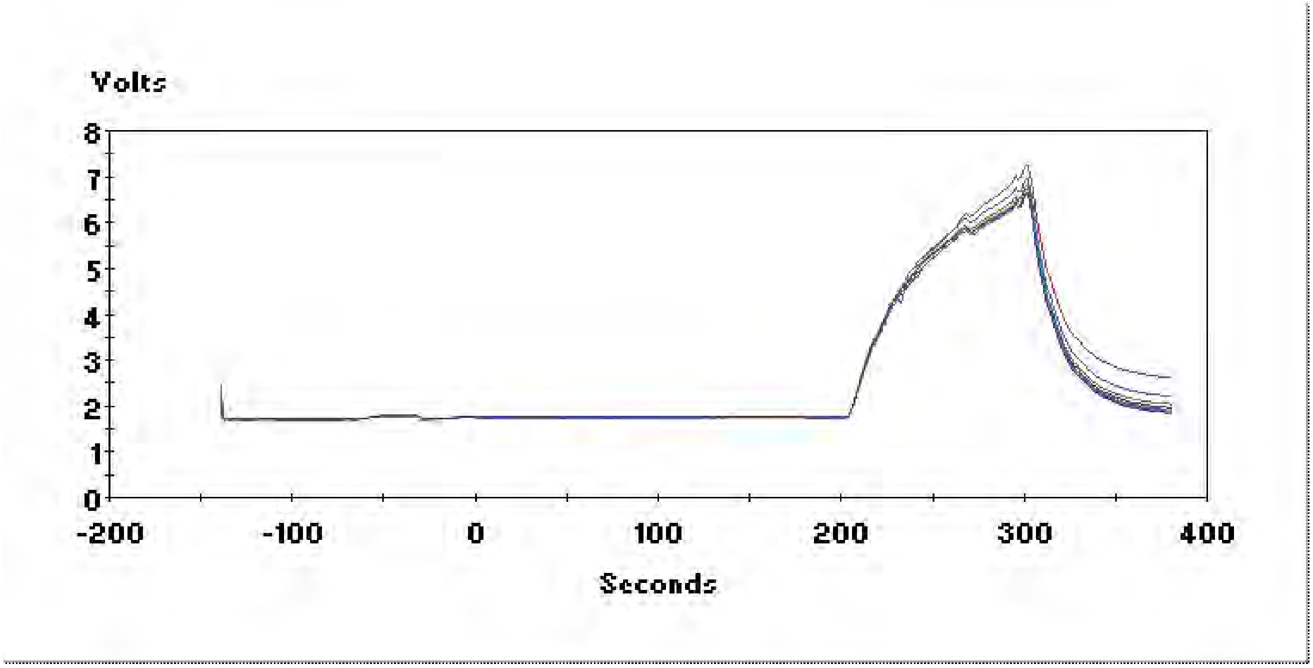

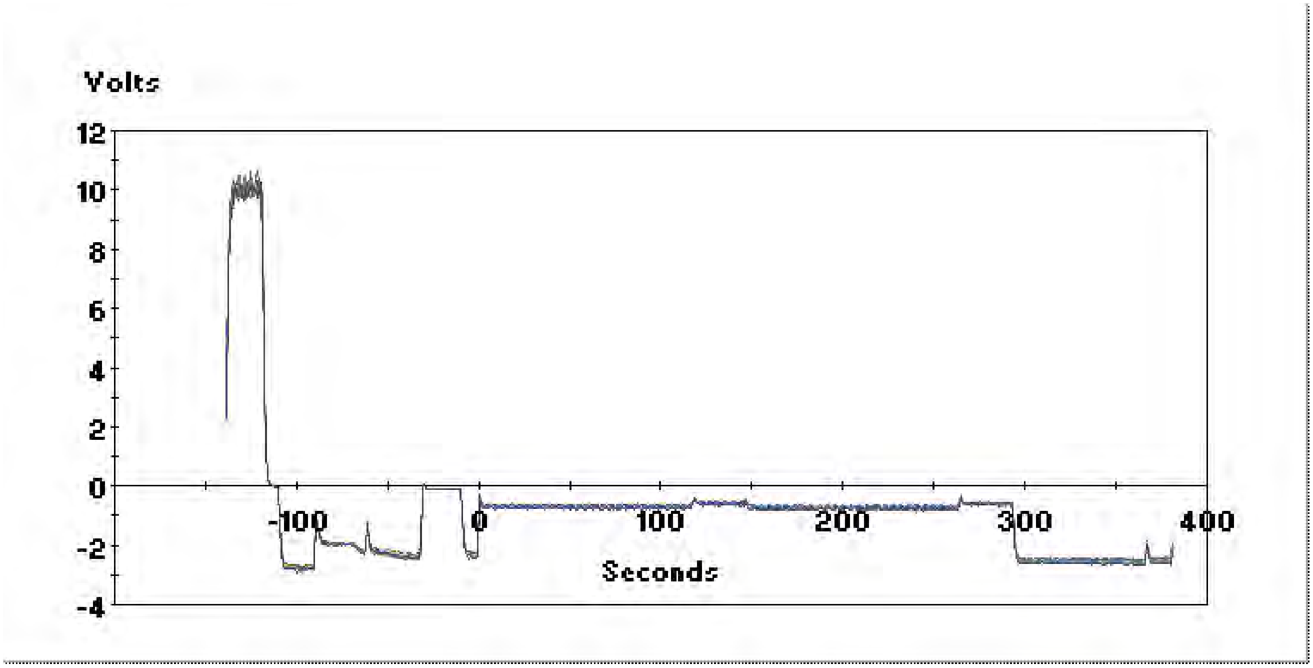

Experiment(x)

**Experiment Name:**	KinDir TsAb-B(2B18.2) vs LCHN-B18	**Start Time:**	Mon Jun 15 17:59:30 2015
**Experiment Type:**	Kinetics, Direct	**End Time:**	Mon Jun 15 20:00:20 2015
**Binding Site Concentration:**	50.00pM	**Buffer:**	PBS/BSA
**Kd:**	20.53pM	**Label:**	aSV5-647
**Titrant:**	1.00nM	**Label Conc:**	0

Comments(x)

beads: XB18 4/10/15

sample volume: 500 ul

detection: aSV5-647

CBP: 50 pM [final] BoNT LCHN-B18 23421972-1 4/27/15

titrant: 1 nM [final] TsAb-B 3/18/15 (260 kDa, 0.3 mg/ml, 1.154 uM)

Timing(x)

Bead Handling (Custom Beads)

Draw Source	Time (sec)	Volume (uL)	Rate (mL/min)	Stir
Backflush	20	0	0.0000	
Buffer	20	500	1.5000	✓
Particle Reservoir 1	20	333	1.0000	✓
Buffer	30	500	1.0000	
Waste	2	8	0.2500	
Buffer	20	0	0.0000	
Buffer	9	150	1.0000	

Sample Timing

Draw Source	Time (sec)	Volume (uL)	Rate (mL/min)	Time Stamp
Rack 2: Tube 1	120	500	0.2500	
Buffer	30	125	0.2500	
Rack 2: Tube 60	120	500	0.2500	
Buffer	30	125	0.2500	
Buffer	90	1500	1.0000	

Analysis(x)

Baseline / Endpoints:
to (sec) from beginning
to (sec) from end

Binding

Ignore	Signal (V)	Time
	1.1171	94
	0.7884	818
	0.6174	1542
	0.4959	2266
	0.4200	2990
	0.4249	3714.5
	0.3719	4439
	0.3348	5163.5
	0.3295	5888
	0.3289	6613

**kon:**	6.909e+05/Ms
**koff:**	1.418e-05/s
**Sig 100%:**	1.17
**NSB:**	0.30
**%Error:**	1.60
**Kd:**	20.53pM
**CBP:**	50.00pM
**Titrant:**	1.00nM

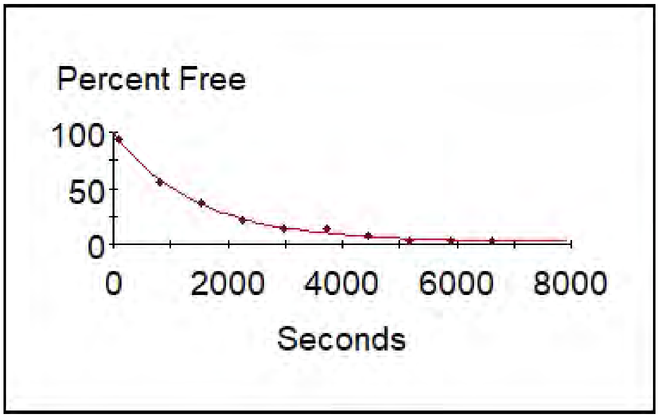

kon: 6.909e+05/Ms
95% confidence interval
kon High: 7.488e+05/Ms
kon Low: 6.364e+05/Ms

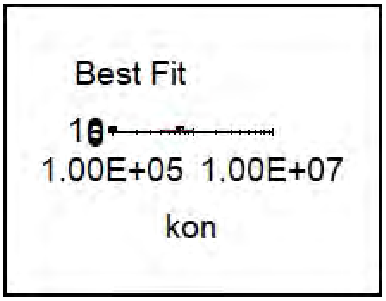

Data Traces(x)

**Cycles:**	10
**Incubation delay (min):**	0
**Mix Time:**	Mon Jun 15 18:03:17 2015

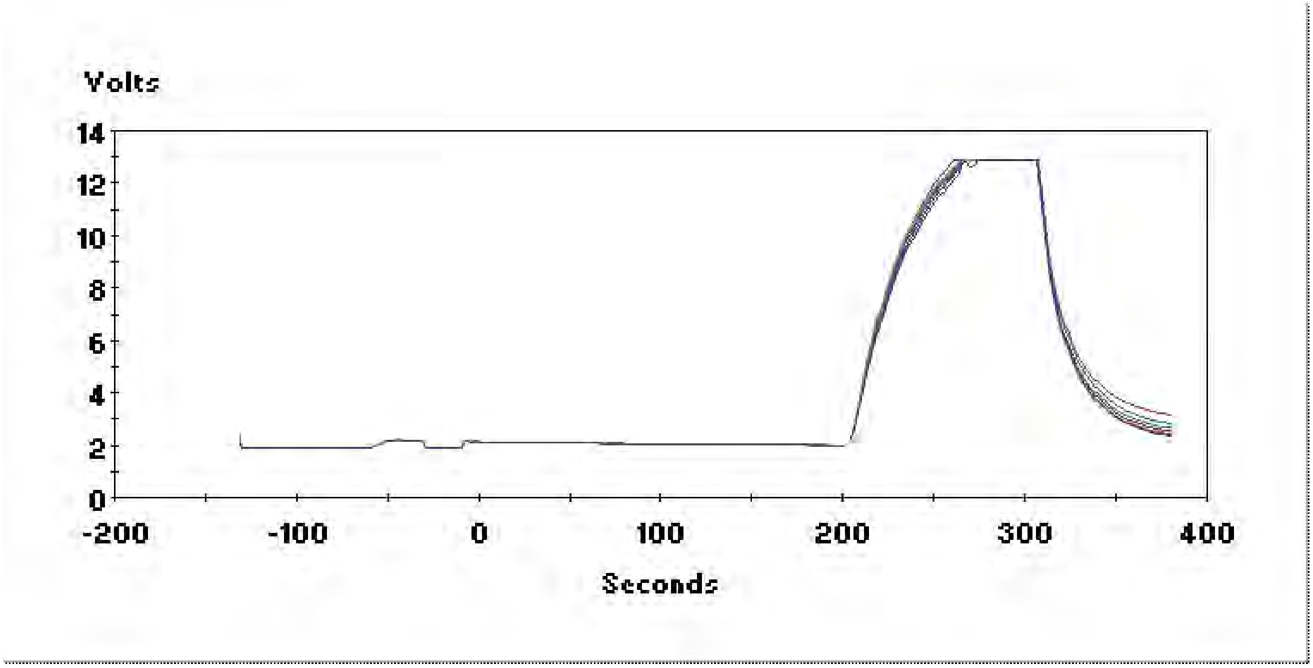

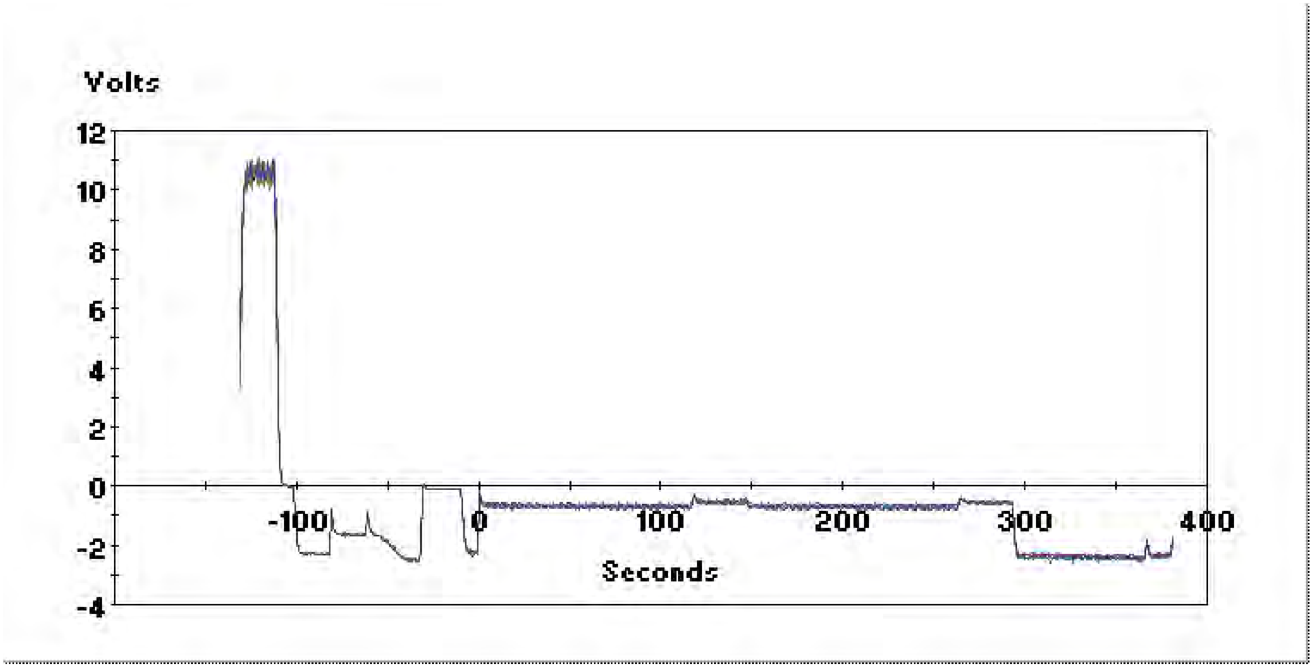

Experiment(x)

**Experiment Name:**	KinDir TsAb-B(2B23.1) vs BoNT B1	**Start Time:**	Thu Mar 26 17:44:38 2015
**Experiment Type:**	Kinetics, Direct	**End Time:**	Thu Mar 26 19:45:09 2015
**Binding Site Concentration:**	120.00pM	**Buffer:**	PBS/BSA
**Kd:**	254.00fM	**Label:**	B6.1-647
**Titrant:**	800.00pM	**Label Conc:**	0

Comments(x)

beads: XB23 3/20/15

sample volume: 500 ul

detection: B6.1-647

CBP: 120 pM [final] BoNT B1 100065 3/26/15

titrant: 800 pM [final] TsAb-B 3/18/15 (260 kDa, 0.3 mg/ml, 1.154 uM)

Timing(x)

Bead Handling (Custom Beads)

Draw Source	Time (sec)	Volume (uL)	Rate (mL/min)	Stir
Backflush	20	0	0.0000	
Buffer	20	500	1.5000	✓
Particle Reservoir 1	20	333	1.0000	✓
Buffer	30	500	1.0000	
Waste	2	8	0.2500	
Buffer	20	0	0.0000	
Buffer	9	150	1.0000	

Sample Timing

Draw Source	Time (sec)	Volume (uL)	Rate (mL/min)	Time Stamp
Rack 2: Tube 1	120	500	0.2500	
Buffer	30	125	0.2500	
Rack 2: Tube 60	120	500	0.2500	
Buffer	30	125	0.2500	
Buffer	90	1500	1.0000	

Analysis(x)

Baseline / Endpoints:
to (sec) from beginning
to (sec) from end

Binding		
Ignore	Signal (V)	Time
	1.1151	80
	0.5925	802
	0.4518	1524
	0.3493	2246
	0.2871	2968.5
	0.2771	3690.5
	0.2651	4413
	0.2383	5136
	0.2305	5858.5
	0.2252	6581.5

**kon:**	1.458e+06/Ms
**koff:**	3.704e-07/s
**Sig 100%:**	1.19
**NSB:**	0.24
**%Error:**	2.02
**Kd:**	254.00fM
**CBP:**	120.00pM
**Titrant:**	800.00pM

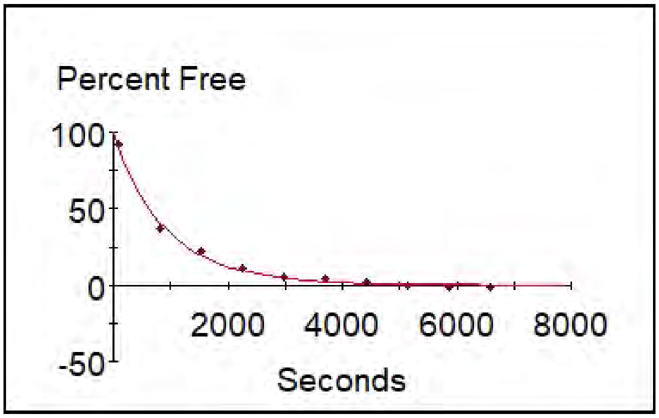

**kon:**	1.458e+06/Ms
**95% confidence interval**	
**kon High:**	1.621e+06/Ms
**kon Low:**	1.313e+06/Ms

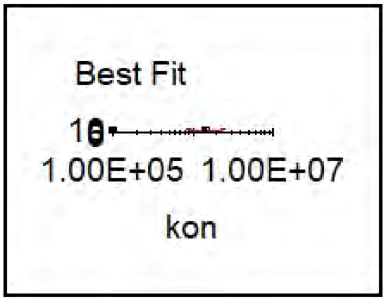

Data Traces(x)

**Cycles:**	10
**Incubation delay (min):**	0
**Mix Time:**	Thu Mar 26 17:48:38 2015

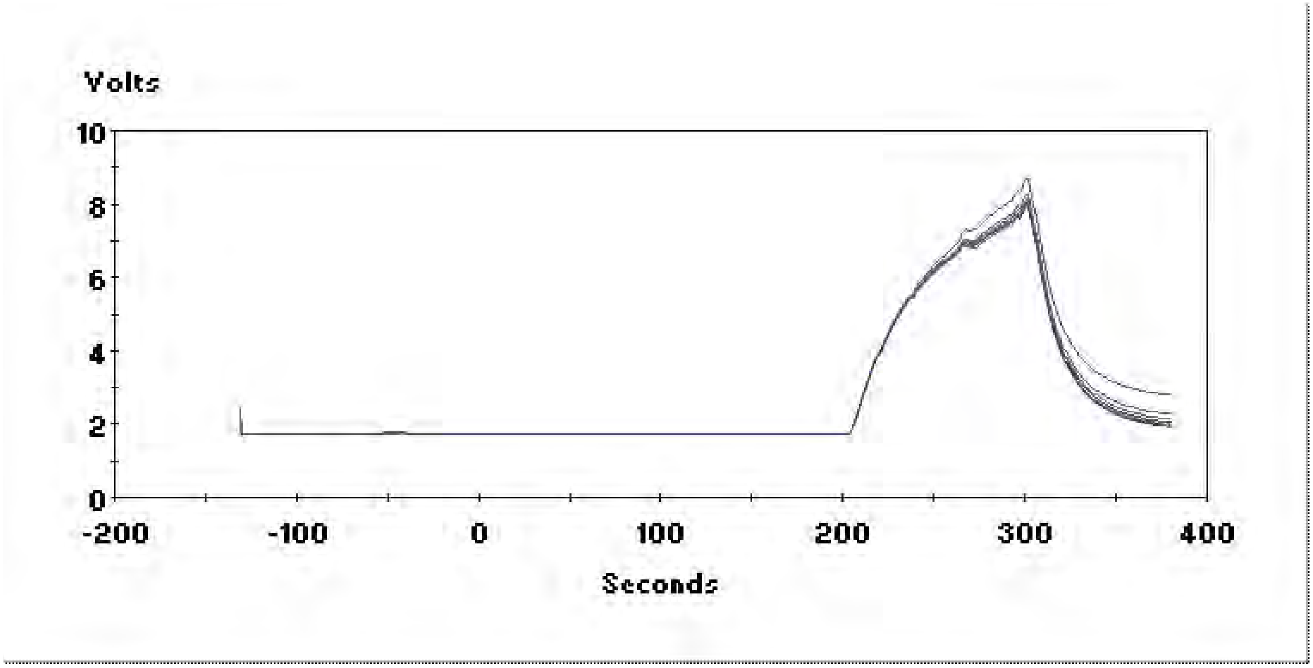

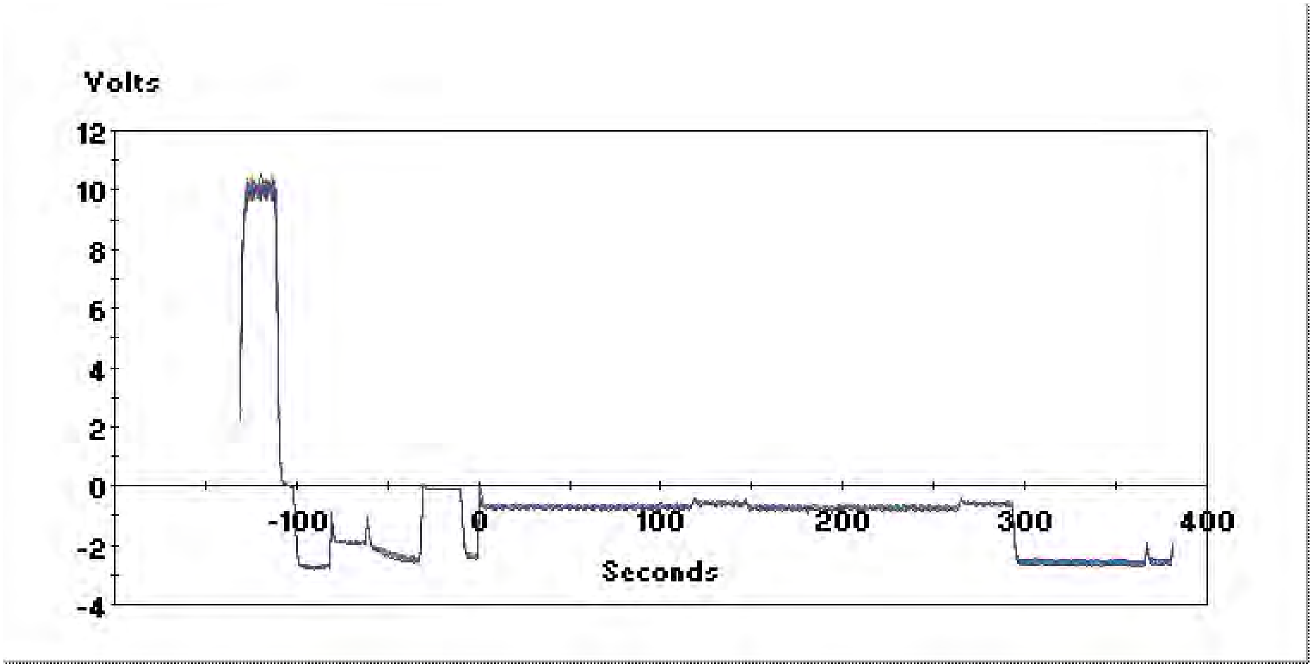

Experiment(x)

**Experiment Name:**	KinDir TsAb-B(2B23.1) vs LCHN-B23domain 061515	**Start Time:**	Tue Jun 16 15:20:18 2015
**Experiment Type:**	Kinetics, Direct	**End Time:**	Tue Jun 16 17:21:09 2015
**Binding Site Concentration:**	50.00pM	**Buffer:**	PBS/BSA
**Kd:**	18.46pM	**Label:**	aSV5-647
**Titrant:**	1.00nM	**Label Conc:**	0

Comments(x)

beads: XB23 4/10/15

sample volume: 500 ul

detection: aSV5-647

CBP: 50 pM [final] BoNT LCHN-B23 23421972-6 4/27/15

titrant: 1 nM [final] TsAb-B 3/18/15 (260 kDa, 0.3 mg/ml, 1.154 uM)

Timing(x)

Bead Handling (Custom Beads)

Draw Source	Time (sec)	Volume (uL)	Rate (mL/min)	Stir
Backflush	20	0	0.0000	
Buffer	20	500	1.5000	✓
Particle Reservoir 1	20	333	1.0000	✓
Buffer	30	500	1.0000	
Waste	2	8	0.2500	
Buffer	20	0	0.0000	
Buffer	9	150	1.0000	

Sample Timing

Draw Source	Time (sec)	Volume (uL)	Rate (mL/min)	Time Stamp
Rack 2: Tube 1	120	500	0.2500	
Buffer	30	125	0.2500	
Rack 2: Tube 60	120	500	0.2500	
Buffer	30	125	0.2500	
Buffer	90	1500	1.0000	

Analysis(x)

Baseline / Endpoints:
to (sec) from beginning
to (sec) from end

Binding

Ignore	Signal (V)	Time
	1.3556	74
	1.0021	798
	0.7563	1522
	0.6038	2246
	0.4917	2970.5
	0.4056	3695
	0.3746	4419
	0.3143	5144
	0.2805	5868.5
	0.2627	6593.5

**kon:**	5.052e+05/Ms
**koff:**	9.325e-06/s
**Sig 100%:**	1.40
**NSB:**	0.20
**%Error:**	0.69
**Kd:**	18.46pM
**CBP:**	50.00pM
**Titrant:**	1.00nM

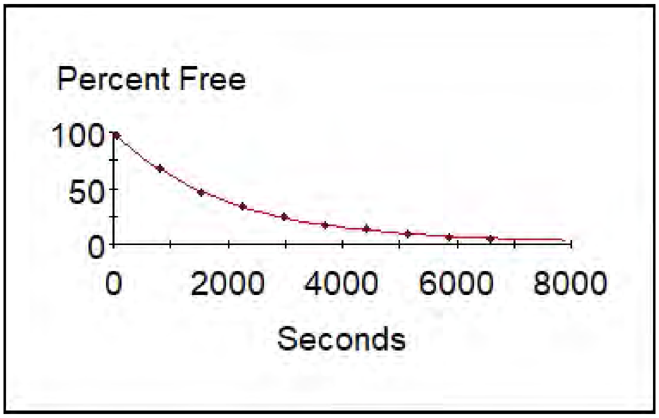

kon: 5.052e+05/Ms
95% confidence interval
kon High: 5.250e+05/Ms
kon Low: 4.854e+05/Ms

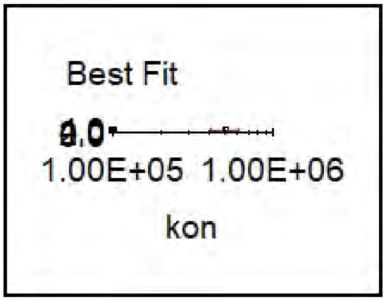

Data Traces(x)

**Cycles:**	10
**Incubation delay (min):**	0
**Mix Time:**	Tue Jun 16 15:24:25 2015

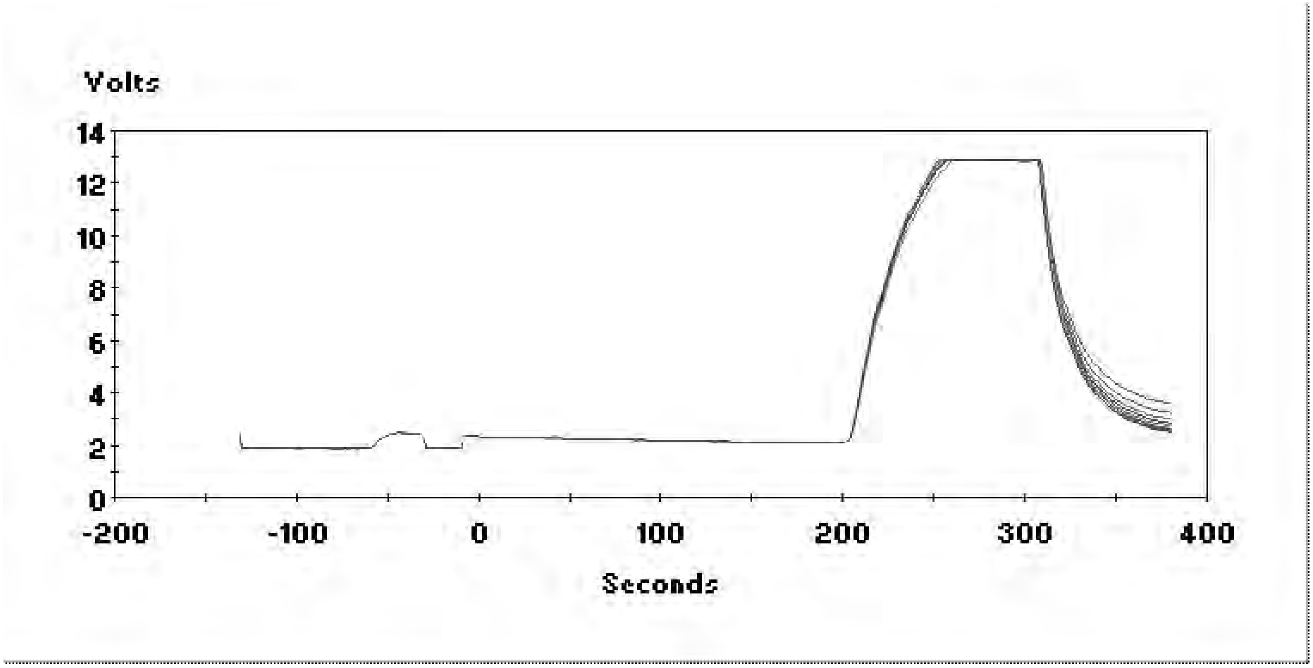

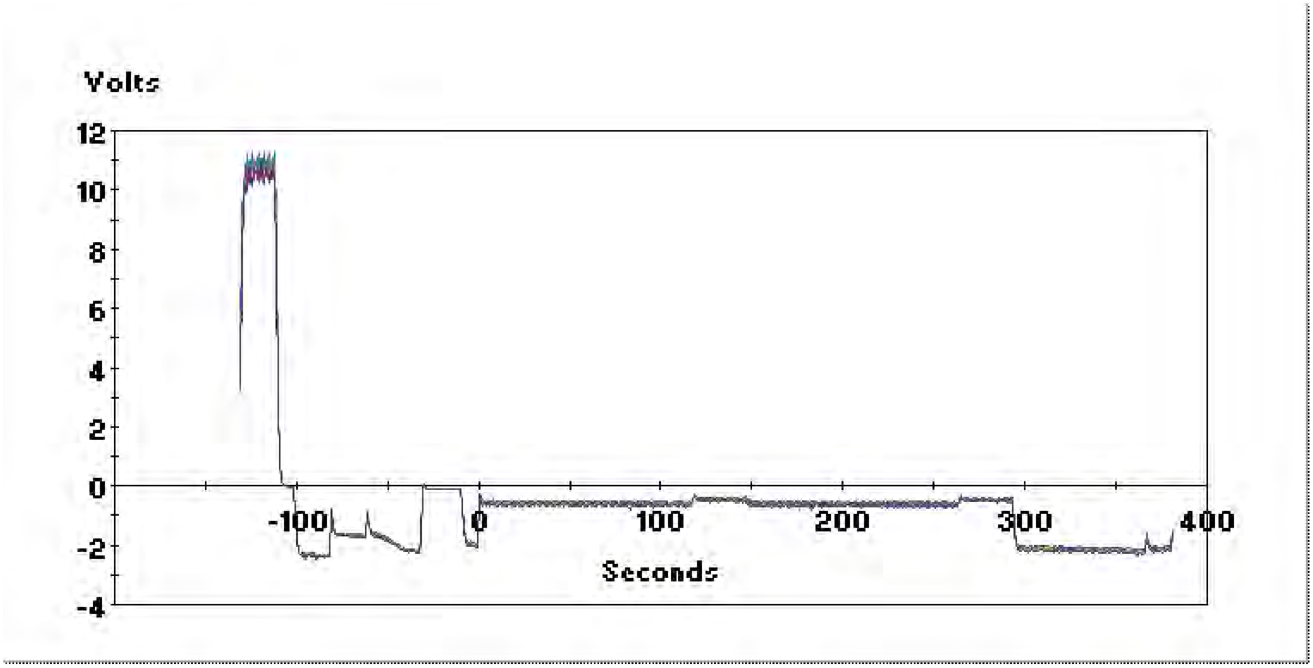

Supplement: Supplementary file 1 [file toxins-17-00281-s001.zip › Fig S1.pdf]
