# Supplementary material for: Single Tri-Epitopic Antibodies (TeAbs) to Botulinum Neurotoxin Serotypes B, E, and F Recapitulate the Full Potency of a Combination of Three Monoclonal Antibodies in Toxin Neutralization
Source: Toxins (Basel). 2025 Jun 4;17(6):281. doi: 10.3390/toxins17060281 (PMC12197607; doi:10.3390/toxins17060281)

Experiment(x)

**Experiment Name:**	Standard Kd 3E2 (beads)-BoNTE3 3E4.1-647	**Start Time:**	Fri Dec 01 17:33:26 2006
**Experiment Type:**	Equilibrium	**End Time:**	Mon Dec 04 15:10:52 2006
**Constant Binding Partner (CBP)**		**Buffer:**	pbs/bsa
**Molecular Concentration:**	3.00pM	**Label:**	4E17.1-647
**Valency:**	1	**Label Conc:**	0
**Binding Site Concentration:**	3.00pM		

Comments(x)

3E4.1-647

Timing(x)

Bead Handling (Custom Beads)

Draw Source	Time (sec)	Volume (uL)	Rate (mL/min)	Stir
Backflush	30	0	0.0000	
Buffer	20	500	1.5000	✓
Particle Reservoir	27	450	1.0000	✓
Buffer	40	333	0.5000	
Waste	5	25	0.3000	
Buffer	2	10	0.3000	
Buffer	20	0	0.0000	
Buffer	9	150	1.0000	

Sample Timing

Draw Source	Time (sec)	Volume (uL)	Rate (mL/min)	Time Stamp
Sample Set 1-13	2880	12000	0.2500	
Buffer	30	125	0.2500	
Inject	120	500	0.2500	
Buffer	30	125	0.2500	
Buffer	120	2000	1.0000	

Analysis(x)

Baseline / Endpoints:
5 to 10 (sec) from beginning
10 to 5 (sec) from end

Ignore	Binding Signal (V)	Concentration
	0.0695	500.00pM
	0.0884	250.00pM
	0.0916	125.00pM
	0.1081	62.50pM
	0.1422	31.25pM
	0.2085	15.63pM
	0.3082	7.81pM
	0.4658	3.90pM
	0.6524	1.95pM
	0.7759	976.56fM
	0.8584	488.28fM
	0.8689	244.14fM
	0.9055	122.07fM
	0.0723	500.00pM
	0.0704	250.00pM
	0.0901	125.00pM
	0.1111	62.50pM
	0.1231	31.25pM
	0.2016	15.63pM
	0.2933	7.81pM
	0.4397	3.90pM
	0.6408	1.95pM
	0.7582	976.56fM
	0.8269	488.28fM
	0.8808	244.14fM
	0.9123	122.07fM

**Kd:**	2.28pM
**Active CBP:**	2.23pM
**CBP %Activity:**	74.48
**Ratio:**	0.9797
**Sig 100%:**	0.93
**Drift (%/run):**	0.0165
**NSB:**	0.07
**Drift (mV/run):**	-0.6274
**%Error:**	1.08

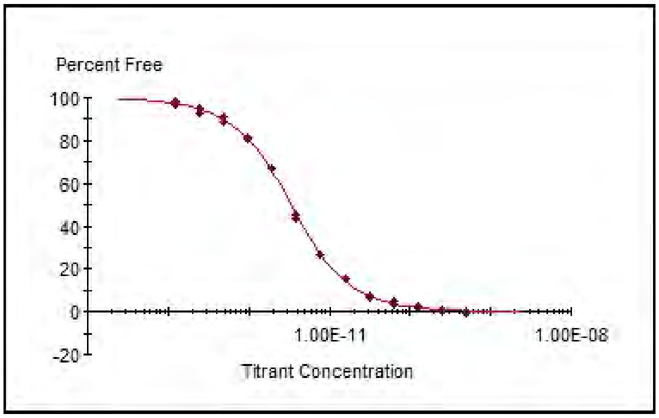

**Kd:**	2.28pM
**95% confidence interval**	
**Kd High:**	2.71pM
**Kd Low:**	1.89pM

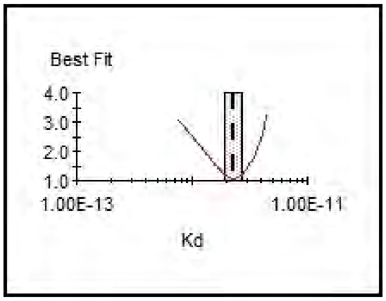

**Active CBP:**	2.23pM
**CBP %Activity:**	74.48
**95% confidence interval**	
**CBP High:**	3.32pM
**%Activity:**	110.67
**CBP Low:**	1.08pM
**%Activity:**	35.94

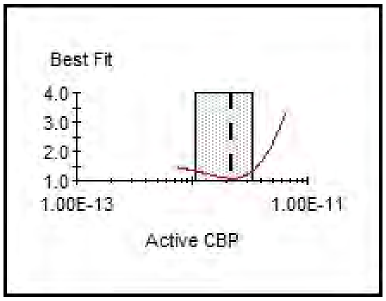

Data Traces(x)

Cycles: 2
Incubation delay (min): 2707
Mix Time:

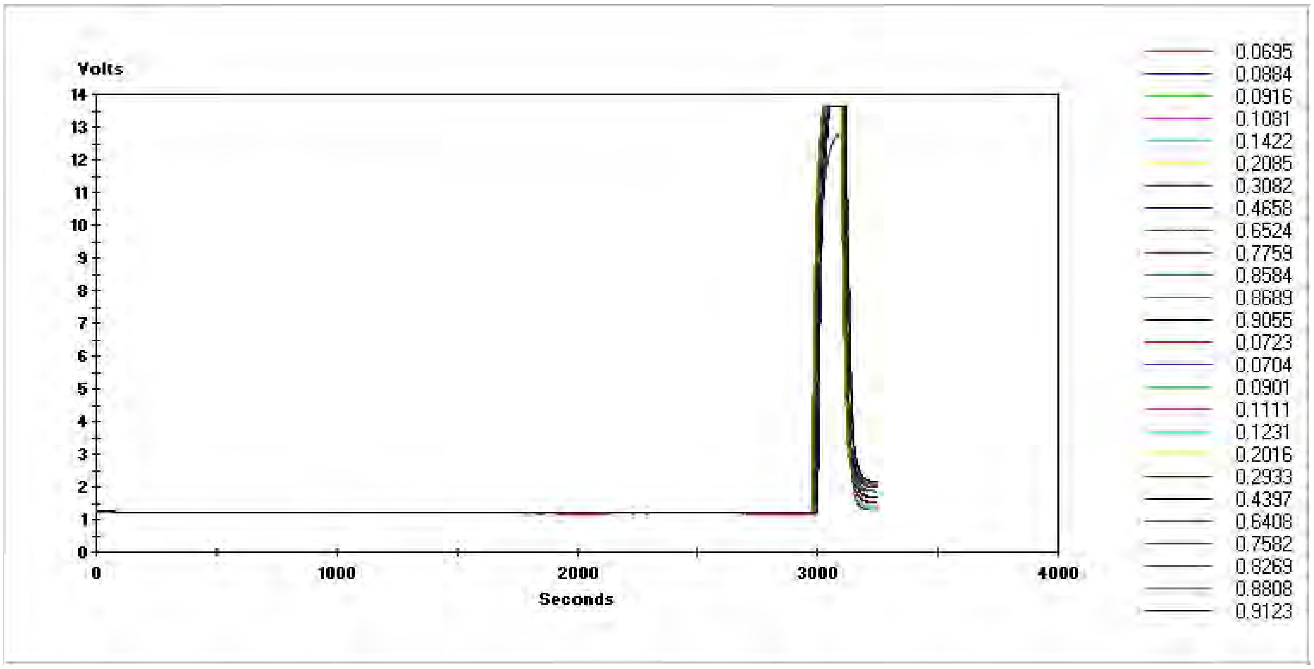

Experiment(x)

**Experiment Name:**	Kd XE02 vs 3E2 E1 LCHN mut 012312	**Start Time:**	Mon Jan 23 13:25:53 2012
**Experiment Type:**	Equilibrium	**End Time:**	Mon Jan 23 22:18:37 2012
**Constant Binding Partner (CBP)**		**Buffer:**	pbs/bsa
**Molecular Concentration:**	10.00pM	**Label:**	aSV5-647
**Valency:**	1	**Label Conc:**	0
**Binding Site Concentration:**	10.00pM		

Comments(x)

3E2 beads 1/23/12

BoNT E1 LCHN Q608A-Q609A-E730A (date unknown)

XE02 041111

aSV5-647

meter: 1.0075

Timing(x)

Bead Handling (Custom Beads)

Draw Source	Time (sec)	Volume (uL)	Rate (mL/min)	Stir
Backflush	30	0	0.0000	
Buffer	20	500	1.5000	✓
Particle Reservoir	28	467	1.0000	✓
Buffer	40	333	0.5000	
Waste	5	25	0.3000	
Buffer	2	10	0.3000	
Buffer	20	0	0.0000	
Buffer	9	150	1.0000	

Sample Timing

Draw Source	Time (sec)	Volume (uL)	Rate (mL/min)	Time Stamp
Sample Set 1-13	720	3000	0.2500	
Buffer	30	125	0.2500	
Inject	120	500	0.2500	
Buffer	30	125	0.2500	
Buffer	120	2000	1.0000	

Analysis(x)

Baseline / Endpoints:
to (sec) from beginning
to (sec) from end

Ignore	Binding Signal (V)	Concentration
	0.2934	1.00nM
	0.3439	500.00pM
	0.3507	250.00pM
	0.4460	125.00pM
	0.6053	62.50pM
	0.7503	31.25pM
	0.9392	15.63pM
	1.1537	7.81pM
	1.2277	3.90pM
	1.2067	1.95pM
	1.3096	976.56fM
	1.3244	488.28fM
	1.3633	244.14fM
	0.2532	1.00nM
	0.2977	500.00pM
	0.3264	250.00pM
	0.4123	125.00pM
	0.4829	62.50pM
	0.7292	31.25pM
	0.9560	15.63pM
	1.0713	7.81pM
	1.1846	3.90pM
	1.2427	1.95pM
	1.2813	976.56fM
	1.3038	488.28fM
	1.3447	244.14fM

**Kd:**	22.09pM
**Active CBP:**	8.59pM
**CBP %Activity:**	85.88
**Ratio:**	0.3887
**Sig 100%:**	1.34
**Drift (%/run):**	-0.2414
**NSB:**	0.24
**Drift (mV/run):**	-3.8810
**%Error:**	2.23

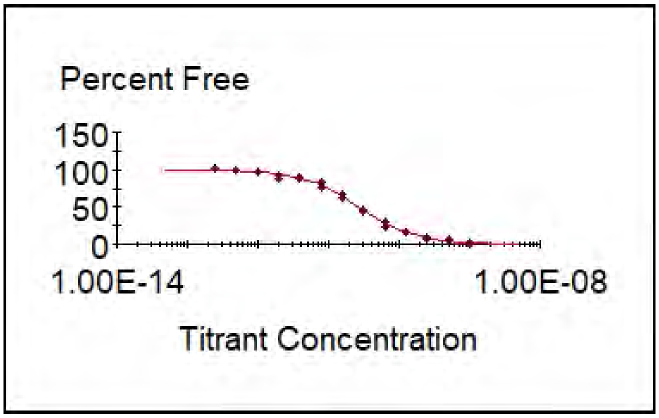

**Kd:**	22.09pM
**95% confidence interval**	
**Kd High:**	30.26pM
**Kd Low:**	14.20pM

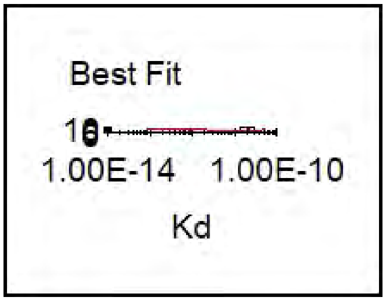

**Active CBP:**	8.59pM
**CBP %Activity:**	85.88
**95% confidence interval**	
**CBP High:**	27.68pM
**%Activity:**	276.79
**CBP Low:**	Less than 31.03fM
**%Activity:**	Less than 0.31

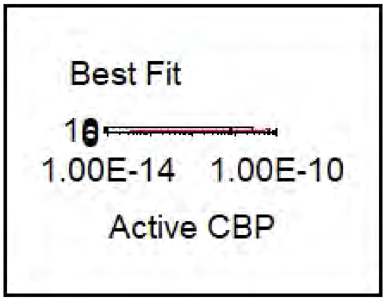

Data Traces(x)

Cycles: 2
Incubation delay (min): 0
Mix Time:

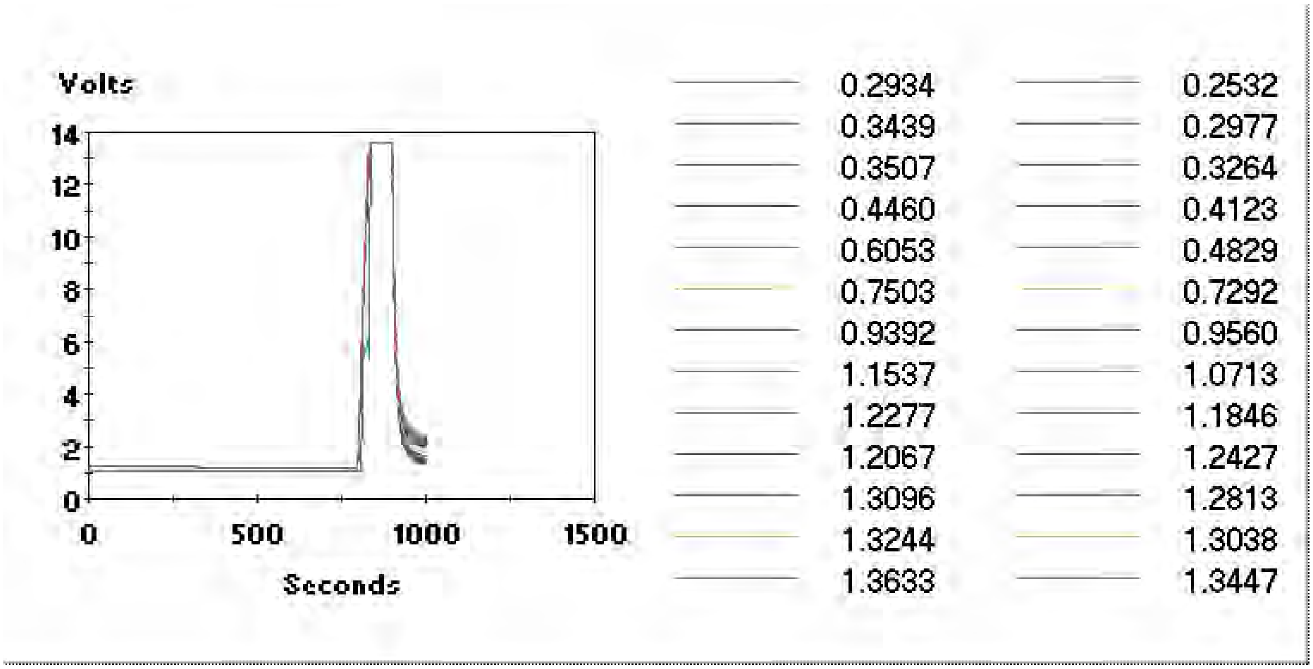

Experiment(x)

**Experiment Name:**	KD 3E6.2 vs E3	**Start Time:**	Mon Mar 17 12:39:51 2008
**Experiment Type:**	Equilibrium	**End Time:**	Tue Mar 18 13:06:22 2008
**Constant Binding Partner (CBP)**		**Buffer:**	pbs/bsa
**Molecular Concentration:**	8.00pM	**Label:**	3E2-647
**Valency:**	1	**Label Conc:**	400.00ng/ml
**Binding Site Concentration:**	8.00pM		

Comments(x)

3E6.1 beads 3/12/08

BoNT E3 11/19/07

3E6.2 IgG 12/3/07

3E2-647 1:2000

meter: 0.7047

Timing(x)

Bead Handling (Custom Beads)

Draw Source	Time (sec)	Volume (uL)	Rate (mL/min)	Stir
Backflush	30	0	0.0000	
Buffer	20	500	1.5000	✓
Particle Reservoir	20	333	1.0000	✓
Buffer	40	333	0.5000	
Waste	5	25	0.3000	
Buffer	2	10	0.3000	
Buffer	20	0	0.0000	
Buffer	9	150	1.0000	

Sample Timing

Draw Source	Time (sec)	Volume (uL)	Rate (mL/min)	Time Stamp
Sample Set 1-13	2880	12000	0.2500	
Buffer	30	125	0.2500	
Inject	120	500	0.2500	
Buffer	30	125	0.2500	
Buffer	120	2000	1.0000	

Analysis(x)

Baseline / Endpoints:
2500 to 2505 (sec) from beginning
10 to 5 (sec) from end

Ignore	Binding Signal (V)	Concentration
	0.1160	600.00pM
	0.1166	300.00pM
	0.1603	150.00pM
	0.2239	75.00pM
	0.3929	37.50pM
	0.6784	18.75pM
	1.0342	9.38pM
	1.2554	4.69pM
	1.3996	2.34pM
	1.4577	1.17pM
	1.4474	585.94fM
	1.4737	292.97fM
	1.4628	146.48fM
	0.1333	600.00pM
	0.1409	300.00pM
	0.1934	150.00pM
	0.2971	75.00pM
	0.4877	37.50pM
	0.7597	18.75pM
	0.9965	9.38pM
	1.2019	4.69pM
	1.3640	2.34pM
	1.3328	1.17pM
	1.5383	585.94fM
	1.6063	292.97fM
✓	1.0038	146.48fM

**Kd:**	8.55pM
**Active CBP:**	13.14pM
**CBP %Activity:**	164.25
**Ratio:**	1.5371
**Sig 100%:**	1.51
**Drift (%/run):**	0.1595
**NSB:**	0.11
**Drift (mV/run):**	3.2997
**%Error:**	2.82

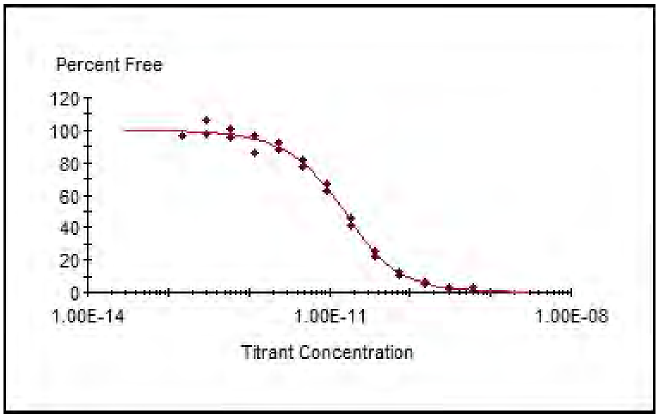

**Kd:**	8.55pM
**95% confidence interval**	
**Kd High:**	14.32pM
**Kd Low:**	4.50pM

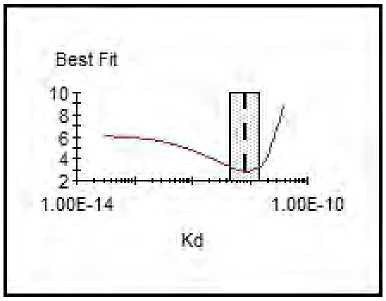

**Active CBP:**	13.14pM
**CBP %Activity:**	164.25
**95% confidence interval**	
**CBP High:**	24.58pM
**%Activity:**	307.29
**CBP Low:**	Less than 47.47fM
**%Activity:**	Less than 0.59

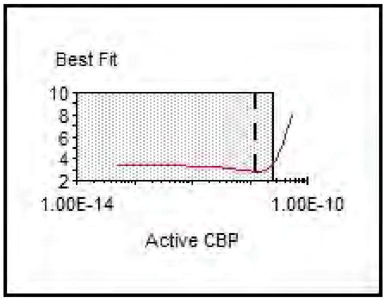

Data Traces (x)

Cycles: 2
Incubation delay (min): 0
Mix Time:

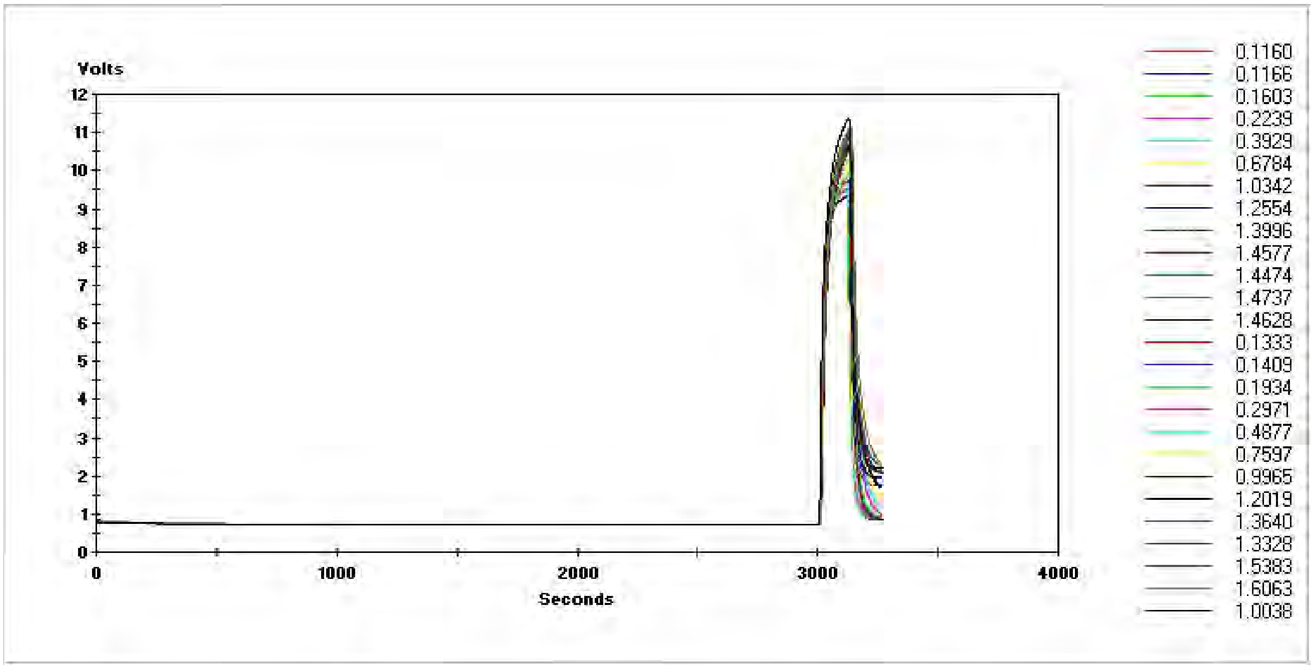

Experiment(x)

**Experiment Name:**	Kd XE06 vs 3E6.1 E1 LCHN mut	**Start Time:**	Wed Jan 25 17:23:06 2012
**Experiment Type:**	Equilibrium	**End Time:**	Thu Jan 26 02:15:49 2012
**Constant Binding Partner (CBP)**		**Buffer:**	pbs/bsa
**Molecular Concentration:**	20.00pM	**Label:**	aSV5-647
**Valency:**	1	**Label Conc:**	0
**Binding Site Concentration:**	20.00pM		

Comments(x)

XE06 beads 1/25/12

BoNT E1 LCHN E730A-E635K-E637K 7/26/11

XE06 081111

aSV5-647

meter: 0.9425

Timing(x)

Bead Handling (Custom Beads)

Draw Source	Time (sec)	Volume (uL)	Rate (mL/min)	Stir
Backflush	30	0	0.0000	
Buffer	20	500	1.5000	✓
Particle Reservoir	28	467	1.0000	✓
Buffer	40	333	0.5000	
Waste	5	25	0.3000	
Buffer	2	10	0.3000	
Buffer	20	0	0.0000	
Buffer	9	150	1.0000	

Sample Timing

Draw Source	Time (sec)	Volume (uL)	Rate (mL/min)	Time Stamp
Sample Set 1-13	720	3000	0.2500	
Buffer	30	125	0.2500	
Inject	120	500	0.2500	
Buffer	30	125	0.2500	
Buffer	120	2000	1.0000	

Analysis(x)

Baseline / Endpoints:
750 to 755 (sec) from beginning
10 to 5 (sec) from end

Ignore	Binding Signal (V)	Concentration
	0.1933	2.00nM
	0.1715	1.00nM
	0.1898	500.00pM
	0.2634	250.00pM
	0.2470	125.00pM
	0.3273	62.50pM
	0.4778	31.25pM
	0.7538	15.63pM
	0.9837	7.81pM
	1.1294	3.90pM
	1.2320	1.95pM
	1.2178	976.56fM
✓	1.3475	488.28fM
	0.1489	2.00nM
	0.1877	1.00nM
	0.1845	500.00pM
	0.2186	250.00pM
	0.2844	125.00pM
	0.3108	62.50pM
	0.4582	31.25pM
	0.7138	15.63pM
	0.9825	7.81pM
	1.0865	3.90pM
	1.1699	1.95pM
	1.2194	976.56fM
	1.2439	488.28fM

**Kd:**	6.71pM
**Active CBP:**	18.15pM
**CBP %Activity:**	90.76
**Ratio:**	2.7037
**Sig 100%:**	1.29
**Drift (%/run):**	0.1721
**NSB:**	0.18
**Drift (mV/run):**	-0.5275
**%Error:**	1.69

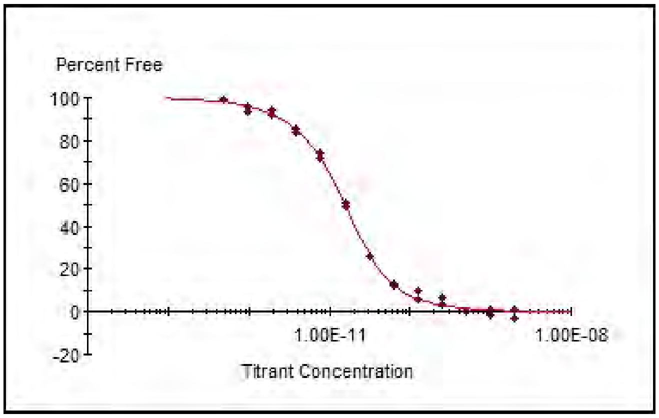

**Kd:**	6.71pM
**95% confidence interval**	
**Kd High:**	9.28pM
**Kd Low:**	4.62pM

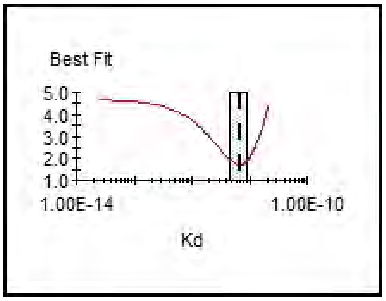

**Active CBP:**	18.15pM
**CBP %Activity:**	90.76
**95% confidence interval**	
**CBP High:**	24.89pM
**%Activity:**	124.46
**CBP Low:**	10.71pM
**%Activity:**	53.56

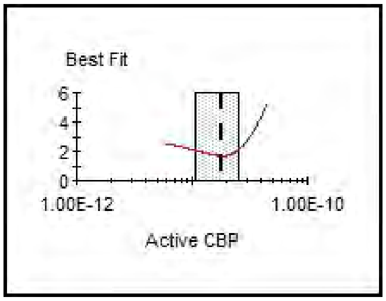

Data Traces (x)

Cycles: 2
Incubation delay (min): 0
Mix Time:

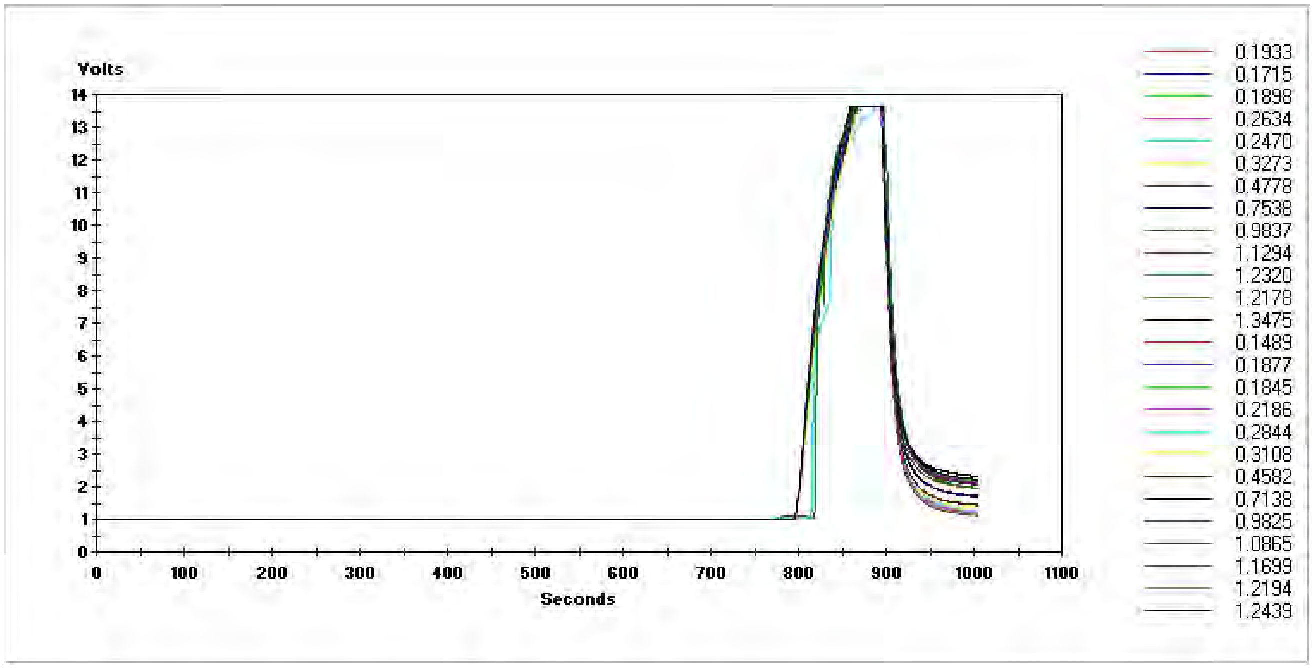

Experiment(x)

**Experiment Name:**	Standard Kd 4E17.1 (beads)-BoNTE3	**Start Time:**	Thu Sep 07 17:13:24 2006
**Experiment Type:**	Equilibrium	**End Time:**	Fri Sep 08 11:59:32 2006
**Constant Binding Partner (CBP)**		**Buffer:**	pbs/bsa
**Molecular Concentration:**	0	**Label:**	3E2-647
**Valency:**	2	**Label Conc:**	0
**Binding Site Concentration:**	0		

Comments(x)

3E2-647

Timing(x)

Bead Handling (Custom Beads)

Draw Source	Time (sec)	Volume (uL)	Rate (mL/min)	Stir
Backflush	30	0	0.0000	
Buffer	20	500	1.5000	✓
Particle Reservoir	25	417	1.0000	✓
Buffer	40	333	0.5000	
Waste	5	25	0.3000	
Buffer	2	10	0.3000	
Buffer	20	0	0.0000	
Buffer	9	150	1.0000	

Sample Timing

Draw Source	Time (sec)	Volume (uL)	Rate (mL/min)	Time Stamp
Sample Set 1-13	720	3000	0.2500	
Buffer	30	125	0.2500	
Inject	120	500	0.2500	
Buffer	30	125	0.2500	
Buffer	120	2000	1.0000	

Analysis(x)

Baseline / Endpoints:
5 to 10 (sec) from beginning
10 to 5 (sec) from end

Ignore	Binding Signal (V)	Concentration
	0.1079	6.00nM
	0.1424	3.00nM
	0.2395	1.50nM
	0.2593	750.00pM
	0.3407	375.00pM
	0.4613	187.50pM
	0.5928	93.75pM
	0.6525	46.88pM
	0.7227	23.44pM
	0.7438	11.72pM
	0.7714	5.86pM
	0.7770	2.93pM
	0.7685	1.46pM
	0.0989	6.00nM
	0.1146	3.00nM
	0.1746	1.50nM
	0.2805	750.00pM
	0.3606	375.00pM
	0.4582	187.50pM
	0.5859	93.75pM
	0.6649	46.88pM
	0.7069	23.44pM
	0.7240	11.72pM
	0.7446	5.86pM
	0.7630	2.93pM
	0.7584	1.46pM

**Kd:**	239.58pM
**Active CBP:**	239.52fM
**CBP %Activity:**	1.#J
**Ratio:**	0.0010
**Sig 100%:**	0.79
**Drift (%/run):**	0.1100
**NSB:**	0.09
**Drift (mV/run):**	997.1010
**%Error:**	2.22

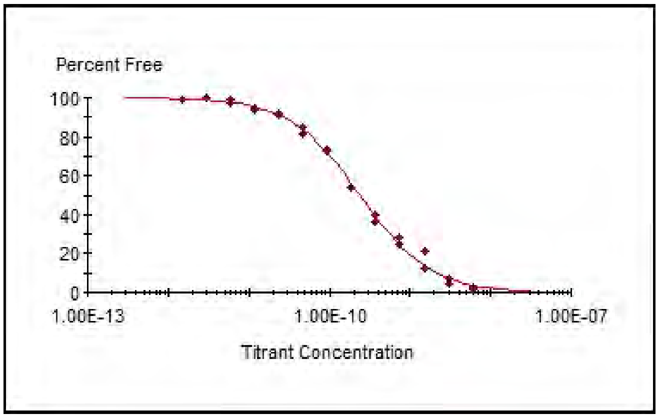

**Kd:**	239.58pM
**95% confidence interval**	
**Kd High:**	281.13pM
**Kd Low:**	181.42pM

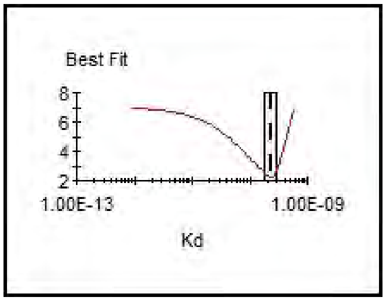

**Active CBP:**	239.52fM
**CBP %Activity:**	1.#J
**95% confidence interval**	
**CBP High:**	Greater than 66.30pM
**%Activity:**	Greater than 1.#J
**CBP Low:**	Less than 865.30aM
**%Activity:**	Less than 1.#J

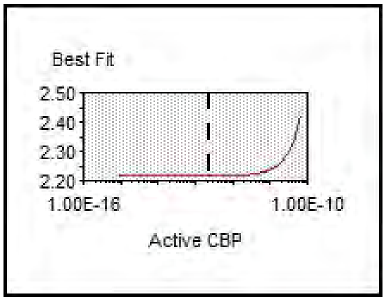

Data Traces(x)

Cycles: 2
Incubation delay (min): 600
Mix Time:

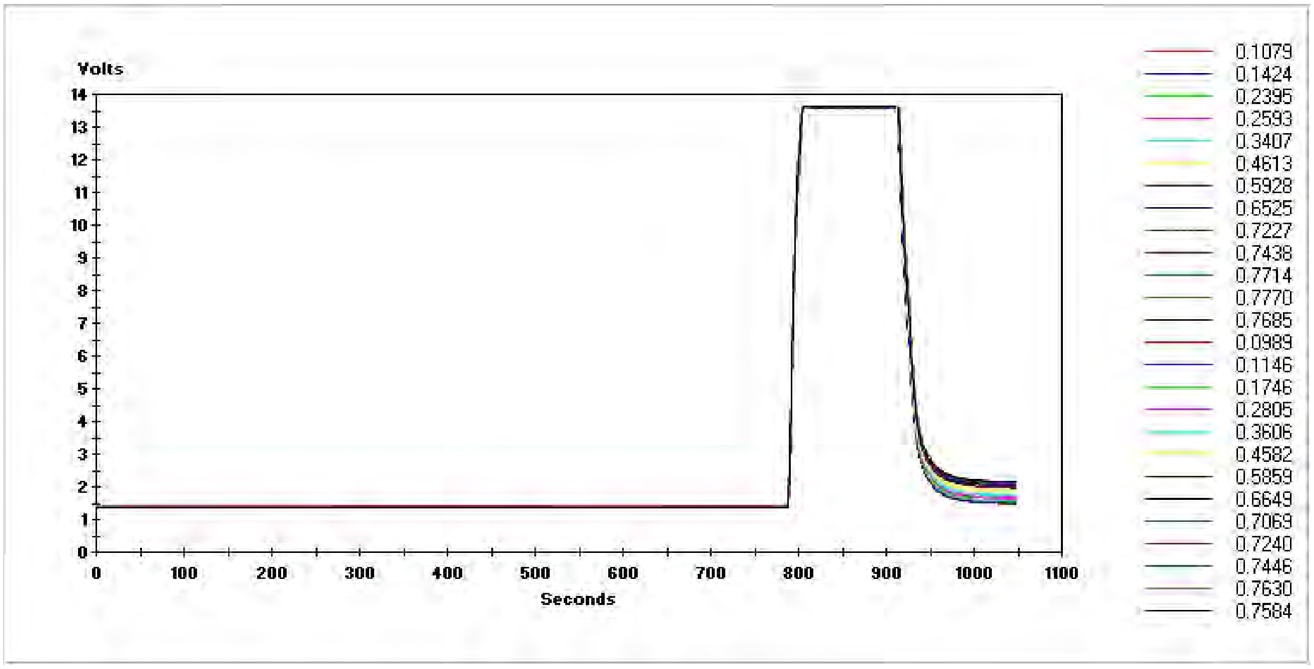

Experiment(x)

**Experiment Name:**	Kd XE17 vs 4E17.1 E1 LCHN mut{3}	**Start Time:**	Thu Jan 26 17:42:52 2012
**Experiment Type:**	Equilibrium	**End Time:**	Fri Jan 27 02:35:36 2012
**Constant Binding Partner (CBP)**		**Buffer:**	pbs/bsa
**Molecular Concentration:**	20.00pM	**Label:**	aSV5-647
**Valency:**	1	**Label Conc:**	0
**Binding Site Concentration:**	20.00pM		

Comments(x)

XE17 beads 1/26/12

BoNT E1 LCHN Q608A-Q609A-E635K-E637K 8/2/11

XE17 120410

aSV5-647

meter: 0.9901

Timing(x)

Bead Handling (Custom Beads)

Draw Source	Time (sec)	Volume (uL)	Rate (mL/min)	Stir
Backflush	30	0	0.0000	
Buffer	20	500	1.5000	✓
Particle Reservoir	28	467	1.0000	✓
Buffer	40	333	0.5000	
Waste	5	25	0.3000	
Buffer	2	10	0.3000	
Buffer	20	0	0.0000	
Buffer	9	150	1.0000	

Sample Timing

Draw Source	Time (sec)	Volume (uL)	Rate (mL/min)	Time Stamp
Sample Set 1-13	720	3000	0.2500	
Buffer	30	125	0.2500	
Inject	120	500	0.2500	
Buffer	30	125	0.2500	
Buffer	120	2000	1.0000	

Analysis(x)

Baseline / Endpoints:
750 to 755 (sec) from beginning
10 to 5 (sec) from end

Ignore	Binding Signal (V)	Concentration
	0.2979	10.00nM
	0.3247	5.00nM
	0.3503	2.50nM
	0.3406	1.25nM
	0.4163	625.00pM
	0.4938	312.50pM
	0.6053	156.25pM
	0.7218	78.13pM
	0.8847	39.06pM
	0.9307	19.53pM
	0.9766	9.77pM
✓	1.1135	4.88pM
	1.0434	2.44pM
	0.2003	10.00nM
	0.2380	5.00nM
	0.2732	2.50nM
	0.2645	1.25nM
✓	0.4307	625.00pM
	0.4275	312.50pM
	0.5387	156.25pM
	0.7254	78.13pM
	0.7542	39.06pM
	0.8916	19.53pM
	0.9759	9.77pM
	0.9890	4.88pM
	1.0080	2.44pM

**Kd:**	115.63pM
**Active CBP:**	108.48fM
**CBP %Activity:**	0.54
**Ratio:**	0.0009
**Sig 100%:**	1.05
**Drift (%/run):**	-0.4910
**NSB:**	0.23
**Drift (mV/run):**	-6.4689
**%Error:**	2.39

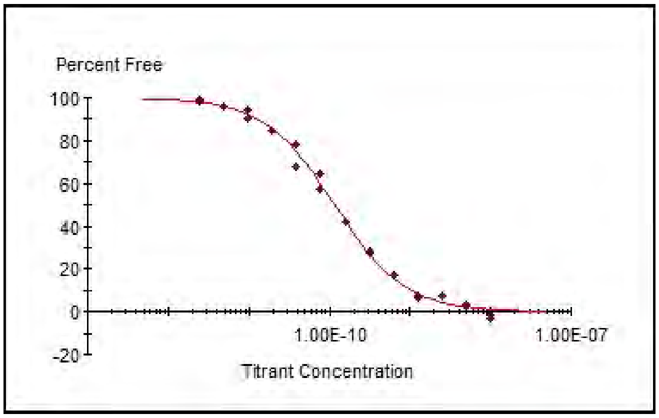

**Kd:**	115.63pM
**95% confidence interval**	
**Kd High:**	136.72pM
**Kd Low:**	88.91pM

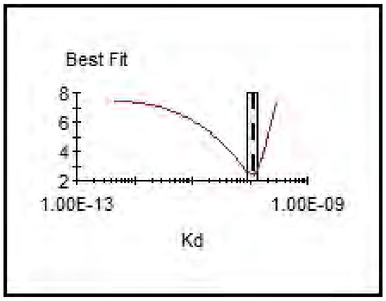

**Active CBP:**	108.48fM
**CBP %Activity:**	0.54
**95% confidence interval**	
**CBP High:**	Greater than 30.03pM
**%Activity:**	Greater than 150.14
**CBP Low:**	Less than 391.91aM
**%Activity:**	Less than 0.00

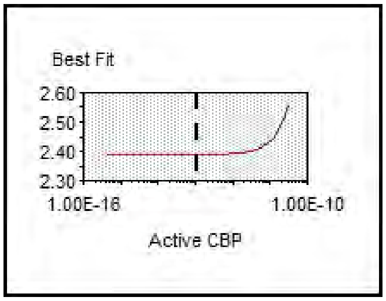

Data Traces(x)

Cycles: 2
Incubation delay (min): 0
Mix Time:

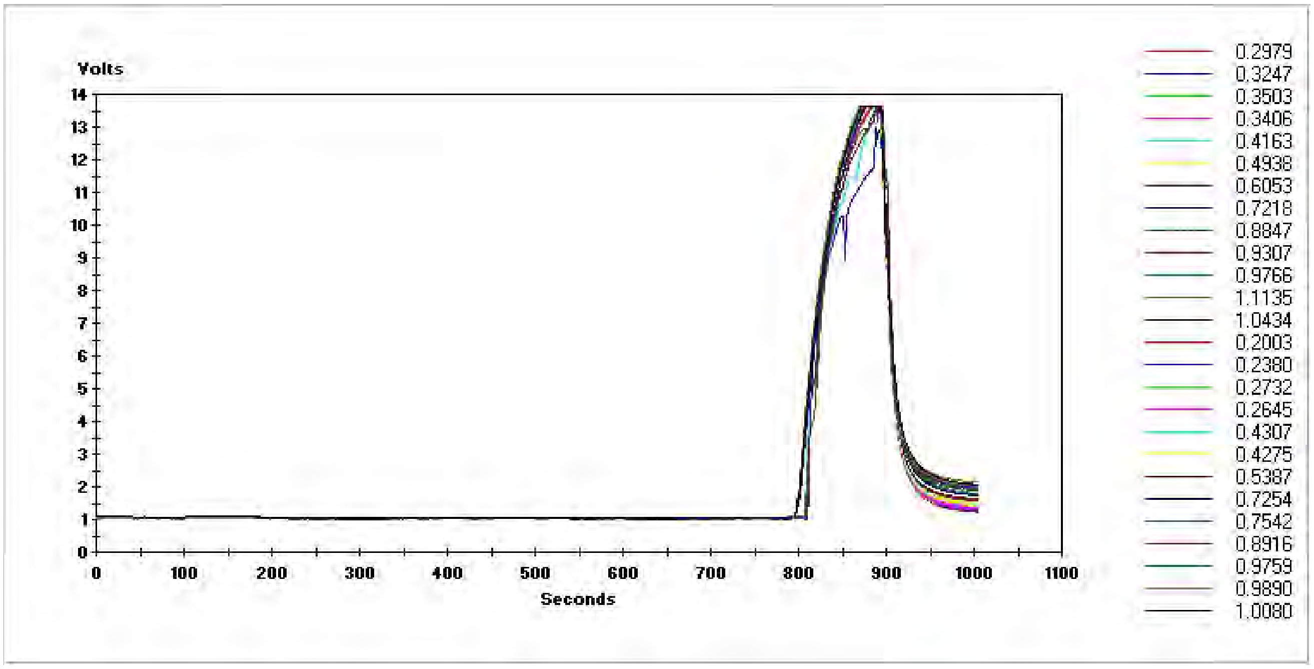

Experiment(x)

**Experiment Name:**	KD TsAb-E(3E2) vs E3 toxin 052915	**Start Time:**	Thu May 7 16:03:47 2015
**Experiment Type:**	Equilibrium	**End Time:**	Sat May 9 13:48:40 2015
**Constant Binding Partner (CBP)**		**Buffer:**	PBS/BSA
**Molecular Concentration:**	15.00pM	**Label:**	3E6.2-647
**Valency:**	1	**Label Conc:**	0
**Binding Site Concentration:**	15.00pM		

Comments(x)

beads: XE02 5/7/15

sample volume: 20 ml

detection: 3E6.2-647

CBP: 15 pM BoNT E3 100208 4/30/15

titrant: TsAb-E 3/18/15 (260 kDa, 0.3 mg/ml, 1.154 uM)

titration: 13 samples: 500 pM - 122 fM (1:2); + E3 only

samples:

1) NSB

2) 100% (E3 only)

3-15) titration of TsAb-E

Timing(x)

Bead Handling (Custom Beads)

Draw Source	Time (sec)	Volume (uL)	Rate (mL/min)	Stir
Backflush	20	0	0.0000	
Buffer	20	500	1.5000	✓
Particle Reservoir 1	18	300	1.0000	✓
Buffer	30	500	1.0000	
Waste	2	8	0.2500	
Buffer	20	0	0.0000	
Buffer	9	150	1.0000	

Sample Timing

Draw Source	Time (sec)	Volume (uL)	Rate (mL/min)	Time Stamp
Sample Set 1,101-114	4800	20000	0.2500	
Buffer	30	125	0.2500	
Standards: Tube 3	120	500	0.2500	
Buffer	30	125	0.2500	
Buffer	90	1500	1.0000	

Analysis (x)

Baseline / Endpoints:

to (sec) from beginning

to (sec) from end

Ignore	Binding Signal (V)	Concentration
✓	0.2718	NSB
✓	0.6831	0
✓	0.2177	210.00pM
	0.2015	105.00pM
	0.2005	52.50pM
	0.2164	26.25pM
	0.3199	13.13pM
	0.4525	6.56pM
	0.5260	3.28pM
	0.5850	1.64pM
	0.5874	820.31fM
	0.6149	410.16fM
	0.6282	205.08fM
	0.6020	102.54fM
	0.6052	51.27fM
✓	0.1826	25.63fM
	0.6114	12.82fM
✓	0.1983	210.00pM
	0.1079	105.00pM
	0.1004	52.50pM
	0.0962	26.25pM
	0.1371	13.13pM
	0.2978	6.56pM
	0.3875	3.28pM
	0.4461	1.64pM
	0.4251	820.31fM
✓	0.4228	410.16fM
	0.4469	205.08fM
✓	0.4004	102.54fM
	0.4490	51.27fM

**Kd:**	1.18pM
**Active CBP:**	11.63pM
**CBP %Activity:**	77.54
**Ratio:**	9.8831
**Sig 100%:**	0.60
**Drift (%/run):**	0.5793
**NSB:**	0.11
**Drift (mV/run):**	-7.9989
**%Error:**	2.85

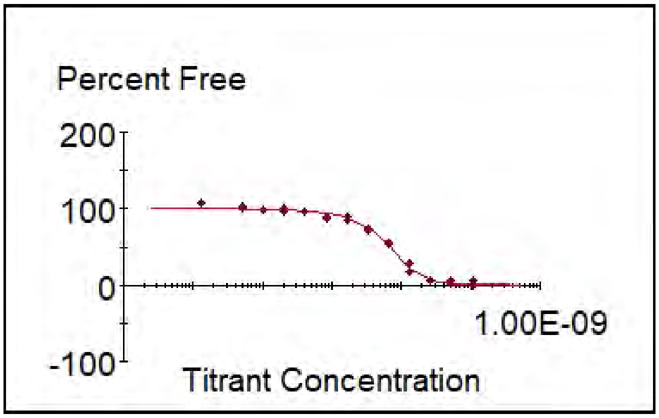

**Kd:**	1.18pM
**95% confidence interval**	
**Kd High:**	2.99pM
**Kd Low:**	256.40fM

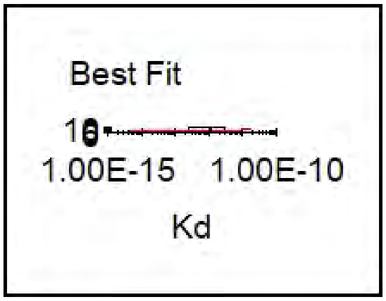

**Active CBP:**	11.63pM
**CBP %Activity:**	77.54
**95% confidence interval**	
**CBP High:**	15.67pM
**%Activity:**	104.49
**CBP Low:**	6.45pM
**%Activity:**	43.02

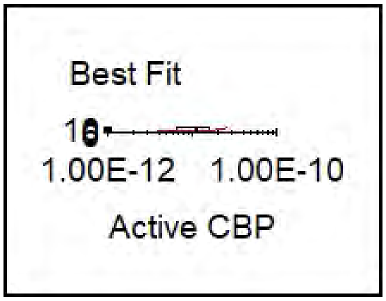

Data Traces(x)

Cycles: 2
Incubation delay (min): 0
Mix Time:

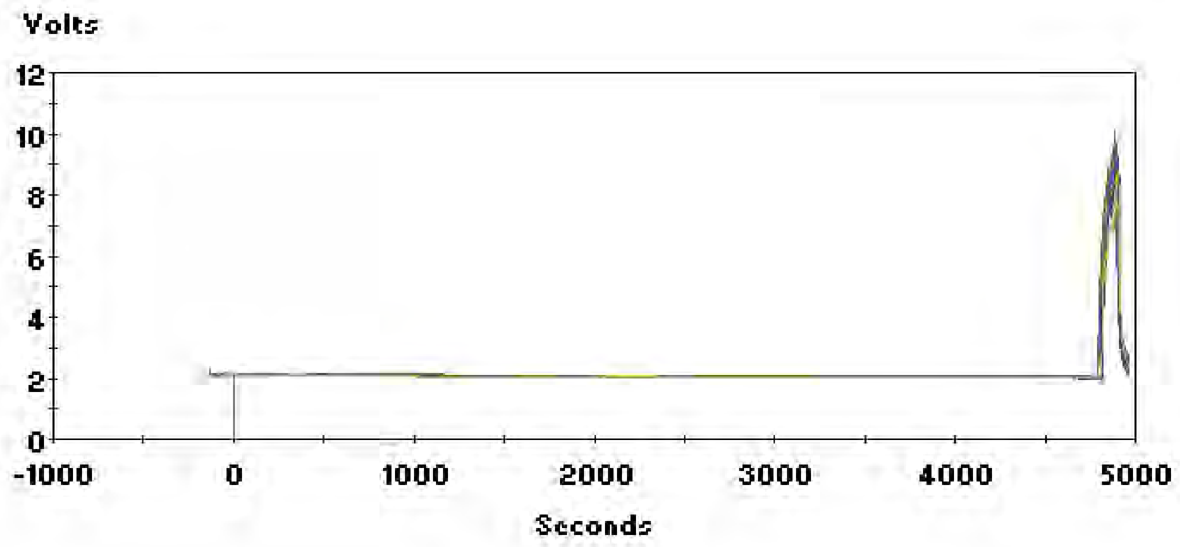

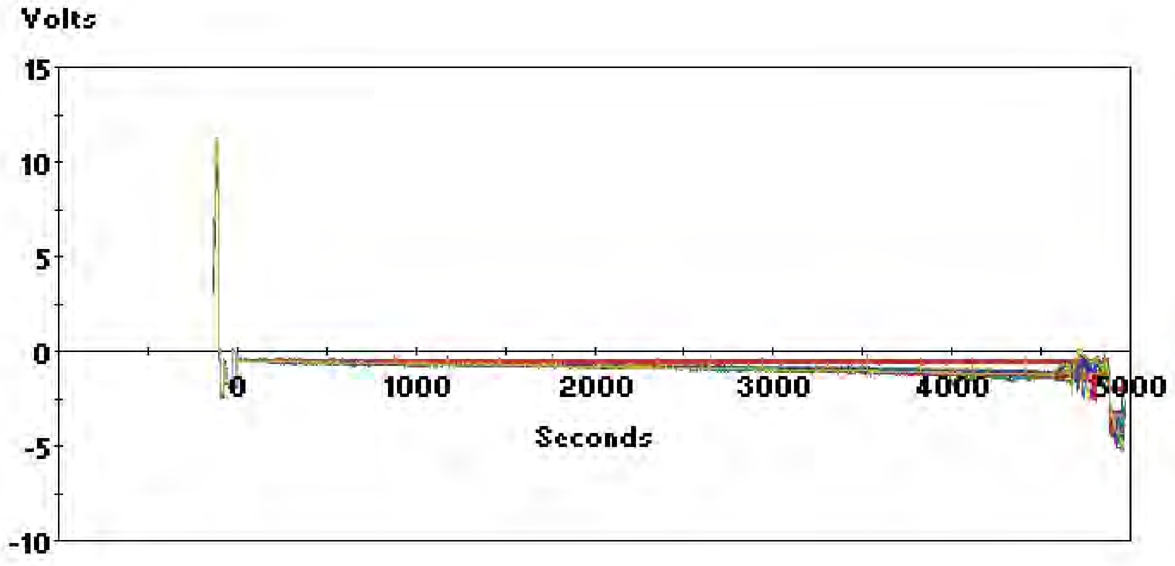

Experiment(x)

**Experiment Name:**	KD TsAb-E(3E2) vs LCHN-E02	**Start Time:**	Wed Aug 12 17:31:41 2015
**Experiment Type:**	Equilibrium	**End Time:**	Thu Aug 13 06:09:16 2015
**Constant Binding Partner (CBP)**		**Buffer:**	PBS/BSA
**Molecular Concentration:**	7.00pM	**Label:**	aSV5-647
**Valency:**	1	**Label Conc:**	0
**Binding Site Concentration:**	7.00pM		

Comments(x)

beads: XE02 8/12/15

sample volume: 3 ml

detection: aSV5-647

CBP: 7 pM BoNT LCHN-E02 16907945-132 4/27/15

titrant: TsAb-E 3/25/15 (thawed 8/10/15; 260 kDa, 0.85 mg/ml, 3269 nM)

titration: 15 samples: 30 nM - 1.83 pM (1:2); + CBP only

samples:

1) NSB

2) 100% (CBP only)

3-15) titration

Timing(x)

Bead Handling (Custom Beads)

Draw Source	Time (sec)	Volume (uL)	Rate (mL/min)	Stir
Backflush	20	0	0.0000	
Buffer	20	500	1.5000	✓
Particle Reservoir 1	18	300	1.0000	✓
Buffer	30	500	1.0000	
Waste	2	8	0.2500	
Buffer	20	0	0.0000	
Buffer	9	150	1.0000	

Sample Timing

Draw Source	Time (sec)	Volume (uL)	Rate (mL/min)	Time Stamp
Sample Set 1,201-216	720	3000	0.2500	
Buffer	30	125	0.2500	
Standards: Tube 3	120	500	0.2500	
Buffer	30	125	0.2500	
Buffer	90	1500	1.0000	

Analysis(x)

Baseline / Endpoints:

to (sec) from beginning

to (sec) from end

Ignore	Binding Signal (V)	Concentration
✓	0.3806	0
	2.0551	0
	0.4809	30.00nM
	0.5155	15.00nM
	0.5249	7.50nM
	0.6379	3.75nM
	0.7559	1.88nM
	1.0341	937.50pM
	1.3404	468.75pM
	1.6381	234.38pM
	1.7883	117.19pM
	1.8674	58.59pM
	1.9283	29.30pM
	1.9591	14.65pM
	1.9856	7.32pM
	1.9908	3.66pM
	2.0045	1.83pM
✓	0.4031	0
	1.9993	0
	0.4575	30.00nM
	0.4846	15.00nM
	0.4869	7.50nM
	0.5973	3.75nM
	0.7449	1.88nM
	1.0508	937.50pM
	1.3275	468.75pM
	1.5988	234.38pM
	1.7623	117.19pM
	1.8679	58.59pM
	1.9004	29.30pM
	1.9742	14.65pM
	1.9791	7.32pM
	1.9606	3.66pM
	1.9839	1.83pM

**Kd:**	7.48pM
**CBP:**	7.00pM
**Ratio:**	0.9360
**Titrant % Activity:**	1.7955
**Sig 100%:**	1.99
**NSB:**	0.44
**%Error:**	1.31

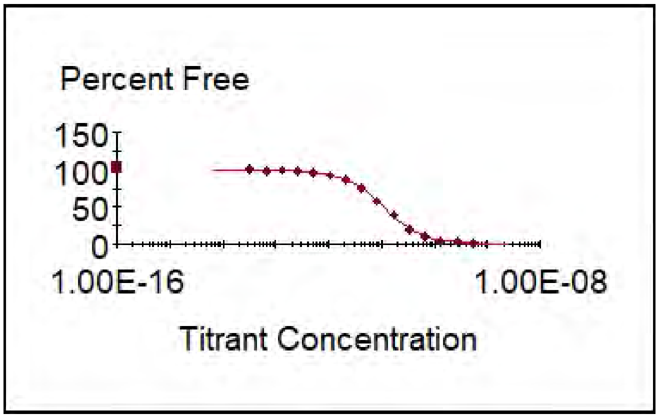

Kd: 7.48pM

95% confidence interval

Kd High: 21.60pM

Kd Low: 3.82pM

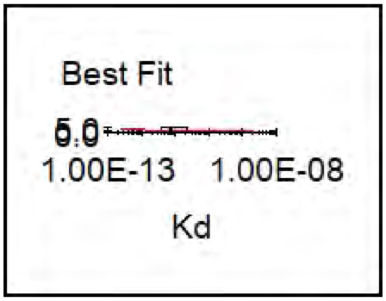

Titrant %Activity: 1.7955

95% confidence interval

Titrant %Activity High: 6.6040

Titrant %Activity Low: 1.0900

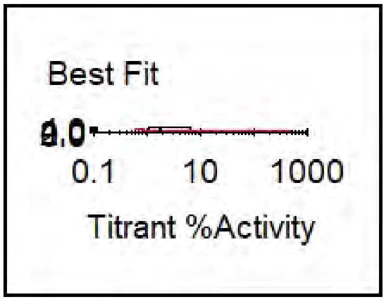

Data Traces(x)

Cycles: 2
Incubation delay (min): 0
Mix Time:

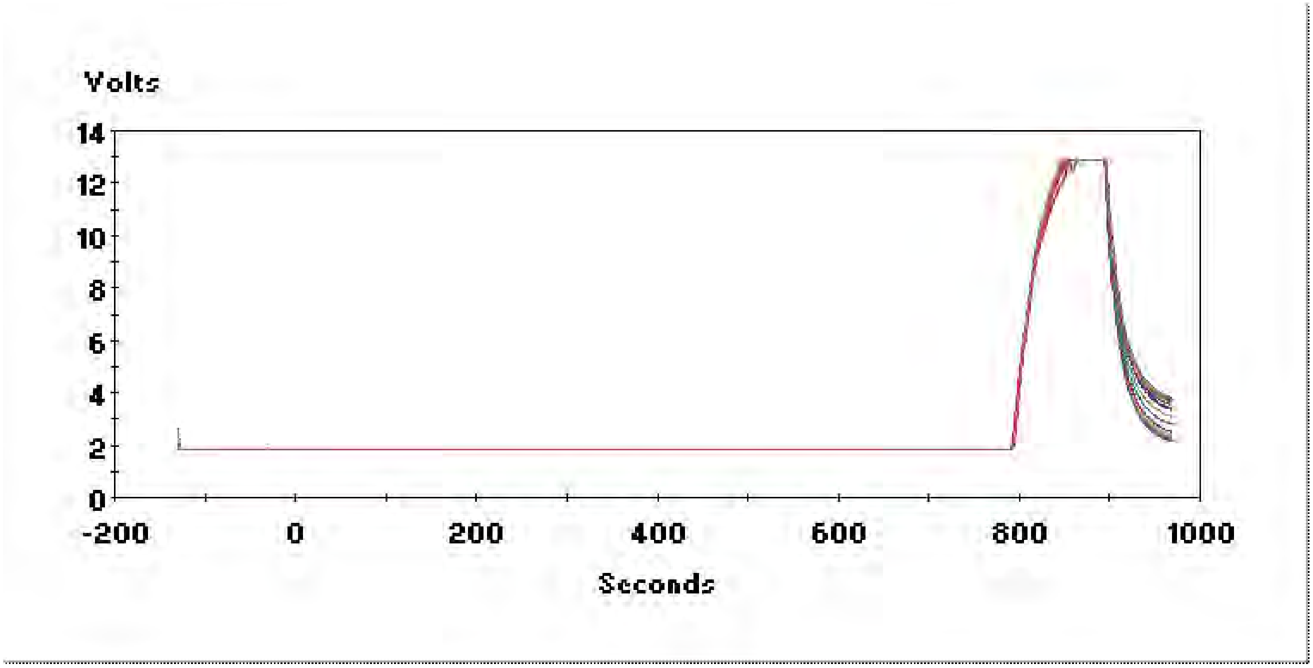

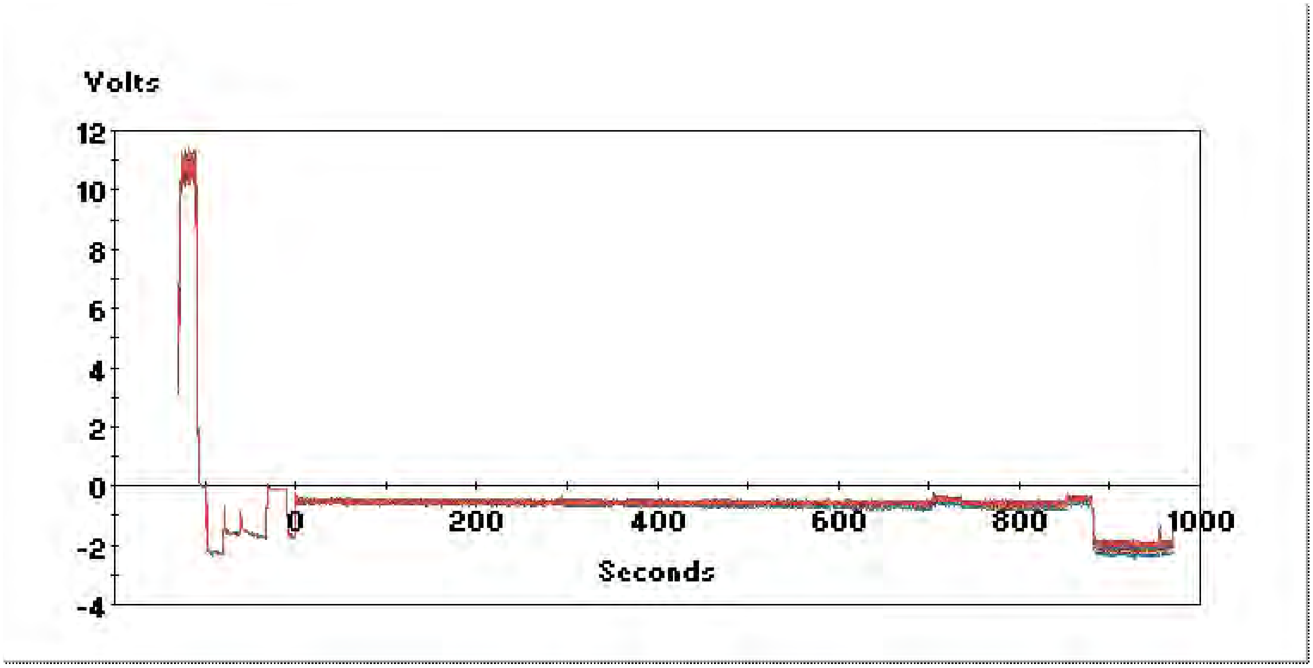

Experiment(x)

**Experiment Name:**	KD TsAb-E(3E6.2) vs E3 042015	**Start Time:**	Sat Apr 18 06:13:31 2015
**Experiment Type:**	Equilibrium	**End Time:**	Sun Apr 19 07:36:40 2015
**Constant Binding Partner (CBP)**		**Buffer:**	PBS/BSA
**Molecular Concentration:**	10.00pM	**Label:**	XE02-647
**Valency:**	1	**Label Conc:**	0
**Binding Site Concentration:**	10.00pM		

Comments(x)

beads:NXE06 4/17/15

sample volume: 10 ml

detection: XE02-647

CBP: 10 pM BoNT E3 100208 4/1/15

titrant: TsAb-E 3/18/15 (260 kDa, 0.3 mg/ml, 1.154 uM)

titration: 13 samples: 200 pM - 48 fM (1:2); + B1 only

samples:

1) NSB

2) 100% (E3 only)

3-15) titration of TsAb-E

Timing(x)

Bead Handling (Custom Beads)

Draw Source	Time (sec)	Volume (uL)	Rate (mL/min)	Stir
Backflush	20	0	0.0000	
Buffer	20	500	1.5000	✓
Particle Reservoir 2	20	333	1.0000	✓
Buffer	30	500	1.0000	
Waste	2	8	0.2500	
Buffer	20	0	0.0000	
Buffer	9	150	1.0000	

Sample Timing

Draw Source	Time (sec)	Volume (uL)	Rate (mL/min)	Time Stamp
Sample Set 1,201-214	2400	10000	0.2500	
Buffer	30	125	0.2500	
Standards: Tube 5	120	500	0.2500	
Buffer	30	125	0.2500	
Buffer	90	1500	1.0000	

Analysis(x)

Baseline / Endpoints:
to (sec) from beginning
to (sec) from end

Ignore	Binding Signal (V)	Concentration
✓	0.1824	0
✓	0.8090	0
	0.1676	200.00pM
	0.1827	100.00pM
	0.1843	50.00pM
	0.2628	25.00pM
	0.4548	12.50pM
	0.6264	6.25pM
	0.7023	3.13pM
	0.7365	1.56pM
	0.7598	781.25fM
	0.7819	390.63fM
	0.7782	195.31fM
	0.7824	97.66fM
	0.7611	48.83fM
✓	0.1735	0
✓	0.7652	0
	0.1794	200.00pM
	0.1746	100.00pM
	0.1703	50.00pM
	0.2533	25.00pM
	0.4253	12.50pM
	0.5859	6.25pM
	0.6580	3.13pM
	0.7120	1.56pM
	0.7154	781.25fM
	0.7478	390.63fM
	0.7457	195.31fM
	0.7444	97.66fM
	0.7441	48.83fM

**Kd:**	851.20fM
**CBP:**	10.00pM
**Ratio:**	11.7481
**Titrant % Activity:**	50.2100
**Sig 100%:**	0.78
**Drift (%/run):**	0.3103
**NSB:**	0.14
**Drift (mV/run):**	-0.4388
**TR NSB:**	2.30e+08
**%Error:**	1.13

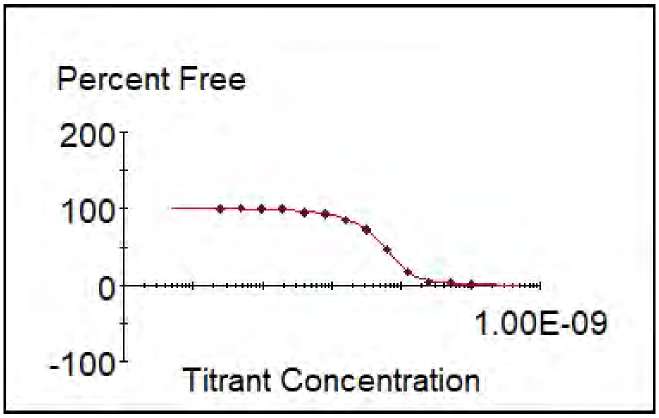

**Kd:**	851.20fM
**95% confidence interval**	
**Kd High:**	1.58pM
**Kd Low:**	439.64fM

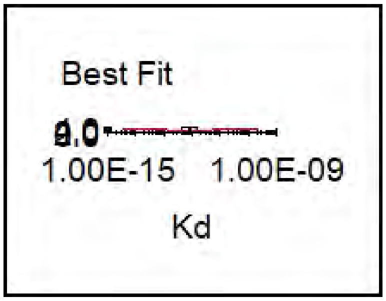

**Titrant %Activity:**	50.2100
**95% confidence interval**	
**Titrant %Activity High:**	57.7272
**Titrant %Activity Low:**	45.1309

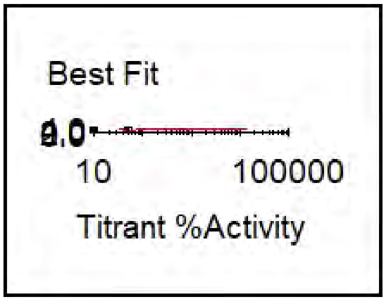

Data Traces(x)

Cycles: 2
Incubation delay (min): 0
Mix Time:

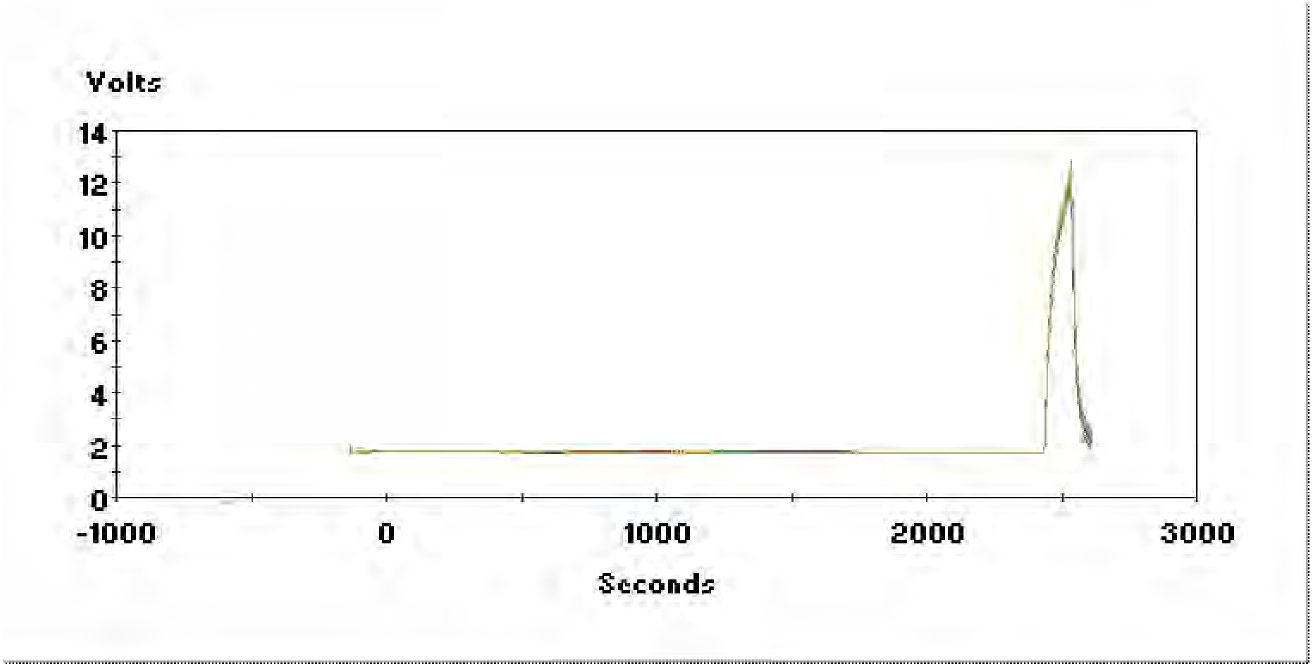

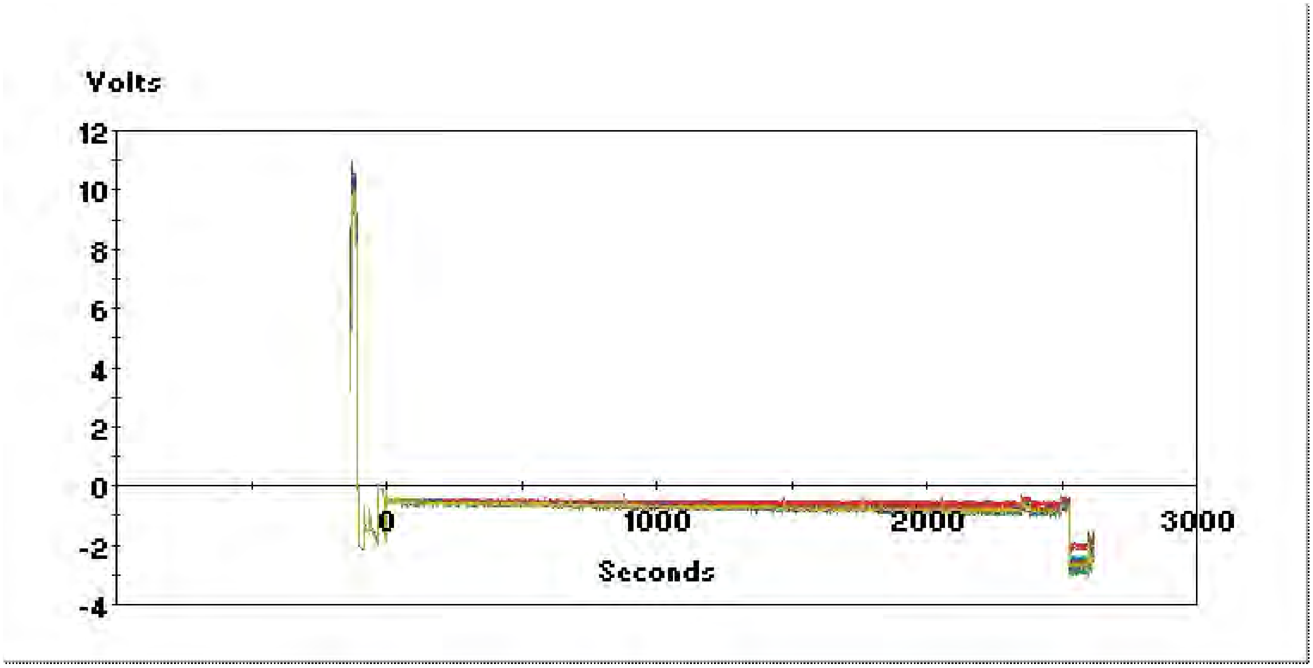

Experiment(x)

**Experiment Name:**	KD TsAb-E(3E6.2) vs LCHN-E06	**Start Time:**	Thu Aug 13 16:56:10 2015
**Experiment Type:**	Equilibrium	**End Time:**	Fri Aug 14 05:34:57 2015
**Constant Binding Partner (CBP)**		**Buffer:**	PBS/BSA
**Molecular Concentration:**	8.00pM	**Label:**	aSV5-647
**Valency:**	1	**Label Conc:**	0
**Binding Site Concentration:**	8.00pM		

Comments(x)

beads: XE06 8/13/15

sample volume: 3 ml

detection: aSV5-647

CBP: 8 pM BoNT LCHN-E06 16907945-143 4/27/15

titrant: TsAb-E 3/25/15 (thawed 8/10/15; 260 kDa, 0.85 mg/ml, 3269 nM)

titration: 15 samples: 25 nM - 1.53 pM (1:2); + CBP only

samples:

1) NSB

2) 100% (CBP only)

3-15) titration

Timing(x)

Bead Handling (Custom Beads)

Draw Source	Time (sec)	Volume (uL)	Rate (mL/min)	Stir
Backflush	20	0	0.0000	
Buffer	20	500	1.5000	✓
Particle Reservoir 1	20	333	1.0000	✓
Buffer	30	500	1.0000	
Waste	2	8	0.2500	
Buffer	20	0	0.0000	
Buffer	9	150	1.0000	

Sample Timing

Draw Source	Time (sec)	Volume (uL)	Rate (mL/min)	Time Stamp
Sample Set 1,201-216	720	3000	0.2500	
Buffer	30	125	0.2500	
Standards: Tube 3	120	500	0.2500	
Buffer	30	125	0.2500	
Buffer	90	1500	1.0000	

Analysis(x)

Baseline / Endpoints:

to (sec) from beginning

to (sec) from end

Ignore	Binding Signal (V)	Concentration
✓	0.4067	0
✓	1.7129	0
	0.4011	25.00nM
	0.4206	12.50nM
	0.4453	6.25nM
	0.4856	3.13nM
	0.5000	1.56nM
	0.6221	781.25pM
	0.7315	390.63pM
	0.9328	195.31pM
	1.1869	97.66pM
	1.4162	48.83pM
	1.5615	24.41pM
	1.6499	12.21pM
	1.6774	6.10pM
	1.6936	3.05pM
	1.6927	1.53pM
✓	0.4394	0
✓	1.7085	0
	0.4269	25.00nM
	0.4244	12.50nM
	0.4604	6.25nM
	0.4719	3.13nM
	0.5056	1.56nM
	0.5842	781.25pM
	0.6925	390.63pM
	0.9082	195.31pM
	1.1423	97.66pM
	1.3869	48.83pM
	1.5285	24.41pM
	1.6171	12.21pM
	1.6314	6.10pM
	1.6799	3.05pM
	1.6843	1.53pM

**Kd:**	13.87pM
**CBP:**	8.00pM
**Ratio:**	0.5769
**Titrant % Activity:**	13.7096
**Sig 100%:**	1.73
**Drift (%/run):**	0.1459
**NSB:**	0.42
**Drift (mV/run):**	-0.1734
**TR NSB:**	2.67e-07
**%Error:**	1.03

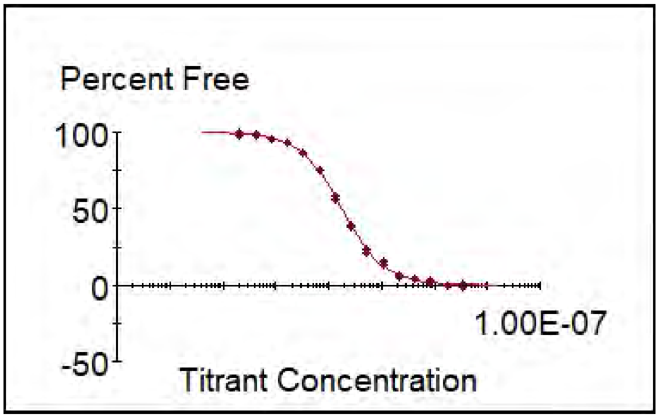

**Kd:**	13.87pM
**95% confidence interval**	
**Kd High:**	42.39pM
**Kd Low:**	7.52pM

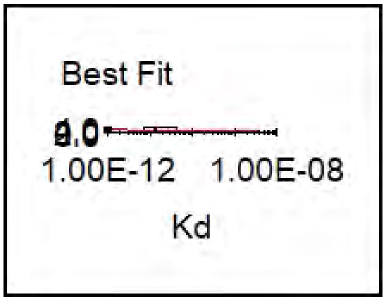

**Titrant %Activity:**	13.7096
**95% confidence interval**	
**Titrant %Activity High:**	67.7883
**Titrant %Activity Low:**	7.8933

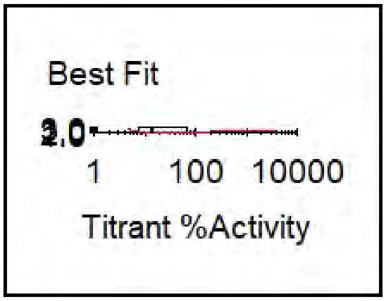

Data Traces(x)

Cycles: 2
Incubation delay (min): 0
Mix Time:

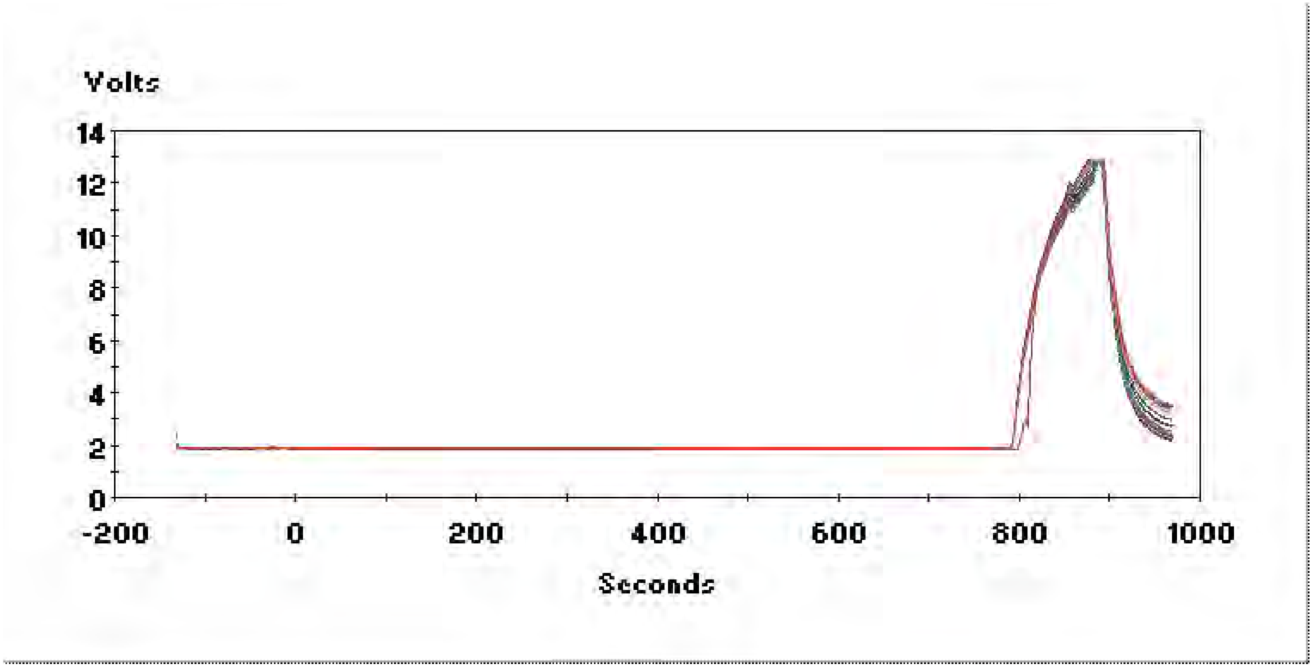

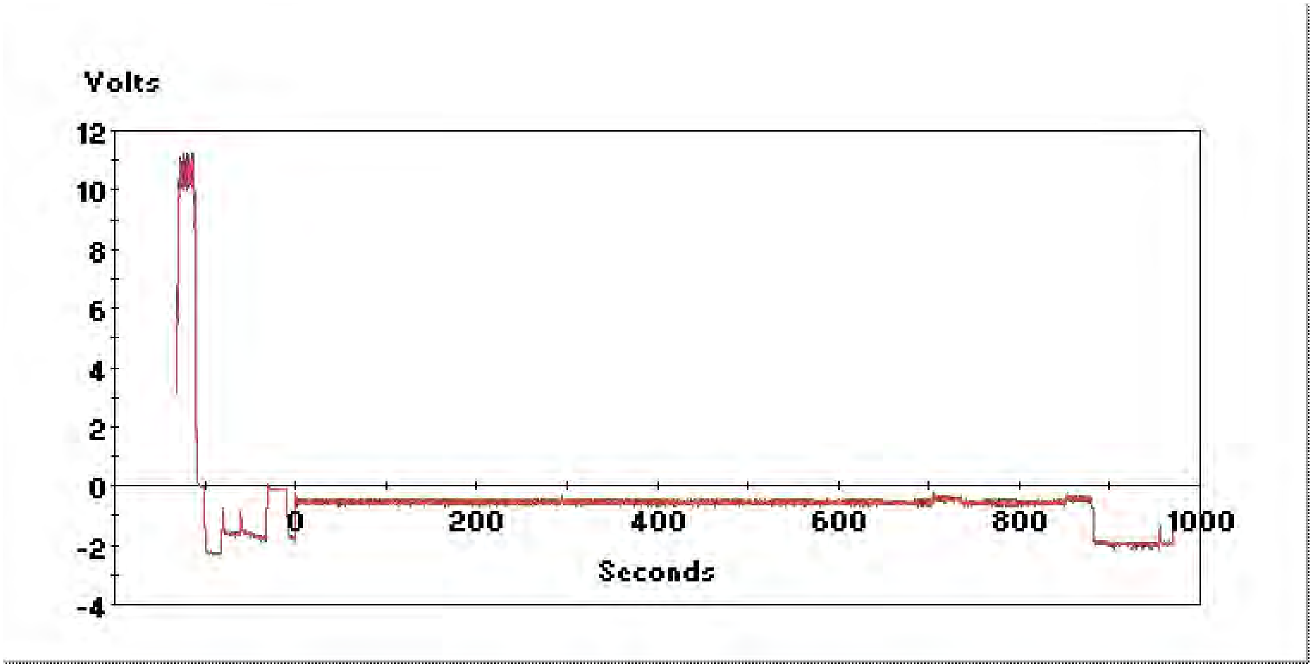

Experiment(x)

**Experiment Name:**	KD TsAb-E(4E17.1) vs E3 042115	**Start Time:**	Mon Apr 20 14:56:30 2015
**Experiment Type:**	Equilibrium	**End Time:**	Tue Apr 21 16:19:15 2015
**Constant Binding Partner (CBP)**		**Buffer:**	PBS/BSA
**Molecular Concentration:**	20.00pM	**Label:**	XE02-647
**Valency:**	1	**Label Conc:**	0
**Binding Site Concentration:**	20.00pM		

Comments(x)

beads: XE17 3/20/15

sample volume: 10 ml

detection: XE02-647

CBP: 20 pM BoNT E3 100208 4/1/15

titrant: TsAb-E 3/18/15 (260 kDa, 0.3 mg/ml, 1.154 uM)

titration: 13 samples: 200 pM - 48 fM (1:2); + E3 only

samples:

1) NSB

2) 100% (E3 only)

3-15) titration of TsAb-E

Timing(x)

Bead Handling (Custom Beads)

Draw Source	Time (sec)	Volume (uL)	Rate (mL/min)	Stir
Backflush	20	0	0.0000	
Buffer	20	500	1.5000	✓
Particle Reservoir 1	20	333	1.0000	✓
Buffer	30	500	1.0000	
Waste	2	8	0.2500	
Buffer	20	0	0.0000	
Buffer	9	150	1.0000	

Sample Timing

Draw Source	Time (sec)	Volume (uL)	Rate (mL/min)	Time Stamp
Sample Set 1,101-114	2400	10000	0.2500	
Buffer	30	125	0.2500	
Standards: Tube 3	120	500	0.2500	
Buffer	30	125	0.2500	
Buffer	90	1500	1.0000	

Analysis(x)

Baseline / Endpoints:

to (sec) from beginning

to (sec) from end

Ignore	Binding Signal (V)	Concentration
✓	0.1686	NSB
	0.5454	0
	0.1253	200.00pM
	0.1455	100.00pM
	0.1616	50.00pM
	0.3010	25.00pM
	0.4305	12.50pM
	0.4768	6.25pM
	0.5127	3.13pM
	0.5406	1.56pM
✓	0.5149	781.25fM
	0.5290	390.63fM
	0.5230	195.31fM
	0.5359	97.66fM
	0.5316	48.83fM
✓	0.1435	NSB
	0.5173	0
	0.1343	200.00pM
	0.1339	100.00pM
	0.1468	50.00pM
	0.2805	25.00pM
	0.3822	12.50pM
	0.4518	6.25pM
	0.4865	3.13pM
	0.4850	1.56pM
	0.4883	781.25fM
	0.5105	390.63fM
	0.4974	195.31fM
	0.5032	97.66fM
	0.4864	48.83fM

**Kd:**	427.61fM
**CBP:**	20.00pM
**Ratio:**	46.7720
**Titrant % Activity:**	50.3200
**Sig 100%:**	0.53
**Drift (%/run):**	0.3994
**NSB:**	0.13
**Drift (mV/run):**	-0.6093
**%Error:**	1.79

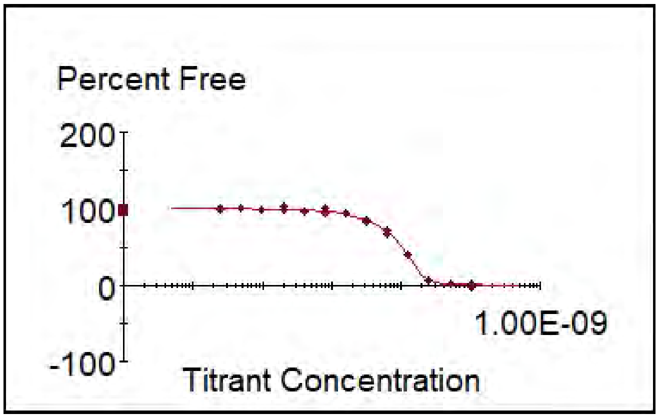

**Kd:**	427.61fM
**95% confidence interval**	
**Kd High:**	1.21pM
**Kd Low:**	42.01fM

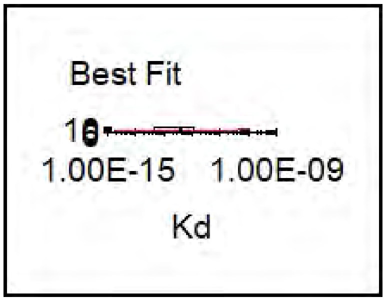

**Titrant %Activity:**	50.3200
**95% confidence interval**	
**Titrant %Activity High:**	57.2204
**Titrant %Activity Low:**	45.4360

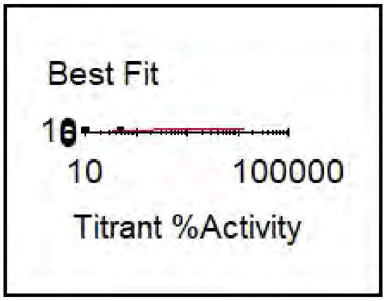

Data Traces(x)

Cycles: 2
Incubation delay (min): 0
Mix Time:

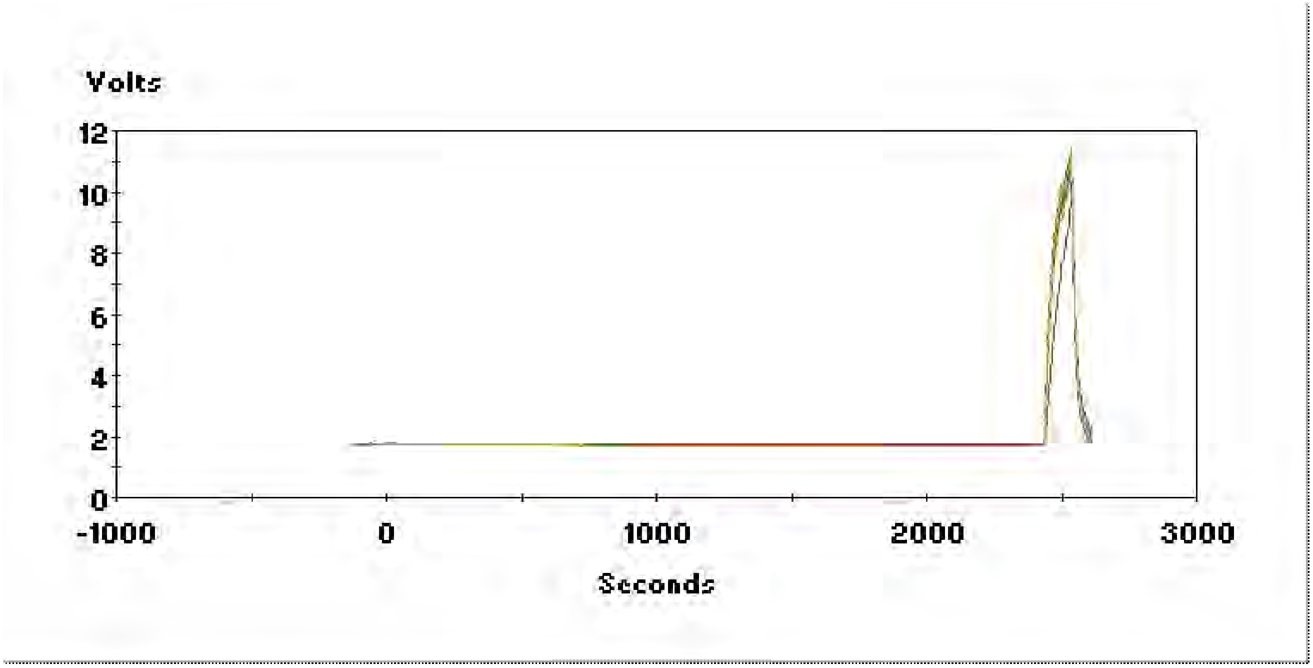

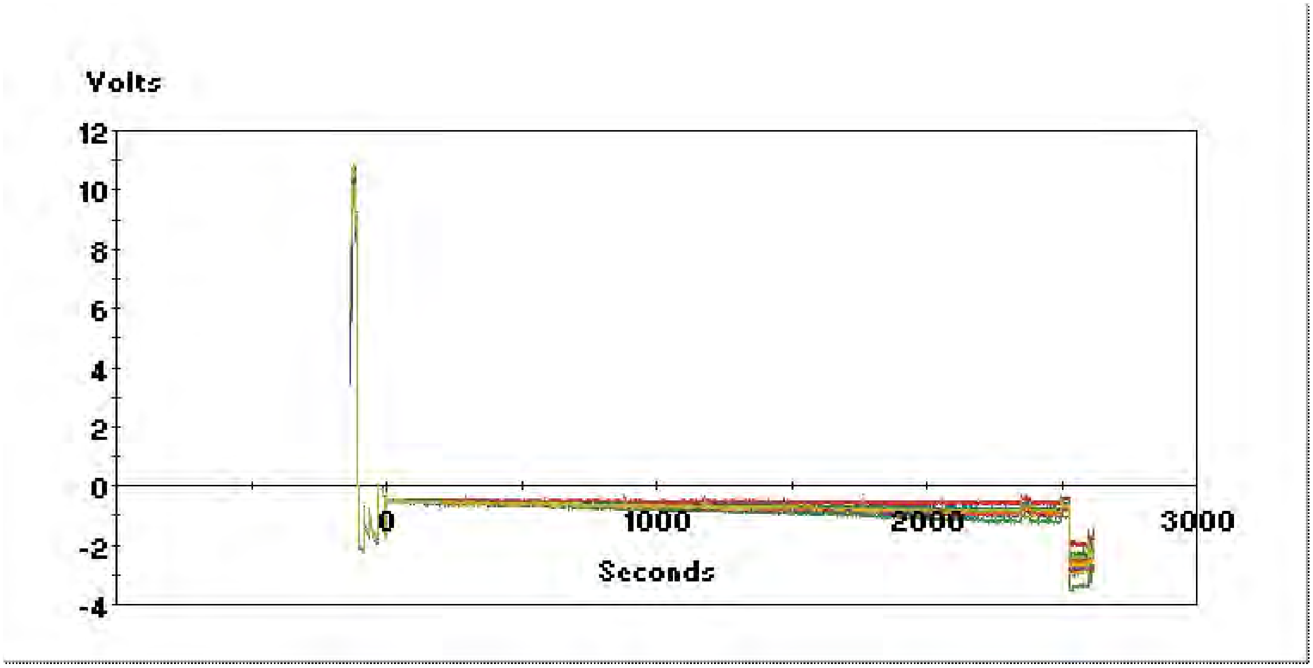

Experiment(x)

**Experiment Name:**	KD TsAb-E(4E17.1) vs LCHN-E17	**Start Time:**	Tue Aug 11 18:14:52 2015
**Experiment Type:**	Equilibrium	**End Time:**	Wed Aug 12 06:52:23 2015
**Constant Binding Partner (CBP)**		**Buffer:**	PBS/BSA
**Molecular Concentration:**	15.00pM	**Label:**	aSV5-647
**Valency:**	1	**Label Conc:**	0
**Binding Site Concentration:**	15.00pM		

Comments(x)

beads: XE17 8/11/15

sample volume: 3 ml

detection: aSV5-647

CBP: 15 pM BoNT LCHN-E17 23421972-9 4/27/15

titrant: TsAb-E 3/25/15 (thawed 8/10/15; 260 kDa, 0.85 mg/ml, 3269 nM)

titration: 15 samples: 80 nM - 4.88 pM (1:2); + CBP only

samples:

1) NSB

2) 100% (CBP only)

3-15) titration

Timing(x)

Bead Handling (Custom Beads)

Draw Source	Time (sec)	Volume (uL)	Rate (mL/min)	Stir
Backflush	20	0	0.0000	
Buffer	20	500	1.5000	✓
Particle Reservoir 1	18	300	1.0000	✓
Buffer	30	500	1.0000	
Waste	2	8	0.2500	
Buffer	20	0	0.0000	
Buffer	9	150	1.0000	

Sample Timing

Draw Source	Time (sec)	Volume (uL)	Rate (mL/min)	Time Stamp
Sample Set 1,201-216	720	3000	0.2500	
Buffer	30	125	0.2500	
Standards: Tube 3	120	500	0.2500	
Buffer	30	125	0.2500	
Buffer	90	1500	1.0000	

Analysis(x)

Baseline / Endpoints:

to (sec) from beginning

to (sec) from end

Ignore	Binding Signal (V)	Concentration
✓	0.5143	0
	1.8423	0
	0.5631	80.00nM
	0.5580	40.00nM
	0.5524	20.00nM
	0.5596	10.00nM
	0.5775	5.00nM
	0.6118	2.50nM
	0.6983	1.25nM
	0.8744	625.00pM
	1.0961	312.50pM
	1.3454	156.25pM
	1.4968	78.13pM
	1.6005	39.06pM
	1.6372	19.53pM
	1.6653	9.77pM
	1.6766	4.88pM
✓	0.3960	0
	1.6891	0
	0.4630	80.00nM
	0.4664	40.00nM
	0.4768	20.00nM
	0.4985	10.00nM
	0.4970	5.00nM
	0.5663	2.50nM
	0.6520	1.25nM
	0.8167	625.00pM
	1.0279	312.50pM
	1.2688	156.25pM
	1.4456	78.13pM
	1.5496	39.06pM
	1.5981	19.53pM
	1.6332	9.77pM
	1.6333	4.88pM

**Kd:**	18.15pM
**CBP:**	15.00pM
**Ratio:**	0.8263
**Titrant % Activity:**	8.7656
**Sig 100%:**	1.72
**Drift (%/run):**	0.0110
**NSB:**	0.48
**Drift (mV/run):**	-4.1486
**TR NSB:**	2.16e-07
**%Error:**	1.30

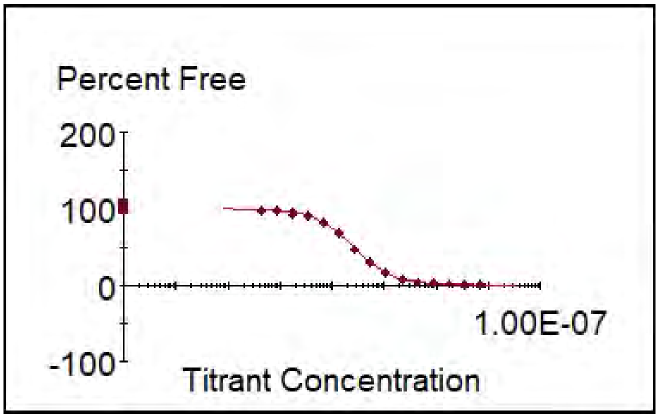

**Kd:**	18.15pM
**95% confidence interval**	
**Kd High:**	51.22pM
**Kd Low:**	9.53pM

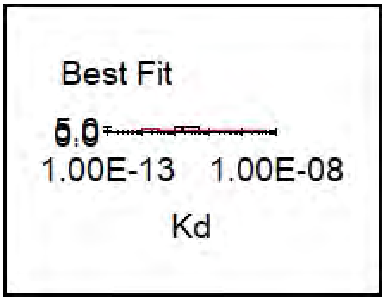

**Titrant %Activity:**	8.7656
**95% confidence interval**	
**Titrant %Activity High:**	51.3052
**Titrant %Activity Low:**	5.1859

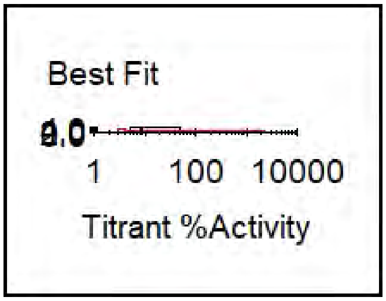

Data Traces(x)

Cycles: 2
Incubation delay (min): 0
Mix Time:

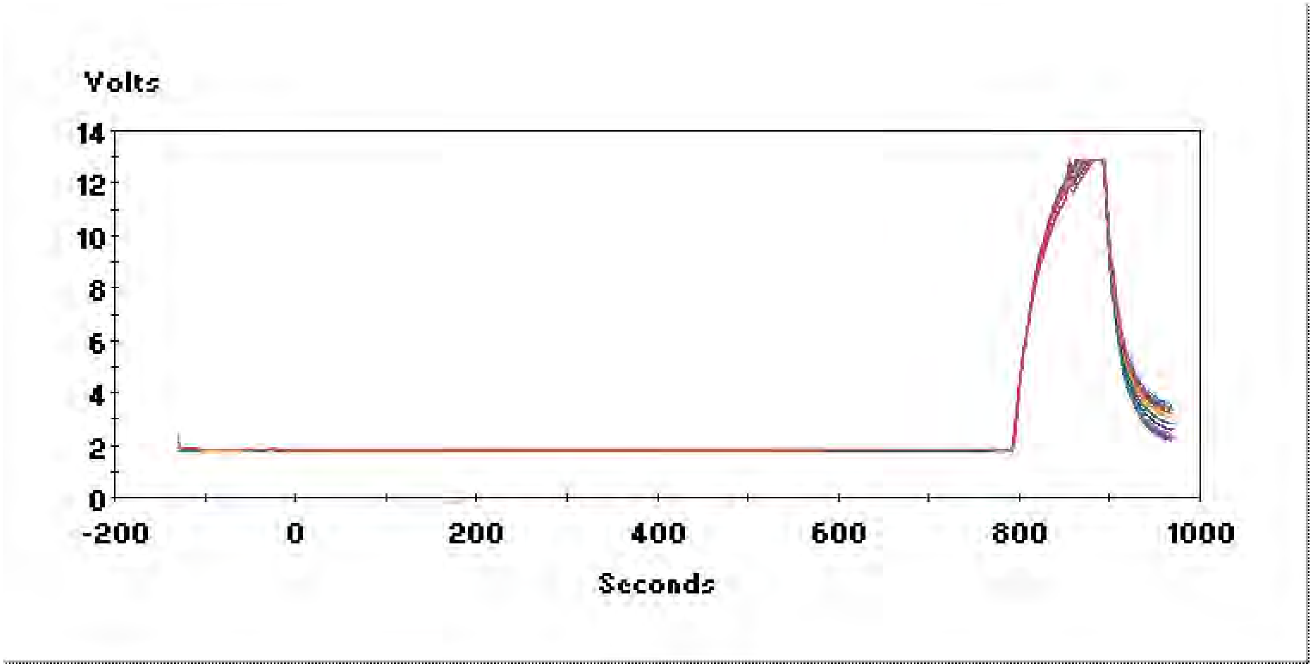

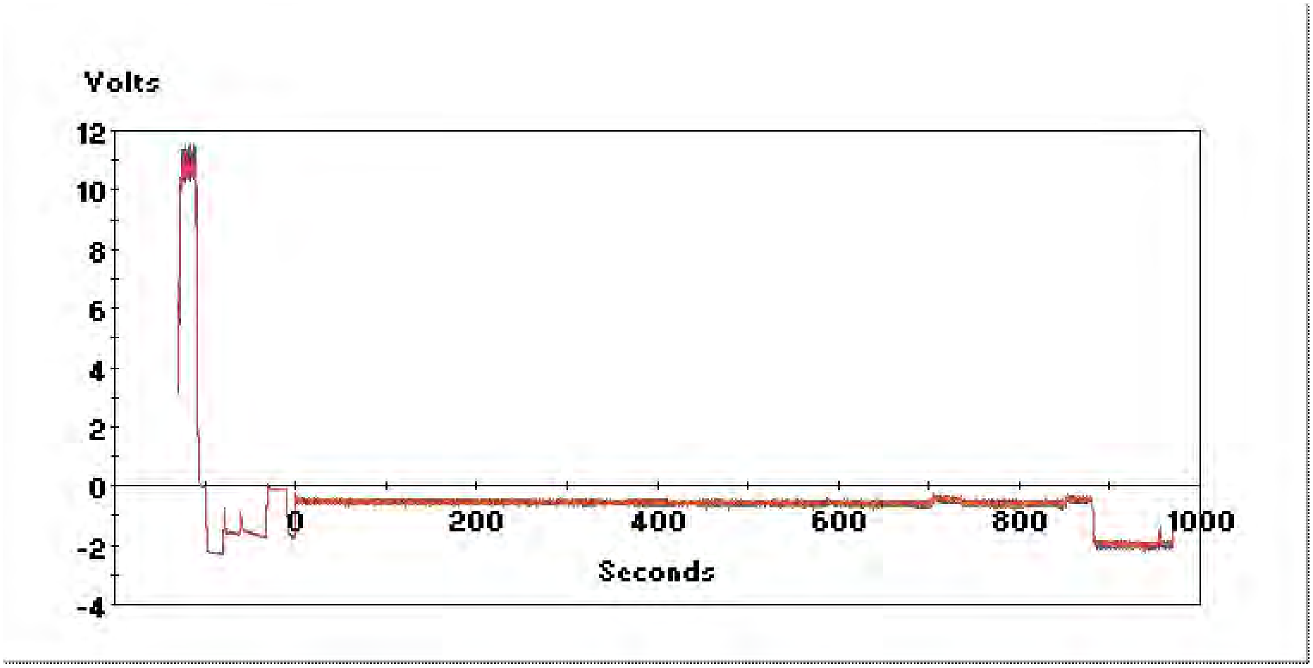

Experiment(x)

**Experiment Name:**	Kinetics Direct 3E2(beads)- BoNTE3	**Start Time:**	Thu Feb 02 14:27:59 2006
**Experiment Type:**	Kinetics, Direct	**End Time:**	
**Binding Site Concentration:**	600.00pM	**Buffer:**	pbs/bsa
**Kd:**	2.28pM	**Label:**	3E4-647
**Titrant:**	1.40nM	**Label Conc:**	7.00ug/ml

Comments(x)

1/750 3E4-647

Timing(x)

Bead Handling (Custom Beads)

Draw Source	Time (sec)	Volume (uL)	Rate (mL/min)	Stir
Backflush	20	0	0.0000	
Buffer	30	500	1.0000	✓
Particle Reservoir	23	383	1.0000	✓
Buffer	40	333	0.5000	
Waste	5	25	0.3000	
Buffer	2	10	0.3000	
Buffer	20	0	0.0000	
Buffer	9	150	1.0000	

Sample Timing

Draw Source	Time (sec)	Volume (uL)	Rate (mL/min)	Time Stamp
Line 3	240	1000	0.2500	
Buffer	30	125	0.2500	
Inject	120	500	0.2500	
Buffer	30	125	0.2500	
Buffer	90	1500	1.0000	

Analysis(x)

Baseline / Endpoints:
5 to 10 (sec) from beginning
10 to 5 (sec) from end

Binding

Ignore	Signal (V)	Time
	0.4340	521.5
	0.2100	1219
	0.1233	1916
	0.0671	2613.5
	0.0555	3311.5
	0.0503	4009
	0.0449	4706
	0.0045	5404

**kon:**	1.080e+006/Ms
**koff:**	2.461e-006/s
**Sig 100%:**	0.84
**NSB:**	0.02
**%Error:**	1.35
**Kd:**	2.28pM
**CBP:**	600.00pM
**Titrant:**	1.40nM

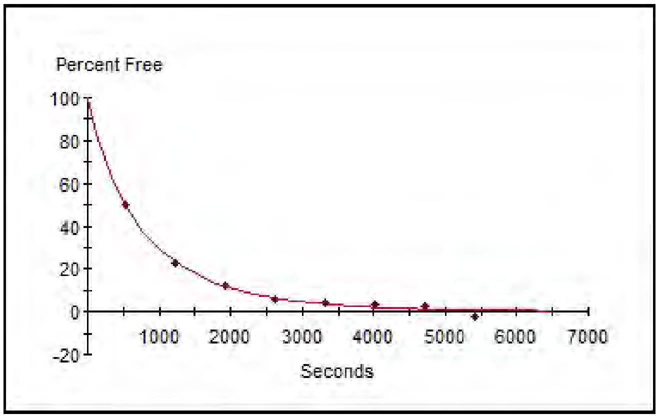

kon: 1.080e+006/Ms
95% confidence interval
kon High: 1.253e+006/Ms
kon Low: 9.268e+005/Ms

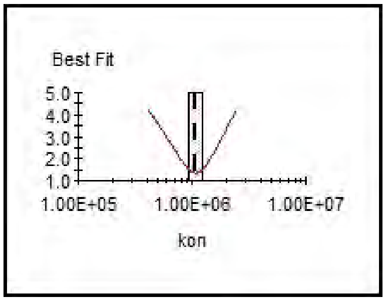

Data Traces(x)

**Cycles:**	14
**Incubation delay (min):**	0
**Mix Time:**	Thu Feb 02 14:24:06 2006

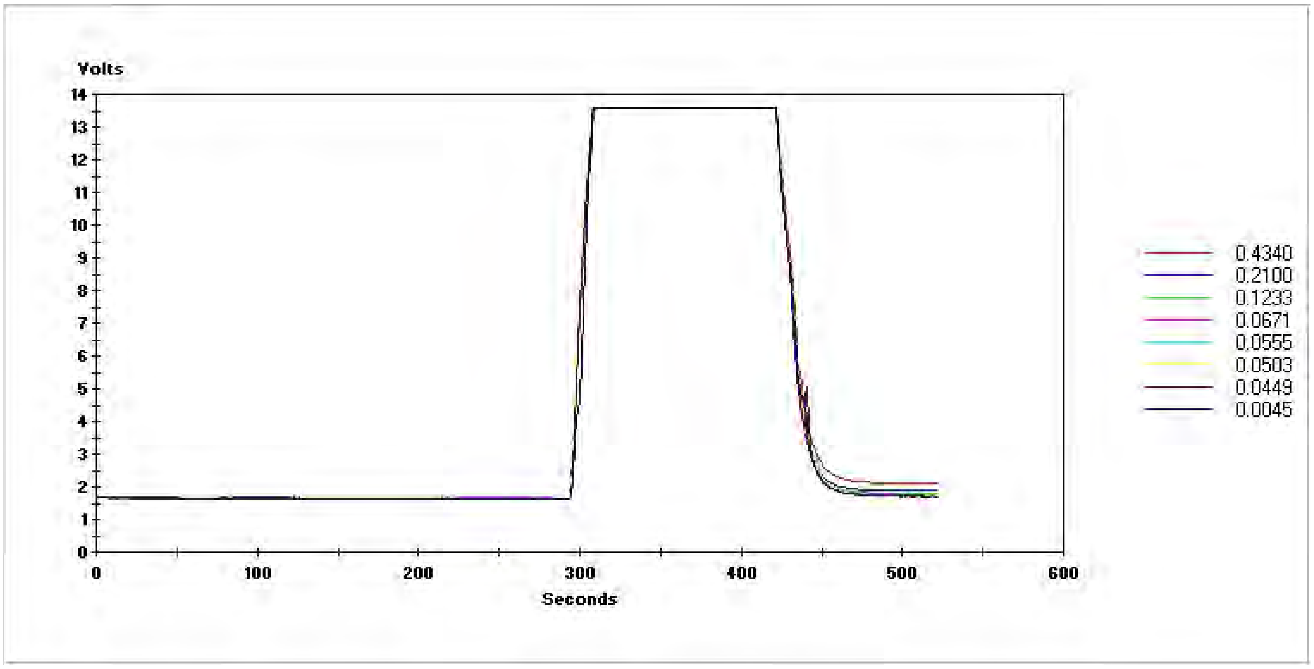

Experiment(x)

**Experiment Name:**	KinDir 3E2 IgG1 vs NXE2 domain 082524	**Start Time:**	Sun Aug 25 16:38:32 2024
**Experiment Type:**	Kinetics, Direct	**End Time:**	Sun Aug 25 18:44:37 2024
**Binding Site Concentration:**	360.00pM	**Buffer:**	PBS
**Kd:**	22.09pM	**Label:**	Anti-His tag-647
**Titrant:**	400.00pM	**Label Conc:**	1.00ug/ml

Comments(x)

3E2 beads 08/23/24

CBP 360pM NXE2 domain (from 3.6uM stock)

3E2 IgG1 400pM (from 07/29/05 stock)

Anti-His-647 1:500

3E2 beads 08/23/24

CBP 360pM NXE2 domain (from 3.6uM stock)

3E2 IgG1 400pM (from 07/29/05 stock)

Anti-His-647 1:500

Timing(x)

Bead Handling (Soft Beads)

Draw Source	Time (sec)	Volume (uL)	Rate (mL/min)	Stir
Backflush	20	0	0.0000	
Buffer	20	500	1.5000	✓
Particle Reservoir 1	23	380	1.0000	✓
Buffer	40	333	0.5000	
Waste	5	25	0.3000	
Buffer	9	150	1.0000	

Sample Timing

Draw Source	Time (sec)	Volume (uL)	Rate (mL/min)	Time Stamp
Rack 2: Tube 1	120	500	0.2500	
Buffer	30	125	0.2500	
Rack 2: Tube 60	120	500	0.2500	
Buffer	30	125	0.2500	
Buffer	90	1500	1.0000	

Analysis(x)

Baseline / Endpoints:

to (sec) from beginning

to (sec) from end

Binding		
Ignore	Signal (V)	Time
	0.6104	280.5
	0.3773	967
	0.3561	1653
	0.2009	2340
	0.2241	3026.5
	0.2574	3713.5
	0.2499	4400.5
	0.1814	5088.5
	0.2235	5775.5
	0.2032	6463.5
	0.2420	7151

**kon:**	3.954e+06/Ms
**koff:**	8.735e-05/s
**Sig 100%:**	0.85
**NSB:**	0.08
**%Error:**	4.09
**Kd:**	22.09pM
**CBP:**	360.00pM
**Titrant:**	400.00pM

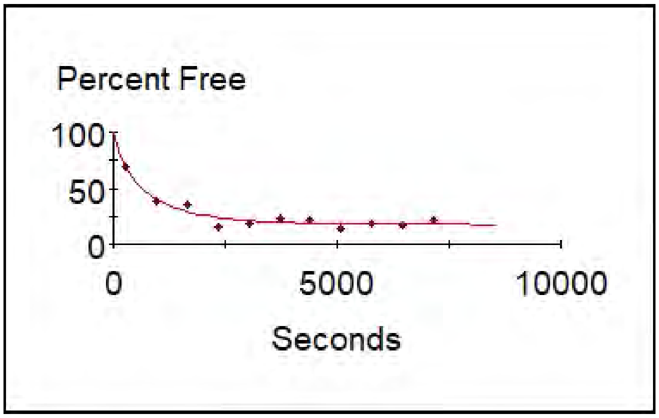

**kon:**	3.954e+06/Ms
**95% confidence interval**	
**kon High:**	6.450e+06/Ms
**kon Low:**	2.429e+06/Ms

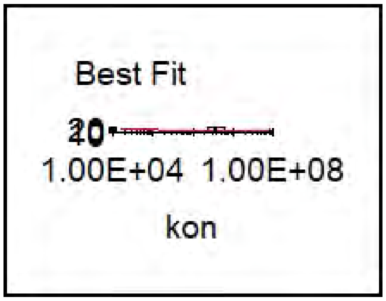

Data Traces(x)

**Cycles:**	11
**Incubation delay (min):**	0
**Mix Time:**	Sun Aug 25 16:38:38 2024

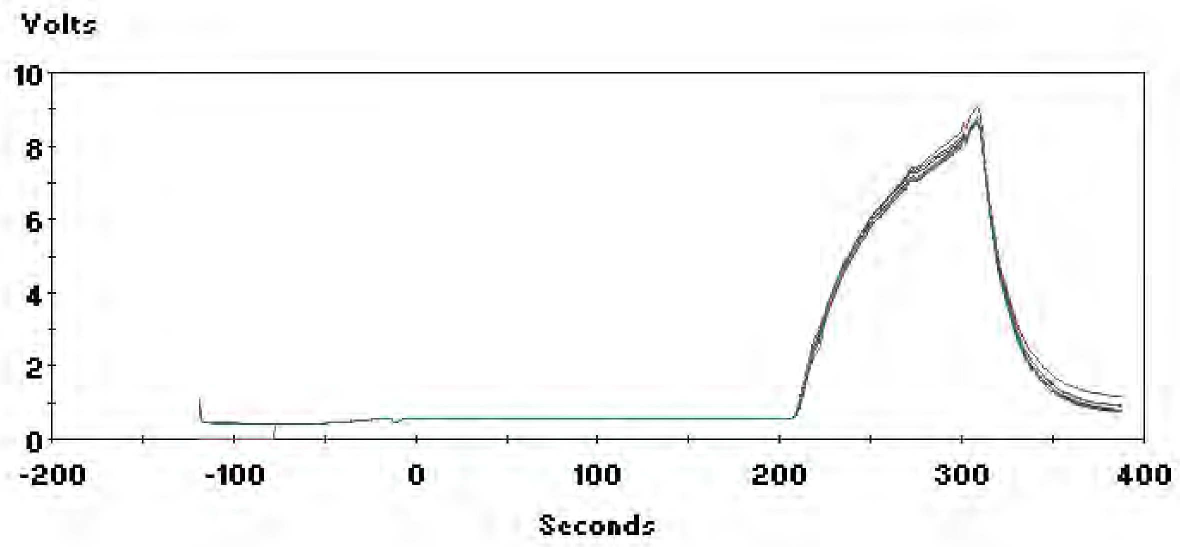

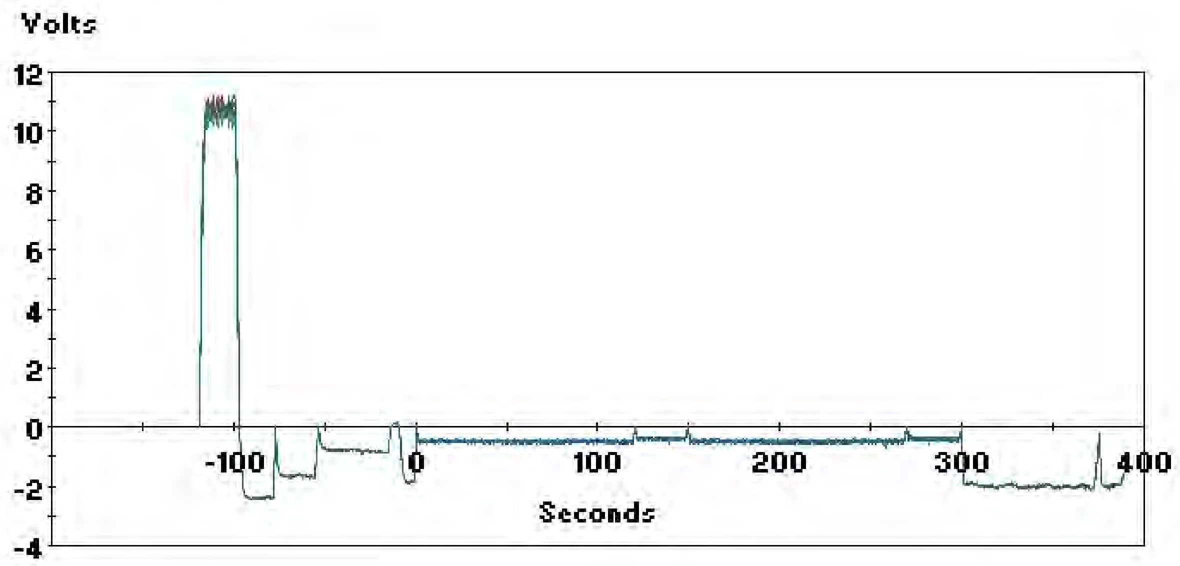

Experiment(x)

**Experiment Name:**	KinDir 3E6.2 IgG1 vs NXE6 domain 082524	**Start Time:**	Sun Aug 25 14:18:37 2024
**Experiment Type:**	Kinetics, Direct	**End Time:**	Sun Aug 25 16:24:44 2024
**Binding Site Concentration:**	360.00pM	**Buffer:**	PBS
**Kd:**	6.71pM	**Label:**	Anti-His tag-647
**Titrant:**	350.00pM	**Label Conc:**	1.00ug/ml

Comments(x)

3E6.2 beads 08/23/24

CBP 360pM NXE6 domain (from 3.6uM stork)

3E6.2 IgG 10/27/07

Anti-His-647 1:500

meter: 0.7029

Timing(x)

Bead Handling (Soft Beads)

Draw Source	Time (sec)	Volume (uL)	Rate (mL/min)	Stir
Backflush	20	0	0.0000	
Buffer	20	500	1.5000	✓
Particle Reservoir 1	23	380	1.0000	✓
Buffer	40	333	0.5000	
Waste	5	25	0.3000	
Buffer	9	150	1.0000	

Sample Timing

Draw Source	Time (sec)	Volume (uL)	Rate (mL/min)	Time Stamp
Rack 2: Tube 1	120	500	0.2500	
Buffer	30	125	0.2500	
Rack 2: Tube 60	120	500	0.2500	
Buffer	30	125	0.2500	
Buffer	90	1500	1.0000	

Analysis(x)

Baseline / Endpoints:

to (sec) from beginning

to (sec) from end

Binding

Ignore	Signal (V)	Time
	0.5853	294.5
	0.3066	980.5
	0.2571	1668
	0.2009	2354.5
	0.2184	3041.5
	0.2099	3728.5
	0.2039	4415.5
	0.1846	5103
✓	0.2970	5790.5
	0.1758	6478
	0.1526	7166

**kon:**	8.314e+06/Ms
**koff:**	5.579e-05/s
**Sig 100%:**	1.03
**NSB:**	0.04
**%Error:**	1.50
**Kd:**	6.71pM
**CBP:**	360.00pM
**Titrant:**	350.00pM

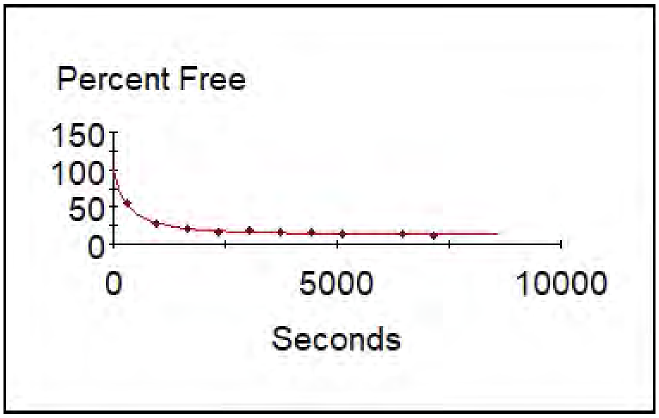

**kon:**	8.314e+06/Ms
**95% confidence interval**	
**kon High:**	1.158e+07/Ms
**kon Low:**	6.083e+06/Ms

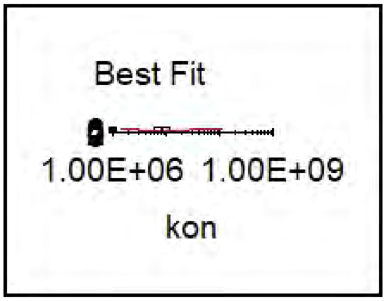

Data Traces(x)

**Cycles:**	11
**Incubation delay (min):**	0
**Mix Time:**	Sun Aug 25 14:18:30 2024

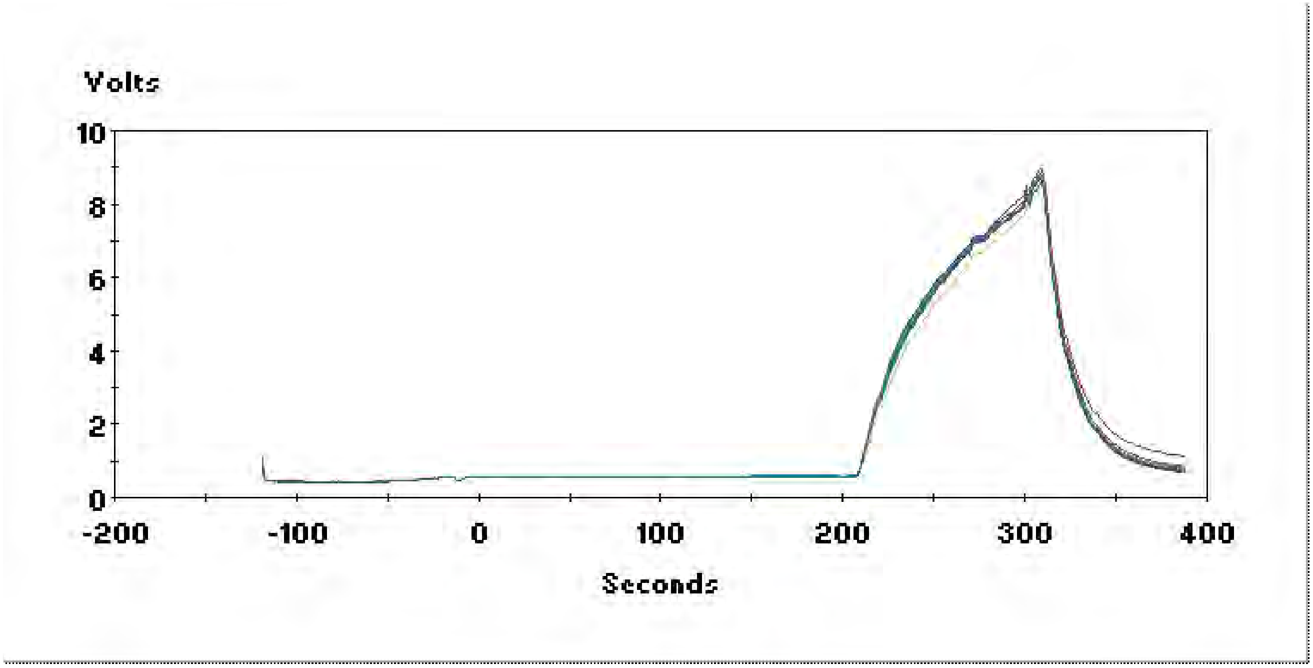

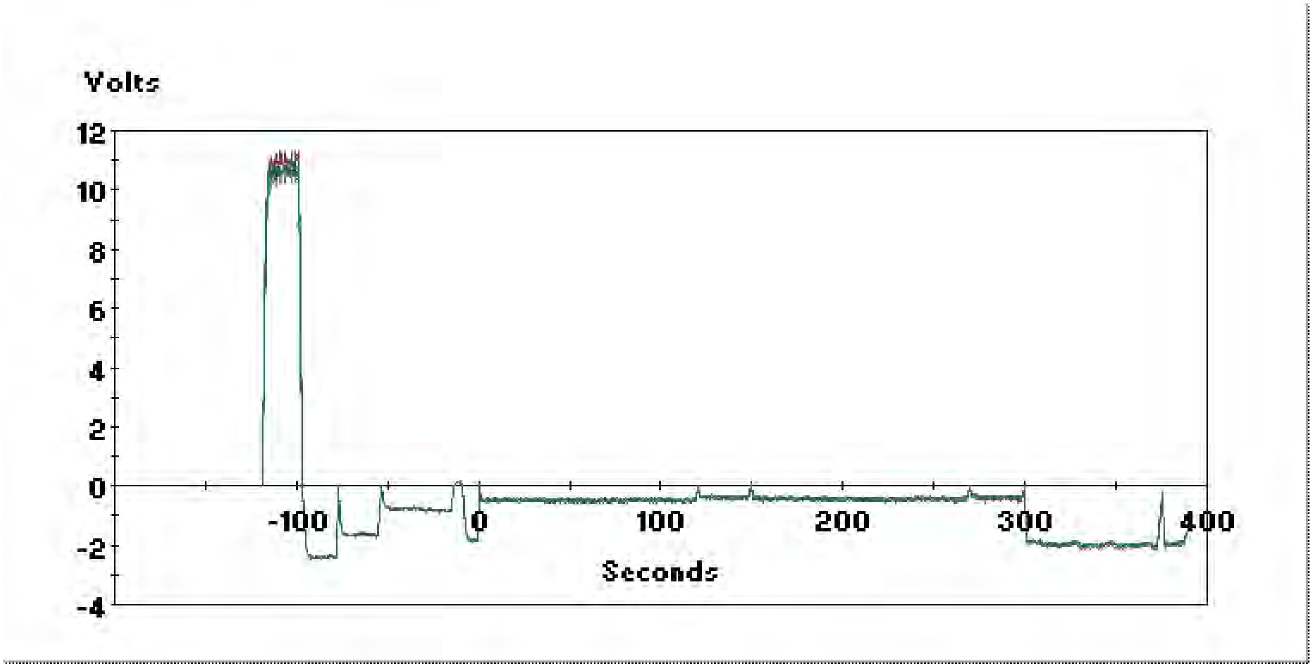

Experiment(x)

**Experiment Name:**	Kinetics Direct 3E6.2 vs BoNTE3	**Start Time:**	Fri Mar 14 14:16:12 2008
**Experiment Type:**	Kinetics, Direct	**End Time:**	Fri Mar 14 16:18:25 2008
**Binding Site Concentration:**	90.00pM	**Buffer:**	PBS
**Kd:**	8.55pM	**Label:**	3E2-647
**Titrant:**	400.00pM	**Label Conc:**	1.00ug/ml

Comments(x)

3E6.1 beads 3/12/08

BoNT E3 11/19/07

3E6.2 IgG 12/3/07

3E2-647 1:2000

meter: 0.7070

Timing(x)

Bead Handling (Soft Beads)

Draw Source	Time (sec)	Volume (uL)	Rate (mL/min)	Stir
Backflush	20	0	0.0000	
Buffer	20	500	1.5000	✓
Particle Reservoir	20	333	1.0000	✓
Buffer	40	333	0.5000	
Waste	5	25	0.3000	
Buffer	2	10	0.3000	
Buffer	20	0	0.0000	
Buffer	9	150	1.0000	

Sample Timing

Draw Source	Time (sec)	Volume (uL)	Rate (mL/min)	Time Stamp
Line 1	120	500	0.2500	
Buffer	30	125	0.2500	
Inject	120	500	0.2500	
Buffer	30	125	0.2500	
Buffer	90	1500	1.0000	

Analysis(x)

Baseline / Endpoints:
5 to 10 (sec) from beginning
10 to 5 (sec) from end

Ignore	Binding Signal (V)	Time
	0.7112	379
	0.4416	943.5
✓	0.4373	1507
	0.3192	2071
	0.2731	2636
✓	0.6064	3200
	0.2259	3763.5
✓	0.2670	4327
	0.1728	4891
✓	0.3123	5455
	0.1523	6019
	0.1518	6583
	0.1557	7147

kon: 2.136e+006/Ms
koff: 1.827e-005/s
Sig 100%: 0.89
NSB: 0.14
%Error: 2.62
Kd: 8.55pM
CBP: 90.00pM
Titrant: 400.00pM

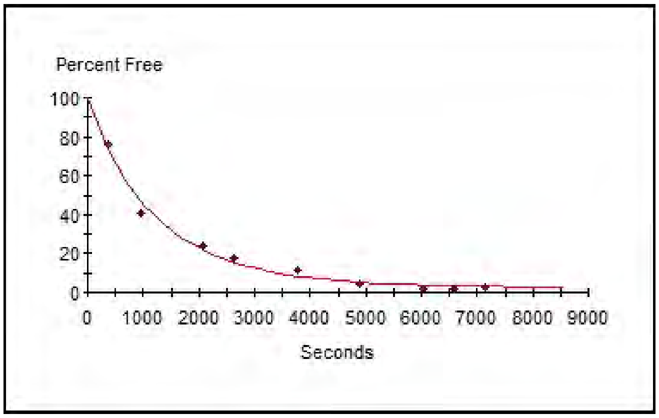

kon: 2.136e+006/Ms
95% confidence interval
kon High: 2.705e+006/Ms
kon Low: 1.687e+006/Ms

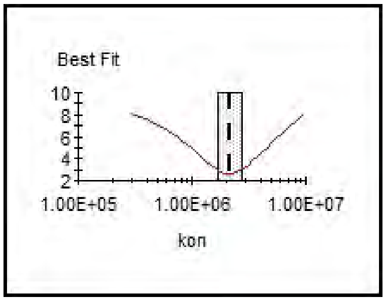

Data Traces(x)

**Cycles:**	13
**Incubation delay (min):**	0
**Mix Time:**	Fri Mar 14 14:13:28 2008

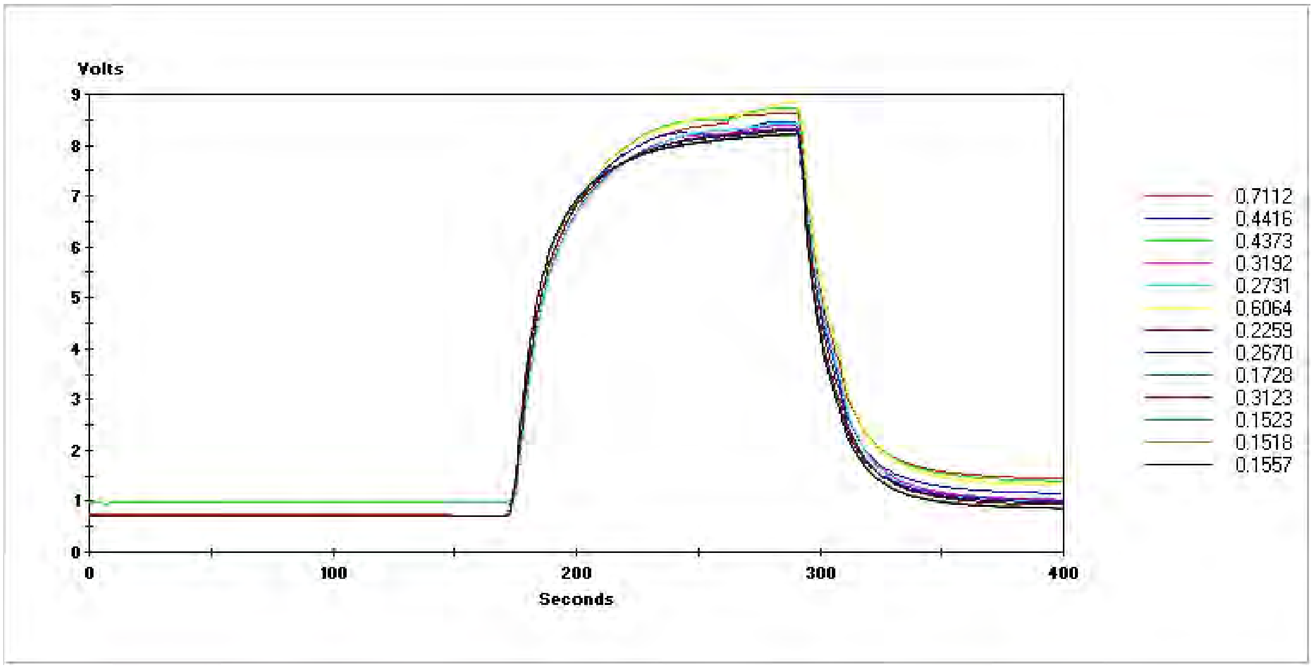

Experiment(x)

**Experiment Name:**	Kinetics Direct 4E17.1(beads)-BoNTE3 redo	**Start Time:**	Thu Sep 07 12:39:30 2006
**Experiment Type:**	Kinetics, Direct	**End Time:**	
**Binding Site Concentration:**	200.00pM	**Buffer:**	pbs/bsa
**Kd:**	239.58pM	**Label:**	3E2-647
**Titrant:**	950.00pM	**Label Conc:**	5.00ug/ml

Comments(x)

3E2-647

Timing(x)

Bead Handling (Soft Beads)

Draw Source	Time (sec)	Volume (uL)	Rate (mL/min)	Stir
Backflush	20	0	0.0000	
Buffer	20	500	1.5000	✓
Particle Reservoir	25	417	1.0000	✓
Buffer	40	333	0.5000	
Waste	5	25	0.3000	
Buffer	2	10	0.3000	
Buffer	20	0	0.0000	
Buffer	9	150	1.0000	

Sample Timing

Draw Source	Time (sec)	Volume (uL)	Rate (mL/min)	Time Stamp
Line 7	120	500	0.2500	
Buffer	30	125	0.2500	
Inject	120	500	0.2500	
Buffer	30	125	0.2500	
Buffer	90	1500	1.0000	

Analysis(x)

Baseline / Endpoints:
5 to 10 (sec) from beginning
10 to 5 (sec) from end

Binding		
**Ignore**	**Signal (V)**	**Time**
	1.0375	393
	0.7731	964
	0.6472	1534
	0.5595	2105
	0.5236	2676
	0.4898	3247
	0.4765	3819
	0.4649	4390
	0.4539	4960
	0.4112	5531.5

**kon:**	7.968e+005/Ms
**koff:**	1.909e-004/s
**Sig 100%:**	1.30
**NSB:**	0.18
**%Error:**	1.16
**Kd:**	239.58pM
**CBP:**	200.00pM
**Titrant:**	950.00pM

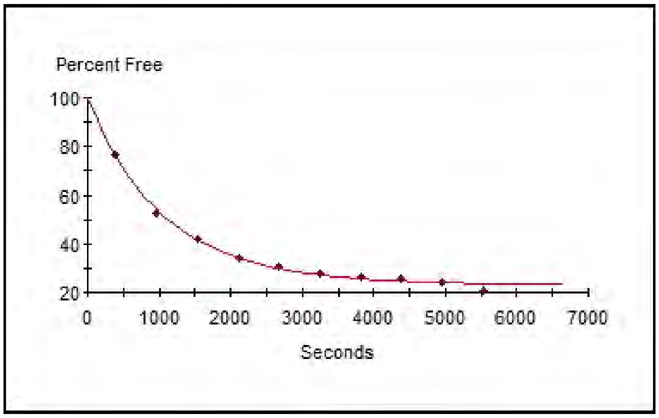

kon: 7.968e+005/Ms
95% confidence interval
kon High: 8.863e+005/Ms
kon Low: 7.151e+005/Ms

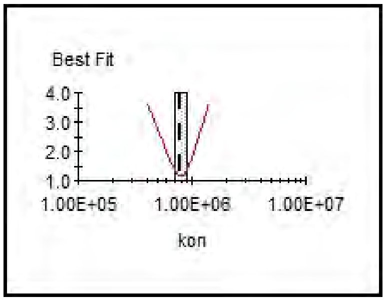

Data Traces(x)

**Cycles:**	14
**Incubation delay (min):**	0
**Mix Time:**	Thu Sep 07 12:36:38 2006

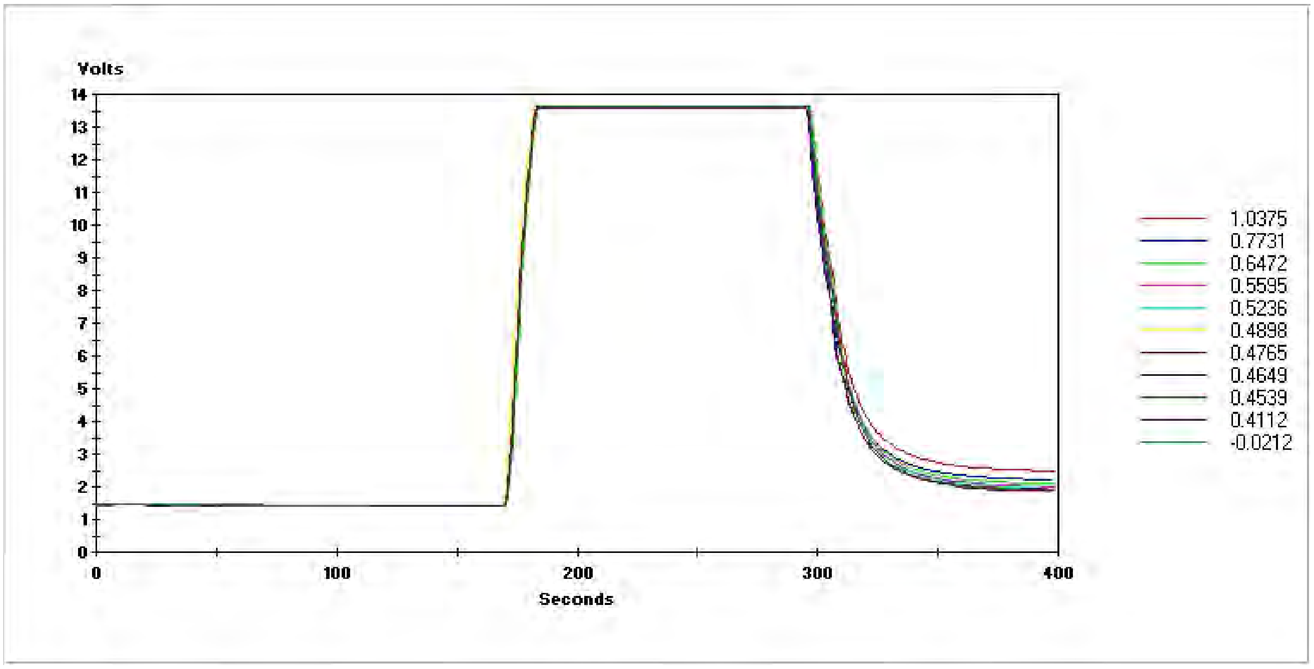

Experiment(x)

**Experiment Name:**	KinDir 4E17.1 IgG1 vs NXE17 domain 082624	**Start Time:**	Mon Aug 26 11:45:35 2024
**Experiment Type:**	Kinetics, Direct	**End Time:**	Mon Aug 26 13:51:41 2024
**Binding Site Concentration:**	349.00pM	**Buffer:**	PBS
**Kd:**	115.63pM	**Label:**	Anti-SV5 tag-647
**Titrant:**	400.00pM	**Label Conc:**	1.00ug/ml

Comments(x)

4E17.1 beads 08/23/24

CBP 349pM NXE17 domain (from 6.98uM stork)

4E17.1 IgG 10/27/07

Anti-SV5-647 1:800

4E17.1 beads 08/23/24

CBP 349pM NXE17 domain (from 6.98uM stork)

4E17.1 IgG 10/27/07

Anti-SV5-647 1:800

Timing(x)

Bead Handling (Soft Beads)

Draw Source	Time (sec)	Volume (uL)	Rate (mL/min)	Stir
Backflush	20	0	0.0000	
Buffer	20	500	1.5000	✓
Particle Reservoir 1	23	380	1.0000	✓
Buffer	40	333	0.5000	
Waste	5	25	0.3000	
Buffer	9	150	1.0000	

Sample Timing

Draw Source	Time (sec)	Volume (uL)	Rate (mL/min)	Time Stamp
Rack 2: Tube 1	120	500	0.2500	
Buffer	30	125	0.2500	
Rack 2: Tube 60	120	500	0.2500	
Buffer	30	125	0.2500	
Buffer	90	1500	1.0000	

Analysis(x)

Baseline / Endpoints:

to (sec) from beginning

to (sec) from end

	**Binding**	
**Ignore**	**Signal (V)**	**Time**
	1.7284	292.5
	0.4924	979
	0.4291	1665.5
	0.2614	2352.5
	0.2712	3039
	0.4337	3726.5
	0.3131	4413.5
	0.2926	5101
	0.2937	5788
	0.2592	6476
	0.2297	7163.5

kon: 5.737e+06/Ms
koff: 6.634e-04/s
Sig 100%: 4.01
NSB: -2.02
%Error: 0.96
Kd: 115.63pM
CBP: 349.00pM
Titrant: 400.00pM

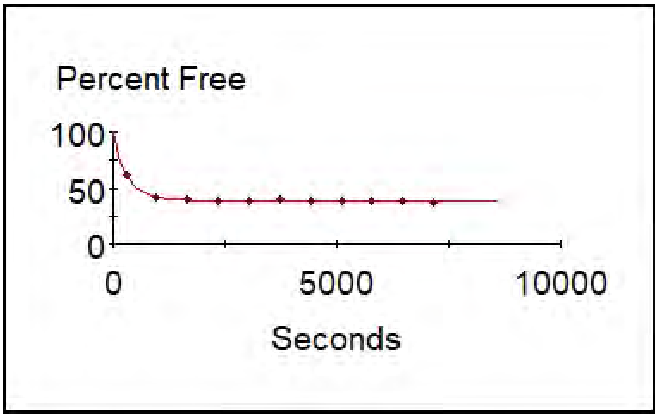

kon: 5.737e+06/Ms
95% confidence interval
kon High: 8.527e+06/Ms
kon Low: 4.364e+06/Ms

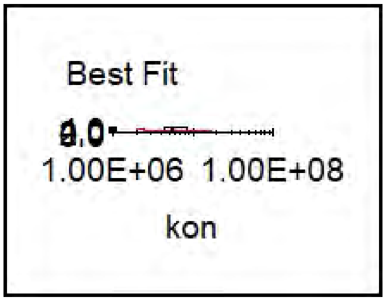

Data Traces(x)

**Cycles:**	11
**Incubation delay (min):**	0
**Mix Time:**	Mon Aug 26 11:45:29 2024

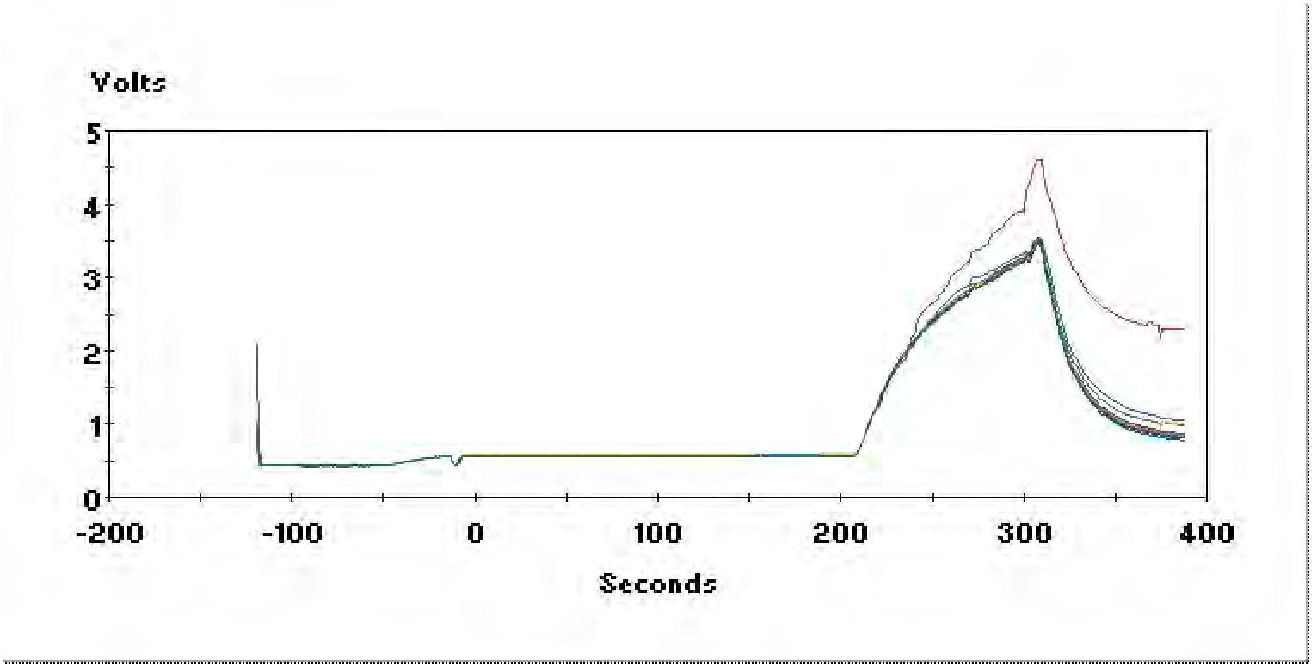

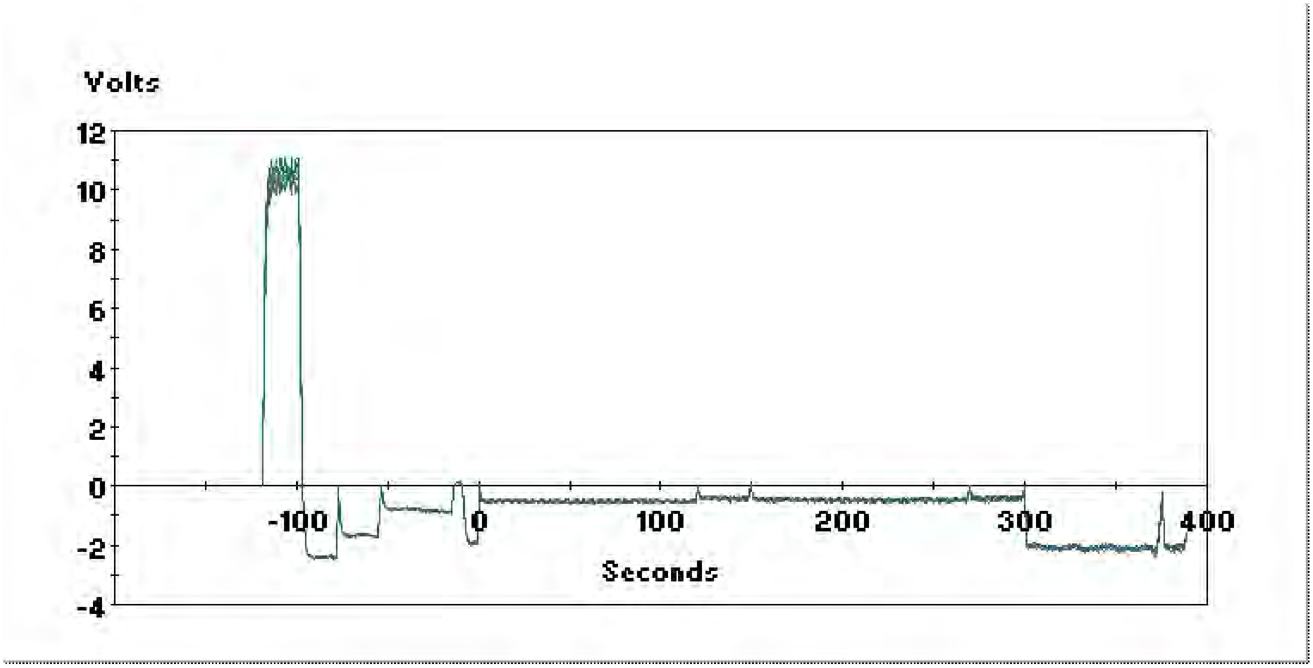

Experiment(x)

**Experiment Name:**	KinDir TsAb-E(3E2) vs BoNT E3{2}	**Start Time:**	Tue Mar 31 14:52:07 2015
**Experiment Type:**	Kinetics, Direct	**End Time:**	Tue Mar 31 16:52:38 2015
**Binding Site Concentration:**	500.00pM	**Buffer:**	PBS/BSA
**Kd:**	1.18pM	**Label:**	3E6.2-647
**Titrant:**	2.50nM	**Label Conc:**	0

Comments(x)

beads: XE02 3/20/15

sample volume: 500 ul

detection: 3E6.2-647

CBP: 500 pM [final] BoNT E3 100208 3/26/15

titrant: 2.5 nM [final] TsAb-E 3/18/15 (260 kDa, 0.3 mg/ml, 1.154 uM)

Timing(x)

Bead Handling (Custom Beads)

Draw Source	Time (sec)	Volume (uL)	Rate (mL/min)	Stir
Backflush	20	0	0.0000	
Buffer	20	500	1.5000	✓
Particle Reservoir 1	20	333	1.0000	✓
Buffer	30	500	1.0000	
Waste	2	8	0.2500	
Buffer	20	0	0.0000	
Buffer	9	150	1.0000	

Sample Timing

Draw Source	Time (sec)	Volume (uL)	Rate (mL/min)	Time Stamp
Rack 2: Tube 1	120	500	0.2500	
Buffer	30	125	0.2500	
Rack 2: Tube 60	120	500	0.2500	
Buffer	30	125	0.2500	
Buffer	90	1500	1.0000	

Analysis(x)

Baseline / Endpoints:
to (sec) from beginning
to (sec) from end

Binding

Ignore	Signal (V)	Time
	1.0125	81
	0.6907	803
	0.4764	1525
	0.3839	2247
	0.3207	2969.5
	0.2507	3692
	0.2694	4414
	0.2512	5137
✓	0.4544	5860
	0.2078	6582.5

**kon:**	3.195e+05/Ms
**koff:**	3.770e-07/s
**Sig 100%:**	1.07
**NSB:**	0.21
**%Error:**	1.55
**Kd:**	1.18pM
**CBP:**	500.00pM
**Titrant:**	2.50nM

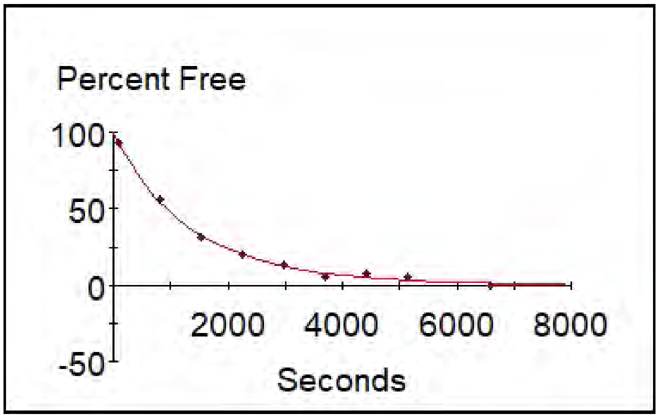

kon: 3.195e+05/Ms
95% confidence interval
kon High: 3.469e+05/Ms
kon Low: 2.937e+05/Ms

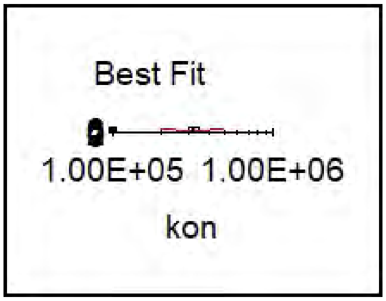

Data Traces(x)

**Cycles:**	10
**Incubation delay (min):**	0
**Mix Time:**	Tue Mar 31 14:56:06 2015

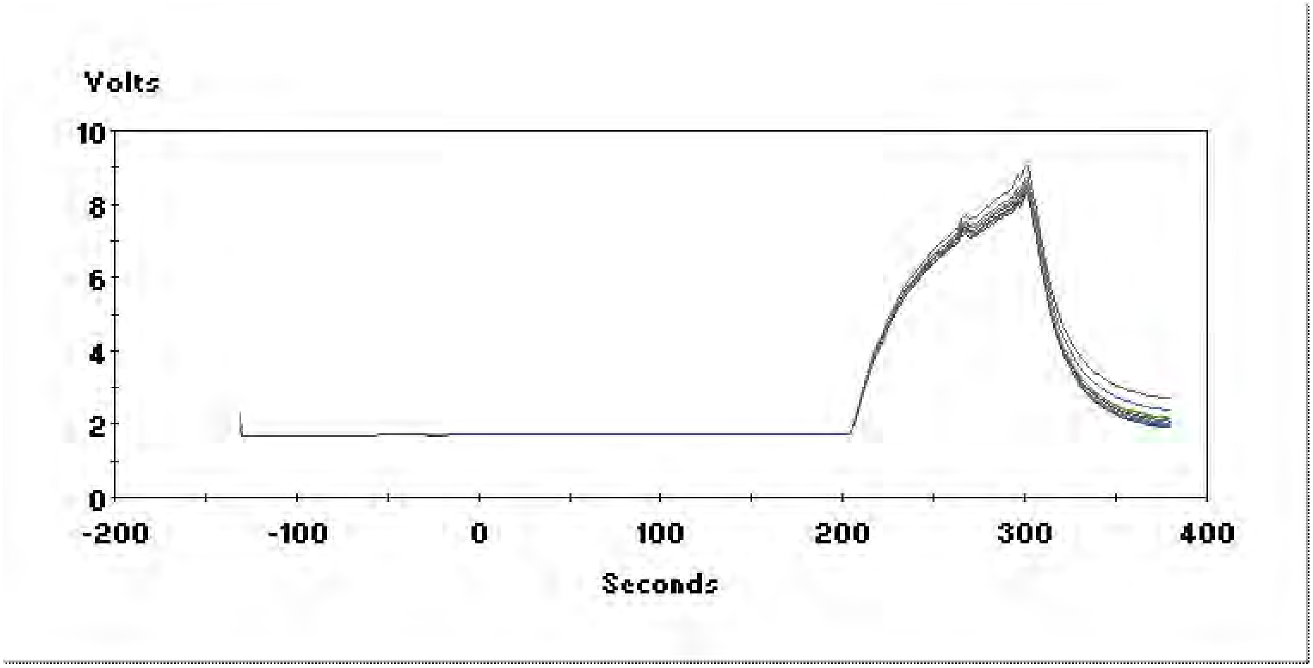

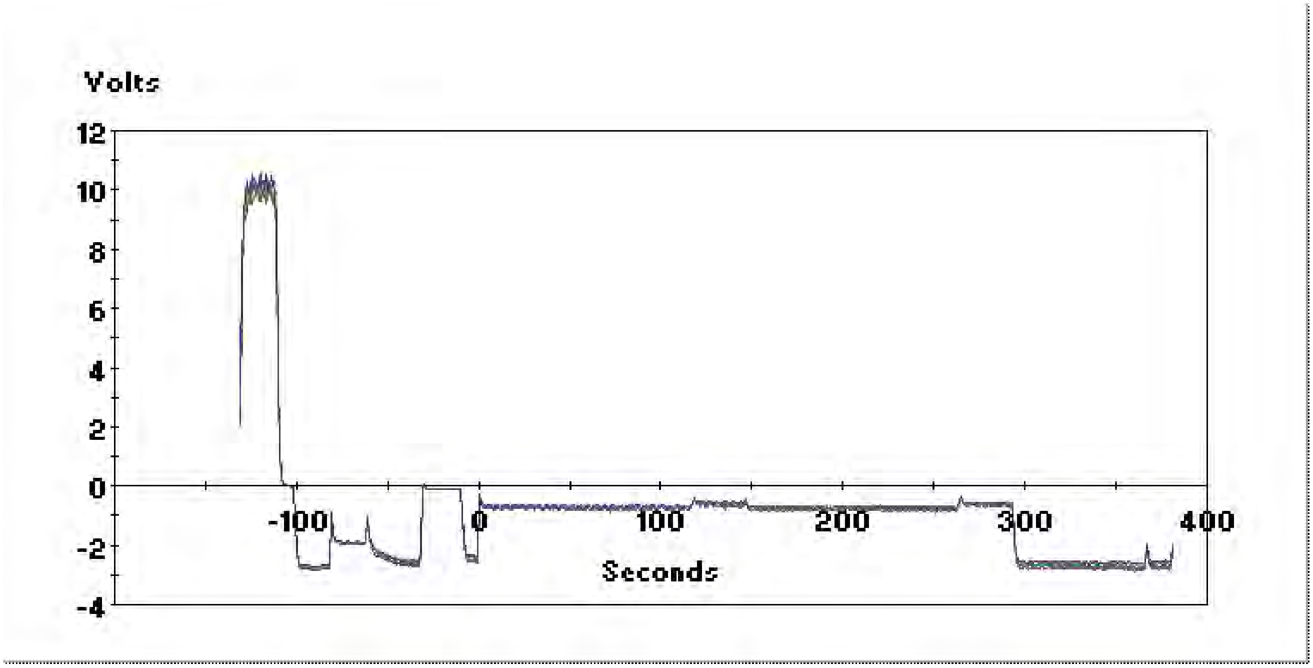

Experiment(x)

**Experiment Name:**	KinDir TsAb-E(3E2) vs LCHN-E02	**Start Time:**	Wed Jun 17 12:02:08 2015
**Experiment Type:**	Kinetics, Direct	**End Time:**	Wed Jun 17 13:02:42 2015
**Binding Site Concentration:**	50.00pM	**Buffer:**	PBS/BSA
**Kd:**	7.48pM	**Label:**	aSV5-647
**Titrant:**	1.00nM	**Label Conc:**	0

Comments(x)

beads: XE02 5/7/15

sample volume: 500 ul

detection: aSV5-647

CBP: 50 pM [final] BoNT LCHN-E02 16907945-132 4/27/15

titrant: 1 nM [final] TsAb-E 3/18/15 (260 kDa, 0.3 mg/ml, 1.154 uM)

Timing(x)

Bead Handling (Custom Beads)

Draw Source	Time (sec)	Volume (uL)	Rate (mL/min)	Stir
Backflush	20	0	0.0000	
Buffer	20	500	1.5000	✓
Particle Reservoir 1	18	300	1.0000	✓
Buffer	30	500	1.0000	
Waste	2	8	0.2500	
Buffer	20	0	0.0000	
Buffer	9	150	1.0000	

Sample Timing

Draw Source	Time (sec)	Volume (uL)	Rate (mL/min)	Time Stamp
Rack 2: Tube 1	120	500	0.2500	
Buffer	30	125	0.2500	
Rack 2: Tube 60	120	500	0.2500	
Buffer	30	125	0.2500	
Buffer	90	1500	1.0000	

Analysis(x)

Baseline / Endpoints:

to (sec) from beginning
to (sec) from end

Binding

Ignore	Signal (V)	Time
	1.4156	81.5
	1.3359	803
	1.2345	1525
	1.1731	2246.5
	1.1114	2968.5
✓	0.0000	0

**kon:**	1.859e+05/Ms
**koff:**	1.391e-06/s
**Sig 100%:**	1.43
**NSB:**	0.67
**%Error:**	0.77
**Kd:**	7.48pM
**CBP:**	50.00pM
**Titrant:**	1.00nM

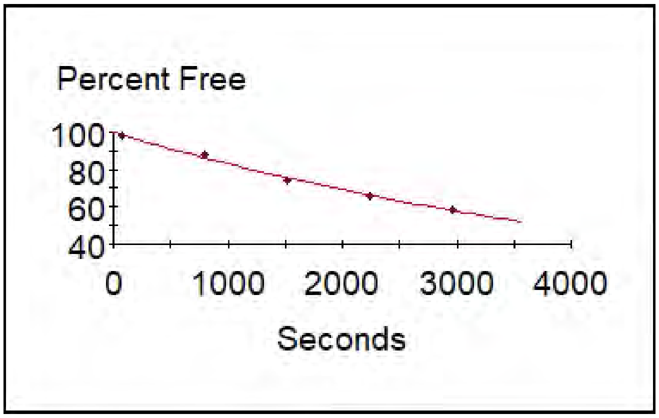

**kon:**	1.859e+05/Ms
**95% confidence interval**	
**kon High:**	2.567e+05/Ms
**kon Low:**	1.088e+05/Ms

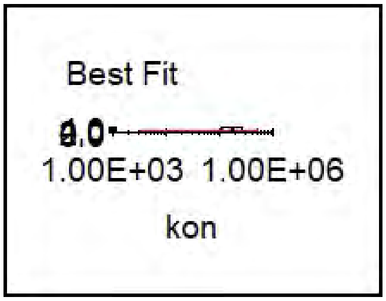

Data Traces(x)

**Cycles:**	10
**Incubation delay (min):**	0
**Mix Time:**	Wed Jun 17 12:06:06 2015

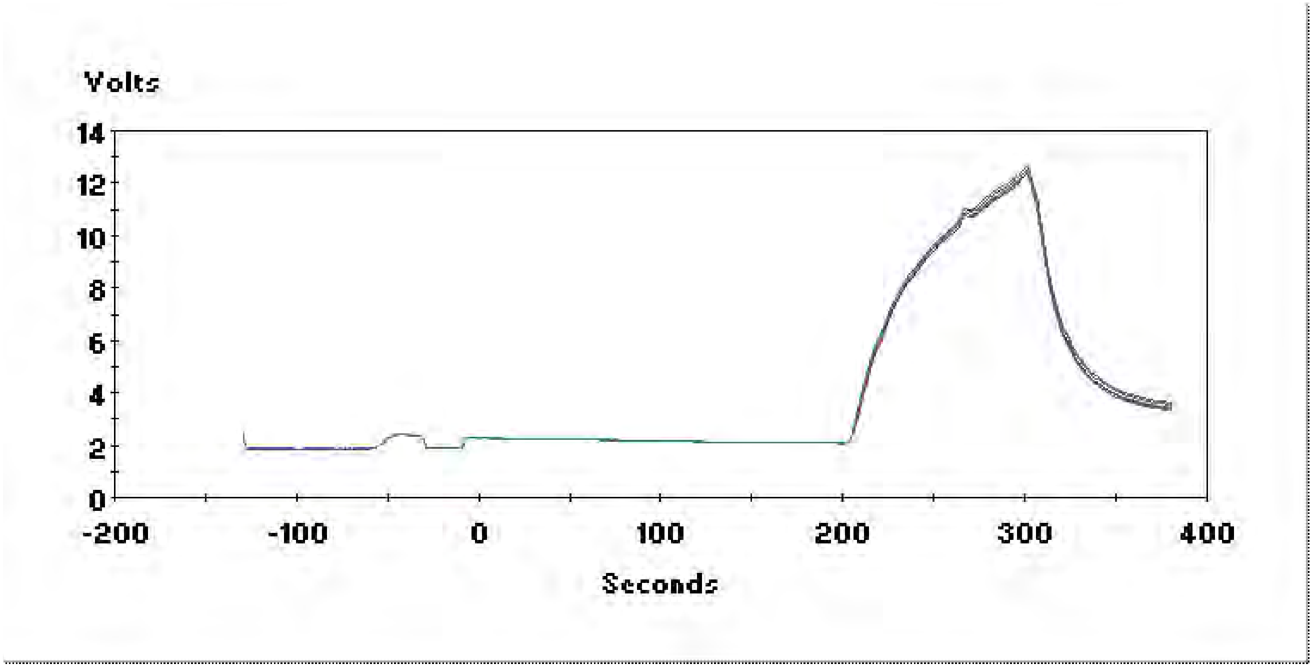

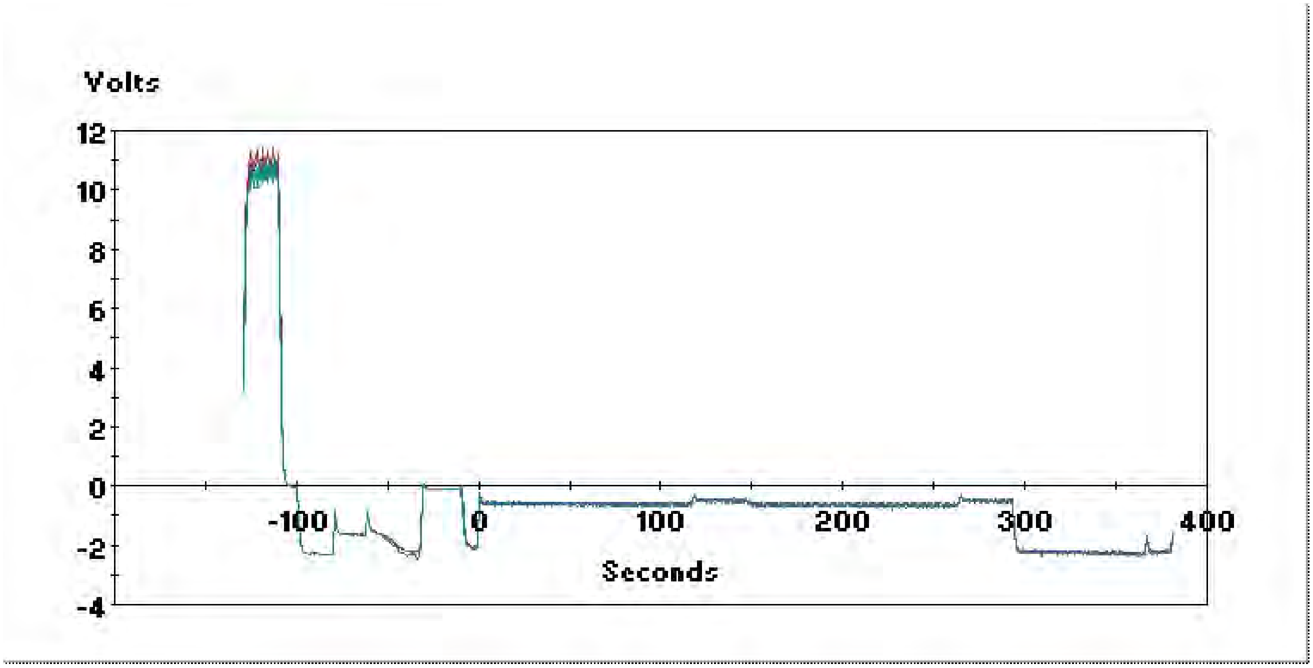

Experiment (x)

**Experiment Name:**	KinDir TsAb-E(3E6.2) vs BoNT E3 2015	**Start Time:**	Tue Mar 31 17:24:55 2015
**Experiment Type:**	Kinetics, Direct	**End Time:**	Tue Mar 31 19:26:31 2015
**Binding Site Concentration:**	250.00pM	**Buffer:**	PBS/BSA
**Kd:**	851.00fM	**Label:**	XE02-647
**Titrant:**	2.00nM	**Label Conc:**	0

Comments (x)

beads: XE06 3/20/15

sample volume: 500 ul

detection: XE02-647

CBP: 250 pM [final] BoNT E3 100208 3/26/15

titrant: 2 nM [final] TsAb-E 3/18/15 (260 kDa, 0.3 mg/ml, 1.154 uM)

Timing (x)

Bead Handling (Custom Beads)

Draw Source	Time (sec)	Volume (uL)	Rate (mL/min)	Stir
Backflush	20	0	0.0000	
Buffer	20	500	1.5000	✓
Particle Reservoir 1	26	433	1.0000	✓
Buffer	30	500	1.0000	
Waste	2	8	0.2500	
Buffer	20	0	0.0000	
Buffer	9	150	1.0000	

Sample Timing

Draw Source	Time (sec)	Volume (uL)	Rate (mL/min)	Time Stamp
Rack 2: Tube 1	120	500	0.2500	
Buffer	30	125	0.2500	
Rack 2: Tube 60	120	500	0.2500	
Buffer	30	125	0.2500	
Buffer	90	1500	1.0000	

Analysis(x)

Baseline / Endpoints:

to (sec) from beginning

to (sec) from end

	Binding	
**Ignore**	**Signal (V)**	**Time**
	1.0323	89.5
	0.7182	818
	0.5042	1546
	0.3757	2275
	0.2863	3004
	0.2426	3732.5
	0.2149	4461.5
	0.1897	5190.5
	0.1747	5919.5
	0.1696	6649

**kon:**	3.290e+05/Ms
**koff:**	2.800e-07/s
**Sig 100%:**	1.09
**NSB:**	0.15
**%Error:**	0.60
**Kd:**	851.00fM
**CBP:**	250.00pM
**Titrant:**	2.00nM

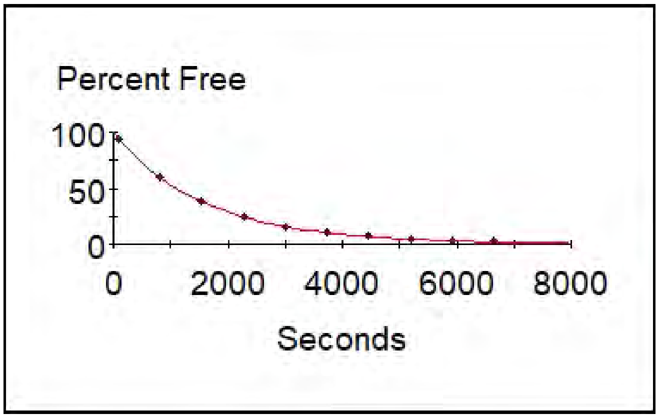

**kon:**	3.290e+05/Ms
**95% confidence interval**	
**kon High:**	3.391e+05/Ms
**kon Low:**	3.189e+05/Ms

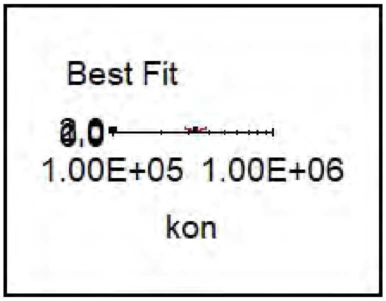

Data Traces(x)

**Cycles:**	10
**Incubation delay (min):**	0
**Mix Time:**	Tue Mar 31 17:28:52 2015

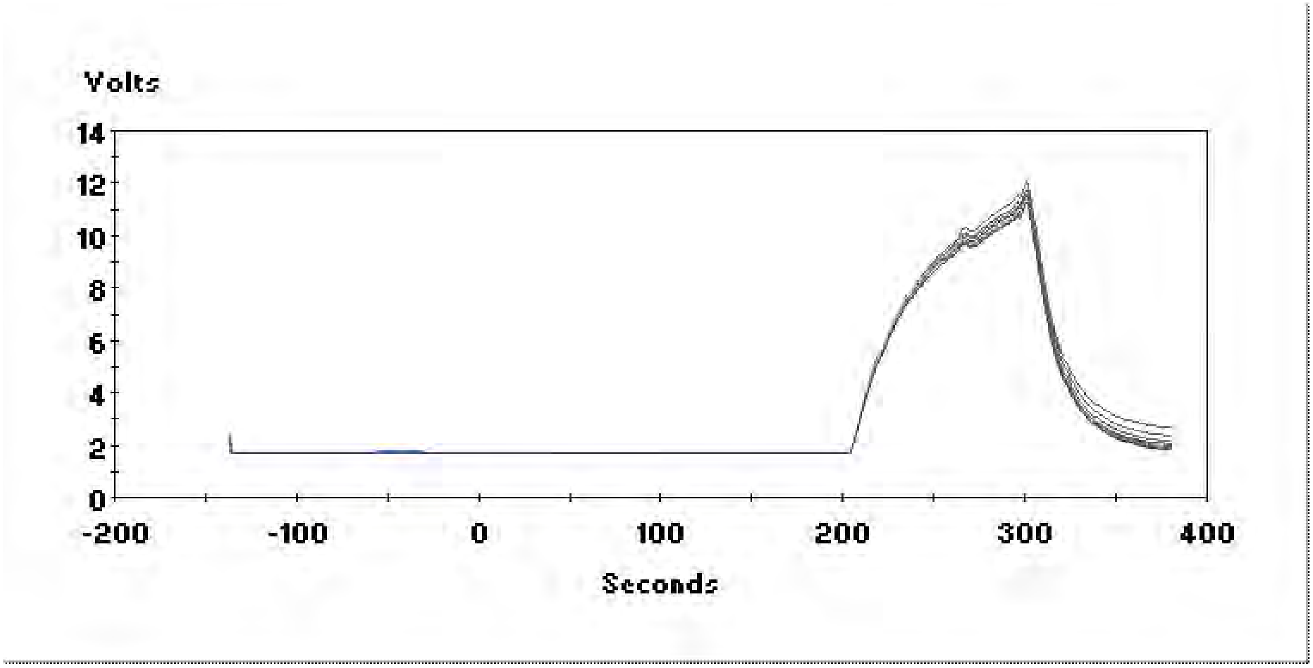

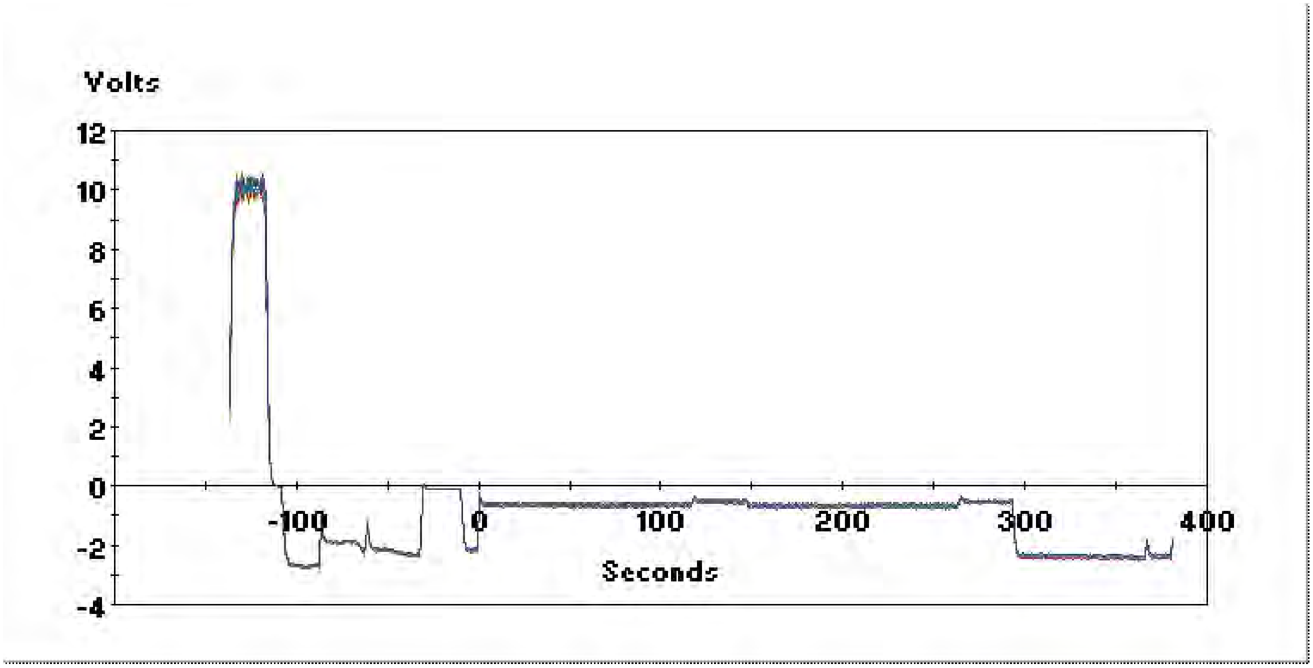

Experiment(x)

**Experiment Name:**	KinDir TsAb-E(3E6.2) vs LCHN-E06 2015	**Start Time:**	Wed Jun 24 11:27:30 2015
**Experiment Type:**	Kinetics, Direct	**End Time:**	Wed Jun 24 13:27:47 2015
**Binding Site Concentration:**	50.00pM	**Buffer:**	PBS/BSA
**Kd:**	13.87pM	**Label:**	aSV5-647
**Titrant:**	5.00nM	**Label Conc:**	0

Comments(x)

beads: XE06 5/13/15

sample volume: 500 ul

detection: aSV5-647

CBP: 50 pM [final] BoNT LCHN-E06 16907945-143 4/27/15

titrant: 5 nM [final] TsAb-E 3/18/15 (260 kDa, 0.3 mg/ml, 1.154 uM)

Timing(x)

Bead Handling (Custom Beads)

Draw Source	Time (sec)	Volume (uL)	Rate (mL/min)	Stir
Backflush	20	0	0.0000	
Buffer	20	500	1.5000	✓
Particle Reservoir 1	17	283	1.0000	✓
Buffer	30	500	1.0000	
Waste	2	8	0.2500	
Buffer	20	0	0.0000	
Buffer	9	150	1.0000	

Sample Timing

Draw Source	Time (sec)	Volume (uL)	Rate (mL/min)	Time Stamp
Rack 2: Tube 1	120	500	0.2500	
Buffer	30	125	0.2500	
Rack 2: Tube 60	120	500	0.2500	
Buffer	30	125	0.2500	
Buffer	90	1500	1.0000	

Analysis(x)

Baseline / Endpoints:

to (sec) from beginning

to (sec) from end

Binding

Ignore	Signal (V)	Time
	1.2872	85
	1.0283	805
	0.8208	1526
	0.6838	2246.5
	0.5900	2967.5
	0.5072	3688
	0.4661	4409.5
	0.4313	5130.5
	0.3781	5852
	0.3758	6573

**kon:**	8.980e+04/Ms
**koff:**	1.246e-06/s
**Sig 100%:**	1.33
**NSB:**	0.32
**%Error:**	0.73
**Kd:**	13.87pM
**CBP:**	50.00pM
**Titrant:**	5.00nM

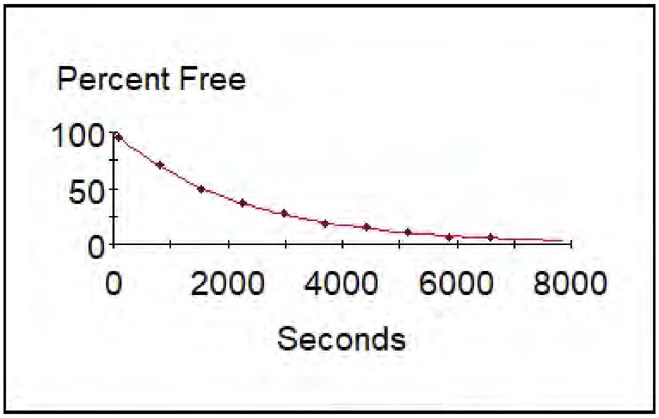

kon: 8.980e+04/Ms

95% confidence interval

kon High: 9.367e+04/Ms

kon Low: 8.595e+04/Ms

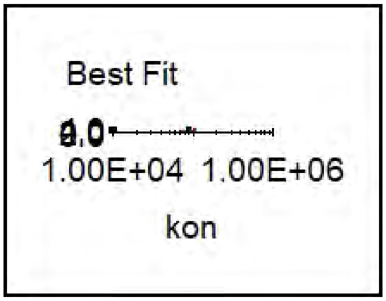

Data Traces(x)

**Cycles:**	10
**Incubation delay (min):**	0
**Mix Time:**	Wed Jun 24 11:31:23 2015

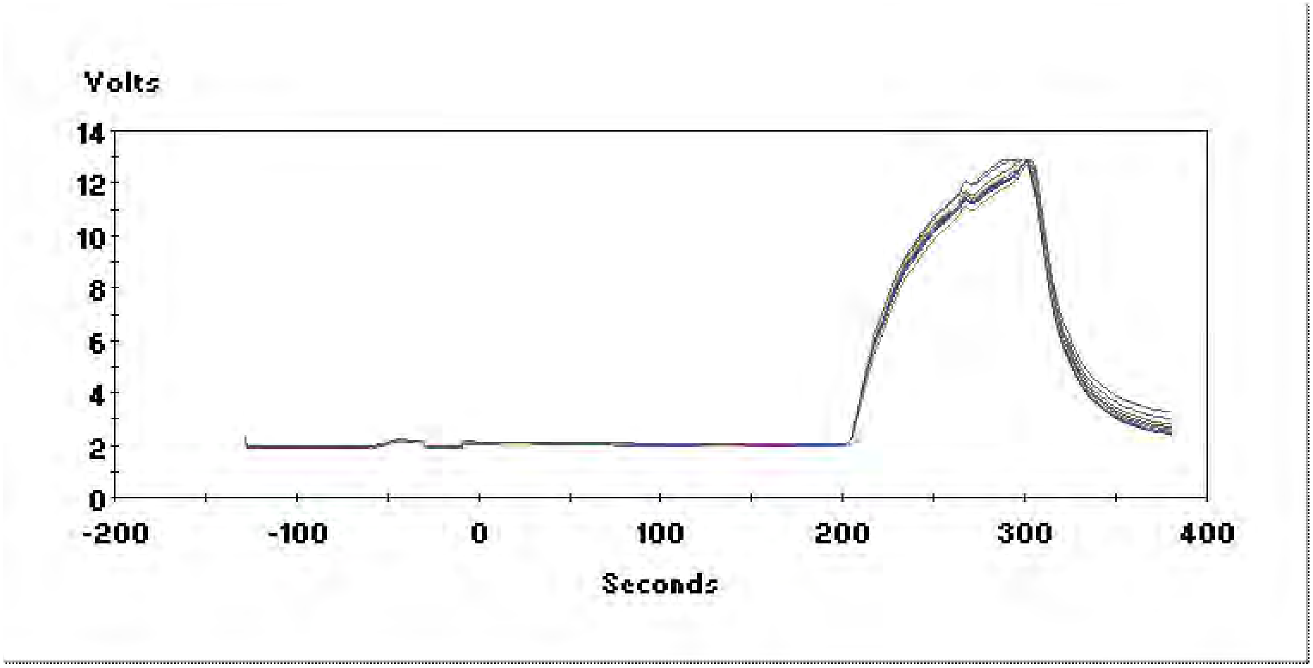

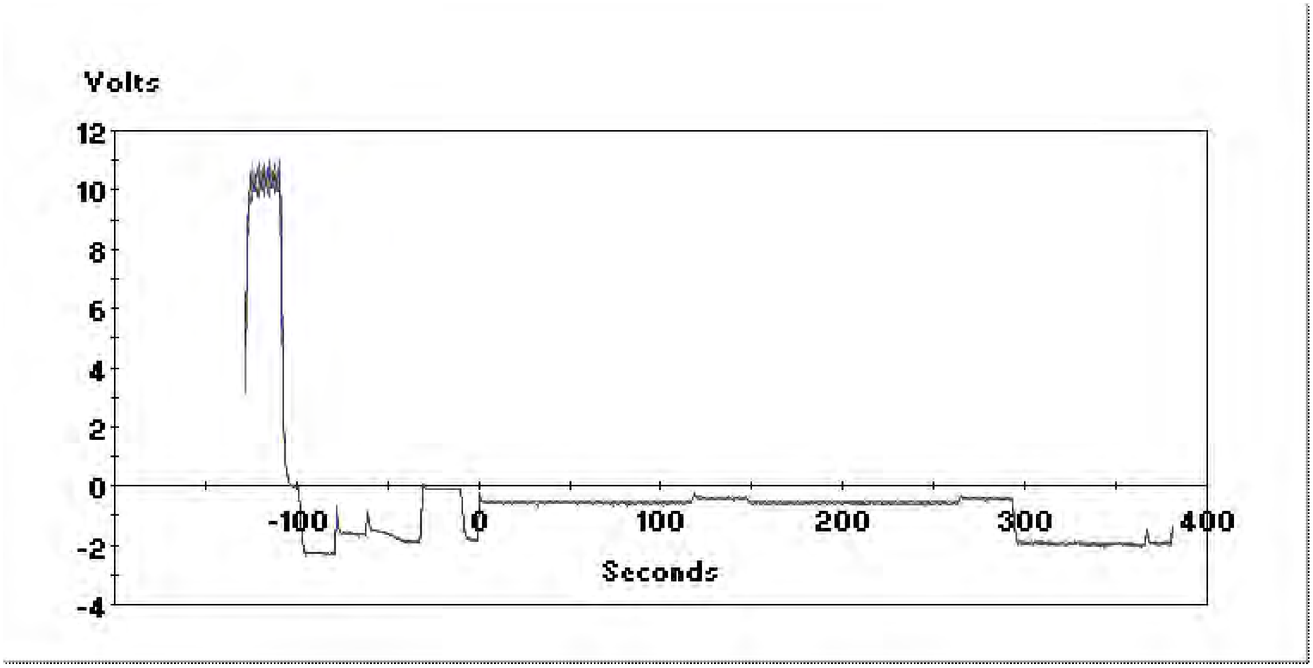

Experiment(x)

**Experiment Name:**	KinDir TsAb-E(4E17.1) vs BoNT E3	**Start Time:**	Wed Apr 1 09:53:34 2015
**Experiment Type:**	Kinetics, Direct	**End Time:**	Wed Apr 1 11:54:06 2015
**Binding Site Concentration:**	500.00pM	**Buffer:**	PBS/BSA
**Kd:**	428.00fM	**Label:**	XE02-647
**Titrant:**	2.00nM	**Label Conc:**	0

Comments(x)

beads: XE17 3/20/15

sample volume: 500 ul

detection: XE02-647

CBP: 500 pM [final] BoNT E3 100208 3/26/15

titrant: 2 nM [final] TsAb-E 3/18/15 (260 kDa, 0.3 mg/ml, 1.154 uM)

Timing(x)

Bead Handling (Custom Beads)

Draw Source	Time (sec)	Volume (uL)	Rate (mL/min)	Stir
Backflush	20	0	0.0000	
Buffer	20	500	1.5000	✓
Particle Reservoir 1	20	333	1.0000	✓
Buffer	30	500	1.0000	
Waste	2	8	0.2500	
Buffer	20	0	0.0000	
Buffer	9	150	1.0000	

Sample Timing

Draw Source	Time (sec)	Volume (uL)	Rate (mL/min)	Time Stamp
Rack 2: Tube 1	120	500	0.2500	
Buffer	30	125	0.2500	
Rack 2: Tube 60	120	500	0.2500	
Buffer	30	125	0.2500	
Buffer	90	1500	1.0000	

Analysis(x)

Baseline / Endpoints:

to (sec) from beginning

to (sec) from end

Binding

Ignore	Signal (V)	Time
	0.9885	86
	0.7408	808
	0.5580	1530
	0.4663	2252.5
	0.3777	2975
	0.3212	3697.5
	0.2945	4420
	0.2566	5142.5
	0.2319	5865.5
	0.2394	6588.5

**kon:**	2.834e+05/Ms
**koff:**	1.213e-07/s
**Sig 100%:**	1.03
**NSB:**	0.19
**%Error:**	0.91
**Kd:**	428.00fM
**CBP:**	500.00pM
**Titrant:**	2.00nM

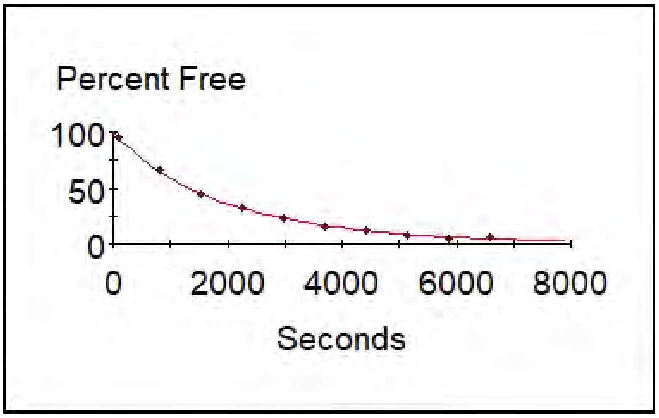

**kon:**	2.834e+05/Ms
**95% confidence interval**	
**kon High:**	2.992e+05/Ms
**kon Low:**	2.677e+05/Ms

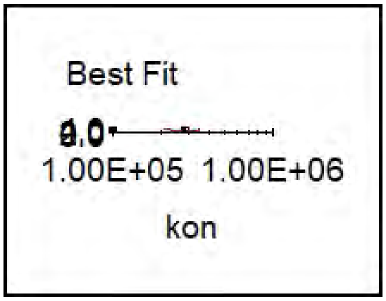

Data Traces(x)

**Cycles:**	10
**Incubation delay (min):**	0
**Mix Time:**	Wed Apr 1 09:57:28 2015

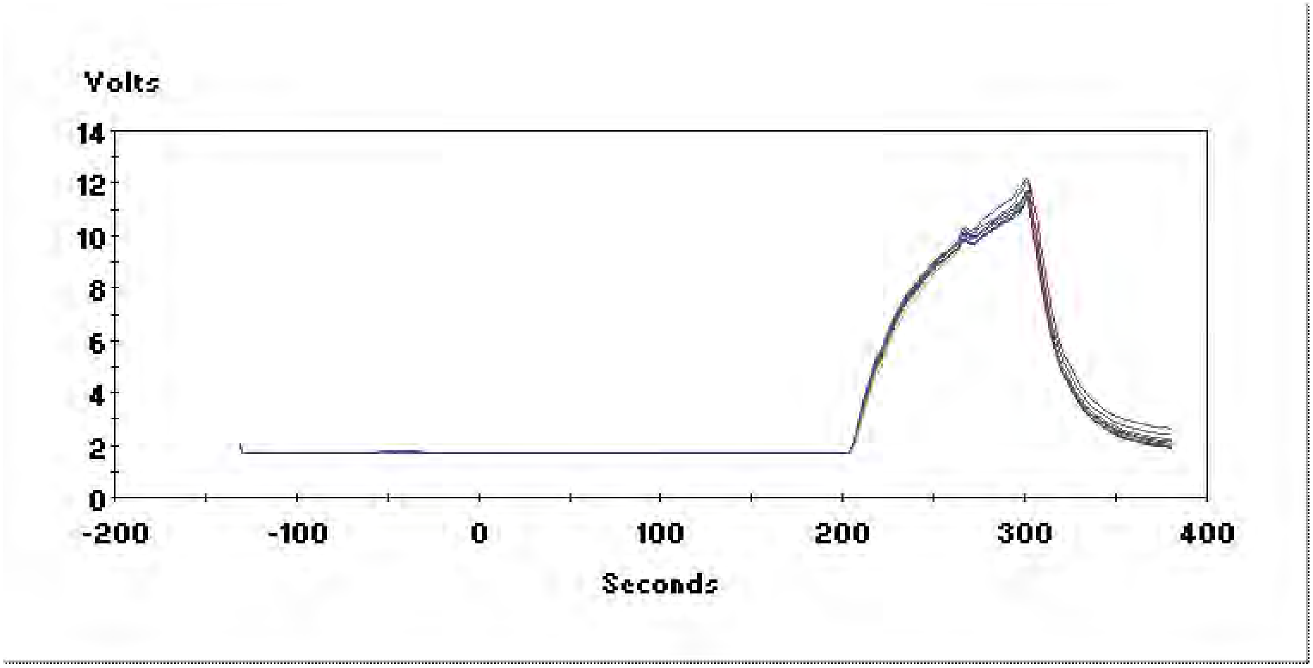

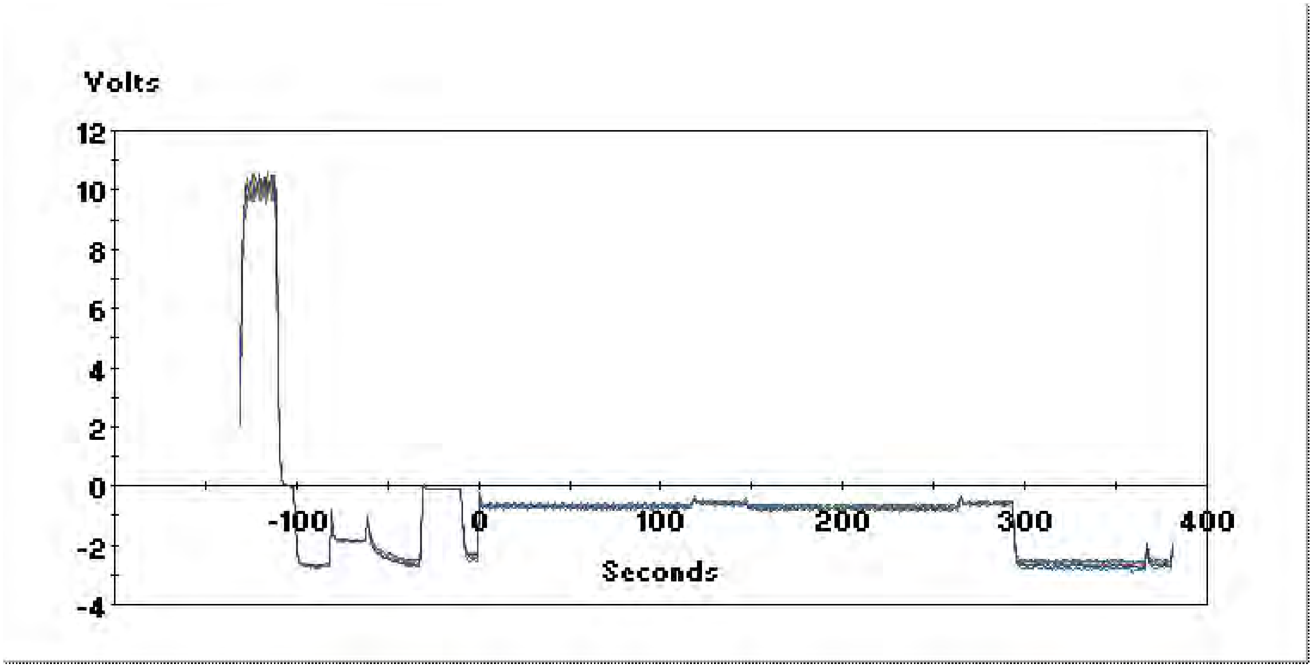

Experiment(x)

**Experiment Name:**	KinDir TsAb-E(4E17.1) vs LCHN-E17	**Start Time:**	Wed Jun 24 14:40:28 2015
**Experiment Type:**	Kinetics, Direct	**End Time:**	Wed Jun 24 16:40:45 2015
**Binding Site Concentration:**	50.00pM	**Buffer:**	PBS/BSA
**Kd:**	18.15pM	**Label:**	aSV5-647
**Titrant:**	5.00nM	**Label Conc:**	0

Comments(x)

beads: XE17 5/13/15

sample volume: 500 ul

detection: aSV5-647

CBP: 50 pM [final] BoNT LCHN-E17 23421972-9 4/27/15

titrant: 5 nM [final] TsAb-E 3/18/15 (260 kDa, 0.3 mg/ml, 1.154 uM)

Timing(x)

Bead Handling (Custom Beads)

Draw Source	Time (sec)	Volume (uL)	Rate (mL/min)	Stir
Backflush	20	0	0.0000	
Buffer	20	500	1.5000	✓
Particle Reservoir 1	17	283	1.0000	✓
Buffer	30	500	1.0000	
Waste	2	8	0.2500	
Buffer	20	0	0.0000	
Buffer	9	150	1.0000	

Sample Timing

Draw Source	Time (sec)	Volume (uL)	Rate (mL/min)	Time Stamp
Rack 2: Tube 1	120	500	0.2500	
Buffer	30	125	0.2500	
Rack 2: Tube 60	120	500	0.2500	
Buffer	30	125	0.2500	
Buffer	90	1500	1.0000	

Analysis(x)

Baseline / Endpoints:
to (sec) from beginning
to (sec) from end

Binding

Ignore	Signal (V)	Time
	0.7836	73
	0.6740	793
	0.5817	1514
	0.5322	2234.5
	0.4881	2955.5
	0.4546	3676.5
	0.4199	4398
	0.4174	5119
	0.3956	5840
	0.3789	6562

**kon:**	8.297e+04/Ms
**koff:**	1.506e-06/s
**Sig 100%:**	0.80
**NSB:**	0.35
**%Error:**	1.17
**Kd:**	18.15pM
**CBP:**	50.00pM
**Titrant:**	5.00nM

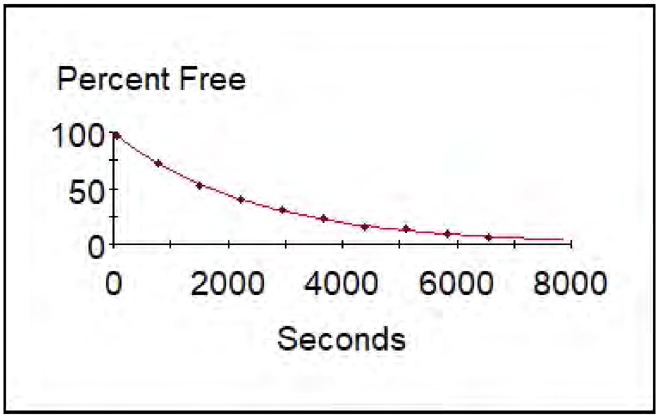

**kon:**	8.297e+04/Ms
**95% confidence interval**	
**kon High:**	8.908e+04/Ms
**kon Low:**	7.690e+04/Ms

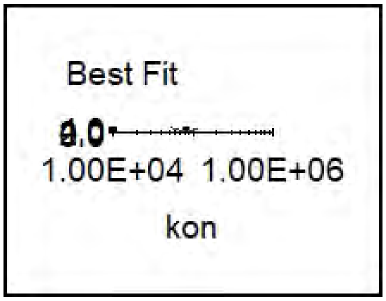

Data Traces(x)

**Cycles:**	10
**Incubation delay (min):**	0
**Mix Time:**	Wed Jun 24 14:44:33 2015

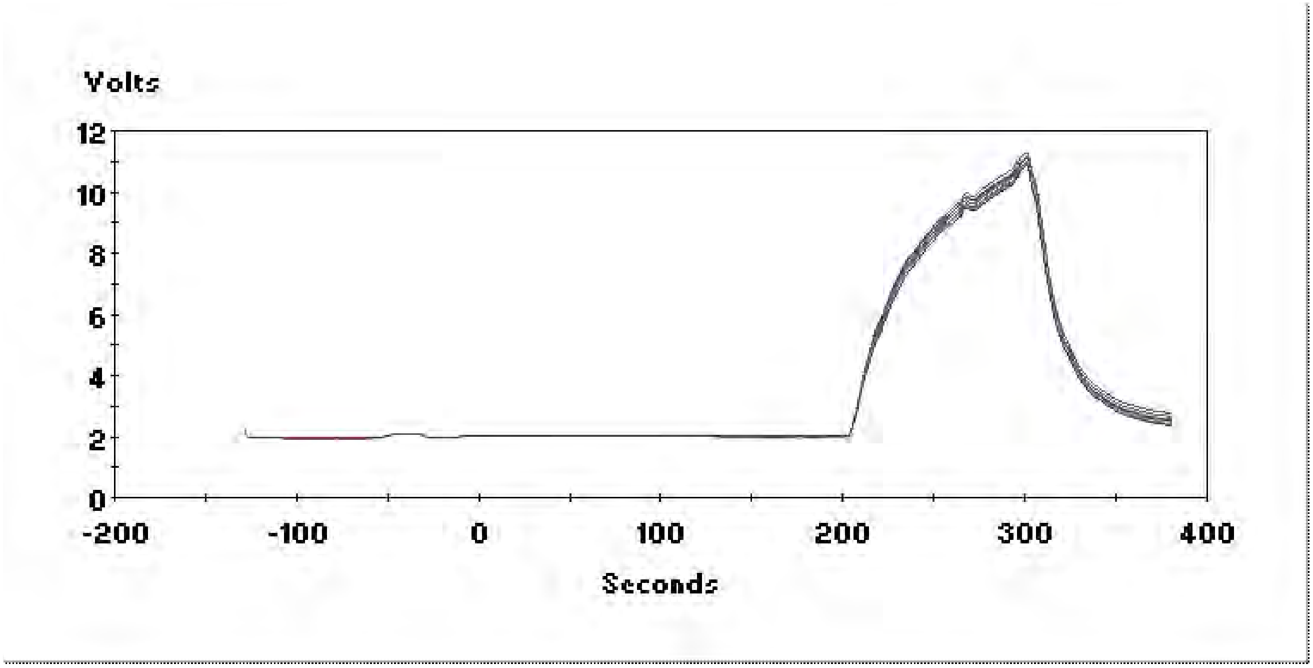

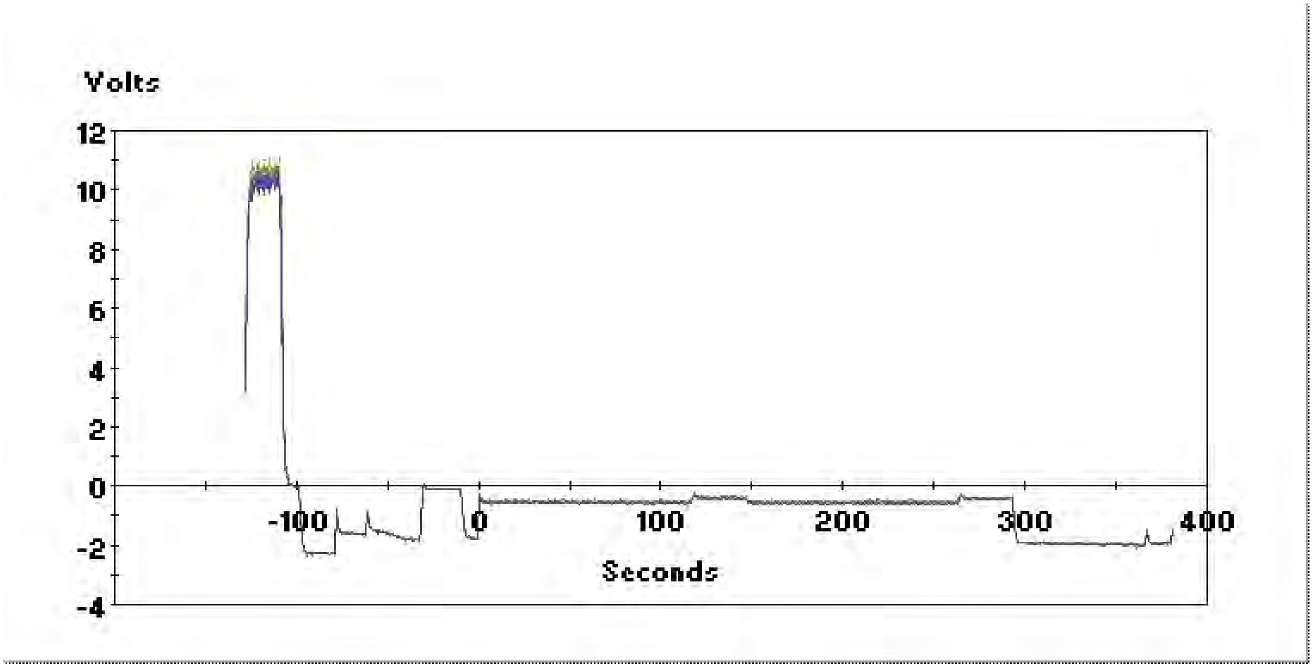

Supplement: Supplementary file 1 [file toxins-17-00281-s001.zip › Fig S2.pdf]
