# Supplementary material for: Single Tri-Epitopic Antibodies (TeAbs) to Botulinum Neurotoxin Serotypes B, E, and F Recapitulate the Full Potency of a Combination of Three Monoclonal Antibodies in Toxin Neutralization
Source: Toxins (Basel). 2025 Jun 4;17(6):281. doi: 10.3390/toxins17060281 (PMC12197607; doi:10.3390/toxins17060281)

Experiment(x)

**Experiment Name:**	KD 6F5.4 IgG1& F1 Toxin 090624	**Start Time:**	Fri Sep 6 23:34:24 2024
**Experiment Type:**	Equilibrium	**End Time:**	Sat Sep 7 10:33:13 2024
**Constant Binding Partner (CBP)**		**Buffer:**	PBS/BSA
**Molecular Concentration:**	200.00pM	**Label:**	Anti-Hu6F15.4&Hu6F10-647
**Valency:**	2	**Label Conc:**	0
**Binding Site Concentration:**	400.00pM		

Comments(x)

beads:6F5.4 IgG1 100ug on 1ml Sepharose 4B

sample volume: 7 ml

detection: Hu6F15.4 &Hu6F10 IgG1-647 (prepared by Lou 090624).1:800

CBP: 200 pM BoNT/F1 090624

titrant: 6F5.4 IgG1

titration: 2 cycles, 14 samples: 2 nM - 0.98pM (1:2) of 6F5.4 IgG1, plus NBS & 200pM BoNT-F1 only ctr

samples:

1-14) titration

1): NBS

2): 200 pM BoNT/F1 only ctr

3 ~ 14): 2 nM - 980fM (1:2) of 6F5.4 IgG1, Incubate 8hr RT

beads:6F5.4 IgG1 100ug on 1ml Sepharose 4B

sample volume: 7 ml

detection: Hu6F15.4 &Hu6F10 IgG1-647 (prepared by Lou 090624).1:800

CBP: 200 pM BoNT/F1 090624

titrant: 6F5.4 IgG1

titration: 2 cycles, 14 samples: 2nM - 980fM (1:2) of 6F5.4 IgG1, plus NBS & 200pM BoNT-F1 only ctr

samples:

1-14) titration

1): NBS

2): 200 pM BoNT/F1 only ctr

3 ~ 14): 2 nM - 980fM (1:2) of 6F5.4 IgG1, Incubate 8hr RT

Timing(x)

Bead Handling (Custom Beads)

Draw Source	Time (sec)	Volume (uL)	Rate (mL/min)	Stir
Backflush	20	0	0.0000	
Buffer	20	500	1.5000	✓
Particle Reservoir 1	23	380	1.0000	✓
Buffer	30	500	1.0000	
Waste	2	8	0.2500	
Buffer	20	0	0.0000	
Buffer	9	150	1.0000	

Sample Timing

Draw Source	Time (sec)	Volume (uL)	Rate (mL/min)	Time Stamp
Sample Set 201-214	720	3000	0.2500	
Buffer	30	125	0.2500	
Rack 1: Tube 21	120	500	0.2500	
Buffer	30	125	0.2500	
Buffer	180	3000	1.0000	

Analysis (x)

Baseline / Endpoints:
to (sec) from beginning
to (sec) from end

Ignore	Binding Signal (V)	Concentration
✓	0.2258	NSB
✓	0.7236	0
	0.2569	2.00nM
	0.2900	1.00nM
	0.3544	500.00pM
	0.4676	250.00pM
	0.5604	125.00pM
	0.6157	62.50pM
	0.6605	31.25pM
	0.6720	15.63pM
	0.6945	7.81pM
	0.6865	3.91pM
	0.6990	1.95pM
	0.7002	976.56fM
✓	0.1591	NSB
✓	0.6867	0
	0.2091	2.00nM
	0.2633	1.00nM
	0.3262	500.00pM
	0.4425	250.00pM
	0.5414	125.00pM
	0.5820	62.50pM
	0.6377	31.25pM
	0.6366	15.63pM
	0.6640	7.81pM
	0.7025	3.91pM
	0.6782	1.95pM
	0.6705	976.56fM

**Kd:**	189.63pM
**Active CBP:**	159.65pM
**CBP %Activity:**	39.91
**Ratio:**	0.8419
**Sig 100%:**	0.70
**Drift (%/run):**	-0.2399
**NSB:**	0.17
**Drift (mV/run):**	-2.6434
**TR NSB:**	2.44e-07
**%Error:**	1.75

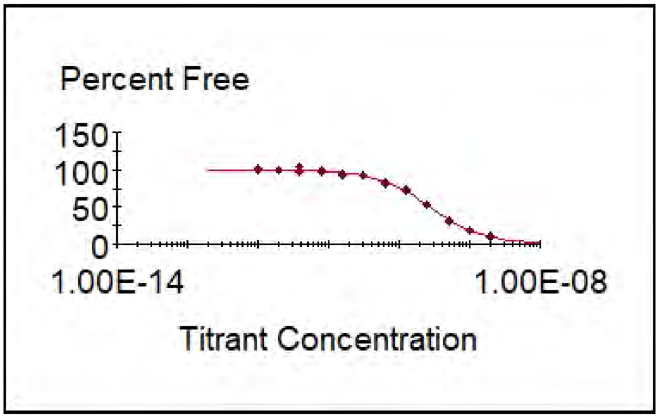

**Kd:**	189.63pM
**95% confidence interval**	
**Kd High:**	497.81pM
**Kd Low:**	99.31pM

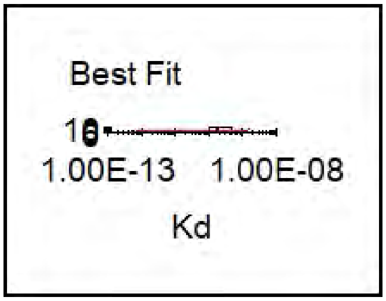

**Active CBP:**	159.65pM
**CBP %Activity:**	39.91
**95% confidence interval**	
**CBP High:**	335.11pM
**%Activity:**	83.78
**CBP Low:**	Less than 576.76fM
**%Activity:**	Less than 0.14

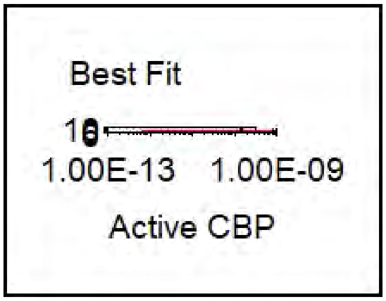

Data Traces(x)

Cycles: 2
Incubation delay (min): 300
Mix Time:

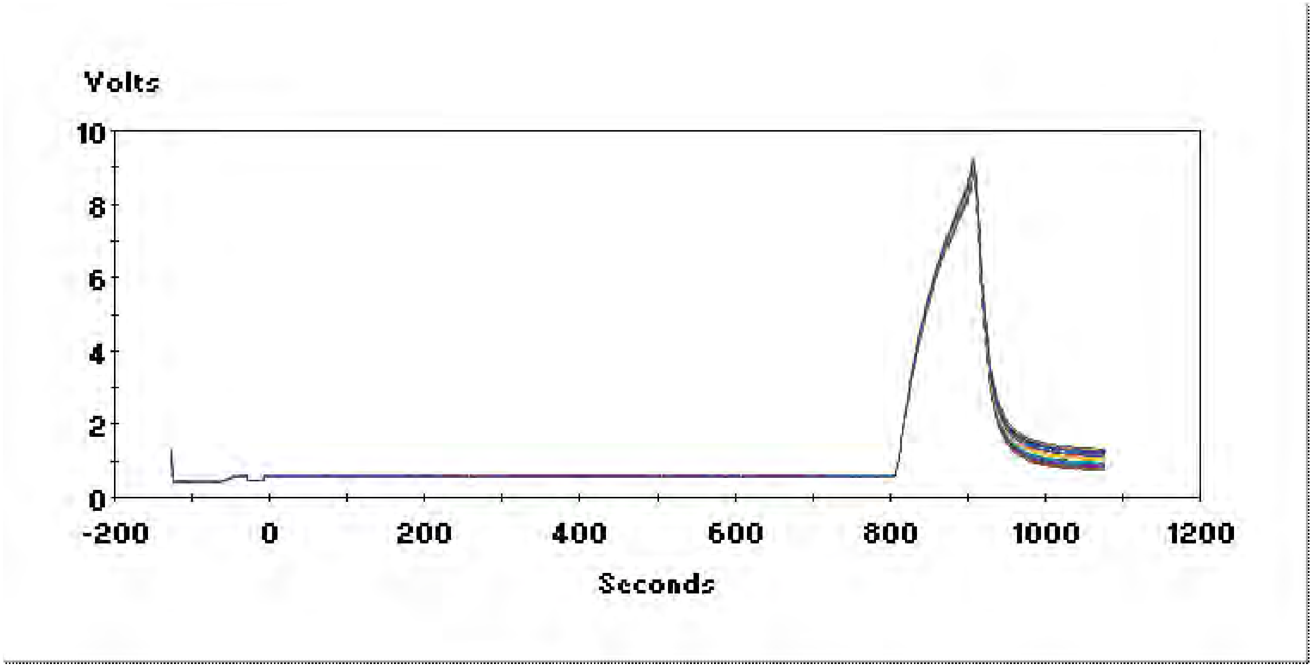

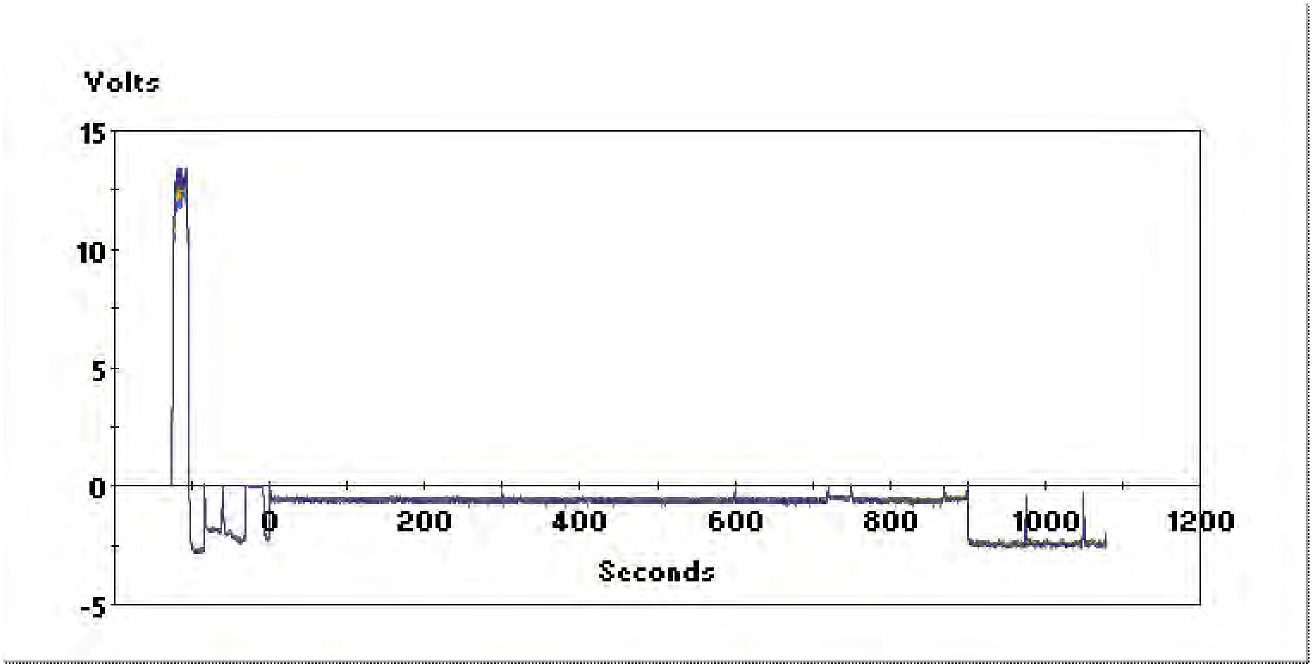

Experiment(x)

**Experiment Name:**	KD 6F5.4 vs 6F5.4-specific F5 LCHN 022120	**Start Time:**	Thu Feb 20 16:34:54 2020
**Experiment Type:**	Equilibrium	**End Time:**	Fri Feb 21 11:15:45 2020
**Constant Binding Partner (CBP)**		**Buffer:**	PBS/BSA
**Molecular Concentration:**	30.00pM	**Label:**	aHis-647
**Valency:**	1	**Label Conc:**	0
**Binding Site Concentration:**	30.00pM		

Comments(x)

beads: 6F5.4 2/14/20

sample volume: 6 ml

detection: aHis-647 (Southern Biotech 4603-31)

CBP: 30 pM 6F5.4-specific F5 LCHN

titrant: 6F5.4 IgG 5/12/14 1 mg/ml (thawed 02/12/20)

titration: 2 cycles, 15 samples: 2 nM - 122 fM

samples:

1) NSB

2-15) titration

Timing(x)

Bead Handling (Custom Beads)

Draw Source	Time (sec)	Volume (uL)	Rate (mL/min)	Stir
Backflush	20	0	0.0000	
Buffer	20	500	1.5000	✓
Particle Reservoir 1	20	333	1.0000	✓
Buffer	30	500	1.0000	
Waste	2	8	0.2500	
Buffer	20	0	0.0000	
Buffer	9	150	1.0000	

Sample Timing

Draw Source	Time (sec)	Volume (uL)	Rate (mL/min)	Time Stamp
Sample Set 1,201-215	1440	6000	0.2500	
Buffer	30	125	0.2500	
Standards: Tube 3	120	500	0.2500	
Buffer	30	125	0.2500	
Buffer	120	2000	1.0000	

Analysis(x)

Baseline / Endpoints:

to (sec) from beginning

to (sec) from end

Ignore	Binding Signal (V)	Concentration
✓	0.0904	NSB
	0.0911	2.00nM
	0.0829	1.00nM
	0.1024	500.00pM
	0.0928	250.00pM
	0.1118	125.00pM
	0.1678	62.50pM
	0.4269	31.25pM
	0.8089	15.63pM
	1.0754	7.81pM
	1.2054	3.91pM
	1.3075	1.95pM
	1.3320	976.56fM
	1.3609	488.28fM
	1.3491	244.14fM
	1.3794	122.07fM
✓	0.0974	NSB
	0.1026	2.00nM
	0.1025	1.00nM
	0.0973	500.00pM
	0.1066	250.00pM
	0.1078	125.00pM
	0.1434	62.50pM
	0.3522	31.25pM
	0.7636	15.63pM
	1.0379	7.81pM
	1.1945	3.91pM
	1.2988	1.95pM
	1.3307	976.56fM
	1.3569	488.28fM
	1.3579	244.14fM
	1.3820	122.07fM

**Kd:**	2.40pM
**Active CBP:**	29.80pM
**CBP %Activity:**	99.32
**Ratio:**	12.4208
**Sig 100%:**	1.37
**NSB:**	0.09
**%Error:**	1.23

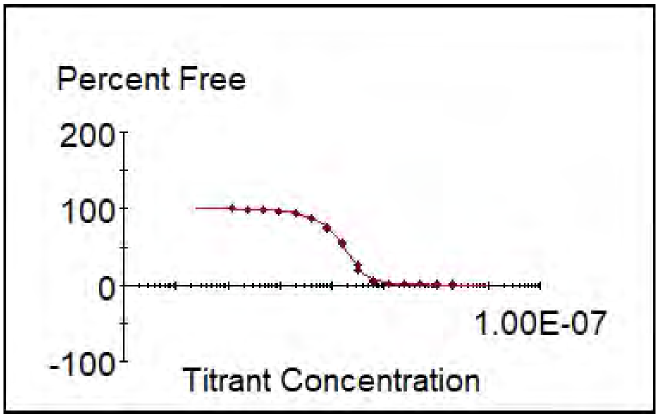

**Kd:**	2.40pM
**95% confidence interval**	
**Kd High:**	3.52pM
**Kd Low:**	1.48pM

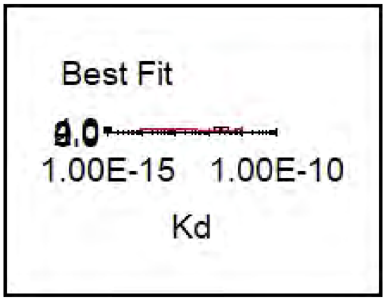

**Active CBP:**	29.80pM
**CBP %Activity:**	99.32
**95% confidence interval**	
**CBP High:**	33.92pM
**%Activity:**	113.08
**CBP Low:**	25.56pM
**%Activity:**	85.20

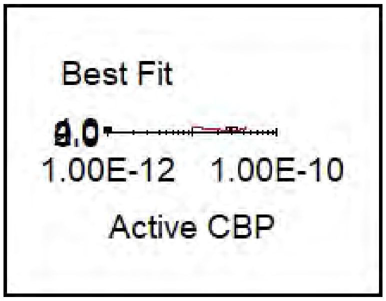

Data Traces(x)

Cycles: 2
Incubation delay (min): 0
Mix Time:

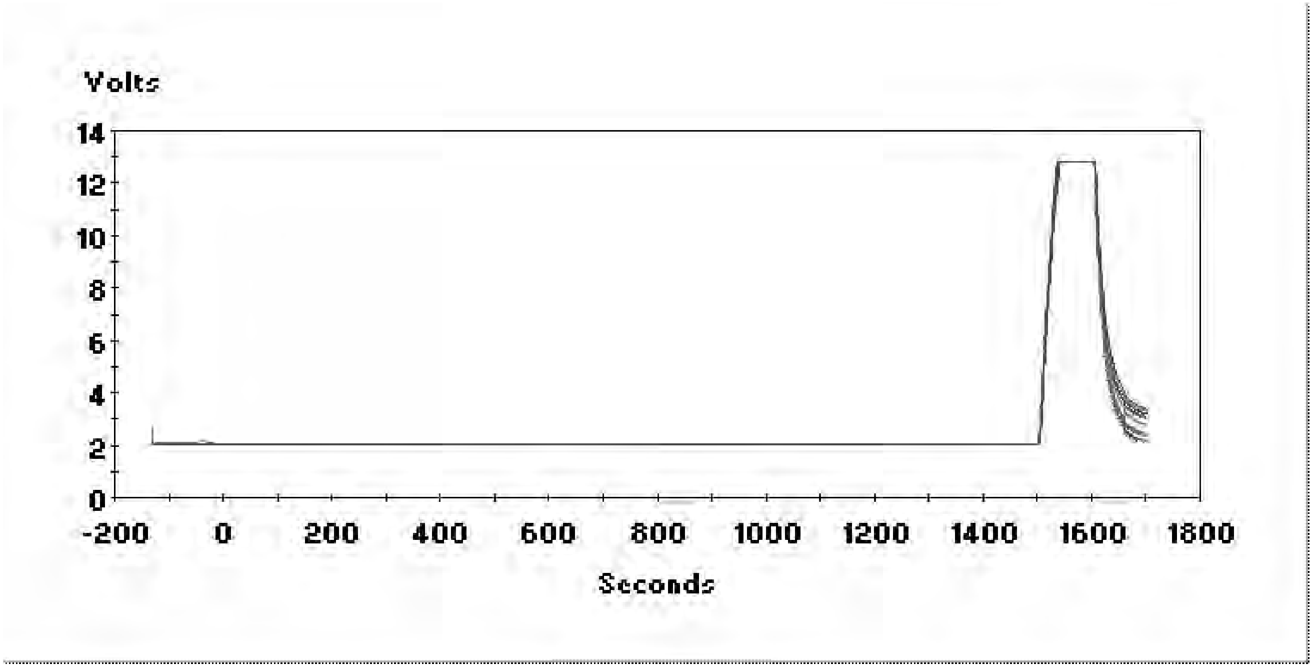

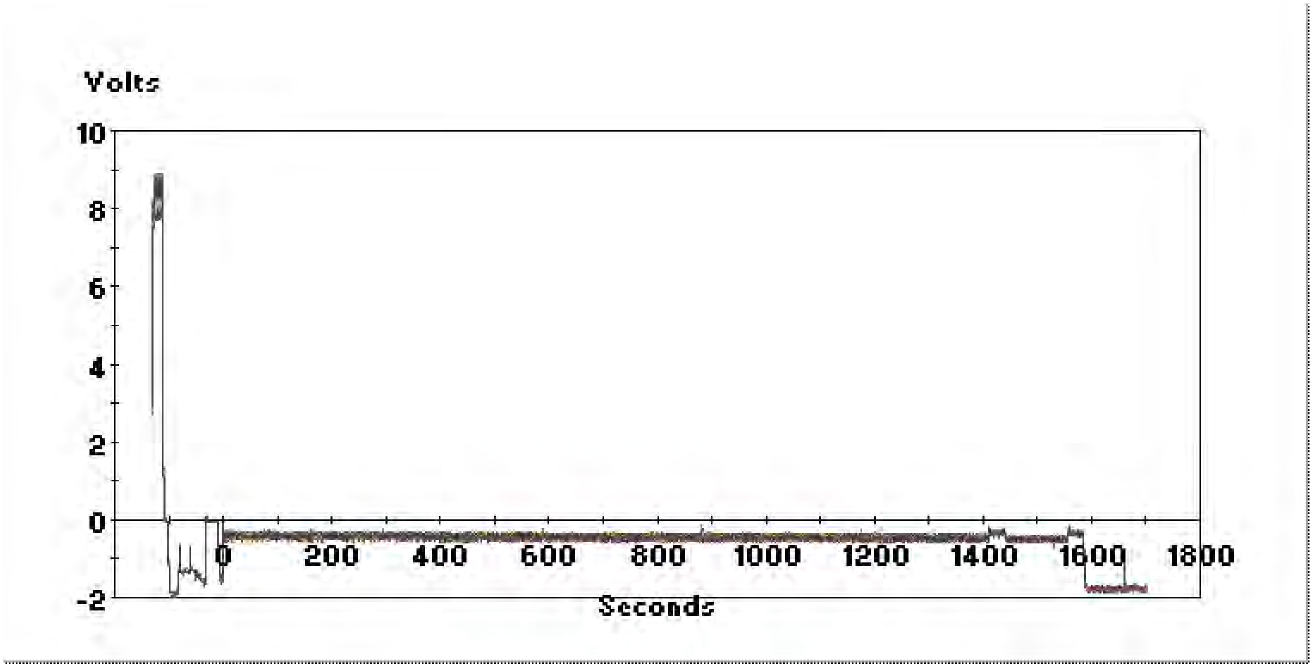

Experiment(x)

**Experiment Name:**	KD TeAb-F IgG1& F1 Toxin(6F5.4) 090324	**Start Time:**	Tue Sep 3 23:18:57 2024
**Experiment Type:**	Equilibrium	**End Time:**	Wed Sep 4 09:54:58 2024
**Constant Binding Partner (CBP)**		**Buffer:**	PBS/BSA
**Molecular Concentration:**	200.00pM	**Label:**	Anti-Hu6F15.4&Hu6F10-647
**Valency:**	1	**Label Conc:**	0
**Binding Site Concentration:**	200.00pM		

Comments(x)

beads:6F5.4 IgG1 100ug on 1ml Sepharose 4B

sample volume: 6 ml

detection: Hu6F15.4 &Hu6F10 IgG1-647 (prepared by Lou 090324).1:800

CBP: 200 pM BoNT/F1 082724

titrant: TeAb-F IgG1

titration: 2 cycles, 14 samples: 200pM - 98fM (1:2) of TeAb-F IgG1, plus NBS & 200pM BoNT-F1 only ctr

samples:

1-14) titration

1): NBS

2): 200 pM BoNT/F1 only ctr

3 ~ 14): 200pM - 98fM (1:2) of TeAb-F IgG1, Incubate 1hr RT and O/N 4 C

beads:6F5.4 IgG1 100ug on 1ml Sepharose 4B

sample volume: 6 ml

detection: Hu6F15.4 &Hu6F10 IgG1-647 (prepared by Lou 090324).1:800

CBP: 200 pM BoNT/F1 082724

titrant: TeAb-F IgG1

titration: 2 cycles, 14 samples: 200pM - 98fM (1:2) of TeAb-F IgG1, plus NBS & 200pM BoNT-F1 only ctr

samples:

1-14) titration

1): NBS

2): 200 pM BoNT/F1 only ctr

3 ~ 14): 200pM - 98fM (1:2) of TeAb-F IgG1, Incubate 1hr RT and O/N 4 C

Timing(x)

Bead Handling (Custom Beads)

Draw Source	Time (sec)	Volume (uL)	Rate (mL/min)	Stir
Backflush	20	0	0.0000	
Buffer	20	500	1.5000	✓
Particle Reservoir 1	23	380	1.0000	✓
Buffer	30	500	1.0000	
Waste	2	8	0.2500	
Buffer	20	0	0.0000	
Buffer	9	150	1.0000	

Sample Timing

Draw Source	Time (sec)	Volume (uL)	Rate (mL/min)	Time Stamp
Sample Set 201-214	672	2800	0.2500	
Buffer	30	125	0.2500	
Rack 1: Tube 21	120	500	0.2500	
Buffer	30	125	0.2500	
Buffer	180	3000	1.0000	

Analysis (x)

Baseline / Endpoints:
to (sec) from beginning
to (sec) from end

Ignore	Binding Signal (V)	Concentration
✓	0.1119	NSB
	0.3073	0
	0.1565	200.00pM
	0.1830	100.00pM
	0.1943	50.00pM
	0.2418	25.00pM
	0.2617	12.50pM
	0.2577	6.25pM
	0.2687	3.13pM
	0.2851	1.56pM
	0.2753	781.25fM
✓	0.2525	390.63fM
	0.2796	195.31fM
✓	0.2596	97.66fM
	0.1035	NSB
✓	0.2507	0
	0.1017	200.00pM
	0.1527	100.00pM
	0.2004	50.00pM
	0.2211	25.00pM
	0.2401	12.50pM
	0.2427	6.25pM
	0.2715	3.13pM
✓	0.2431	1.56pM
	0.2571	781.25fM
	0.2560	390.63fM
	0.2479	195.31fM
✓	0.2463	97.66fM

**Kd:**	26.60pM
**Active CBP:**	59.08pM
**CBP %Activity:**	29.54
**Ratio:**	2.2209
**Sig 100%:**	0.27
**Drift (%/run):**	-0.4572
**NSB:**	0.10
**Drift (mV/run):**	-2.2838
**%Error:**	4.77

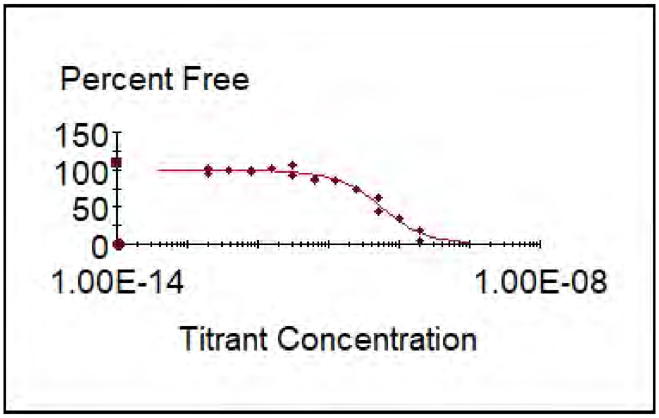

**Kd:**	26.60pM
**95% confidence interval**	
**Kd High:**	68.91pM
**Kd Low:**	5.44pM

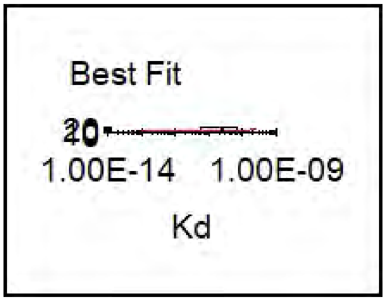

**Active CBP:**	59.08pM
**CBP %Activity:**	29.54
**95% confidence interval**	
**CBP High:**	131.66pM
**%Activity:**	65.83
**CBP Low:**	Less than 213.45fM
**%Activity:**	Less than 0.11

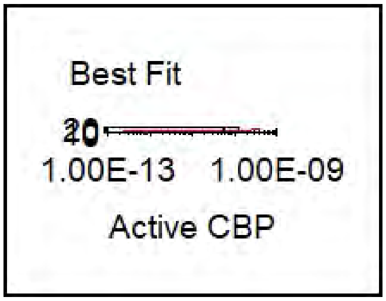

Data Traces(x)

Cycles: 2
Incubation delay (min): 0
Mix Time:

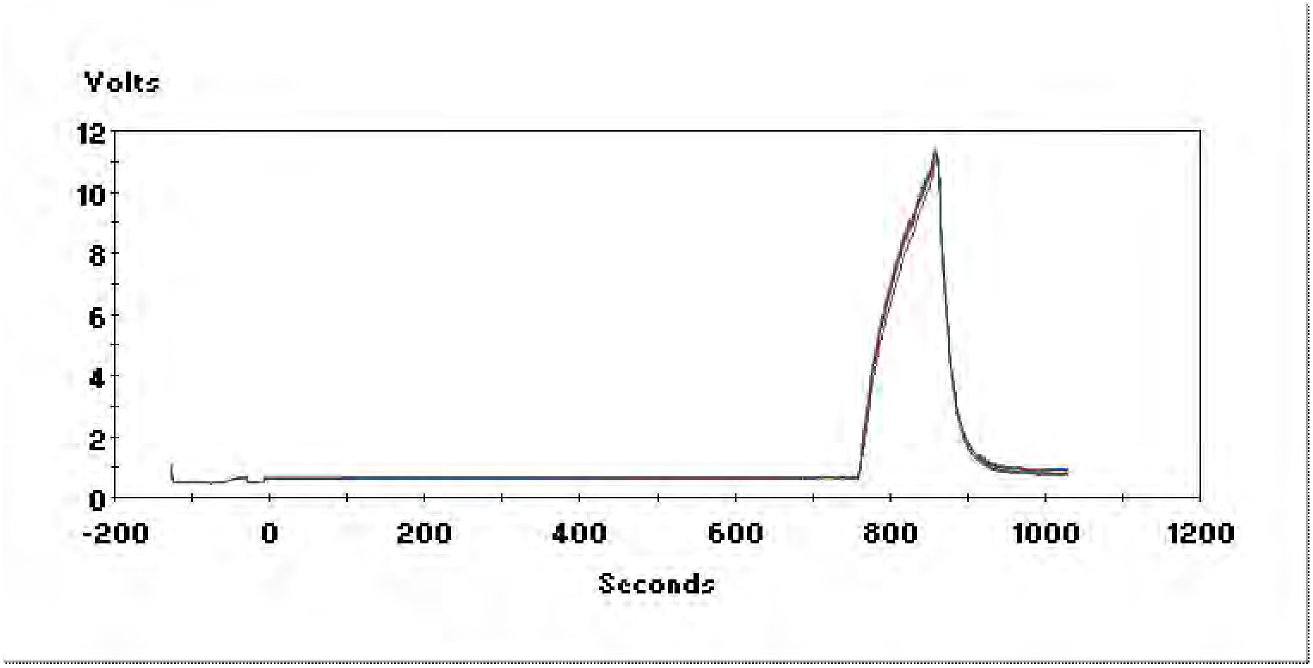

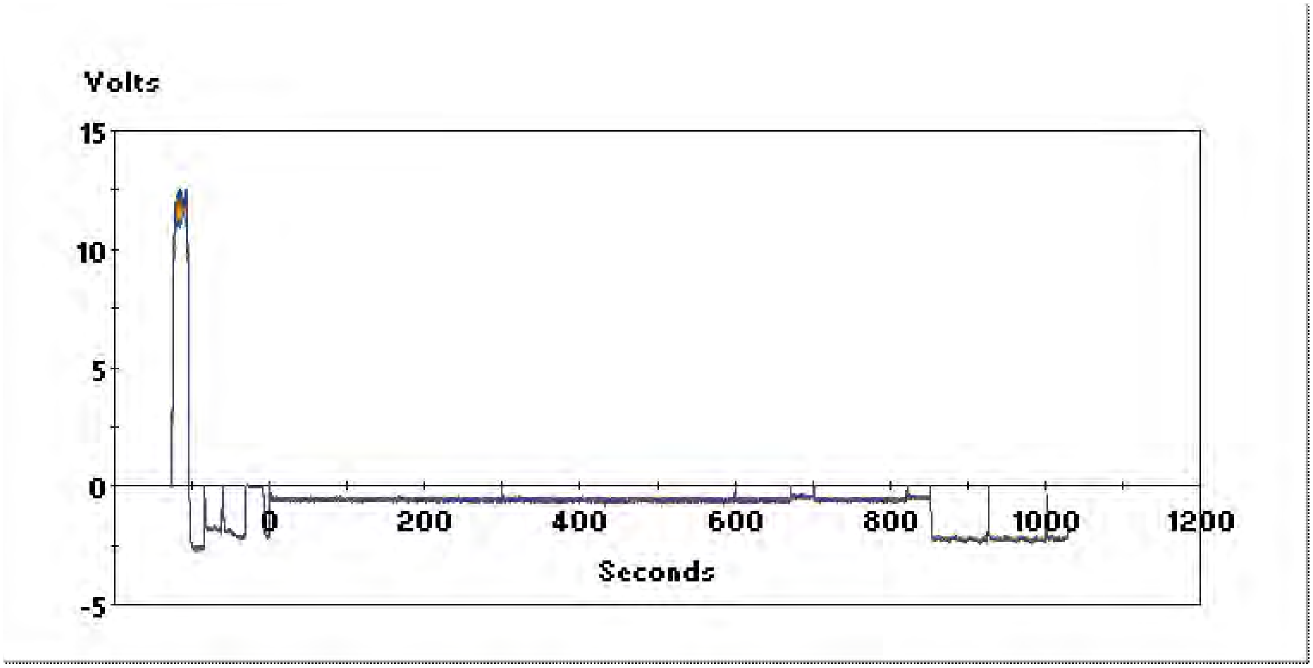

Experiment(x)

**Experiment Name:**	KD 6F11V0 vs BoNT F1 081820	**Start Time:**	Fri Aug 14 16:38:57 2020
**Experiment Type:**	Equilibrium	**End Time:**	Sat Aug 15 20:24:28 2020
**Constant Binding Partner (CBP)**		**Buffer:**	PBS/BSA
**Molecular Concentration:**	10.00pM	**Label:**	6F5.4-647
**Valency:**	1	**Label Conc:**	0
**Binding Site Concentration:**	10.00pM		

Comments(x)

beads: 6F11 8/14/20

sample volume: 12 ml

detection: 6F5.4-647

CBP: 10 pM BoNT F1 100453 8/11/20

titrant: Ology 6F11V0 IgG 6/8/20 5.34 mg/ml

titration: 2 cycles, 13 samples: 200 pM - 48.8 fM

samples:

1) NSB

2-13) titration

Timing(x)

Bead Handling (Custom Beads)

Draw Source	Time (sec)	Volume (uL)	Rate (mL/min)	Stir
Backflush	20	0	0.0000	
Buffer	20	500	1.5000	✓
Particle Reservoir 1	22	367	1.0000	✓
Buffer	30	500	1.0000	
Waste	2	8	0.2500	
Buffer	20	0	0.0000	
Buffer	9	150	1.0000	

Sample Timing

Draw Source	Time (sec)	Volume (uL)	Rate (mL/min)	Time Stamp
Sample Set 1,101-113	2880	12000	0.2500	
Buffer	30	125	0.2500	
Standards: Tube 3	120	500	0.2500	
Buffer	30	125	0.2500	
Buffer	120	2000	1.0000	

Analysis(x)

Baseline / Endpoints:

to (sec) from beginning

to (sec) from end

Ignore	Binding Signal (V)	Concentration
✓	0.1301	NSB
	0.1287	200.00pM
	0.1338	100.00pM
	0.1318	50.00pM
	0.1268	25.00pM
	0.1684	12.50pM
	0.3191	6.25pM
	0.4484	3.13pM
	0.5583	1.56pM
	0.5908	781.25fM
	0.6206	390.63fM
	0.6184	195.31fM
	0.6210	97.66fM
	0.6195	48.83fM
✓	0.1417	NSB
	0.1268	200.00pM
	0.1515	100.00pM
	0.1227	50.00pM
	0.1289	25.00pM
	0.1453	12.50pM
	0.2720	6.25pM
	0.4164	3.13pM
	0.5247	1.56pM
	0.5761	781.25fM
	0.6081	390.63fM
	0.6242	195.31fM
	0.6347	97.66fM
	0.6283	48.83fM

**Kd:**	347.90fM
**Active CBP:**	8.03pM
**CBP % Activity:**	80.31
**Ratio:**	23.0835
**Sig 100%:**	0.63
**NSB:**	0.13
**%Error:**	2.48

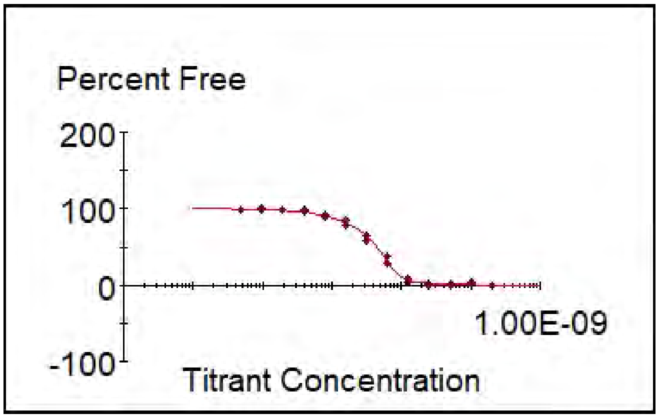

**Kd:**	347.90fM
**95% confidence interval**	
**Kd High:**	873.57fM
**Kd Low:**	21.99fM

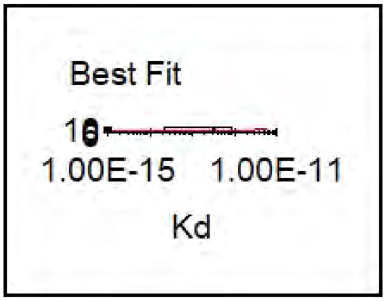

**Active CBP:**	8.03pM
**CBP %Activity:**	80.31
**95% confidence interval**	
**CBP High:**	9.53pM
**%Activity:**	95.28
**CBP Low:**	6.04pM
**%Activity:**	60.43

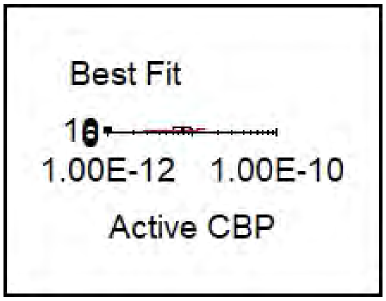

Data Traces(x)

Cycles: 2
Incubation delay (min): 0
Mix Time:

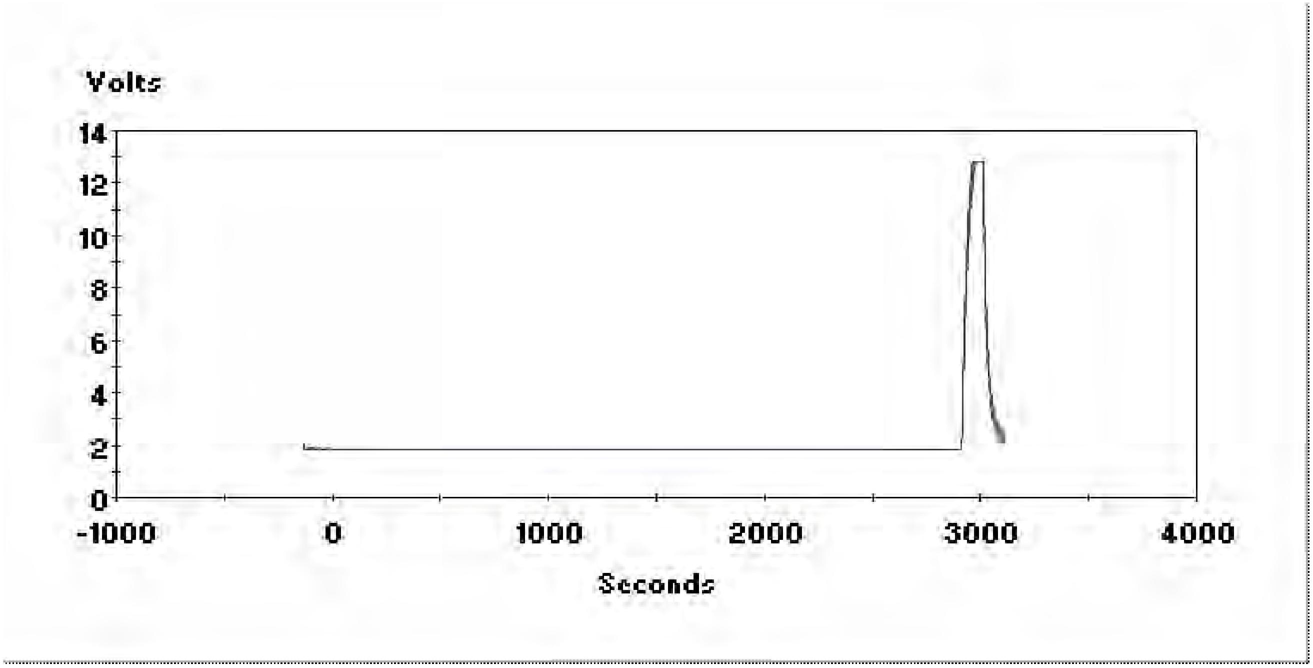

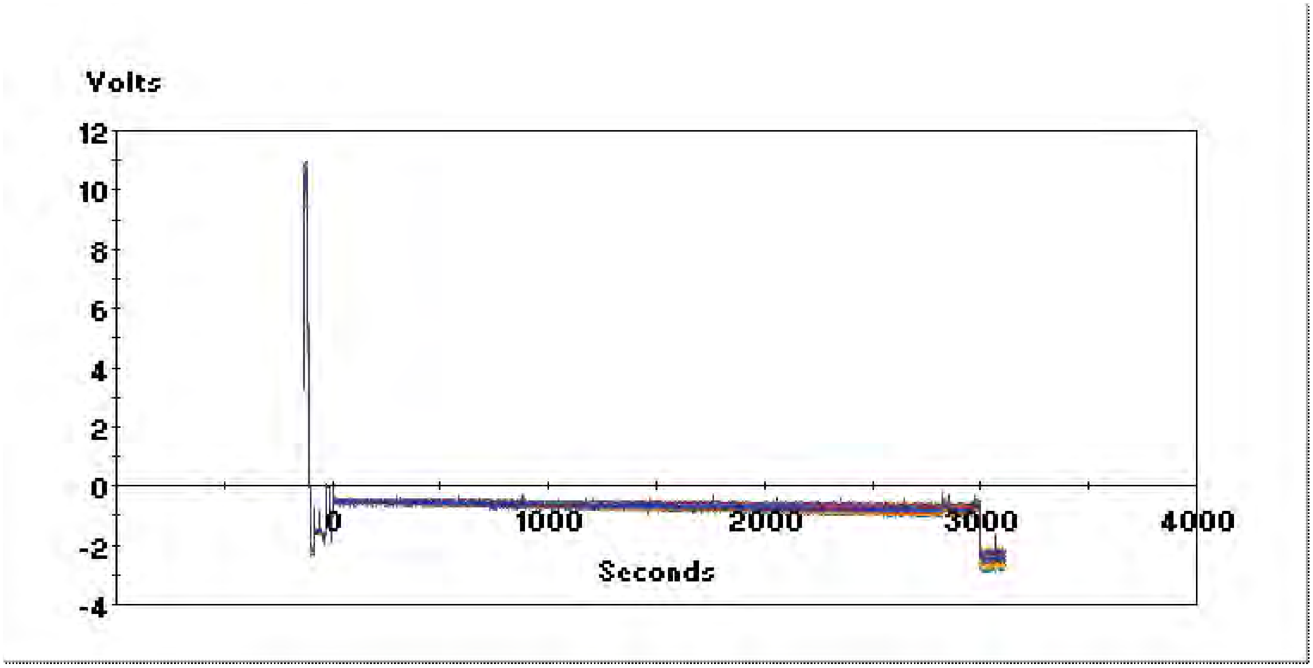

Experiment(x)

**Experiment Name:**	KD Hu6F11 IgG1&NXF11 domain 081424	**Start Time:**	Wed Aug 14 23:25:22 2024
**Experiment Type:**	Equilibrium	**End Time:**	Thu Aug 15 08:11:04 2024
**Constant Binding Partner (CBP)**		**Buffer:**	PBS/BSA
**Molecular Concentration:**	500.00pM	**Label:**	Anti-His tag-647
**Valency:**	1	**Label Conc:**	0
**Binding Site Concentration:**	500.00pM		

Comments(x)

beads: Hu6F11 IgG1 100ug on 1ml Sepharose 4B

sample volume: 7 ml

detection: Anti-His-647 (prepared by Lou 081424).1:800

CBP: 500 pM NXF11 domain 081424

titrant: Hu6F11 IgG1

titration: 2 cycles, 15 samples: 4nM - 976 fM (1:2) of Hu6F11 IgG1, plus NBS & 1nM NXF11 domain only ctr

samples:

1-15) titration

1): NBS

2): 500pM NXF11 only

3 ~ 15): 4nM - 976 fM (1:2) of Hu6F11 IgG1, Incubate 3hr RT & 4hr at 4 C

beads: Hu6F11 IgG1 100ug on 1ml Sepharose 4B

sample volume: 7 ml

detection: Anti-His-647 (prepared by Lou 081424).1:800

CBP: 500 pM NXF11 domain 081424

titrant: Hu6F11 IgG1

titration: 2 cycles, 15 samples: 4nM - 976 fM (1:2) of Hu6F11 IgG1, plus NBS & 1nM NXF11 domain only ctr

samples:

1-15) titration

1): NBS

2): 500pM NXF11 only

3 ~ 15): 4nM - 976 fM (1:2) of Hu6F11 IgG1, Incubate 3hr RT & 4hr at 4 C

Timing(x)

Bead Handling (Custom Beads)

Draw Source	Time (sec)	Volume (uL)	Rate (mL/min)	Stir
Backflush	20	0	0.0000	
Buffer	20	500	1.5000	✓
Particle Reservoir 1	24	400	1.0000	✓
Buffer	30	500	1.0000	
Waste	2	8	0.2500	
Buffer	20	0	0.0000	
Buffer	9	150	1.0000	

Sample Timing

Draw Source	Time (sec)	Volume (uL)	Rate (mL/min)	Time Stamp
Sample Set 201-215	360	3000	0.5000	
Buffer	30	125	0.2500	
Rack 1: Tube 21	120	500	0.2500	
Buffer	30	125	0.2500	
Buffer	180	3000	1.0000	

Analysis(x)

Baseline / Endpoints:
to (sec) from beginning
to (sec) from end

Ignore	Binding Signal (V)	Concentration
	0.0135	NSB
	0.4040	0
✓	0.1160	4.00nM
	0.0158	2.00nM
	0.0249	1.00nM
	0.0460	500.00pM
	0.1454	250.00pM
	0.2581	125.00pM
	0.3127	62.50pM
	0.3486	31.25pM
	0.3702	15.63pM
	0.3690	7.81pM
	0.3696	3.91pM
	0.3880	1.95pM
	0.3855	976.56fM
	0.0081	NSB
	0.3669	0
	0.0221	4.00nM
	0.0122	2.00nM
	0.0188	1.00nM
	0.0420	500.00pM
	0.1305	250.00pM
	0.2371	125.00pM
	0.2894	62.50pM
	0.3273	31.25pM
	0.3441	15.63pM
	0.3528	7.81pM
	0.3606	3.91pM
	0.3574	1.95pM
	0.3739	976.56fM

**Kd:**	25.10pM
**Active CBP:**	308.48pM
**CBP %Activity:**	61.70
**Ratio:**	12.2888
**Sig 100%:**	0.38
**Drift (%/run):**	0.2600
**NSB:**	0.01
**Drift (mV/run):**	-0.3954
**TR NSB:**	2.74e+06
**%Error:**	1.52

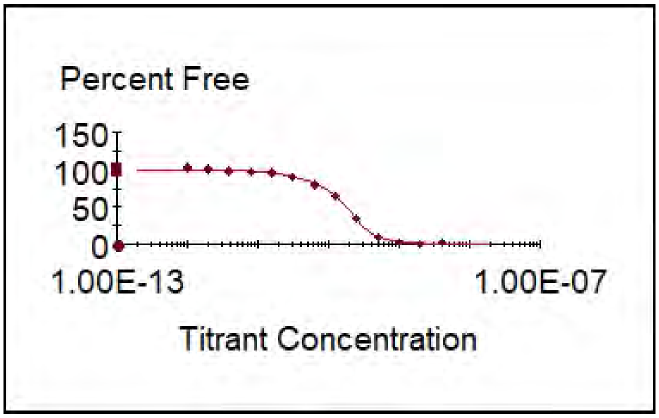

**Kd:**	25.10pM
**95% confidence interval**	
**Kd High:**	41.25pM
**Kd Low:**	12.66pM

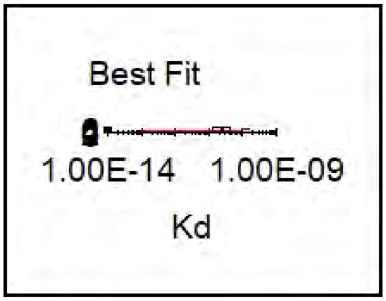

**Active CBP:**	308.48pM
**CBP %Activity:**	61.70
**95% confidence interval**	
**CBP High:**	354.45pM
**%Activity:**	70.89
**CBP Low:**	254.89pM
**%Activity:**	50.98

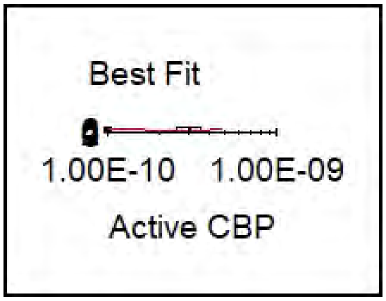

Data Traces(x)

Cycles: 2
Incubation delay (min): 120
Mix Time:

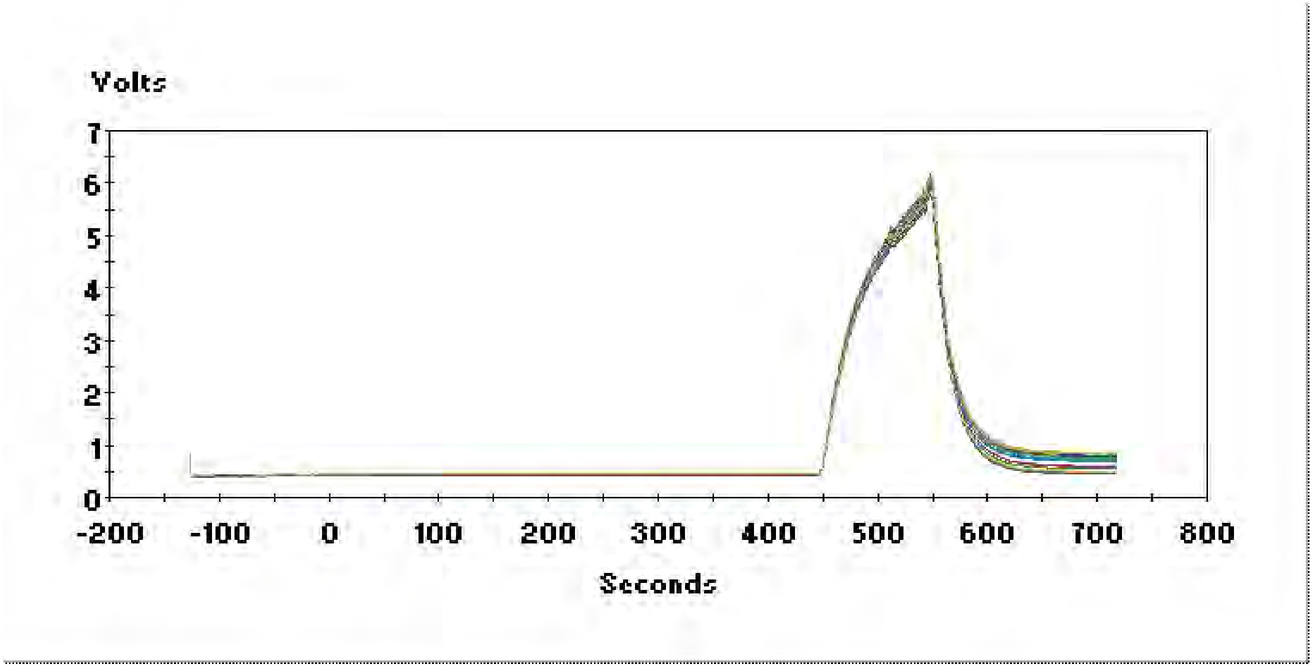

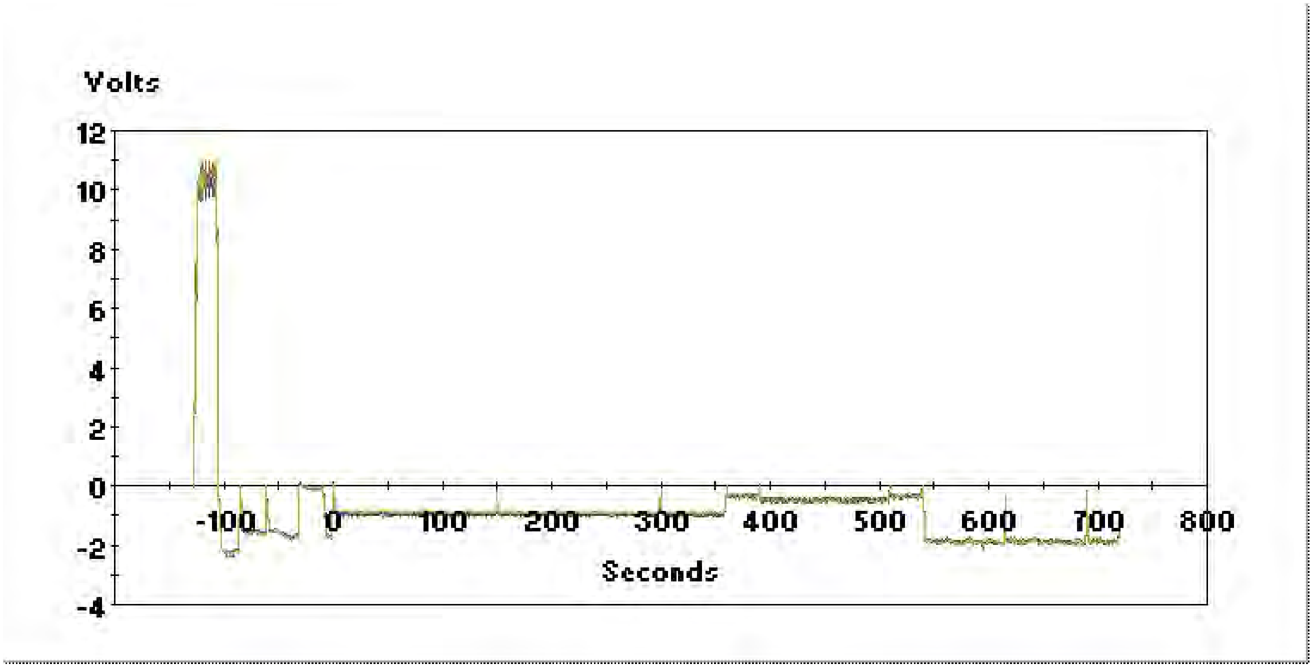

Experiment(x)

**Experiment Name:**	KD Hu6F13.4 IgG1& F1 Toxin 090324	**Start Time:**	Tue Sep 3 12:16:46 2024
**Experiment Type:**	Equilibrium	**End Time:**	Tue Sep 3 23:15:36 2024
**Constant Binding Partner (CBP)**		**Buffer:**	PBS/BSA
**Molecular Concentration:**	200.00pM	**Label:**	Anti-Hu6F15.4&Hu6F10-647
**Valency:**	2	**Label Conc:**	0
**Binding Site Concentration:**	400.00pM		

Comments(x)

beads:Hu6F13.4 IgG1 100ug on 1ml Sepharose 4B

sample volume: 7 ml

detection: Hu6F15.4 &Hu6F10 IgG1-647 (prepared by Lou 090324).1:800

CBP: 200 pM BoNT/F1 082724

titrant: TeAb-F IgG1

titration: 2 cycles, 14 samples: 200pM - 98fM (1:2) of TeAb-F IgG1, plus NBS & 200pM BoNT-F1 only ctr

samples:

1-14) titration

1): NBS

2): 200 pM BoNT/F1 only ctr

3 ~ 14): 200pM - 98fM (1:2) of TeAb-F IgG1, Incubate 1hr RT and O/N 4 C

beads:Hu6F13.4 IgG1 100ug on 1ml Sepharose 4B

sample volume: 7 ml

detection: Hu6F15.4 &Hu6F10 IgG1-647 (prepared by Lou 090324).1:800

CBP: 200 pM BoNT/F1 082724

titrant: TeAb-F IgG1

titration: 2 cycles, 14 samples: 200pM - 98fM (1:2) of TeAb-F IgG1, plus NBS & 200pM BoNT-F1 only ctr

samples:

1-14) titration

1): NBS

2): 200 pM BoNT/F1 only ctr

3 ~ 14): 200pM - 98fM (1:2) of TeAb-F IgG1, Incubate 1hr RT and O/N 4 C

Timing(x)

Bead Handling (Custom Beads)

Draw Source	Time (sec)	Volume (uL)	Rate (mL/min)	Stir
Backflush	20	0	0.0000	
Buffer	20	500	1.5000	✓
Particle Reservoir 1	23	380	1.0000	✓
Buffer	30	500	1.0000	
Waste	2	8	0.2500	
Buffer	20	0	0.0000	
Buffer	9	150	1.0000	

Sample Timing

Draw Source	Time (sec)	Volume (uL)	Rate (mL/min)	Time Stamp
Sample Set 201-214	720	3000	0.2500	
Buffer	30	125	0.2500	
Rack 1: Tube 21	120	500	0.2500	
Buffer	30	125	0.2500	
Buffer	180	3000	1.0000	

Analysis(x)

Baseline / Endpoints:
to (sec) from beginning
to (sec) from end

Ignore	Binding Signal (V)	Concentration
✓	0.1371	NSB
✓	0.5938	0
	0.3170	200.00pM
	0.3693	100.00pM
	0.4088	50.00pM
	0.4731	25.00pM
	0.5003	12.50pM
	0.5372	6.25pM
	0.5392	3.13pM
	0.5258	1.56pM
	0.5573	781.25fM
	0.5052	390.63fM
	0.5417	195.31fM
	0.5045	97.66fM
	0.1102	NSB
	0.4991	0
	0.2713	200.00pM
	0.3237	100.00pM
	0.3898	50.00pM
	0.4317	25.00pM
	0.4718	12.50pM
	0.4721	6.25pM
	0.4850	3.13pM
	0.4783	1.56pM
	0.4979	781.25fM
	0.5033	390.63fM
	0.4981	195.31fM
	0.4801	97.66fM

**Kd:**	80.47pM
**Active CBP:**	28.84pM
**CBP %Activity:**	7.21
**Ratio:**	0.3584
**Sig 100%:**	0.52
**Drift (%/run):**	0.0000
**NSB:**	0.11
**Drift (mV/run):**	-2.8387
**TR NSB:**	2.16e+08
**%Error:**	2.81

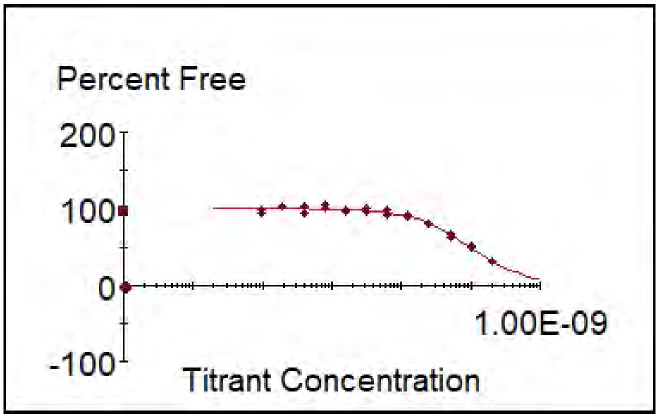

**Kd:**	80.47pM
**95% confidence interval**	
**Kd High:**	139.24pM
**Kd Low:**	13.40pM

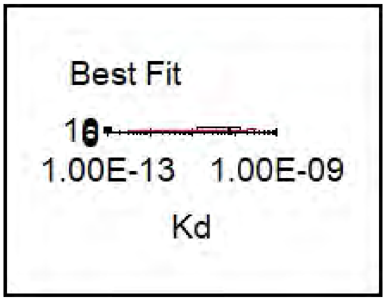

**Active CBP:**	28.84pM
**CBP %Activity:**	7.21
**95% confidence interval**	
**CBP High:**	137.62pM
**%Activity:**	34.41
**CBP Low:**	Less than 104.19fM
**%Activity:**	Less than 0.03

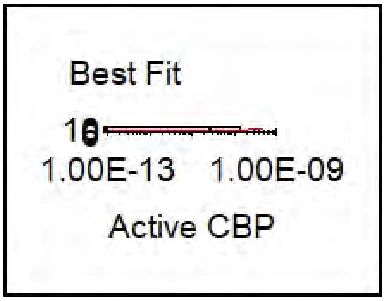

Data Traces(x)

Cycles: 2
Incubation delay (min): 0
Mix Time:

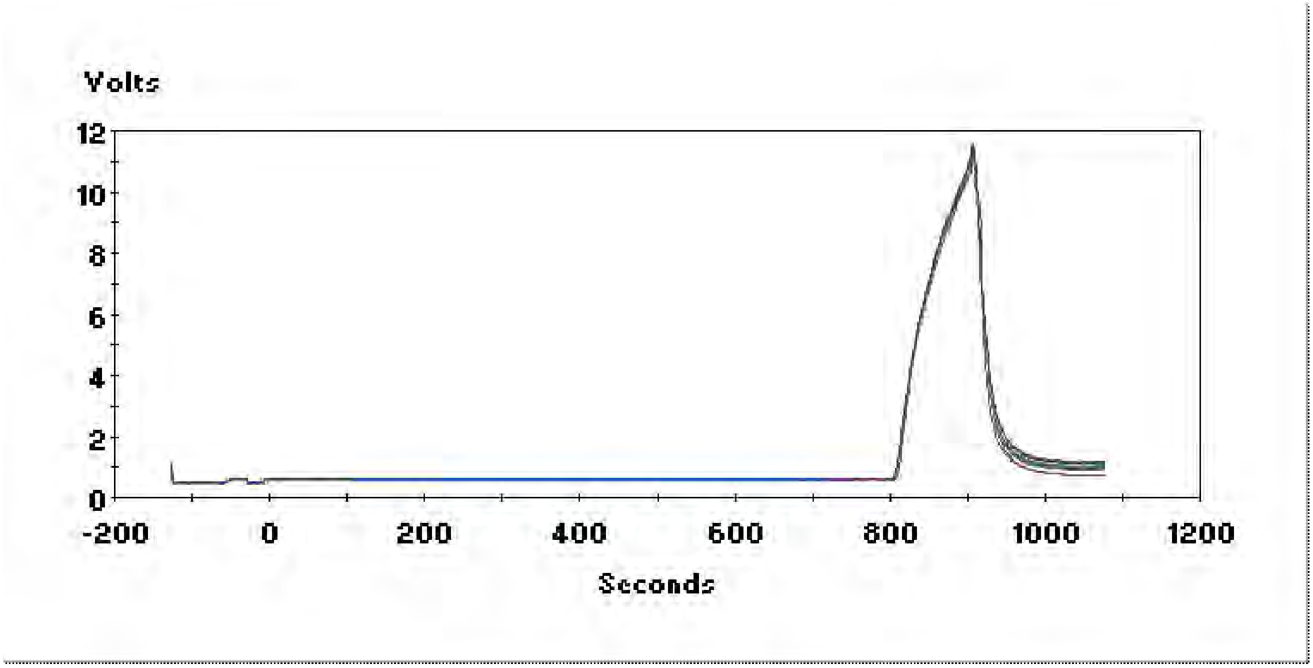

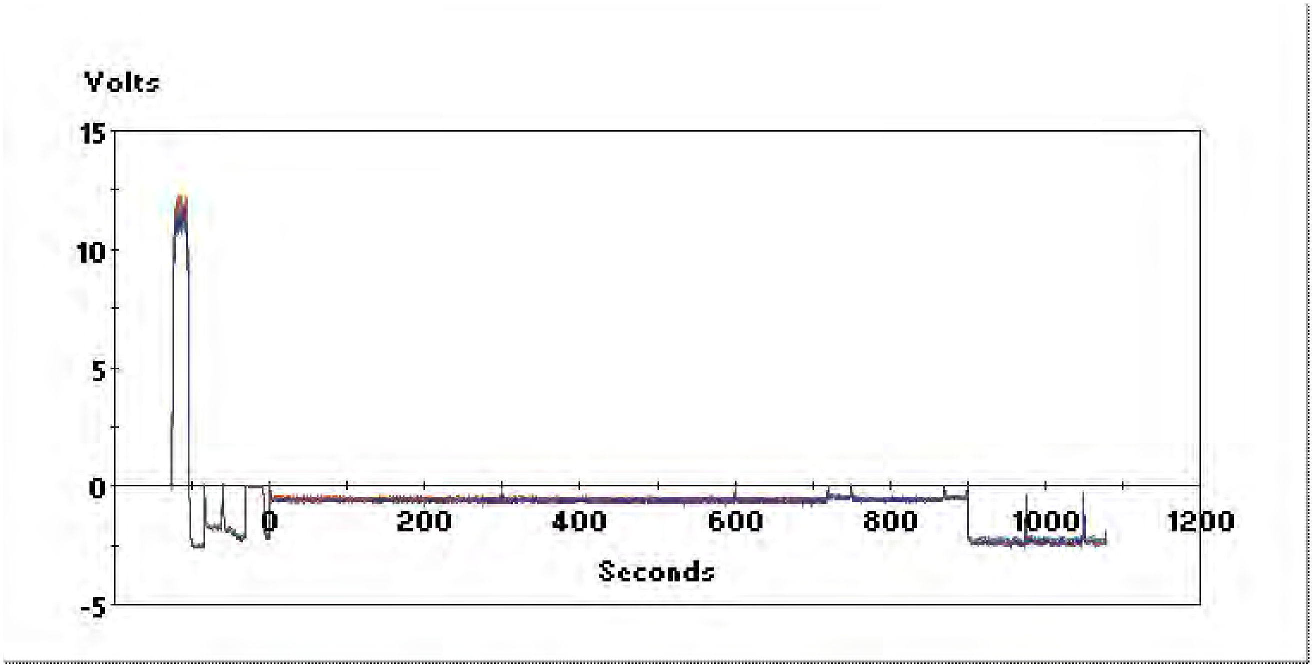

Experiment(x)

**Experiment Name:**	KD Hu6F13.4IgG1&NXF13 domain 081424	**Start Time:**	Wed Aug 14 12:36:57 2024
**Experiment Type:**	Equilibrium	**End Time:**	Wed Aug 14 21:22:38 2024
**Constant Binding Partner (CBP)**		**Buffer:**	PBS/BSA
**Molecular Concentration:**	100.00pM	**Label:**	Anti-His tag-647
**Valency:**	1	**Label Conc:**	0
**Binding Site Concentration:**	100.00pM		

Comments(x)

beads: Hu6F13.4 IgG1 100ug on 1ml Sepharose 4B

sample volume: 7 ml

detection: Anti-His-647 (prepared by Lou 081424).1:800

CBP: 100 pM NXF13 domain 081324

titrant: Hu6F13.4 IgG1

titration: 2 cycles, 15 samples: 640 pM - 156 fM (1:2) of Hu6F13.4 IgG1, plus NBS & 1nM NXF5 domain only ctr

samples:

1-15) titration

1): NBS

2): 1nM NXF13 only

3 ~ 15): 640 pM - 156 fM (1:2) of Hu6F13.4 IgG1, Incubate O/N at 4 C

beads: Hu6F13.4 IgG1 100ug on 1ml Sepharose 4B

sample volume: 7 ml

detection: Anti-His-647 (prepared by Lou 081424).1:800

CBP: 100 pM NXF13 domain 081324

titrant: Hu6F13.4 IgG1

titration: 2 cycles, 15 samples: 640 pM - 156 fM (1:2) of Hu6F13.4 IgG1, plus NBS & 1nM NXF13 domain only ctr

samples:

1-15) titration

1): NBS

2): 1nM NXF13 only

3 ~ 15): 640 pM - 156 fM (1:2) of Hu6F13.4 IgG1, Incubate O/N at 4 C

Timing(x)

Bead Handling (Custom Beads)

Draw Source	Time (sec)	Volume (uL)	Rate (mL/min)	Stir
Backflush	20	0	0.0000	
Buffer	20	500	1.5000	✓
Particle Reservoir 1	24	400	1.0000	✓
Buffer	30	500	1.0000	
Waste	2	8	0.2500	
Buffer	20	0	0.0000	
Buffer	9	150	1.0000	

Sample Timing

Draw Source	Time (sec)	Volume (uL)	Rate (mL/min)	Time Stamp
Sample Set 201-215	360	3000	0.5000	
Buffer	30	125	0.2500	
Rack 1: Tube 21	120	500	0.2500	
Buffer	30	125	0.2500	
Buffer	180	3000	1.0000	

Analysis (x)

Baseline / Endpoints:
to (sec) from beginning
to (sec) from end

Ignore	Binding Signal (V)	Concentration
✓	0.0060	NSB
✓	0.5110	0
	0.0221	640.00pM
✓	0.4434	320.00pM
	0.0558	160.00pM
	0.1323	80.00pM
	0.2094	40.00pM
	0.2934	20.00pM
	0.3471	10.00pM
	0.4318	5.00pM
	0.4196	2.50pM
	0.4518	1.25pM
	0.4300	625.00fM
	0.4453	312.50fM
✓	0.4730	156.25fM
✓	0.0084	NSB
✓	0.4622	0
	0.0226	640.00pM
	0.0301	320.00pM
	0.0499	160.00pM
	0.1027	80.00pM
	0.1937	40.00pM
	0.2576	20.00pM
	0.3300	10.00pM
	0.3480	5.00pM
	0.3842	2.50pM
	0.4124	1.25pM
	0.4319	625.00fM
	0.4383	312.50fM
✓	0.4724	156.25fM

**Kd:**	27.01pM
**Active CBP:**	7.92pM
**CBP %Activity:**	7.92
**Ratio:**	0.2933
**Sig 100%:**	0.45
**Drift (%/run):**	0.2958
**NSB:**	-0.01
**Drift (mV/run):**	-0.7288
**TR NSB:**	3.93e-07
**%Error:**	2.45

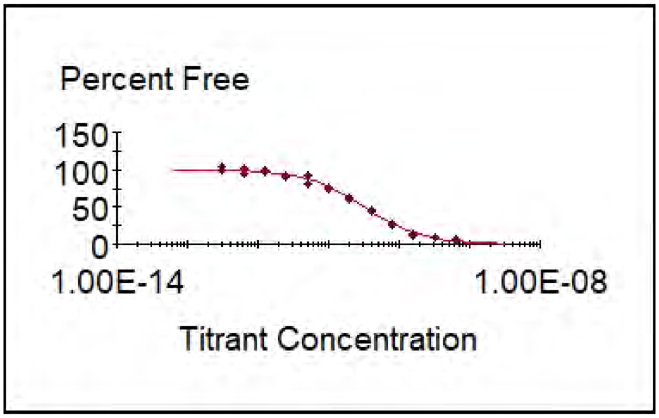

**Kd:**	27.01pM
**95% confidence interval**	
**Kd High:**	49.76pM
**Kd Low:**	14.96pM

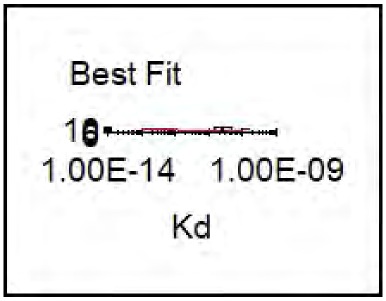

**Active CBP:**	7.92pM
**CBP %Activity:**	7.92
**95% confidence interval**	
**CBP High:**	36.74pM
**%Activity:**	36.74
**CBP Low:**	Less than 28.62fM
**%Activity:**	Less than 0.03

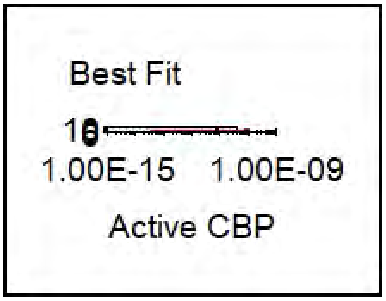

Data Traces(x)

Cycles: 2
Incubation delay (min): 0
Mix Time:

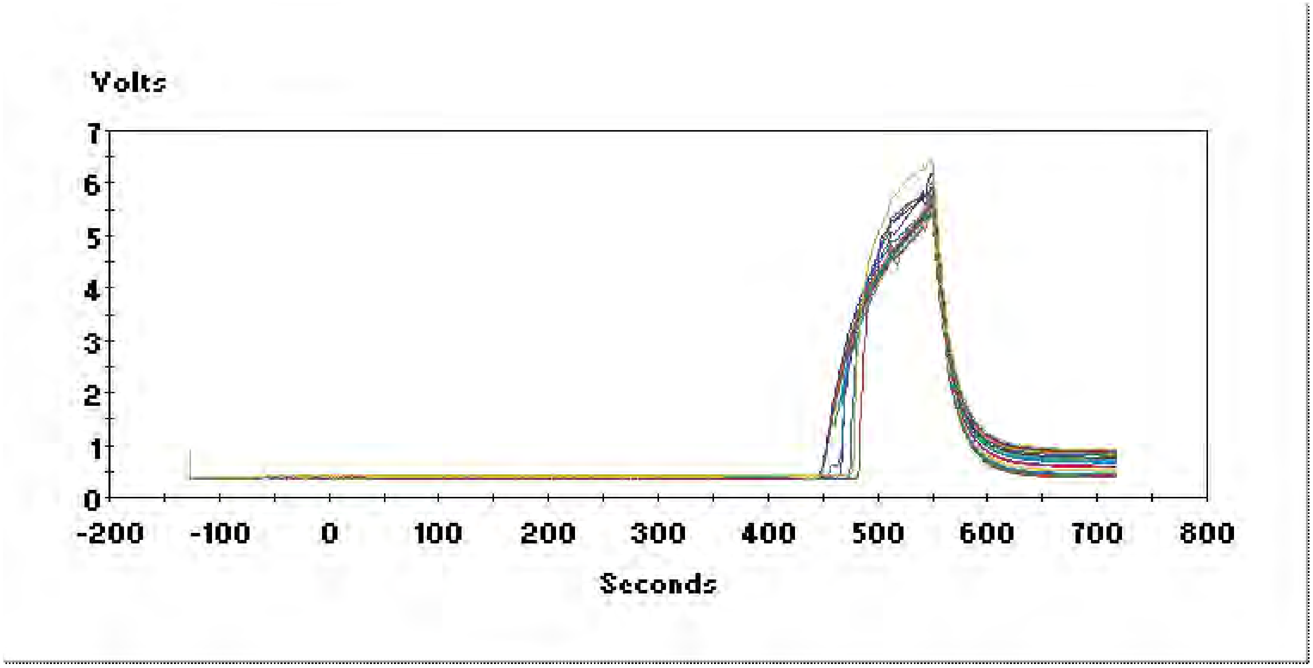

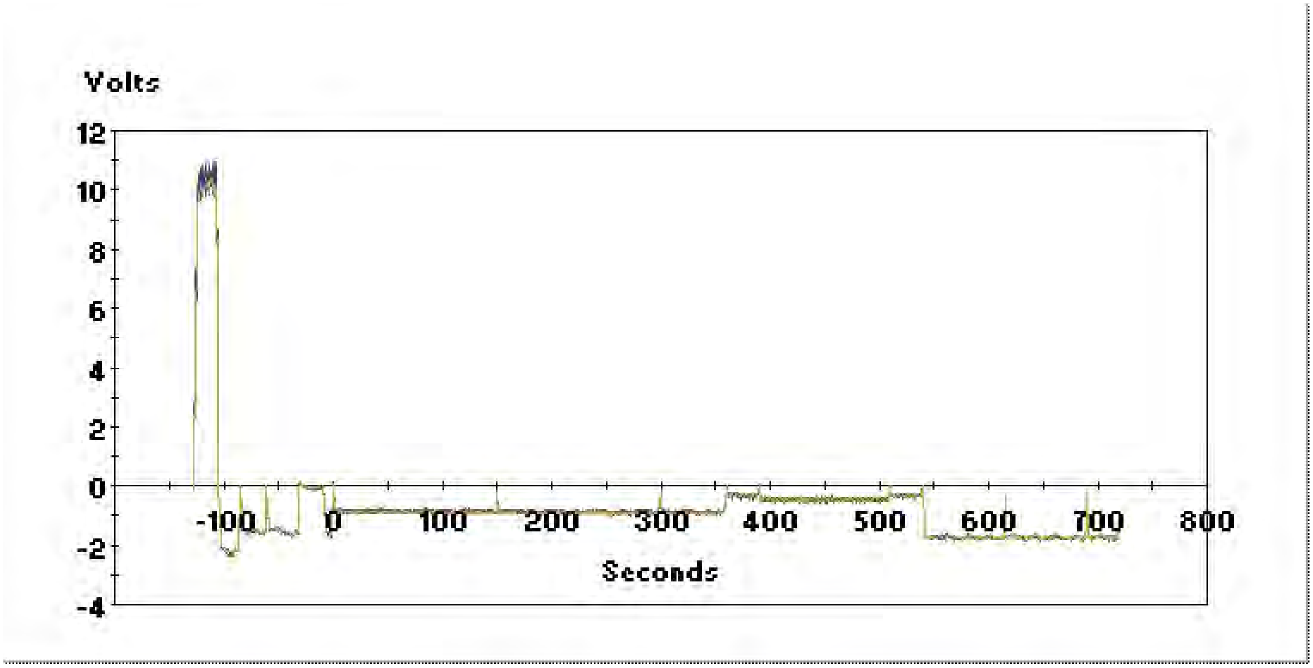

Experiment(x)

**Experiment Name:**	KD TeAb-F IgG1& NXF5 domain 082024	**Start Time:**	Tue Aug 20 22:28:31 2024
**Experiment Type:**	Equilibrium	**End Time:**	Wed Aug 21 14:53:25 2024
**Constant Binding Partner (CBP)**		**Buffer:**	PBS/BSA
**Molecular Concentration:**	175.00pM	**Label:**	Anti-His tag-647
**Valency:**	1	**Label Conc:**	0
**Binding Site Concentration:**	175.00pM		

Comments(x)

beads: 6F5.4 IgG1 100ug on 1ml Sepharose 4B

sample volume: 13 ml

detection: Anti-His-647 (prepared by Lou 082024).1:500

CBP: 350 pM NXF5 domain 081924

titrant: TeAb-F IgG1

titration: 2 cycles, 15 samples: 8 nM - 2pM (1:2) of TeAb-F IgG1, plus NBS & 350pM NXF5 domain only ctr

samples:

1-15) titration

1): NBS

2): 350 pM NXF5 domain only

3 ~ 15): 8nM - 2pM (1:2) of TeAb-F IgG1, Incubate 4hr RT & 6hr 4 C

beads: 6F5.4 IgG1 100ug on 1ml Sepharose 4B

sample volume: 13 ml

detection: Anti-His-647 (prepared by Lou 082024).1:500

CBP: 350 pM NXF5 domain 081924

titrant: TeAb-F IgG1

titration: 2 cycles, 15 samples: 8 nM - 2pM (1:2) of TeAb-F IgG1, plus NBS & 350pM NXF5 domain only ctr

samples:

1-15) titration

1): NBS

2): 350 pM NXF5 domain only

3 ~ 15): 8nM - 2pM (1:2) of TeAb-F IgG1, Incubate 4hr RT & 6hr 4 C

Timing(x)

Bead Handling (Custom Beads)

Draw Source	Time (sec)	Volume (uL)	Rate (mL/min)	Stir
Backflush	20	0	0.0000	
Buffer	20	500	1.5000	✓
Particle Reservoir 1	22	360	1.0000	✓
Buffer	30	500	1.0000	
Waste	2	8	0.2500	
Buffer	20	0	0.0000	
Buffer	9	150	1.0000	

Sample Timing

Draw Source	Time (sec)	Volume (uL)	Rate (mL/min)	Time Stamp
Sample Set 201-217	1029	6000	0.3500	
Buffer	30	125	0.2500	
Rack 1: Tube 21	120	500	0.2500	
Buffer	30	125	0.2500	
Buffer	180	3000	1.0000	

Analysis(x)

Baseline / Endpoints:
to (sec) from beginning
to (sec) from end

Ignore	Binding Signal (V)	Concentration
	0.0201	NSB
✓	0.7922	0
	0.1419	8.00nM
	0.1051	4.00nM
	0.0861	2.00nM
	0.1386	1.00nM
	0.3893	500.00pM
	0.5697	250.00pM
	0.6590	125.00pM
	0.7056	62.50pM
	0.7085	31.25pM
	0.7315	15.63pM
	0.7530	7.81pM
✓	0.8123	3.91pM
✓	0.6904	1.95pM
	0.7083	976.56fM
	0.7147	488.28fM
	0.0396	NSB
✓	0.7617	0
	0.1130	8.00nM
	0.0842	4.00nM
	0.0780	2.00nM
	0.0966	1.00nM
	0.3476	500.00pM
	0.5239	250.00pM
	0.6160	125.00pM
	0.6590	62.50pM
	0.6600	31.25pM
✓	0.6382	15.63pM
	0.6913	7.81pM
	0.6716	3.91pM
	0.6591	1.95pM
	0.6457	976.56fM
	0.6479	488.28fM

**Kd:**	4.41pM
**CBP:**	175.00pM
**Ratio:**	39.6988
**Titrant % Activity:**	19.2532
**Sig 100%:**	0.72
**Drift (%/run):**	0.4304
**NSB:**	0.03
**Drift (mV/run):**	-0.7795
**TR NSB:**	6.11e+07
**%Error:**	1.57

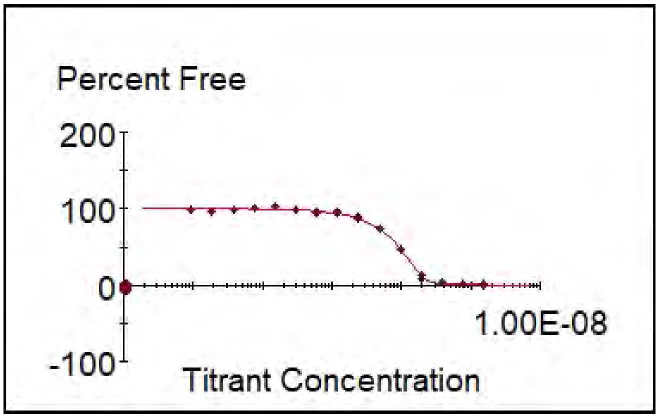

**Kd:**	4.41pM
**95% confidence interval**	
**Kd High:**	9.86pM
**Kd Low:**	1.46pM

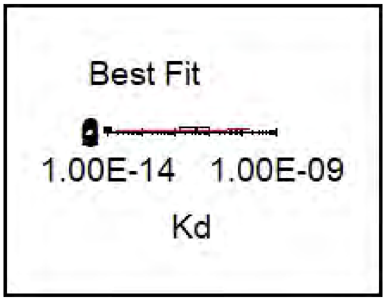

**Titrant %Activity:**	19.2532
**95% confidence interval**	
**Titrant %Activity High:**	21.8227
**Titrant %Activity Low:**	17.2130

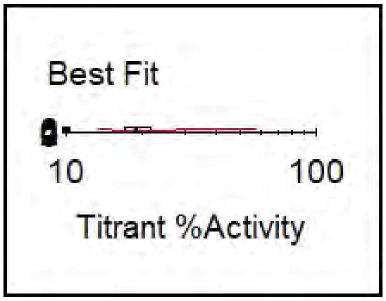

Data Traces(x)

Cycles: 2
Incubation delay (min): 0
Mix Time:

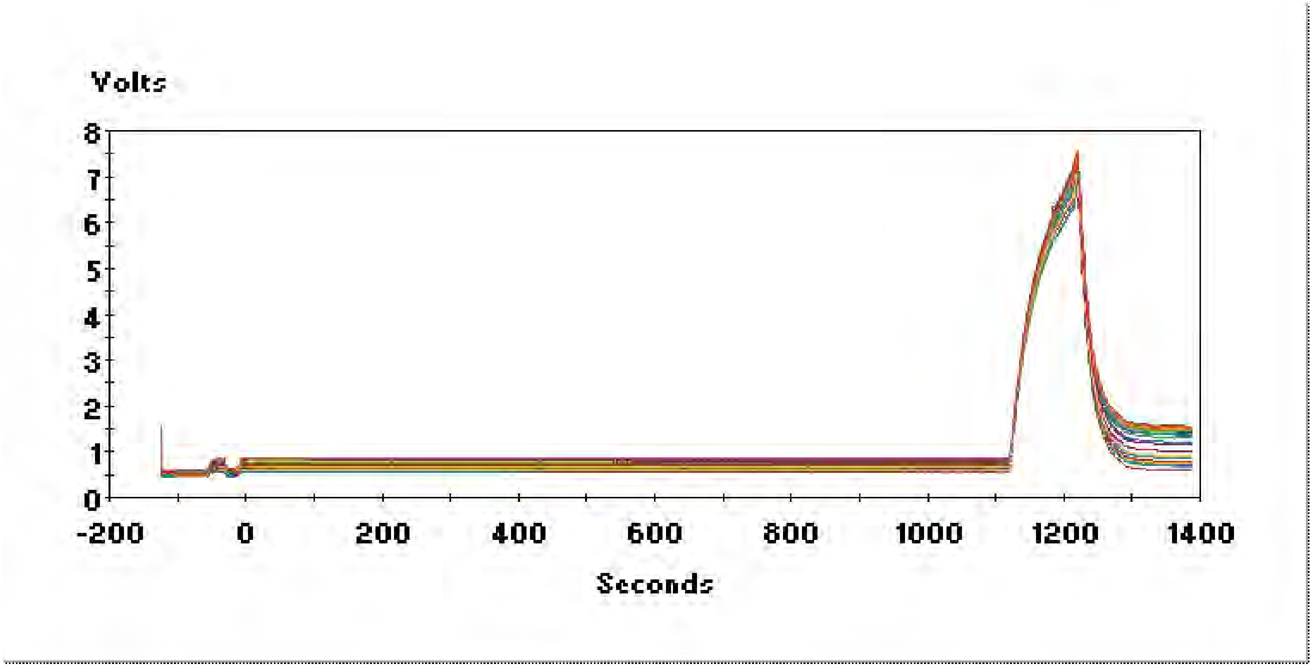

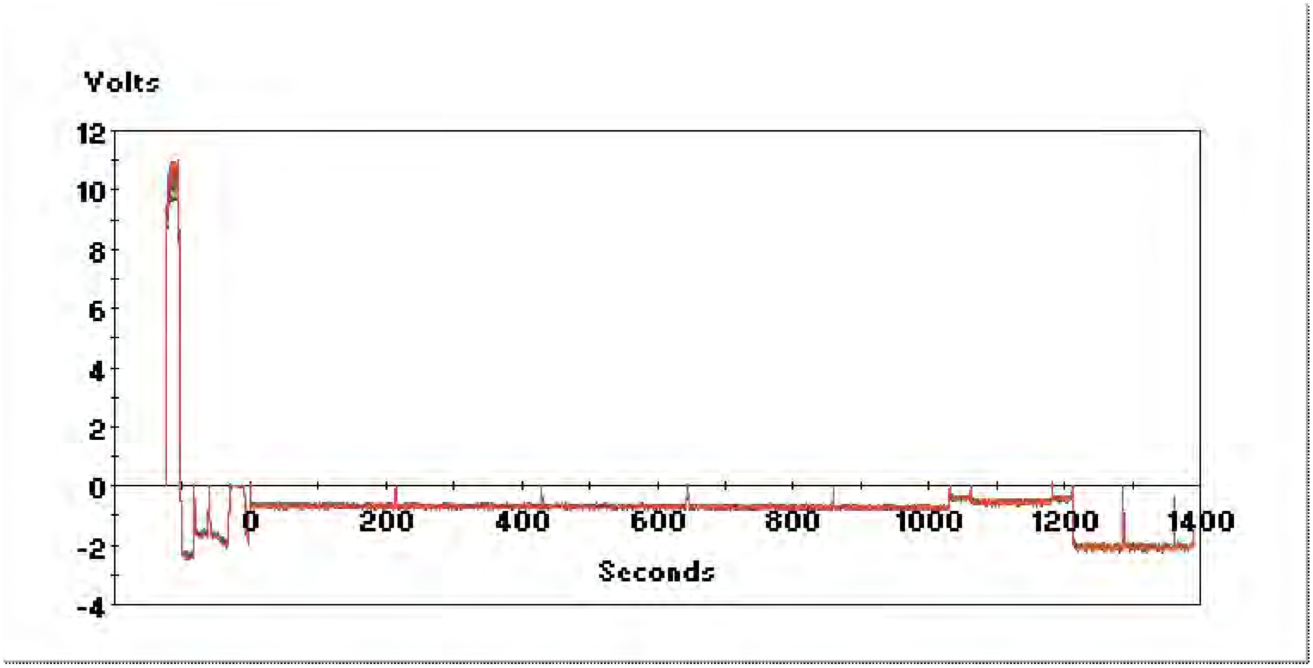

Experiment(x)

**Experiment Name:**	KD TeAb-F IgG1& F1 Toxin(Hu6F11) 090124{2}	**Start Time:**	Sun Sep 1 16:30:30 2024
**Experiment Type:**	Equilibrium	**End Time:**	Mon Sep 2 03:29:48 2024
**Constant Binding Partner (CBP)**		**Buffer:**	PBS/BSA
**Molecular Concentration:**	200.00pM	**Label:**	Anti-Hu6F15.4&Hu6F10-647
**Valency:**	1	**Label Conc:**	0
**Binding Site Concentration:**	200.00pM		

Comments(x)

beads:Hu6F11 IgG1 100ug on 1ml Sepharose 4B

sample volume: 7 ml

detection: Hu6F15.4 &Hu6F10 IgG1-647 (prepared by Lou 083024).1:500

CBP: 200 pM BoNT/F1 082724

titrant: TeAb-F IgG1

titration: 2 cycles, 14 samples: 200pM - 98fM (1:2) of TeAb-F IgG1, plus NBS & 200pM BoNT-F1 only ctr

samples:

1-14) titration

1): NBS

2): 200 pM BoNT/F1 only ctr

3 ~ 14): 200pM - 98fM (1:2) of TeAb-F IgG1, Incubate 1hr RT and O/N 4 C

beads:Hu6F11 IgG1 100ug on 1ml Sepharose 4B

sample volume: 7 ml

detection: Hu6F15.4 &Hu6F10 IgG1-647 (prepared by Lou 083024).1:500

CBP: 200 pM BoNT/F1 082724

titrant: TeAb-F IgG1

titration: 2 cycles, 14 samples: 200pM - 98fM (1:2) of TeAb-F IgG1, plus NBS & 200pM BoNT-F1 only ctr

samples:

1-14) titration

1): NBS

2): 200 pM BoNT/F1 only ctr

3 ~ 14): 200pM - 98fM (1:2) of TeAb-F IgG1, Incubate 1hr RT and O/N 4 C

Timing(x)

Bead Handling (Custom Beads)

Draw Source	Time (sec)	Volume (uL)	Rate (mL/min)	Stir
Backflush	20	0	0.0000	
Buffer	20	500	1.5000	✓
Particle Reservoir 1	24	400	1.0000	✓
Buffer	30	500	1.0000	
Waste	2	8	0.2500	
Buffer	20	0	0.0000	

Sample Timing

Draw Source	Time (sec)	Volume (uL)	Rate (mL/min)	Time Stamp
Sample Set 201-214	720	3000	0.2500	
Buffer	30	125	0.2500	
Rack 1: Tube 21	120	500	0.2500	
Buffer	30	125	0.2500	
Buffer	180	3000	1.0000	

Buffer	9	150	1.0000

Analysis(x)

Baseline / Endpoints:
to (sec) from beginning
to (sec) from end

Ignore	Binding Signal (V)	Concentration
✓	0.1447	NSB
	0.2556	0
	0.1605	200.00pM
	0.1701	100.00pM
	0.2073	50.00pM
	0.2303	25.00pM
	0.2240	12.50pM
✓	0.2595	6.25pM
✓	0.2665	3.13pM
	0.2389	1.56pM
	0.2411	781.25fM
	0.2506	390.63fM
	0.2466	195.31fM
	0.2547	97.66fM
	0.1279	NSB
	0.2531	0
	0.1487	200.00pM
	0.1557	100.00pM
	0.1836	50.00pM
	0.2025	25.00pM
	0.2269	12.50pM
	0.2478	6.25pM
	0.2406	3.13pM
	0.2425	1.56pM
	0.2355	781.25fM
	0.2530	390.63fM
✓	0.2639	195.31fM
	0.2471	97.66fM

**Kd:**	23.30pM
**Active CBP:**	53.72pM
**CBP %Activity:**	26.86
**Ratio:**	2.3054
**Sig 100%:**	0.25
**Drift (%/run):**	-1.1525
**NSB:**	0.13
**Drift (mV/run):**	-1.5944
**%Error:**	4.83

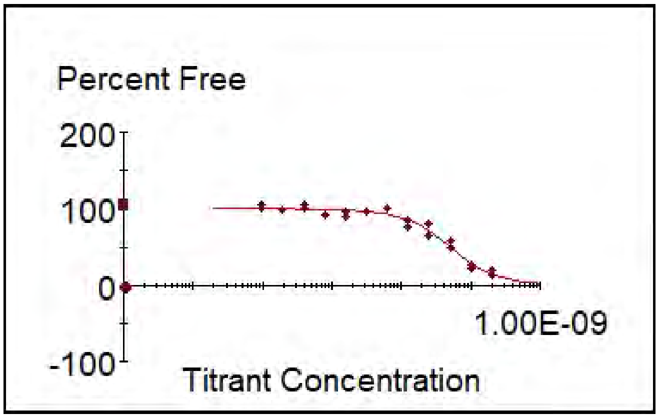

**Kd:**	23.30pM
**95% confidence interval**	
**Kd High:**	65.18pM
**Kd Low:**	3.01pM

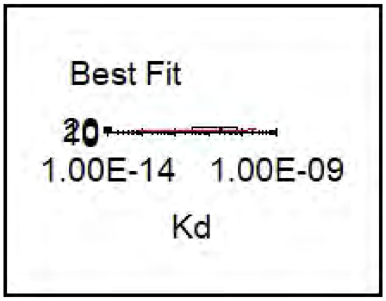

**Active CBP:**	53.72pM
**CBP %Activity:**	26.86
**95% confidence interval**	
**CBP High:**	111.04pM
**%Activity:**	55.52
**CBP Low:**	Less than 194.09fM
**%Activity:**	Less than 0.10

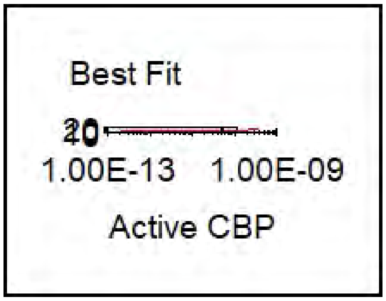

Data Traces (x)

Cycles: 2
Incubation delay (min): 0
Mix Time:

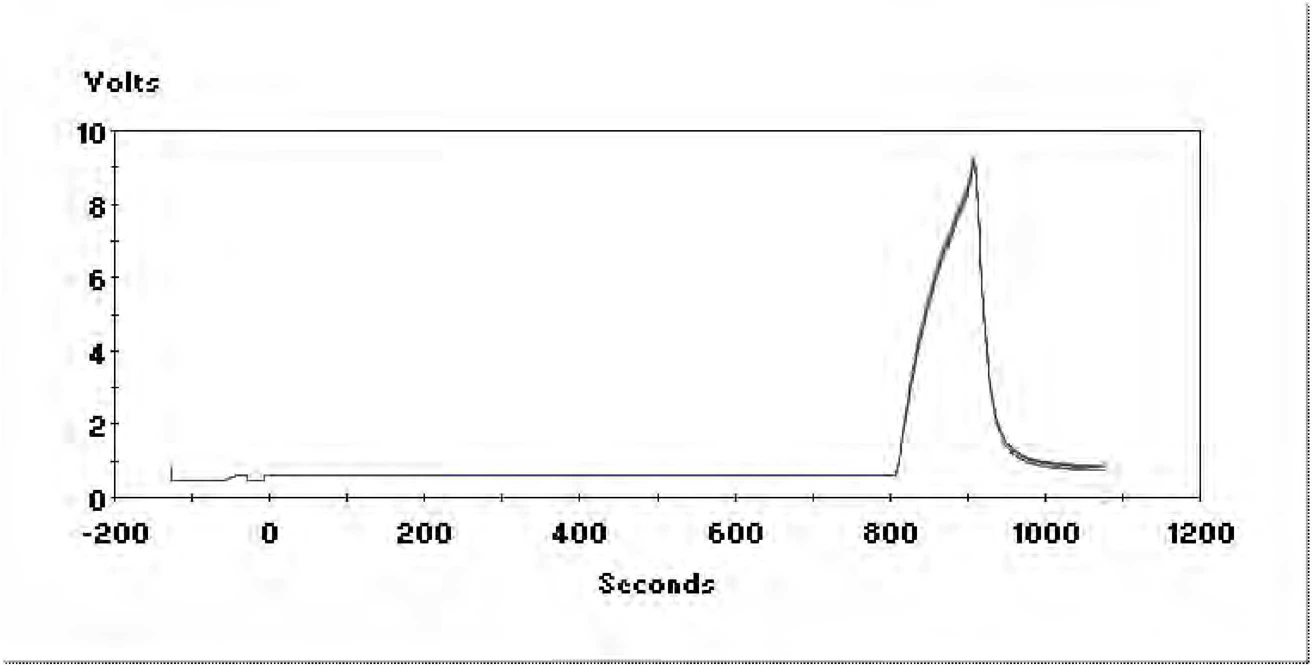

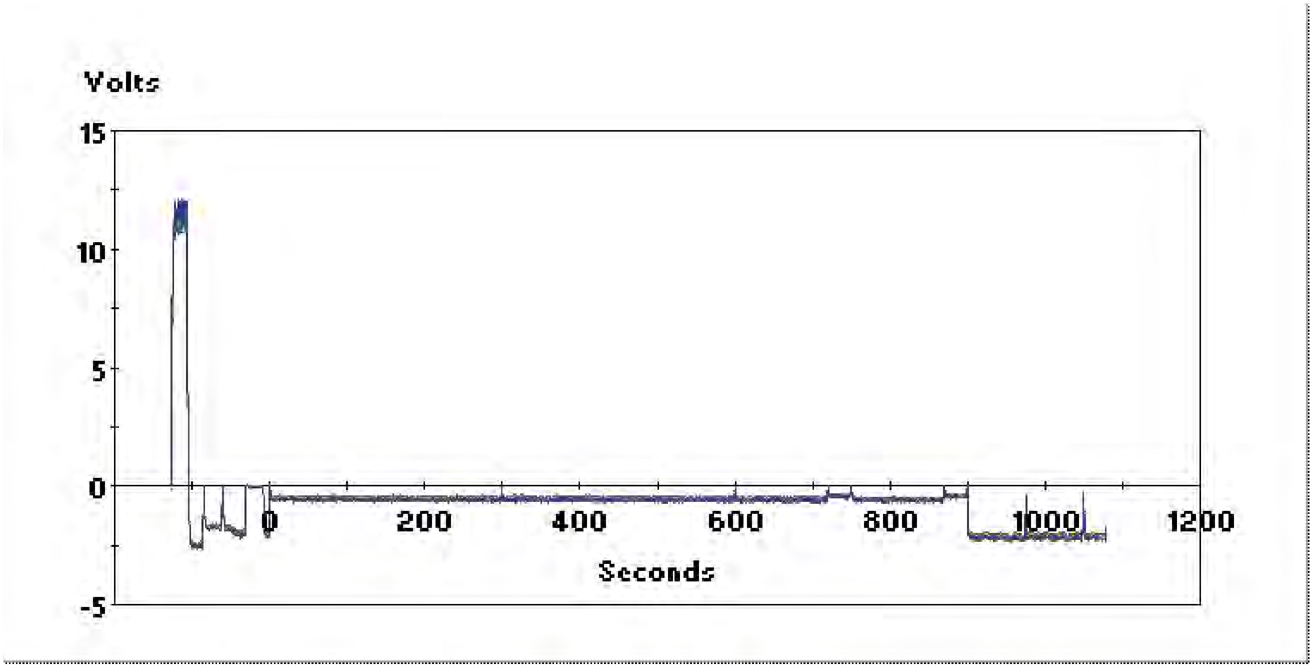

Experiment(x)

**Experiment Name:**	KD TeAb-F IgG1& NXF11 domain 081624	**Start Time:**	Fri Aug 16 12:25:32 2024
**Experiment Type:**	Equilibrium	**End Time:**	Fri Aug 16 21:10:44 2024
**Constant Binding Partner (CBP)**		**Buffer:**	PBS/BSA
**Molecular Concentration:**	500.00pM	**Label:**	Anti-His tag-647
**Valency:**	1	**Label Conc:**	0
**Binding Site Concentration:**	500.00pM		

Comments(x)

beads: Hu6F11 IgG1 100ug on 1ml Sepharose 4B

sample volume: 7 ml

detection: Anti-His-647 (prepared by Lou 081624).1:800

CBP: 500 pM NXF11 domain 081524

titrant: TeAb-F IgG1

titration: 2 cycles, 15 samples: 2 nM - 488fM (1:2) of TeAb-F IgG1, plus NBS & 100pM NXF11 domain only ctr

samples:

1-15) titration

1): NBS

2): 500 pM NXF11 only

3 ~ 15): 2nM - 488 fM (1:2) of Hu6F11 IgG1, Incubate O/N at 4 C

beads: Hu6F11 IgG1 100ug on 1ml Sepharose 4B

sample volume: 7 ml

detection: Anti-His-647 (prepared by Lou 081624).1:800

CBP: 500 pM NXF11 domain 081524

titrant: TeAb-F IgG1

titration: 2 cycles, 15 samples: 2 nM - 488fM (1:2) of TeAb-F IgG1, plus NBS & 100pM NXF11 domain only ctr

samples:

1-15) titration

1): NBS

2): 500 pM NXF11 only

3 ~ 15): 2nM - 488 fM (1:2) of Hu6F11 IgG1, Incubate O/N at 4 C

Timing(x)

Bead Handling (Custom Beads)

Draw Source	Time (sec)	Volume (uL)	Rate (mL/min)	Stir
Backflush	20	0	0.0000	
Buffer	20	500	1.5000	✓
Particle Reservoir 1	23	380	1.0000	✓
Buffer	30	500	1.0000	
Waste	2	8	0.2500	
Buffer	20	0	0.0000	

Sample Timing

Draw Source	Time (sec)	Volume (uL)	Rate (mL/min)	Time Stamp
Sample Set 201-215	360	3000	0.5000	
Buffer	30	125	0.2500	
Rack 1: Tube 21	120	500	0.2500	
Buffer	30	125	0.2500	
Buffer	180	3000	1.0000	

Buffer	9	150	1.0000

Analysis (x)

Baseline / Endpoints:

to (sec) from beginning

to (sec) from end

Ignore	Binding Signal (V)	Concentration
	0.0107	NSB
✓	1.1075	0
	0.0842	2.00nM
	0.2346	1.00nM
	0.4031	500.00pM
	0.6715	250.00pM
	0.8557	125.00pM
	0.9624	62.50pM
	1.0033	31.25pM
	1.0059	15.63pM
	1.0304	7.81pM
✓	1.1090	3.91pM
	1.0537	1.95pM
	1.0317	976.56fM
	1.0450	488.28fM
	0.0122	NSB
✓	1.0079	0
	0.0701	2.00nM
	0.1208	1.00nM
	0.3214	500.00pM
	0.5817	250.00pM
	0.7778	125.00pM
	0.8627	62.50pM
	0.8970	31.25pM
	0.9145	15.63pM
✓	0.9695	7.81pM
✓	0.9689	3.91pM
	0.9228	1.95pM
	0.9623	976.56fM
	0.9733	488.28fM

**Kd:**	125.07pM
**Active CBP:**	421.03pM
**CBP %Activity:**	84.21
**Ratio:**	3.3664
**Sig 100%:**	1.03
**Drift (%/run):**	0.3079
**NSB:**	-0.01
**Drift (mV/run):**	-2.9458
**%Error:**	1.65

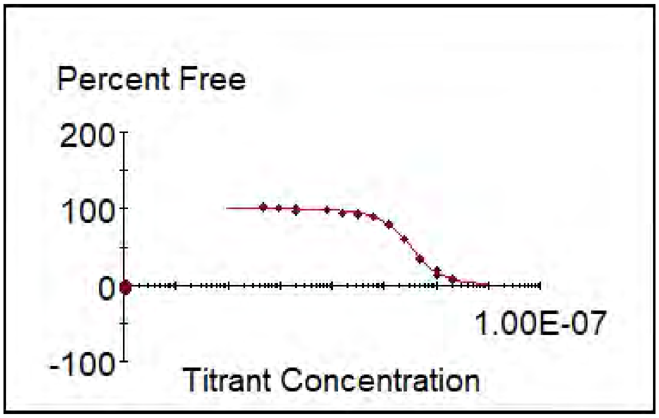

**Kd:**	125.07pM
**95% confidence interval**	
**Kd High:**	181.21pM
**Kd Low:**	80.10pM

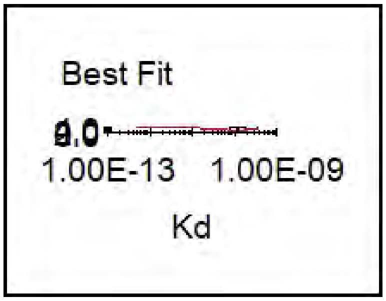

**Active CBP:**	421.03pM
**CBP %Activity:**	84.21
**95% confidence interval**	
**CBP High:**	548.93pM
**%Activity:**	109.79
**CBP Low:**	278.16pM
**%Activity:**	55.63

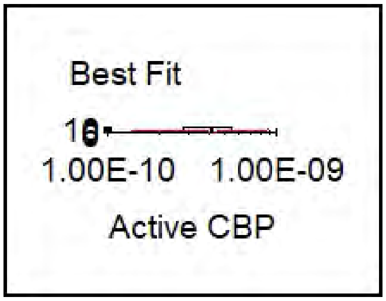

Data Traces(x)

Cycles: 2
Incubation delay (min): 0
Mix Time:

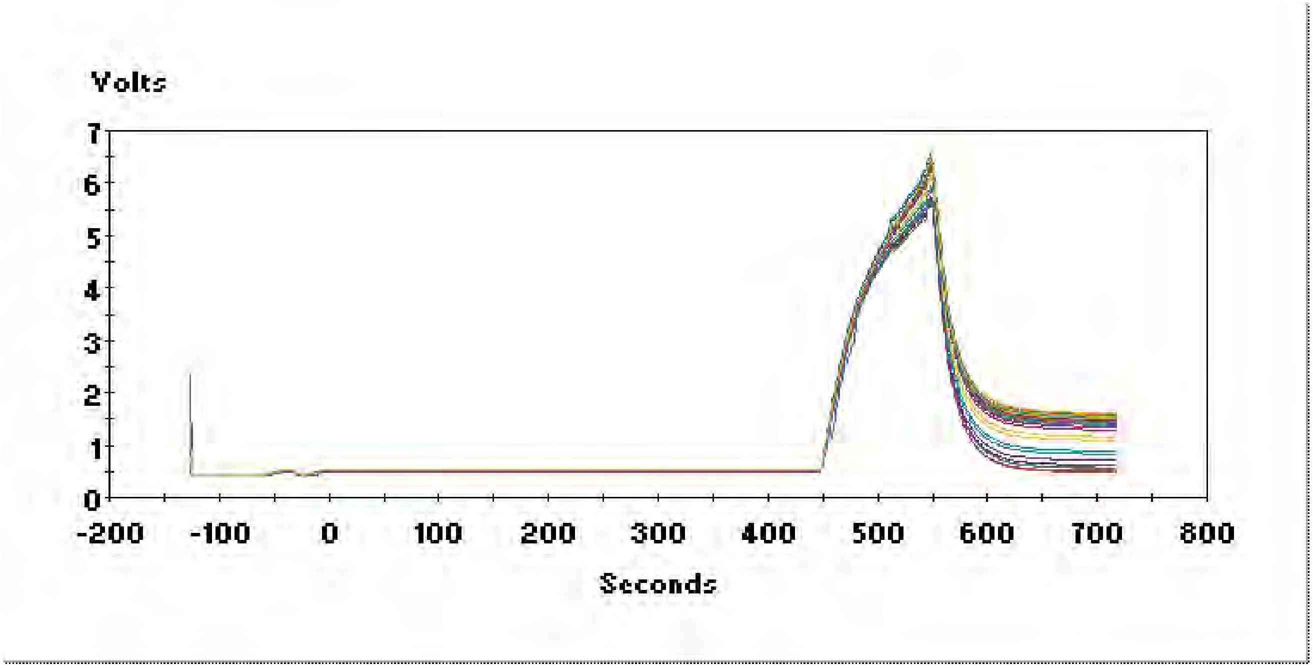

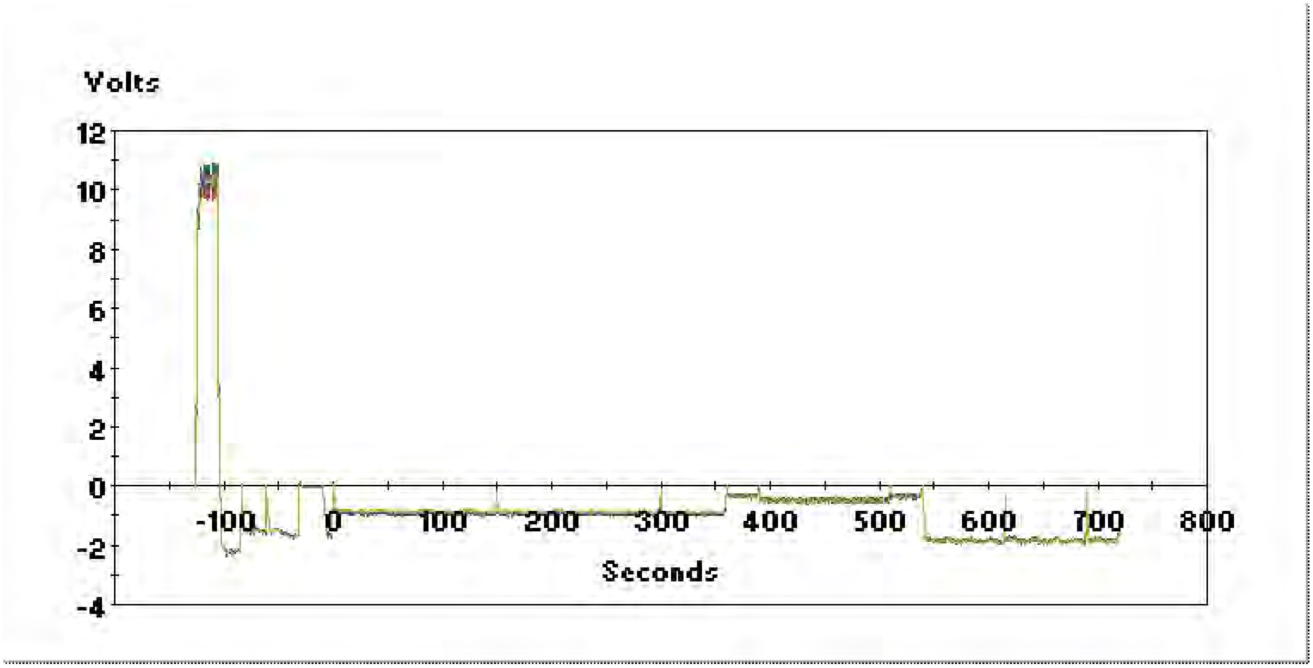

Experiment(x)

**Experiment Name:**	KD TeAb-F IgG1& F1 Toxin(Hu6F13.4) 090524	**Start Time:**	Thu Sep 5 12:05:56 2024
**Experiment Type:**	Equilibrium	**End Time:**	Thu Sep 5 23:04:47 2024
**Constant Binding Partner (CBP)**		**Buffer:**	PBS/BSA
**Molecular Concentration:**	200.00pM	**Label:**	Anti-Hu6F15.4&Hu6F10-647
**Valency:**	1	**Label Conc:**	0
**Binding Site Concentration:**	200.00pM		

Comments(x)

beads:Hu6F13.4 IgG1 100ug on 1ml Sepharose 4B

sample volume: 7 ml

detection: Hu6F15.4 &Hu6F10 IgG1-647 (prepared by Lou 090324).1:500

CBP: 200 pM BoNT/F1 082724

titrant: TeAb-F IgG1

titration: 2 cycles, 14 samples: 200pM - 98fM (1:2) of TeAb-F IgG1, plus NBS & 200pM BoNT-F1 only ctr

samples:

1-14) titration

1): NBS

2): 200 pM BoNT/F1 only ctr

3 ~ 14): 200pM - 98fM (1:2) of TeAb-F IgG1, Incubate 4hr RT and O/N 4 C

beads:Hu6F13.4 IgG1 100ug on 1ml Sepharose 4B

sample volume: 7 ml

detection: Hu6F15.4 &Hu6F10 IgG1-647 (prepared by Lou 090324).1:500

CBP: 200 pM BoNT/F1 082724

titrant: TeAb-F IgG1

titration: 2 cycles, 14 samples: 200pM - 98fM (1:2) of TeAb-F IgG1, plus NBS & 200pM BoNT-F1 only ctr

samples:

1-14) titration

1): NBS

2): 200 pM BoNT/F1 only ctr

3 ~ 14): 200pM - 98fM (1:2) of TeAb-F IgG1, Incubate 4hr RT and O/N 4 C

Timing(x)

Bead Handling (Custom Beads)

Draw Source	Time (sec)	Volume (uL)	Rate (mL/min)	Stir
Backflush	20	0	0.0000	
Buffer	20	500	1.5000	✓
Particle Reservoir 1	23	380	1.0000	✓
Buffer	30	500	1.0000	
Waste	2	8	0.2500	
Buffer	20	0	0.0000	

Sample Timing

Draw Source	Time (sec)	Volume (uL)	Rate (mL/min)	Time Stamp
Sample Set 201-214	720	3000	0.2500	
Buffer	30	125	0.2500	
Rack 1: Tube 21	120	500	0.2500	
Buffer	30	125	0.2500	
Buffer	180	3000	1.0000	

Buffer	9	150	1.0000

Analysis(x)

Baseline / Endpoints:
to (sec) from beginning
to (sec) from end

Ignore	Binding Signal (V)	Concentration
✓	0.1093	NSB
	0.4176	0
	0.1288	200.00pM
	0.2100	100.00pM
	0.2821	50.00pM
	0.3221	25.00pM
	0.3383	12.50pM
	0.3529	6.25pM
	0.3532	3.13pM
	0.3780	1.56pM
	0.3582	781.25fM
✓	0.3970	390.63fM
	0.3647	195.31fM
	0.3533	97.66fM
	0.0580	NSB
✓	0.3390	0
	0.0883	200.00pM
	0.1575	100.00pM
	0.2405	50.00pM
	0.2827	25.00pM
	0.3220	12.50pM
	0.3347	6.25pM
	0.3398	3.13pM
	0.3351	1.56pM
	0.3412	781.25fM
	0.3483	390.63fM
	0.3473	195.31fM
	0.3397	97.66fM

**Kd:**	15.50pM
**Active CBP:**	114.42pM
**CBP %Activity:**	57.21
**Ratio:**	7.3803
**Sig 100%:**	0.36
**Drift (%/run):**	-0.5722
**NSB:**	0.06
**Drift (mV/run):**	-3.7185
**%Error:**	2.80

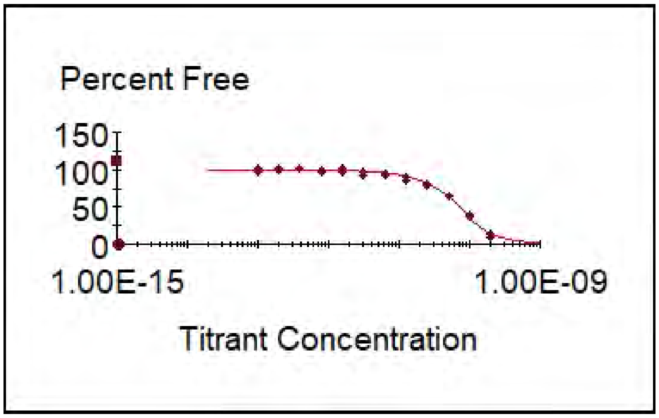

**Kd:**	15.50pM
**95% confidence interval**	
**Kd High:**	35.52pM
**Kd Low:**	3.48pM

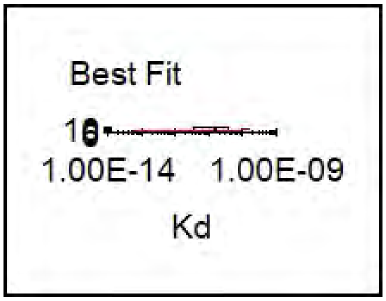

**Active CBP:**	114.42pM
**CBP %Activity:**	57.21
**95% confidence interval**	
**CBP High:**	154.09pM
**%Activity:**	77.04
**CBP Low:**	61.40pM
**%Activity:**	30.70

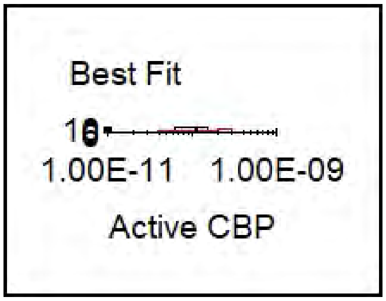

Data Traces(x)

Cycles: 2
Incubation delay (min): 0
Mix Time:

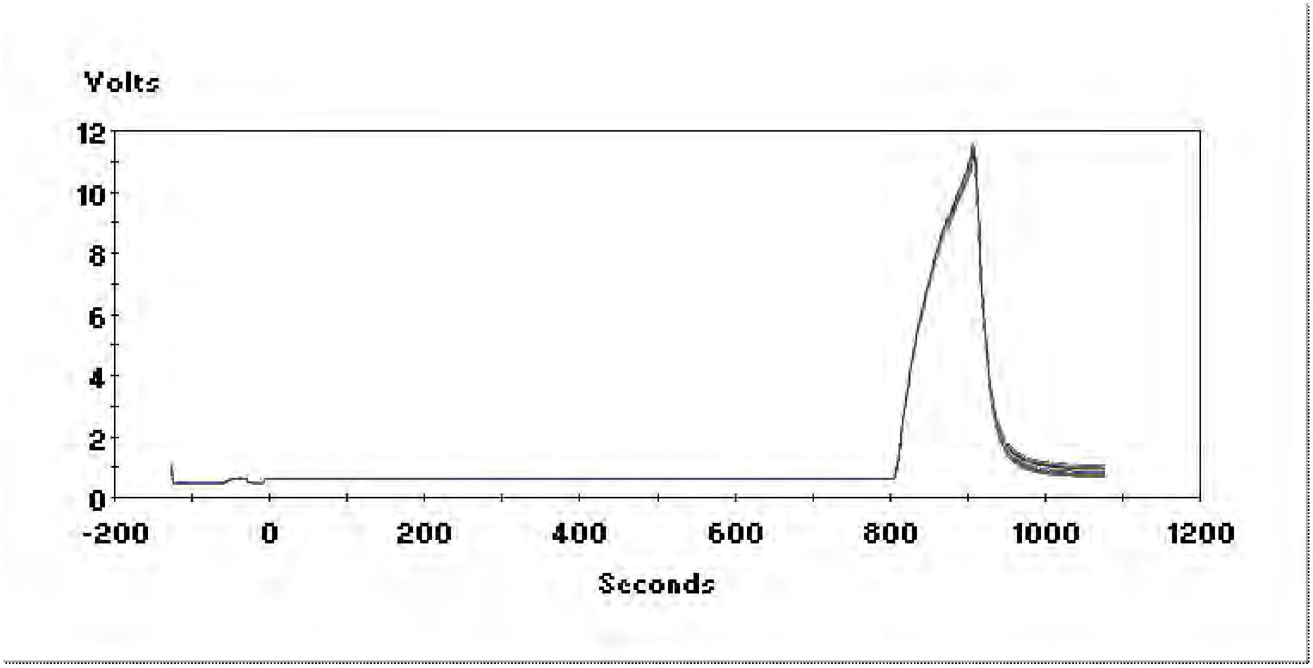

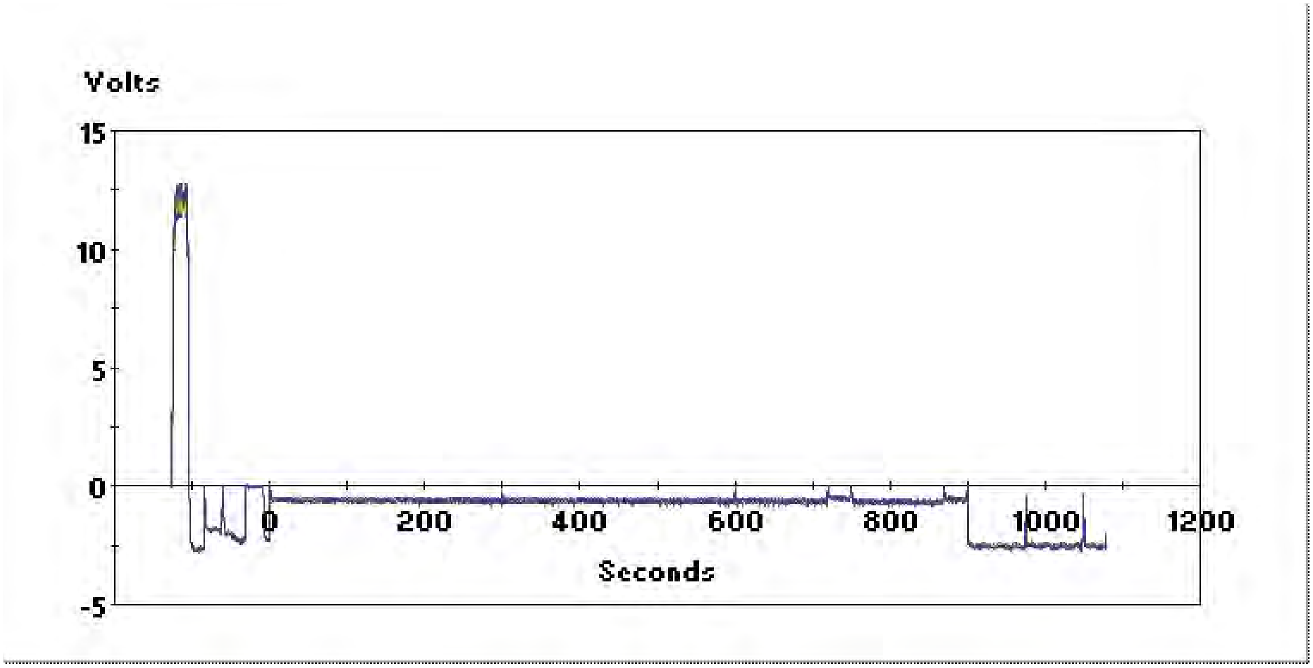

Experiment(x)

**Experiment Name:**	KD TeAb-F IgG1& NXF13 domain 013025	**Start Time:**	Thu Jan 30 14:50:44 2025
**Experiment Type:**	Equilibrium	**End Time:**	Thu Jan 30 21:07:28 2025
**Constant Binding Partner (CBP)**		**Buffer:**	PBS/BSA
**Molecular Concentration:**	200.00pM	**Label:**	Anti-His tag-647
**Valency:**	1	**Label Conc:**	0
**Binding Site Concentration:**	200.00pM		

Comments(x)

beads: Hu6F13.4 IgG1 100ug on 1ml Sepharose 4B (12/15/24)

sample volume: 7 ml

detection: Anti-His-647 (prepared by Lou 121624).1:2000

CBP: 200 pM NXF13 domain 121624

titrant: TeAb-F IgG1(Batch 091418 MW258KD 0.24mg/ml)

titration: 1 cycles, 16 samples: 16 nM - 1.95 pM (1:2) of TeAb-F IgG1, plus NBS & 200pM NXF13 domain only ctr

samples:

1-15) titration,16 nM - 1.95 pM (1:2) of TeAb-F IgG1

16): 200 pM NXF13 only

beads: Hu6F13.4 IgG1 100ug on 1ml Sepharose 4B (12/15/24)

sample volume: 7 ml

detection: Anti-His-647 (prepared by Lou 121624).1:2000

CBP: 200 pM NXF13 domain 121624

titrant: TeAb-F IgG1(Batch 091418 MW258KD 0.24mg/ml)

titration: 1 cycles, 16 samples: 16 nM - 1.95 pM (1:2) of TeAb-F IgG1, plus NBS & 200pM NXF13 domain only ctr

samples:

1-15) titration,16 nM - 1.95 pM (1:2) of TeAb-F IgG1

16): 200 pM NXF13 only

Timing(x)

Bead Handling (Custom Beads)

Draw Source	Time (sec)	Volume (uL)	Rate (mL/min)	Stir
Backflush	20	0	0.0000	
Buffer	20	500	1.5000	✓
Particle Reservoir 1	24	400	1.0000	✓
Buffer	30	500	1.0000	
Waste	2	8	0.2500	
Buffer	20	0	0.0000	
Buffer	9	150	1.0000	

Sample Timing

Draw Source	Time (sec)	Volume (uL)	Rate (mL/min)	Time Stamp
Sample Set 201-216	720	3000	0.2500	
Buffer	30	125	0.2500	
Rack 1: Tube 21	120	500	0.2500	
Buffer	30	125	0.2500	
Buffer	180	3000	1.0000	

Analysis (x)

Baseline / Endpoints:
to (sec) from beginning
to (sec) from end

Ignore	Binding Signal (V)	Concentration
	0.0592	NSB
	0.1560	8.00nM
	0.3905	4.00nM
	0.3947	2.00nM
	0.6279	1.00nM
	0.6231	500.00pM
	0.8117	250.00pM
	0.8806	125.00pM
	0.8657	62.50pM
	0.8847	31.25pM
	0.8578	15.63pM
	0.9118	7.81pM
	0.8736	3.91pM
	0.8575	1.95pM
	0.8750	976.56fM
	0.8581	488.28fM

**Kd:**	1.79nM
**Active CBP:**	1.78pM
**CBP %Activity:**	0.89
**Ratio:**	0.0010
**Sig 100%:**	0.88
**NSB:**	0.05
**%Error:**	4.87

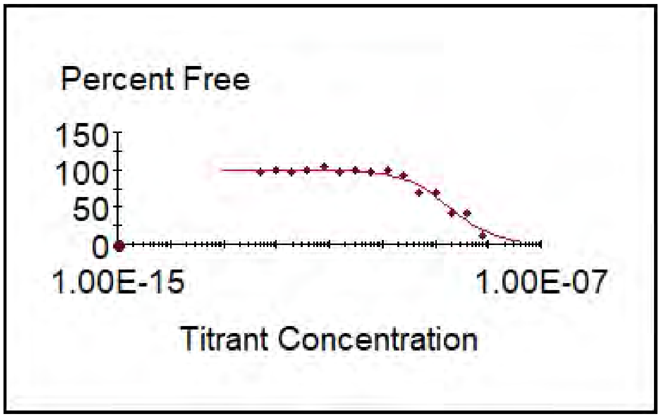

**Kd:**	1.79nM
**95% confidence interval**	
**Kd High:**	2.50nM
**Kd Low:**	755.38pM

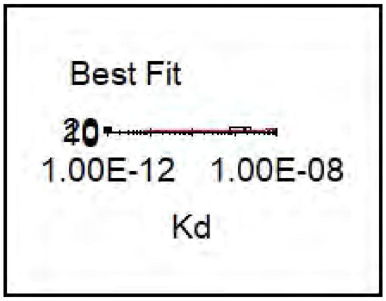

**Active CBP:**	1.78pM
**CBP %Activity:**	0.89
**95% confidence interval**	
**CBP High:**	Greater than 492.32pM
**%Activity:**	Greater than 246.16
**CBP Low:**	Less than 6.43fM
**%Activity:**	Less than 0.00

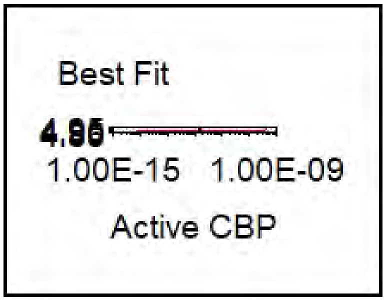

Data Traces(x)

Cycles: 1
Incubation delay (min): 30
Mix Time:

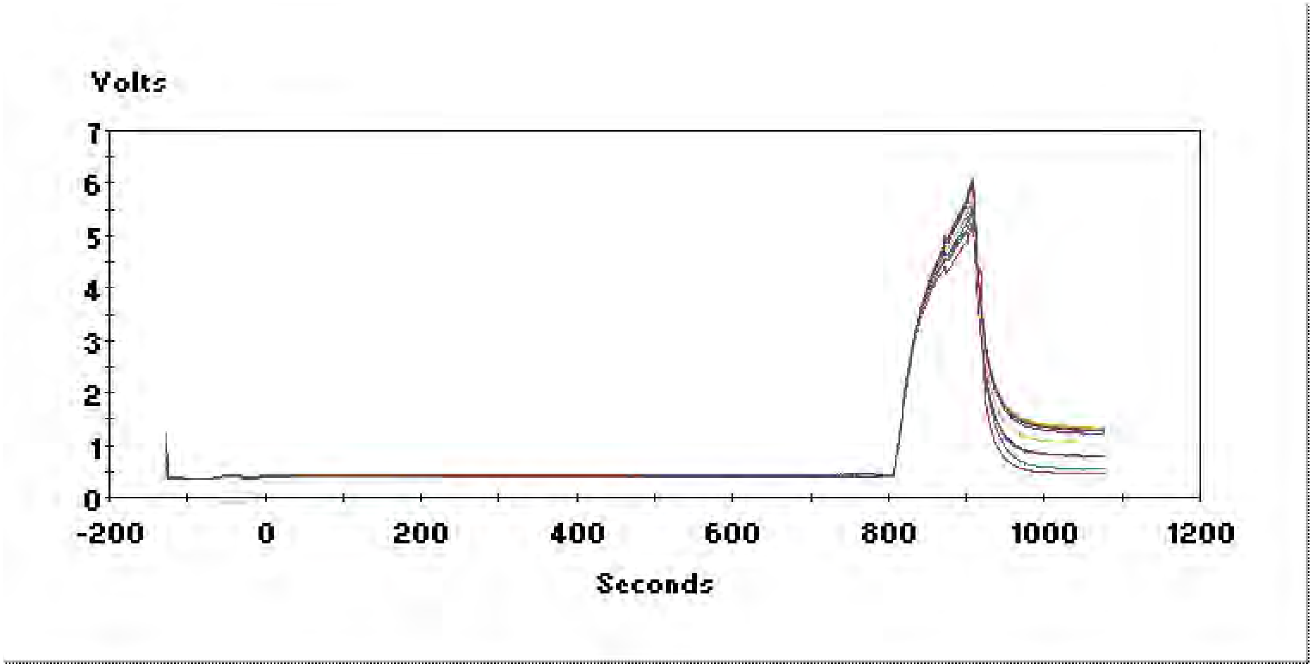

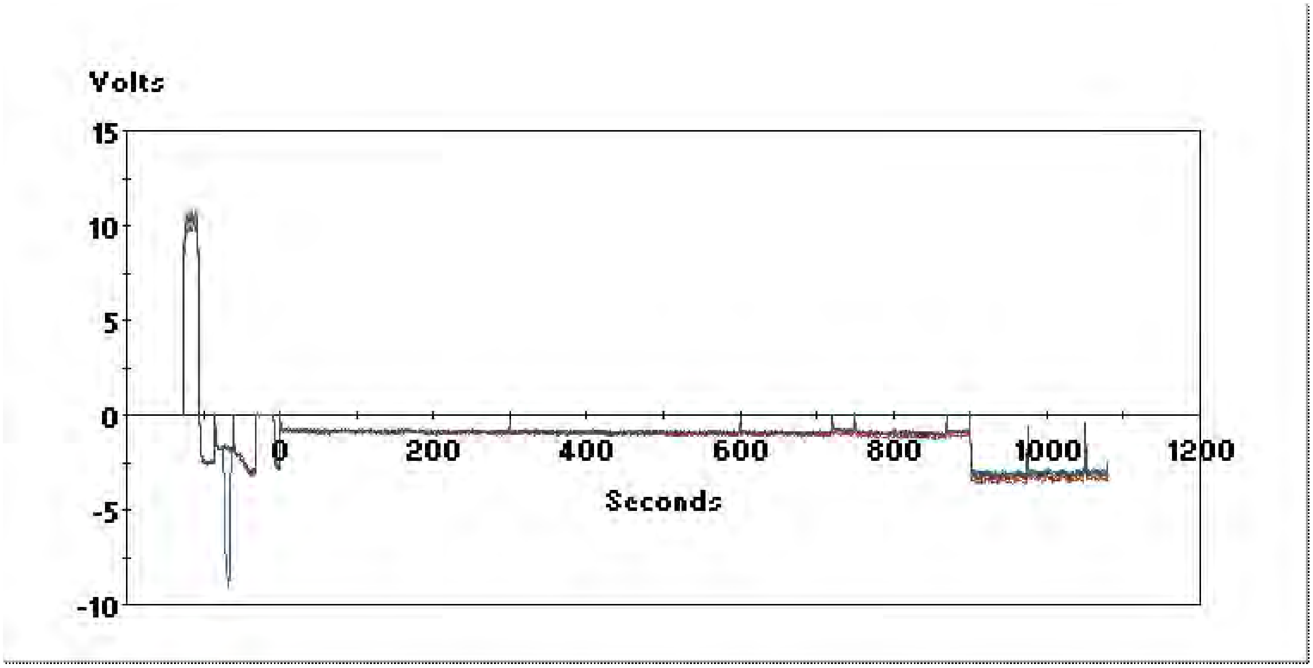

Experiment(x)

**Experiment Name:**	KinDir 6F5.4 IgG1 vs BoNT	**Start Time:**	Sat Sep 7 20:25:01 2024
**Experiment Type:**	Kinetics, Direct	**End Time:**	Sat Sep 7 22:21:21 2024
**Binding Site Concentration:**	200.00pM	**Buffer:**	PBS/BSA
**Kd:**	189.63pM	**Label:**	Anti-His-647
**Titrant:**	200.00pM	**Label Conc:**	0

Comments(x)

beads: 6F5.4 IgG1 coated 09/06/24

sample volume: 500 ul

detection: Hu6F10&Hu6F15.4 -647 (1:800)

CBP: 100 pM [final] BoNT/F1

titrant: 200 pM [final] 6F5.4 IgG 09/07/24

beads: 6F5.4 IgG1 coated 09/06/24

sample volume: 500 ul

detection: Hu6F10&Hu6F15.4 -647 (1:800)

CBP: 100 pM [final] BoNT/F1

titrant: 200 pM [final] 6F5.4 IgG 09/07/24

Timing(x)

Bead Handling (Custom Beads)

Draw Source	Time (sec)	Volume (uL)	Rate (mL/min)	Stir
Backflush	20	0	0.0000	
Buffer	20	500	1.5000	✓
Particle Reservoir 1	23	380	1.0000	✓
Buffer	30	500	1.0000	
Waste	2	8	0.2500	
Buffer	20	0	0.0000	
Buffer	9	150	1.0000	

Sample Timing

Draw Source	Time (sec)	Volume (uL)	Rate (mL/min)	Time Stamp
Rack 2: Tube 1	120	500	0.2500	
Buffer	30	125	0.2500	
Rack 1: Tube 21	120	500	0.2500	
Buffer	30	125	0.2500	
Buffer	90	1500	1.0000	

Analysis(x)

Baseline / Endpoints:
to (sec) from beginning
to (sec) from end

Binding		
**Ignore**	**Signal (V)**	**Time**
	0.4103	308
	0.4016	1005
	0.3943	1701
	0.3903	2398.5
	0.3847	3095
	0.3701	3792.5
	0.3758	4489.5
	0.3795	5187
	0.3692	5884.5
	0.3818	6583

**kon:**	9.731e+05/Ms
**koff:**	1.845e-04/s
**Sig 100%:**	0.42
**NSB:**	0.30
**%Error:**	3.97
**Kd:**	189.63pM
**CBP:**	200.00pM
**Titrant:**	200.00pM

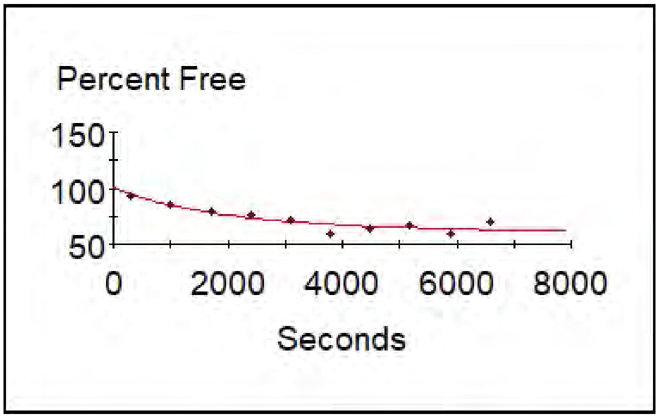

**kon:**	9.731e+05/Ms
**95% confidence interval**	
**kon High:**	1.717e+06/Ms
**kon Low:**	4.249e+05/Ms

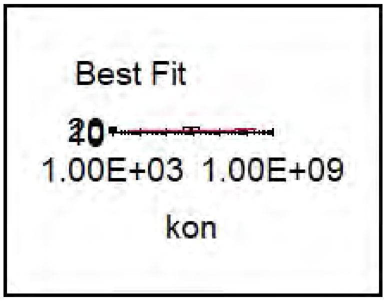

Data Traces(x)

**Cycles:**	10
**Incubation delay (min):**	0
**Mix Time:**	Sat Sep 7 20:24:50 2024

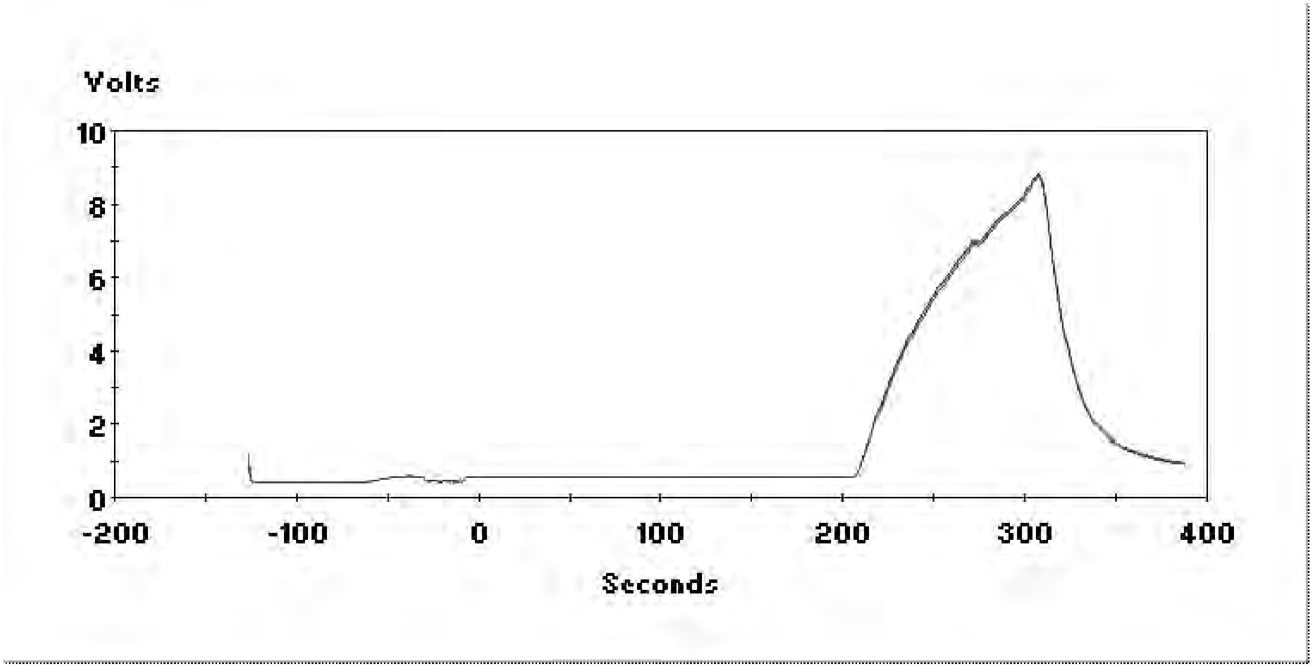

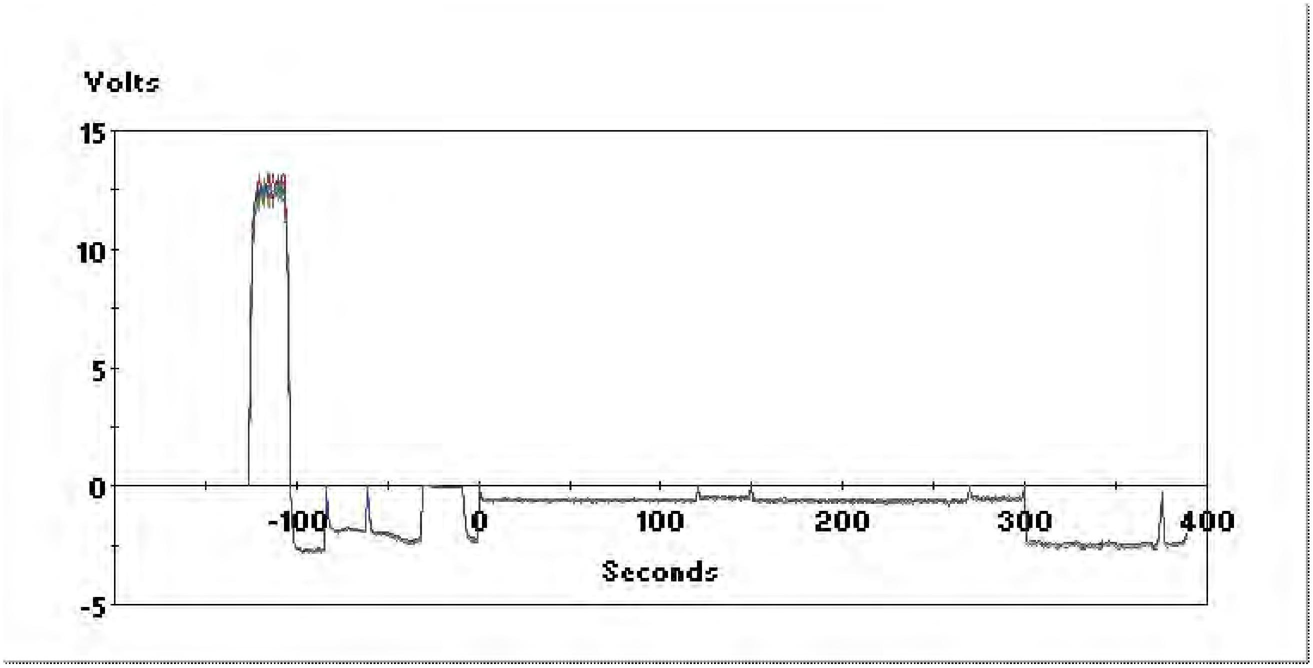

Experiment(x)

**Experiment Name:**	KinDir 6F5.4 IgG1 vs NXF5 domain Kon Rep 082024	**Start Time:**	Tue Aug 20 15:01:15 2024
**Experiment Type:**	Kinetics, Direct	**End Time:**	Tue Aug 20 16:57:31 2024
**Binding Site Concentration:**	100.00pM	**Buffer:**	PBS/BSA
**Kd:**	2.40pM	**Label:**	Anti-His-647
**Titrant:**	200.00pM	**Label Conc:**	0

Comments(x)

beads: 6F5.4 IgG1 coated 08/16/24

sample volume: 500 ul

detection: Anti-His -647 (1:500)

CBP: 100 pM [final] NXF5 Domain

titrant: 200 pM [final] 6F5.4 IgG 08/20/24

Timing(x)

Bead Handling (Custom Beads)

Draw Source	Time (sec)	Volume (uL)	Rate (mL/min)	Stir
Backflush	20	0	0.0000	
Buffer	20	500	1.5000	✓
Particle Reservoir 1	23	380	1.0000	✓
Buffer	30	500	1.0000	
Waste	2	8	0.2500	
Buffer	20	0	0.0000	
Buffer	9	150	1.0000	

Sample Timing

Draw Source	Time (sec)	Volume (uL)	Rate (mL/min)	Time Stamp
Rack 2: Tube 1	120	500	0.2500	
Buffer	30	125	0.2500	
Rack 2: Tube 60	120	500	0.2500	
Buffer	30	125	0.2500	
Buffer	90	1500	1.0000	

Analysis(x)

Baseline / Endpoints:
to (sec) from beginning
to (sec) from end

Binding

Ignore	Signal (V)	Time
✓	0.1887	305
✓	0.1912	1000.5
	0.2553	1697.5
	0.2113	2393.5
	0.1851	3090.5
✓	0.2026	3787
	0.1947	4484.5
	0.1981	5181
	0.1865	5878.5
✓	0.2081	6576

**kon:**	1.837e+07/Ms
**koff:**	4.409e-05/s
**Sig 100%:**	3.62
**NSB:**	0.11
**%Error:**	0.13
**Kd:**	2.40pM
**CBP:**	100.00pM
**Titrant:**	200.00pM

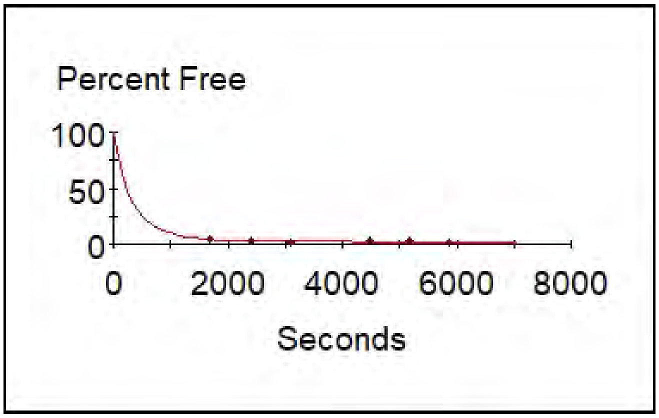

kon: 1.837e+07/Ms
95% confidence interval
kon High: 2.314e+07/Ms
kon Low: 1.351e+07/Ms

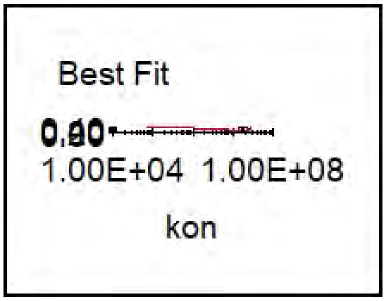

Data Traces(x)

**Cycles:**	10
**Incubation delay (min):**	0
**Mix Time:**	Tue Aug 20 15:01:07 2024

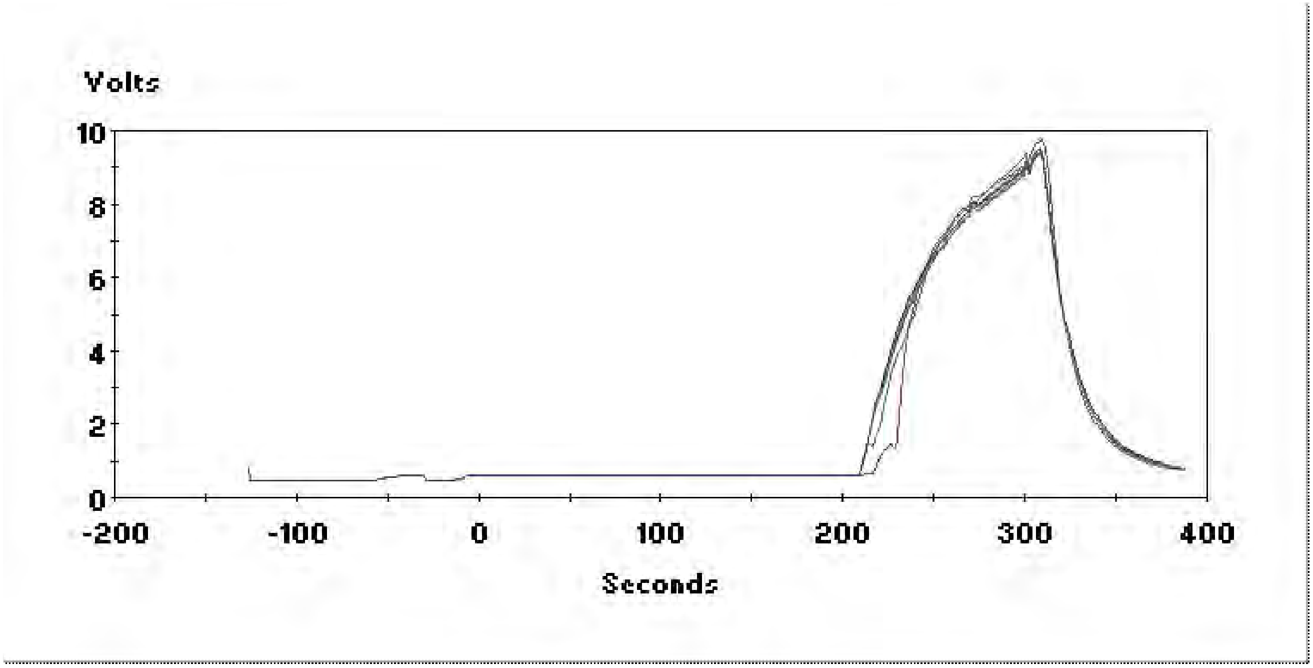

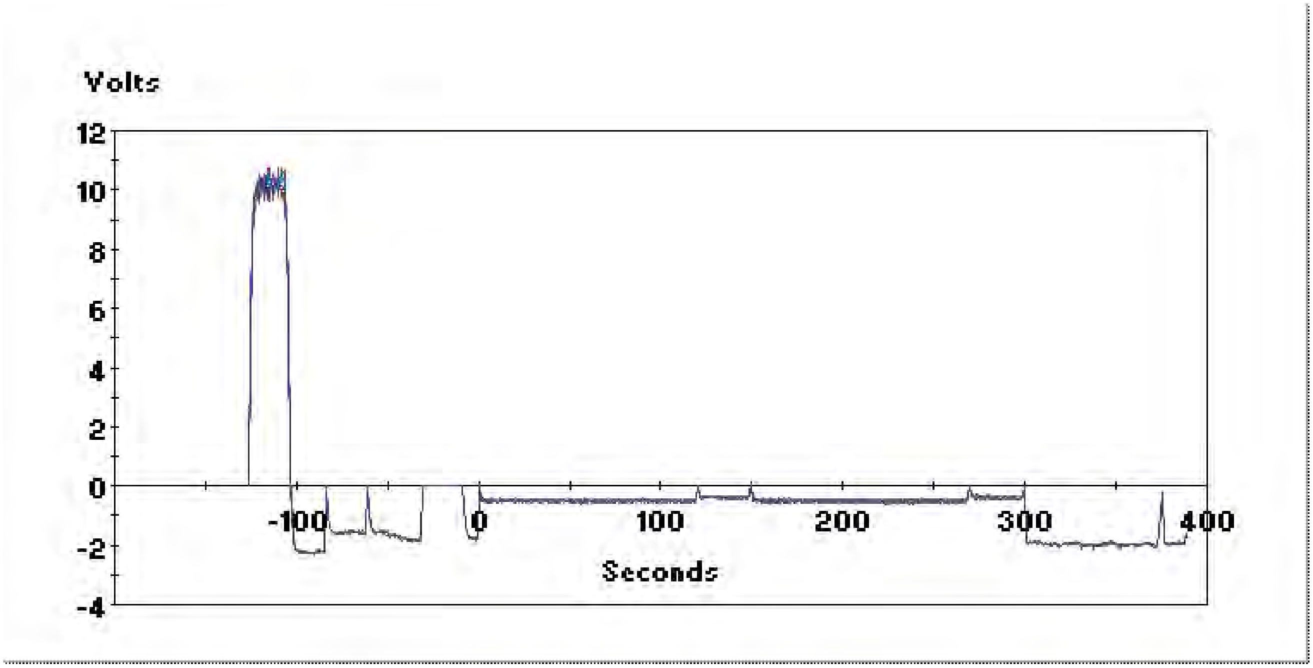

Experiment(x)

**Experiment Name:**	KinDir hu6F11 vs F1	**Start Time:**	Wed Jul 10 11:05:15 2013
**Experiment Type:**	Kinetics, Direct	**End Time:**	Wed Jul 10 13:28:46 2013
**Binding Site Concentration:**	300.00pM	**Buffer:**	PBS/BSA
**Kd:**	348.00fM	**Label:**	6F5.1-647
**Titrant:**	1.00nM	**Label Conc:**	0

Comments(x)

hu6F11 beads 6/21/13

BoNT F1 100250 6/21/13

hu6F11 IgG 6/20/13

6F5.1-647

meter: 1.2676

Timing(x)

Bead Handling (Soft Beads)

Draw Source	Time (sec)	Volume (uL)	Rate (mL/min)	Stir
Backflush	20	0	0.0000	
Buffer	20	500	1.5000	✓
Particle Reservoir	22	367	1.0000	✓
Buffer	40	333	0.5000	
Waste	5	25	0.3000	
Buffer	2	10	0.3000	
Buffer	20	0	0.0000	
Buffer	20	83	0.2500	
Waste	5	25	0.3000	
Buffer	2	10	0.3000	
Buffer	20	0	0.0000	
Buffer	36	150	0.2500	

Sample Timing

Draw Source	Time (sec)	Volume (uL)	Rate (mL/min)	Time Stamp
Line 1	120	500	0.2500	
Buffer	30	125	0.2500	
Inject	120	500	0.2500	
Buffer	30	125	0.2500	
Buffer	90	1500	1.0000	

Analysis(x)

Baseline / Endpoints:
to (sec) from beginning
to (sec) from end

Binding		
**Ignore**	**Signal (V)**	**Time**
	0.6874	463
	0.4456	1125
	0.3216	1787
	0.2487	2449.5
	0.2068	3113
	0.1807	3775
	0.1692	4437.5
	0.1521	5100
	0.1458	5762
	0.1408	6424
	0.1488	7087
	0.1431	7749
	0.1493	8412

**kon:**	1.035e+06/Ms
**koff:**	3.602e-07/s
**Sig 100%:**	1.00
**NSB:**	0.14
**%Error:**	0.41
**Kd:**	348.00fM
**CBP:**	300.00pM
**Titrant:**	1.00nM

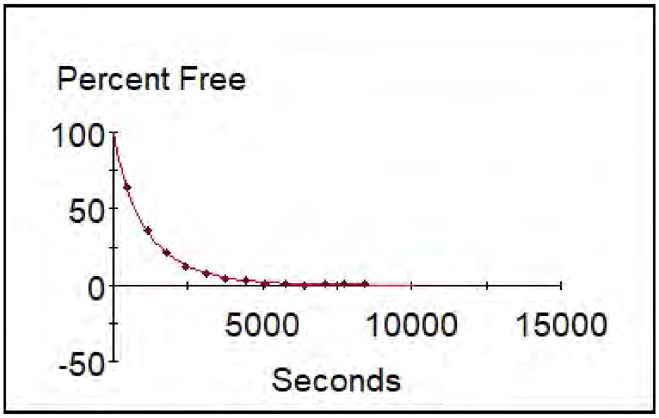

**kon:**	1.035e+06/Ms
**95% confidence interval**	
**kon High:**	1.068e+06/Ms
**kon Low:**	1.003e+06/Ms

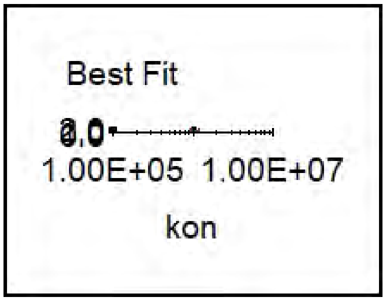

Data Traces(x)

**Cycles:**	13
**Incubation delay (min):**	0
**Mix Time:**	Wed Jul 10 11:02:40 2013

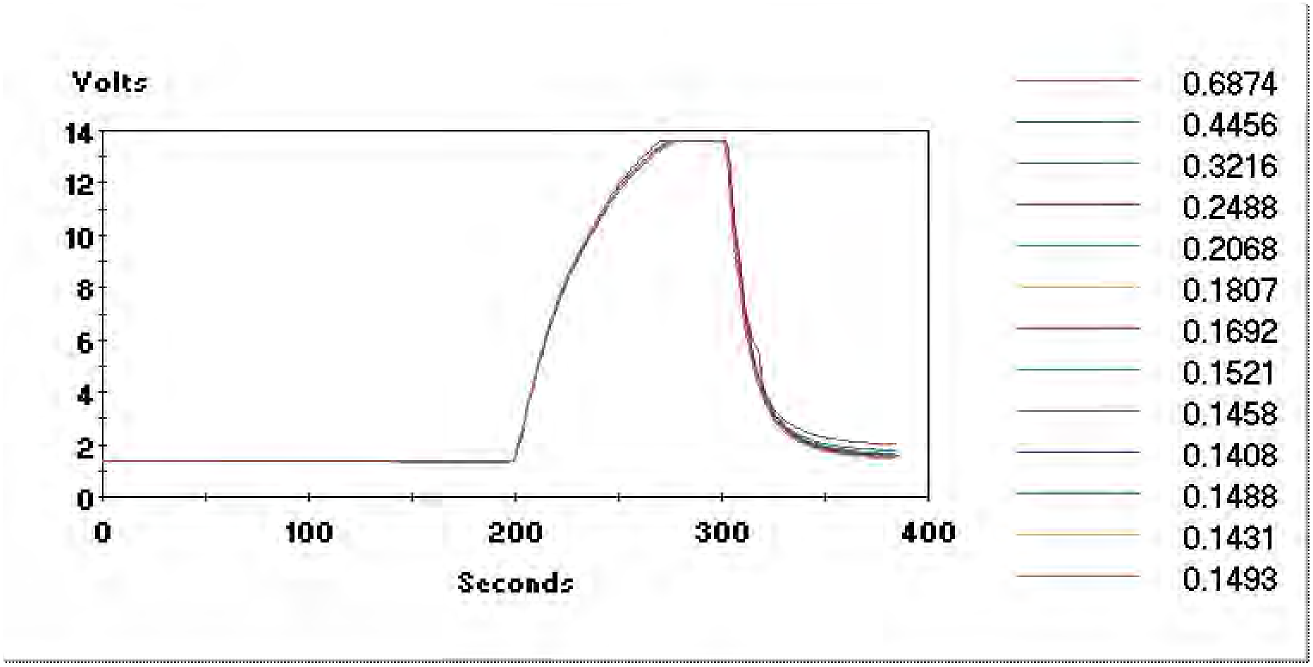

Experiment(x)

**Experiment Name:**	KinDir Hu6F11 vs NXF11 domain 082624	**Start Time:**	Mon Aug 26 14:25:45 2024
**Experiment Type:**	Kinetics, Direct	**End Time:**	Mon Aug 26 16:33:40 2024
**Binding Site Concentration:**	335.00pM	**Buffer:**	PBS/BSA
**Kd:**	25.10pM	**Label:**	Anti-His-647
**Titrant:**	200.00pM	**Label Conc:**	0

Comments(x)

beads: Hu6F11 IgG1 coated 08/20/24

sample volume: 500 ul

detection: Anti-His -647 (1:500)

CBP: 335 pM [final] NXF11 Domain

titrant: 200 pM [final] Hu6F11 IgG1 08/26/24

beads: Hu6F11 IgG1 coated 08/20/24

sample volume: 500 ul

detection: Anti-His -647 (1:500)

CBP: 335 pM [final] NXF11 Domain

titrant: 200 pM [final] Hu6F11 IgG1 08/26/24

Timing(x)

Bead Handling (Custom Beads)

Draw Source	Time (sec)	Volume (uL)	Rate (mL/min)	Stir
Backflush	20	0	0.0000	
Buffer	20	500	1.5000	✓
Particle Reservoir 1	23	380	1.0000	✓
Buffer	30	500	1.0000	
Waste	2	8	0.2500	
Buffer	20	0	0.0000	
Buffer	9	150	1.0000	

Sample Timing

Draw Source	Time (sec)	Volume (uL)	Rate (mL/min)	Time Stamp
Rack 2: Tube 1	120	500	0.2500	
Buffer	30	125	0.2500	
Rack 2: Tube 60	120	500	0.2500	
Buffer	30	125	0.2500	
Buffer	90	1500	1.0000	

Analysis(x)

Baseline / Endpoints:
to (sec) from beginning
to (sec) from end

Binding

Ignore	Signal (V)	Time
	0.5822	299
	0.4989	995
	0.4497	1691.5
	0.4138	2388
	0.4190	3085
	0.3844	3781.5
	0.3751	4478.5
	0.3335	5175.5
	0.3440	5873.5
	0.3501	6570.5
	0.3051	7268.5

**kon:**	1.154e+06/Ms
**koff:**	2.895e-05/s
**Sig 100%:**	0.61
**NSB:**	-0.00
**%Error:**	2.16
**Kd:**	25.10pM
**CBP:**	335.00pM
**Titrant:**	200.00pM

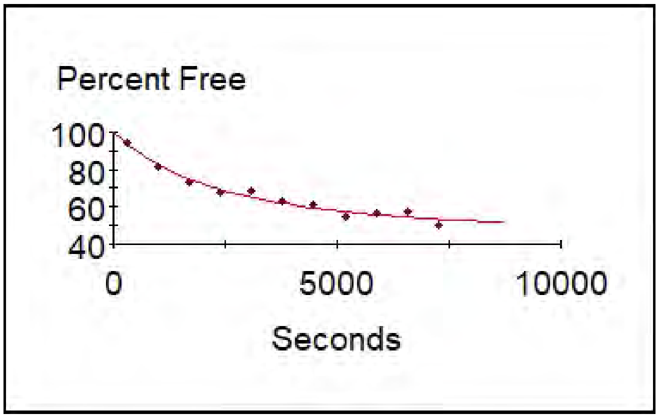

**kon:**	1.154e+06/Ms
**95% confidence interval**	
**kon High:**	1.693e+06/Ms
**kon Low:**	7.108e+05/Ms

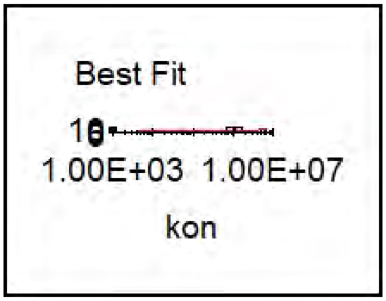

Data Traces(x)

**Cycles:**	11
**Incubation delay (min):**	0
**Mix Time:**	Mon Aug 26 14:25:43 2024

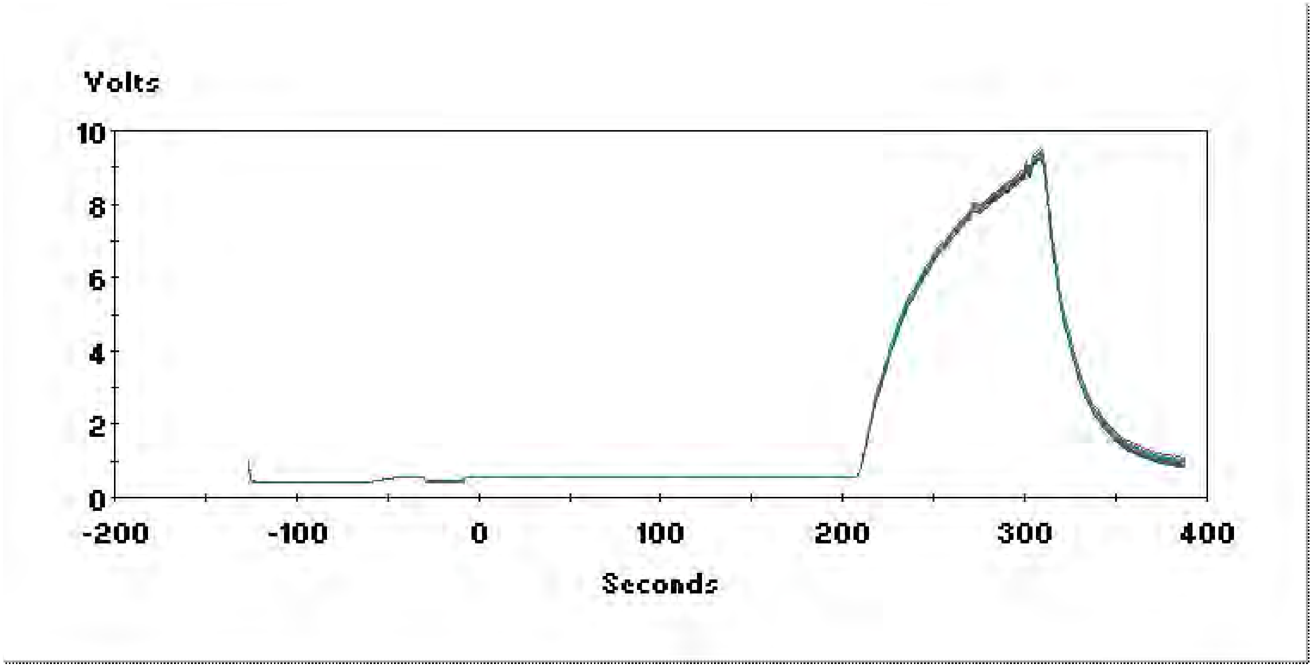

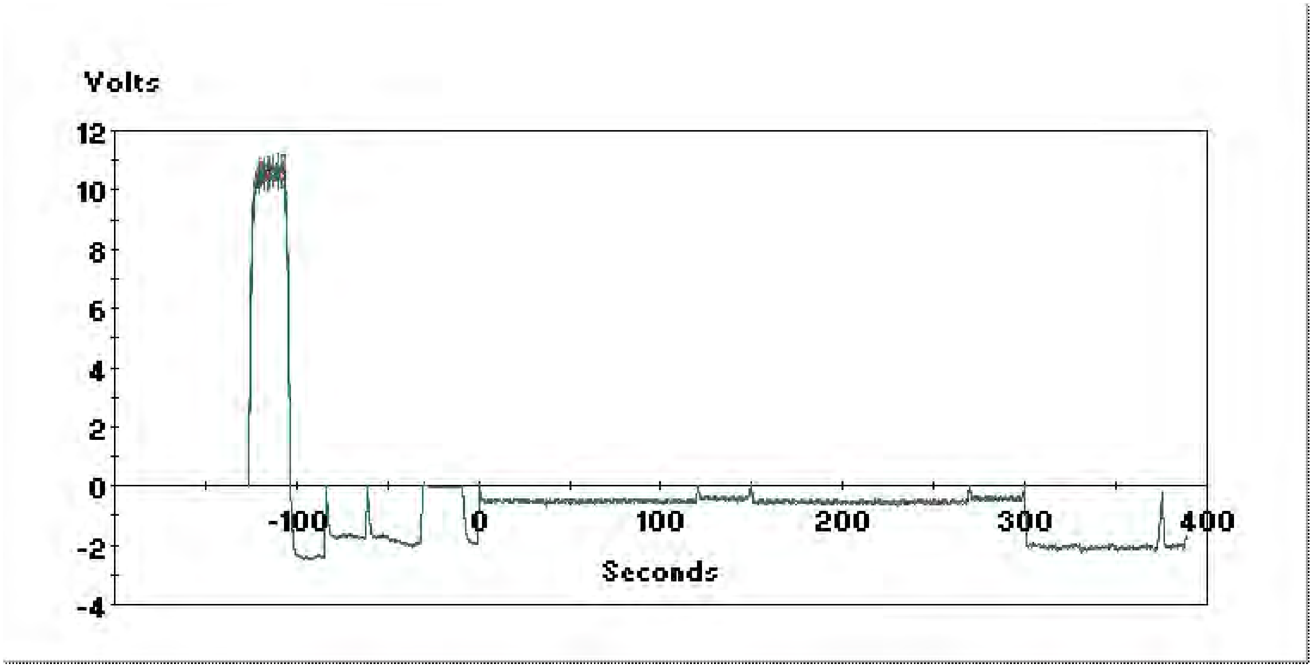

Experiment(x)

**Experiment Name:**	KinDir !3.4IgG1 vs F1 toxin 090224	**Start Time:**	Mon Sep 2 17:21:25 2024
**Experiment Type:**	Kinetics, Direct	**End Time:**	Mon Sep 2 19:29:24 2024
**Binding Site Concentration:**	200.00pM	**Buffer:**	PBS/BSA
**Kd:**	80.47pM	**Label:**	Hu6F10&Hu6F15.4-647
**Titrant:**	100.00pM	**Label Conc:**	0

Comments(x)

beads: Hu6F13.4 IgG1 coated 08/28/24

sample volume: 500 ul

detection: Hu6F10&Hu6F15.4-647 (1:800)

CBP: 100 pM [final] BoNT/F1

titrant: 100 pM [final] Hu6F14.3 IgG 09/02/24

beads: Hu6F13.4 IgG1 coated 08/28/24

sample volume: 500 ul

detection: Hu6F10&Hu6F15.4-647 (1:800)

CBP: 100 pM [final] BoNT/F1

titrant: 100 pM [final] Hu6F14.3 IgG 09/02/24

Timing(x)

Bead Handling (Custom Beads)

Draw Source	Time (sec)	Volume (uL)	Rate (mL/min)	Stir
Backflush	20	0	0.0000	
Buffer	20	500	1.5000	✓
Particle Reservoir 1	23	380	1.0000	✓
Buffer	30	500	1.0000	
Waste	2	8	0.2500	
Buffer	20	0	0.0000	
Buffer	9	150	1.0000	

Sample Timing

Draw Source	Time (sec)	Volume (uL)	Rate (mL/min)	Time Stamp
Rack 2: Tube 1	120	500	0.2500	
Buffer	30	125	0.2500	
Rack 1: Tube 21	120	500	0.2500	
Buffer	30	125	0.2500	
Buffer	90	1500	1.0000	

Analysis(x)

Baseline / Endpoints:
to (sec) from beginning
to (sec) from end

Ignore	Binding Signal (V)	Time
	0.4972	299.5
	0.4204	996
	0.4116	1693.5
✓	0.5846	2389.5
	0.4141	3087
	0.3985	3783.5
	0.3549	4481.5
	0.4034	5178.5
	0.3850	5876.5
	0.3880	6574
	0.3661	7272.5

**kon:**	4.129e+06/Ms
**koff:**	3.322e-04/s
**Sig 100%:**	0.54
**NSB:**	0.04
**%Error:**	3.02
**Kd:**	80.47pM
**CBP:**	200.00pM
**Titrant:**	100.00pM

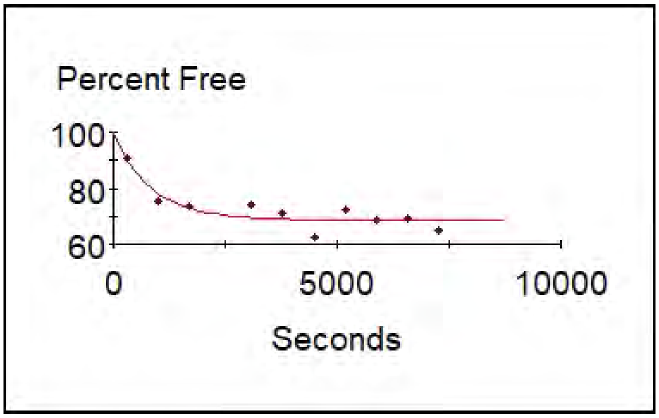

**kon:**	4.129e+06/Ms
**95% confidence interval**	
**kon High:**	1.140e+07/Ms
**kon Low:**	1.438e+06/Ms

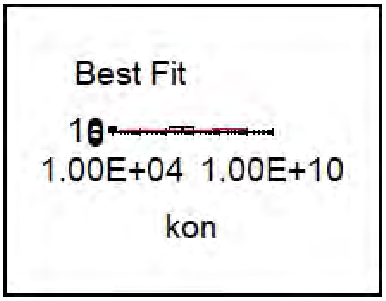

Data Traces(x)

**Cycles:**	11
**Incubation delay (min):**	0
**Mix Time:**	Mon Sep 2 17:21:23 2024

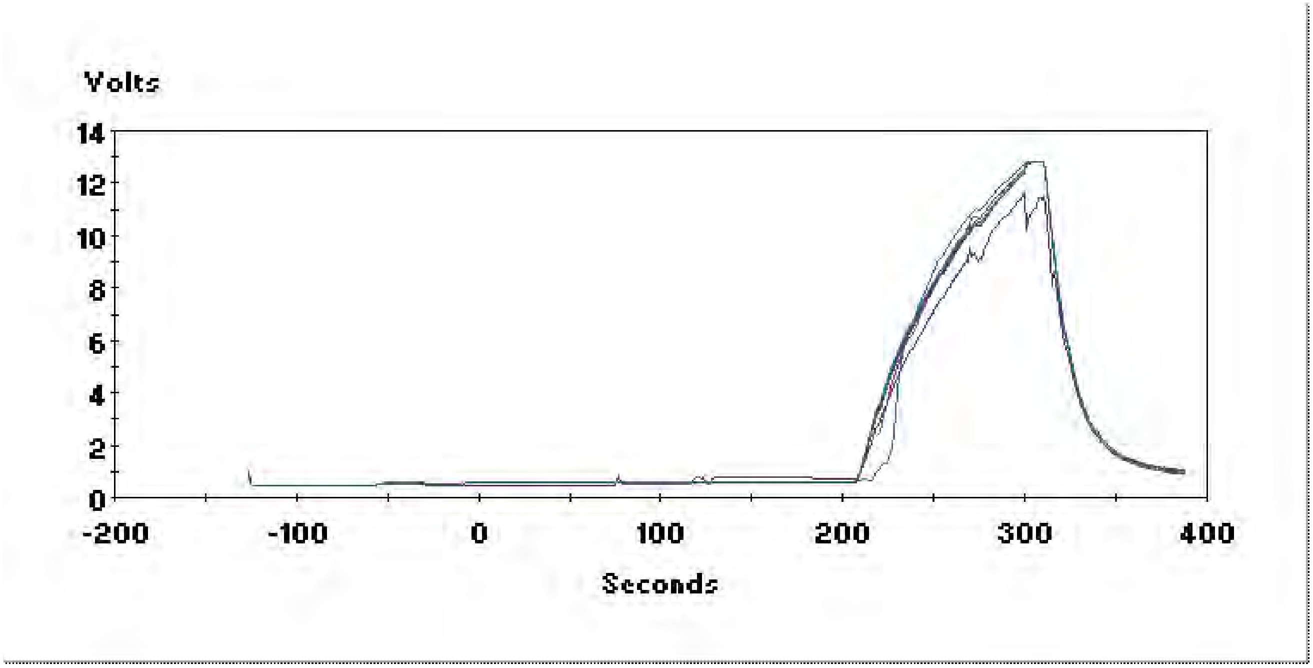

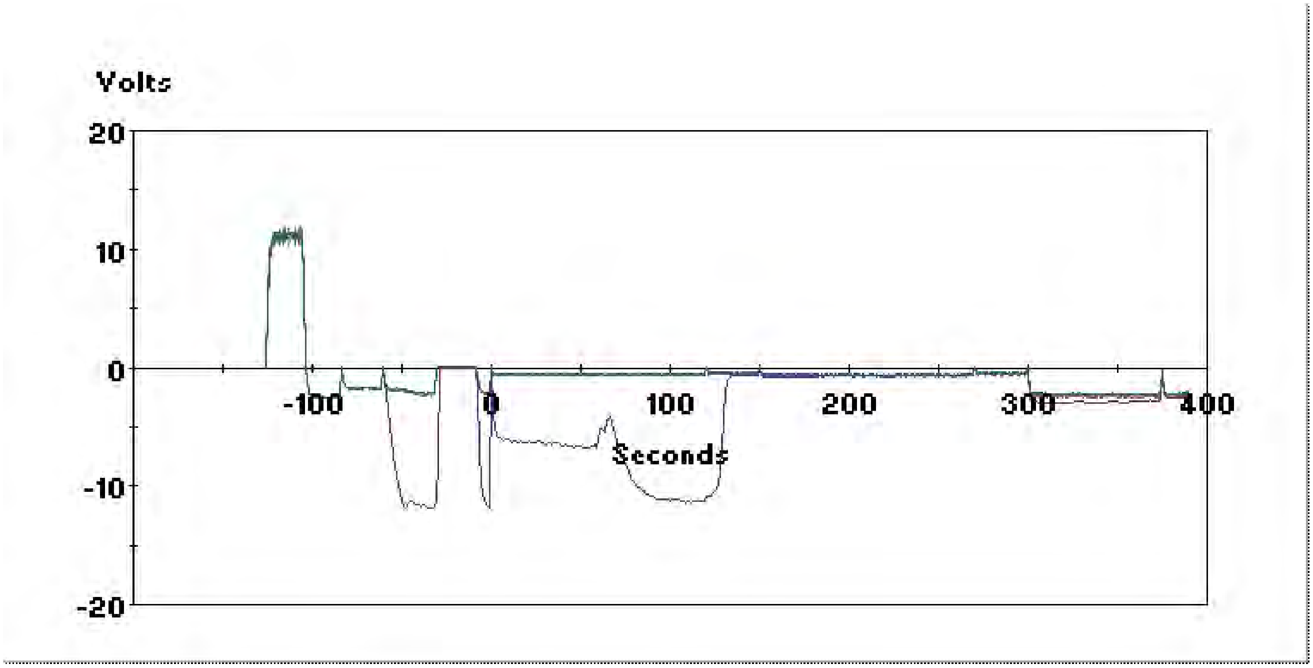

Experiment(x)

**Experiment Name:**	KinDir Hu6F13.4 vs NXF13 domain Kon 082224	**Start Time:**	Thu Aug 22 15:45:23 2024
**Experiment Type:**	Kinetics, Direct	**End Time:**	Thu Aug 22 17:53:27 2024
**Binding Site Concentration:**	100.00pM	**Buffer:**	PBS/BSA
**Kd:**	27.01pM	**Label:**	Anti-His-647
**Titrant:**	100.00pM	**Label Conc:**	0

Comments(x)

beads: Hu6F13.4 IgG1 coated 08/20/24

sample volume: 500 ul

detection: Anti-His -647 (1:500)

CBP: 200 pM [final] NXF13 Domain

titrant: 100 pM [final] TeAb-F IgG 08/21/24

beads: Hu6F13.4 IgG1 coated 08/20/24

sample volume: 500 ul

detection: Anti-His -647 (1:500)

CBP: 200 pM [final] NXF13 Domain

titrant: 100 pM [final] TeAb-F IgG 08/21/24

Timing(x)

Bead Handling (Custom Beads)

Draw Source	Time (sec)	Volume (uL)	Rate (mL/min)	Stir
Backflush	20	0	0.0000	
Buffer	20	500	1.5000	✓
Particle Reservoir 1	24	400	1.0000	✓
Buffer	30	500	1.0000	
Waste	2	8	0.2500	
Buffer	20	0	0.0000	
Buffer	9	150	1.0000	

Sample Timing

Draw Source	Time (sec)	Volume (uL)	Rate (mL/min)	Time Stamp
Rack 2: Tube 1	120	500	0.2500	
Buffer	30	125	0.2500	
Rack 2: Tube 60	120	500	0.2500	
Buffer	30	125	0.2500	
Buffer	90	1500	1.0000	

Analysis(x)

Baseline / Endpoints:
to (sec) from beginning
to (sec) from end

Binding		
**Ignore**	**Signal (V)**	**Time**
	0.2435	298
	0.2238	995.5
	0.2106	1692.5
	0.1934	2390.5
	0.1909	3087.5
	0.1969	3785.5
✓	0.2056	4483.5
	0.1886	5182
	0.1824	5880
	0.1816	6578.5
	0.1799	7277

**kon:**	4.062e+06/Ms
**koff:**	1.097e-04/s
**Sig 100%:**	0.26
**NSB:**	0.13
**%Error:**	2.57
**Kd:**	27.01pM
**CBP:**	100.00pM
**Titrant:**	100.00pM

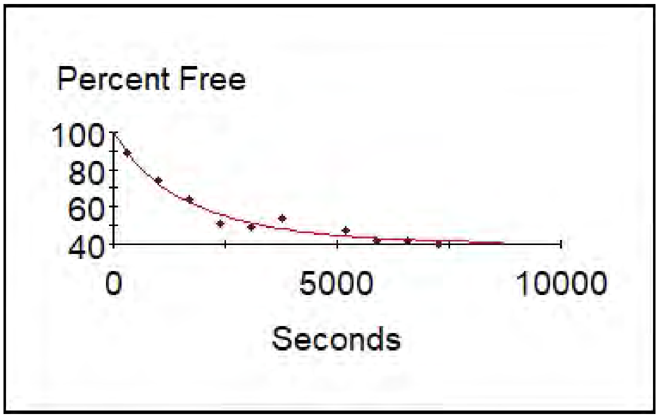

kon: 4.062e+06/Ms
95% confidence interval
kon High: 5.443e+06/Ms
kon Low: 2.896e+06/Ms

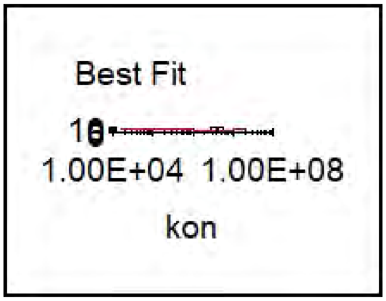

Data Traces(x)

**Cycles:**	11
**Incubation delay (min):**	0
**Mix Time:**	Thu Aug 22 15:45:22 2024

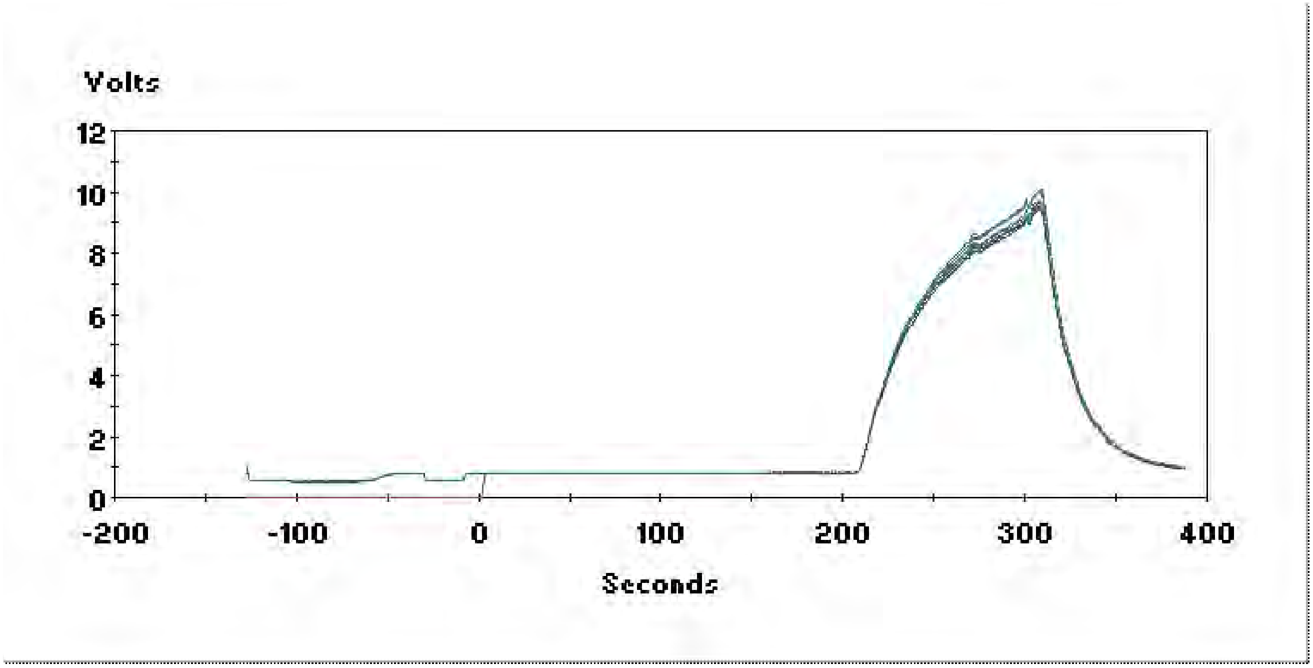

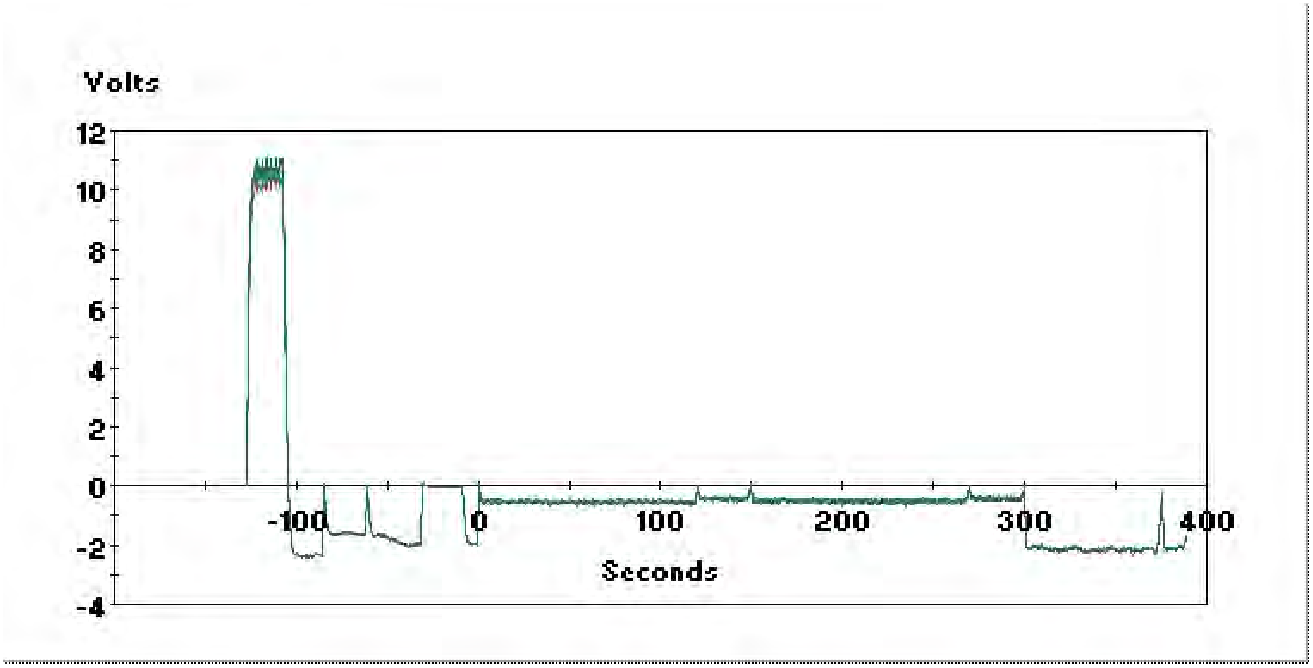

Experiment(x)

**Experiment Name:**	Kinetics Direct TeAb-F (6F5.4) vs F1 090418	**Start Time:**	Tue Sep 4 15:56:11 2018
**Experiment Type:**	Kinetics, Direct	**End Time:**	Tue Sep 4 17:57:27 2018
**Binding Site Concentration:**	800.00pM	**Buffer:**	PBS/BSA
**Kd:**	26.60pM	**Label:**	Hu6F13.2-647
**Titrant:**	800.00pM	**Label Conc:**	0

Comments(x)

beads: 6F5.4 10/11/17

sample volume: 500 ul

detection: Hu6F13.2-647

CBP: 800 pM [final] BoNT F1 100252 09/04/18

titrant: 800 pM [final] TeAb F 082818

Timing(x)

Bead Handling (Custom Beads)

Draw Source	Time (sec)	Volume (uL)	Rate (mL/min)	Stir
Backflush	20	0	0.0000	
Buffer	20	500	1.5000	✓
Particle Reservoir 1	26	433	1.0000	✓
Buffer	30	500	1.0000	
Waste	2	8	0.2500	
Buffer	20	0	0.0000	
Buffer	9	150	1.0000	

Sample Timing

Draw Source	Time (sec)	Volume (uL)	Rate (mL/min)	Time Stamp
Rack 2: Tube 1	120	500	0.2500	
Buffer	30	125	0.2500	
Rack 2: Tube 60	120	500	0.2500	
Buffer	30	125	0.2500	
Buffer	90	1500	1.0000	

Analysis(x)

Baseline / Endpoints:

to (sec) from beginning

to (sec) from end

Binding

Ignore	Signal (V)	Time
	1.2700	102
	0.9760	828.5
	0.8708	1555
	0.7921	2281.5
	0.7462	3008
	0.7442	3735
	0.7035	4462
	0.6736	5189
	0.6544	5916
	0.6259	6644

**kon:**	9.628e+05/Ms
**koff:**	2.561e-05/s
**Sig 100%:**	1.33
**NSB:**	0.47
**%Error:**	1.75
**Kd:**	26.60pM
**CBP:**	800.00pM
**Titrant:**	800.00pM

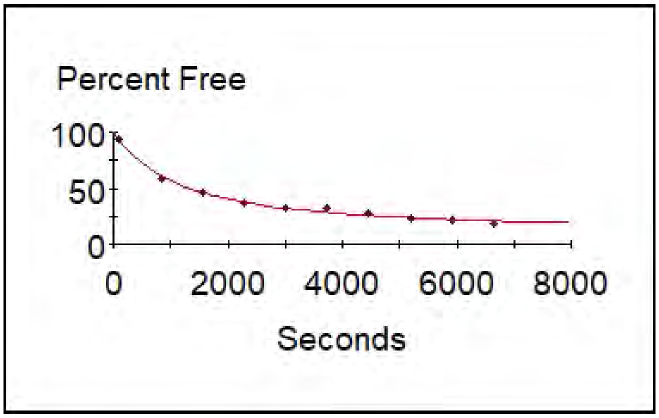

**kon:**	9.628e+05/Ms
**95% confidence interval**	
**kon High:**	1.141e+06/Ms
**kon Low:**	8.055e+05/Ms

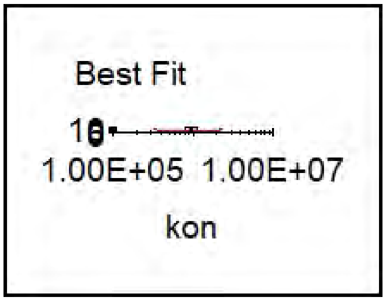

Data Traces(x)

**Cycles:**	10
**Incubation delay (min):**	0
**Mix Time:**	Tue Sep 4 15:59:54 2018

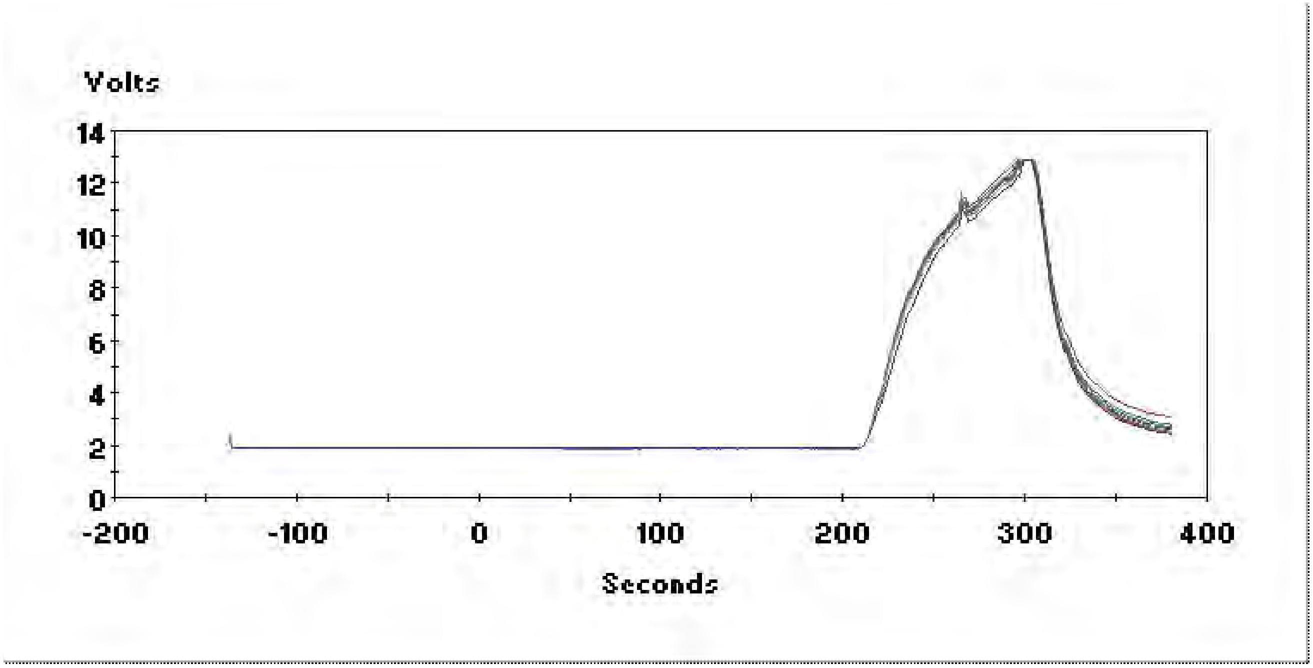

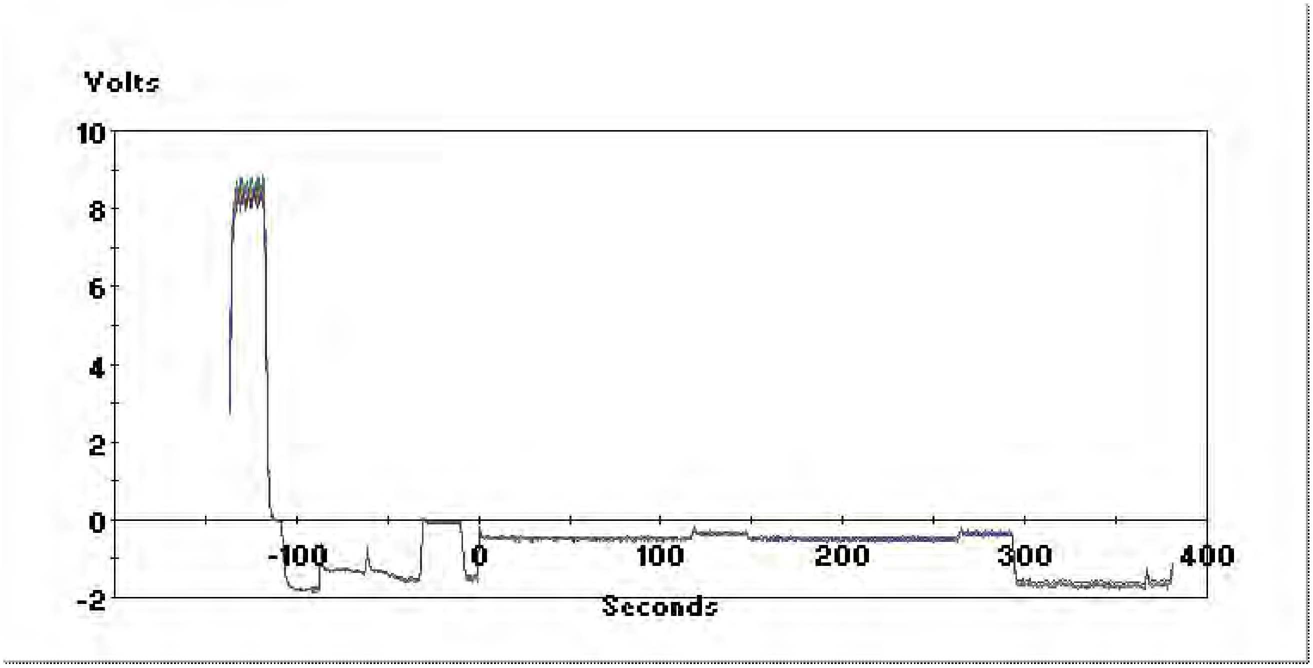

Experiment(x)

**Experiment Name:**	KinDir TeAb-F vs NXF5 domain Kon 082124	**Start Time:**	Wed Aug 21 15:33:06 2024
**Experiment Type:**	Kinetics, Direct	**End Time:**	Wed Aug 21 17:52:38 2024
**Binding Site Concentration:**	100.00pM	**Buffer:**	PBS/BSA
**Kd:**	4.41pM	**Label:**	Anti-His-647
**Titrant:**	50.00pM	**Label Conc:**	0

Comments(x)

beads: 6F5.4 IgG1 coated 08/20/24

sample volume: 500 ul

detection: Anti-His -647 (1:500)

CBP: 100 pM [final] NXF5 Domain

titrant: 50 pM [final] TeAb-F IgG 08/21/24

beads: 6F5.4 IgG1 coated 08/20/24

sample volume: 500 ul

detection: Anti-His -647 (1:500)

CBP: 100 pM [final] NXF5 Domain

titrant: 50 pM [final] TeAb-F IgG 08/21/24

Timing(x)

Bead Handling (Custom Beads)

Draw Source	Time (sec)	Volume (uL)	Rate (mL/min)	Stir
Backflush	20	0	0.0000	
Buffer	20	500	1.5000	✓
Particle Reservoir 1	23	380	1.0000	✓
Buffer	30	500	1.0000	
Waste	2	8	0.2500	
Buffer	20	0	0.0000	
Buffer	9	150	1.0000	

Sample Timing

Draw Source	Time (sec)	Volume (uL)	Rate (mL/min)	Time Stamp
Rack 2: Tube 1	120	500	0.2500	
Buffer	30	125	0.2500	
Rack 2: Tube 60	120	500	0.2500	
Buffer	30	125	0.2500	
Buffer	90	1500	1.0000	

Analysis(x)

Baseline / Endpoints:
to (sec) from beginning
to (sec) from end

Binding

Ignore	Signal (V)	Time
	0.2358	310
	0.2122	1006
	0.1988	1702.5
	0.2085	2399
	0.1954	3096
	0.1941	3792.5
	0.2094	4489.5
	0.1871	5186.5
	0.2058	5884
	0.1837	6581
	0.1975	7279.5
	0.1920	7976.5

**kon:**	1.390e+07/Ms
**koff:**	6.131e-05/s
**Sig 100%:**	0.26
**NSB:**	0.12
**%Error:**	5.49
**Kd:**	4.41pM
**CBP:**	100.00pM
**Titrant:**	50.00pM

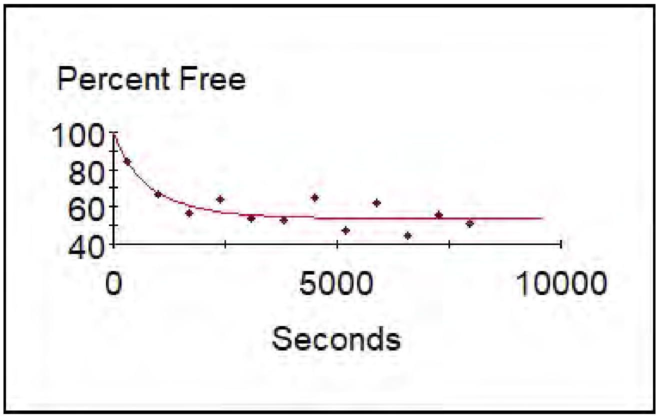

**kon:**	1.390e+07/Ms
**95% confidence interval**	
**kon High:**	5.970e+07/Ms
**kon Low:**	3.823e+06/Ms

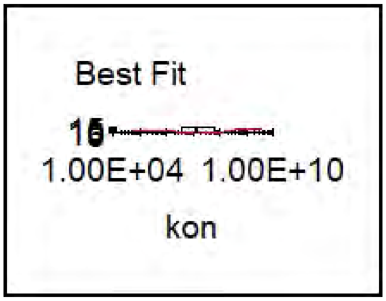

Data Traces(x)

**Cycles:**	12
**Incubation delay (min):**	0
**Mix Time:**	Wed Aug 21 15:32:53 2024

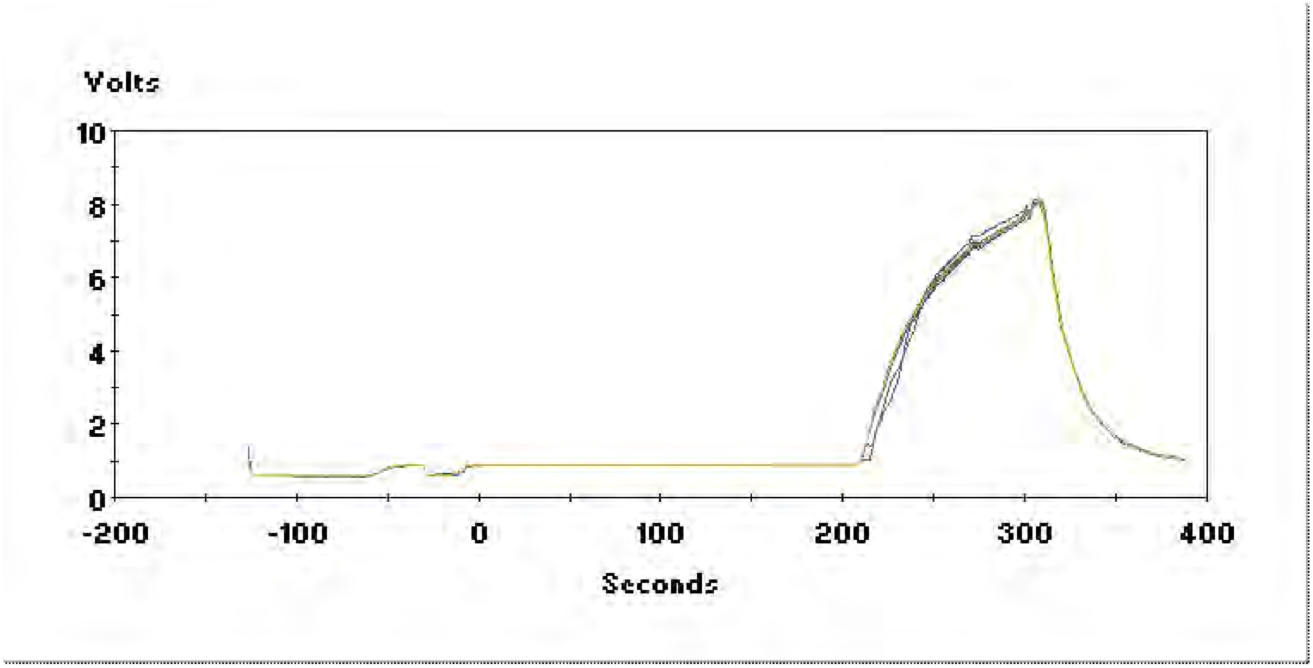

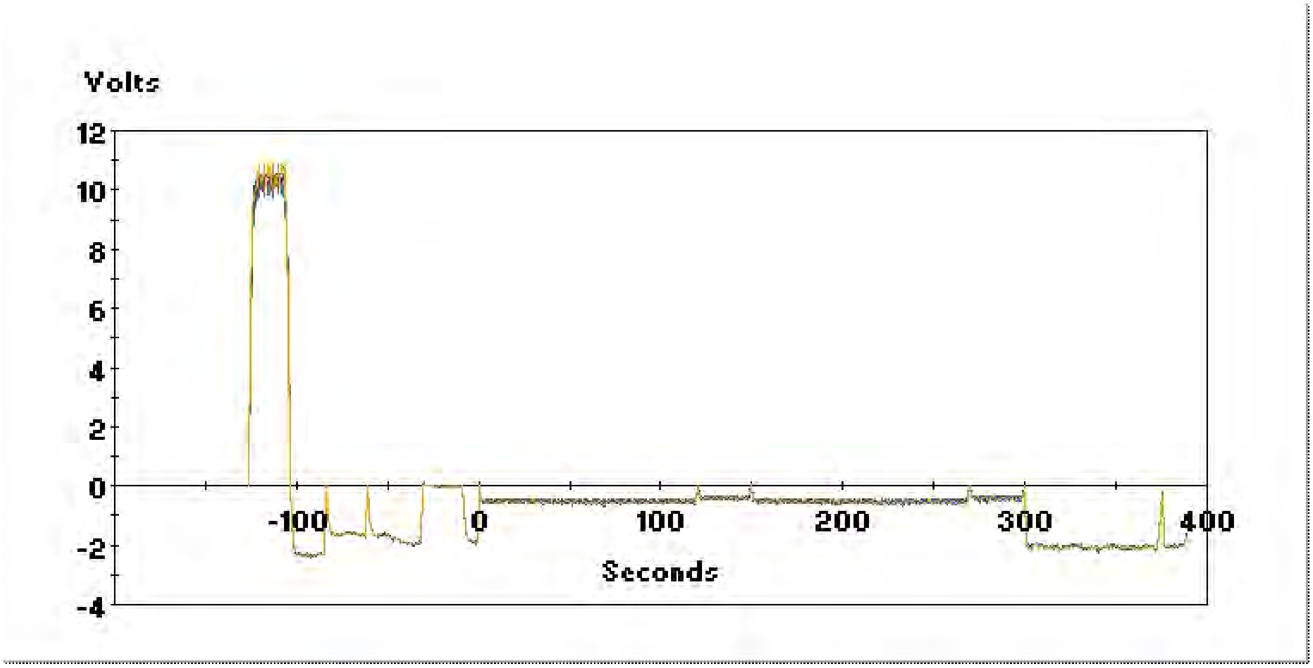

Experiment(x)

**Experiment Name:**	Kinetics Direct TeAb-F (Hu6F11) vs F1	**Start Time:**	Wed Sep 5 13:47:36 2018
**Experiment Type:**	Kinetics, Direct	**End Time:**	Wed Sep 5 15:47:47 2018
**Binding Site Concentration:**	500.00pM	**Buffer:**	PBS/BSA
**Kd:**	23.30pM	**Label:**	6F5.4-647
**Titrant:**	800.00pM	**Label Conc:**	0

Comments(x)

beads:Hu6F11 09/04/18

sample volume: 500 ul

detection: 6F5.4-647

CBP: 500 pM [final] BoNT F1 100252 09/05/18

titrant: 800 pM [final] TeAb F 082818

Timing(x)

Bead Handling (Custom Beads)

Draw Source	Time (sec)	Volume (uL)	Rate (mL/min)	Stir
Backflush	20	0	0.0000	
Buffer	20	500	1.5000	✓
Particle Reservoir 1	20	333	1.0000	✓
Buffer	30	500	1.0000	
Waste	2	8	0.2500	
Buffer	20	0	0.0000	
Buffer	9	150	1.0000	

Sample Timing

Draw Source	Time (sec)	Volume (uL)	Rate (mL/min)	Time Stamp
Rack 2: Tube 1	120	500	0.2500	
Buffer	30	125	0.2500	
Rack 2: Tube 60	120	500	0.2500	
Buffer	30	125	0.2500	
Buffer	90	1500	1.0000	

Analysis(x)

Baseline / Endpoints:
to (sec) from beginning
to (sec) from end

Binding

Ignore	Signal (V)	Time
	0.9288	97
	0.6970	817.5
	0.5788	1537
	0.5112	2257.5
	0.4699	2977
	0.4369	3698
	0.4149	4418
	0.3960	5139
	0.3742	5859
	0.3772	6581

**kon:**	9.523e+05/Ms
**koff:**	2.219e-05/s
**Sig 100%:**	0.97
**NSB:**	0.31
**%Error:**	0.63
**Kd:**	23.30pM
**CBP:**	500.00pM
**Titrant:**	800.00pM

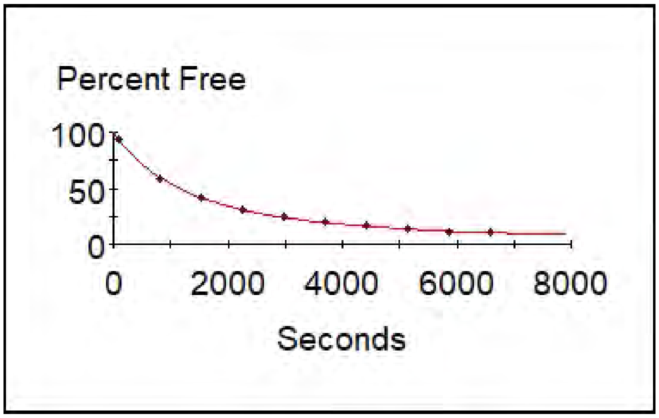

**kon:**	9.523e+05/Ms
**95% confidence interval**	
**kon High:**	9.982e+05/Ms
**kon Low:**	9.067e+05/Ms

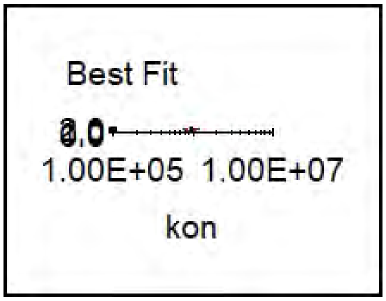

Data Traces(x)

**Cycles:**	10
**Incubation delay (min):**	0
**Mix Time:**	Wed Sep 5 13:51:17 2018

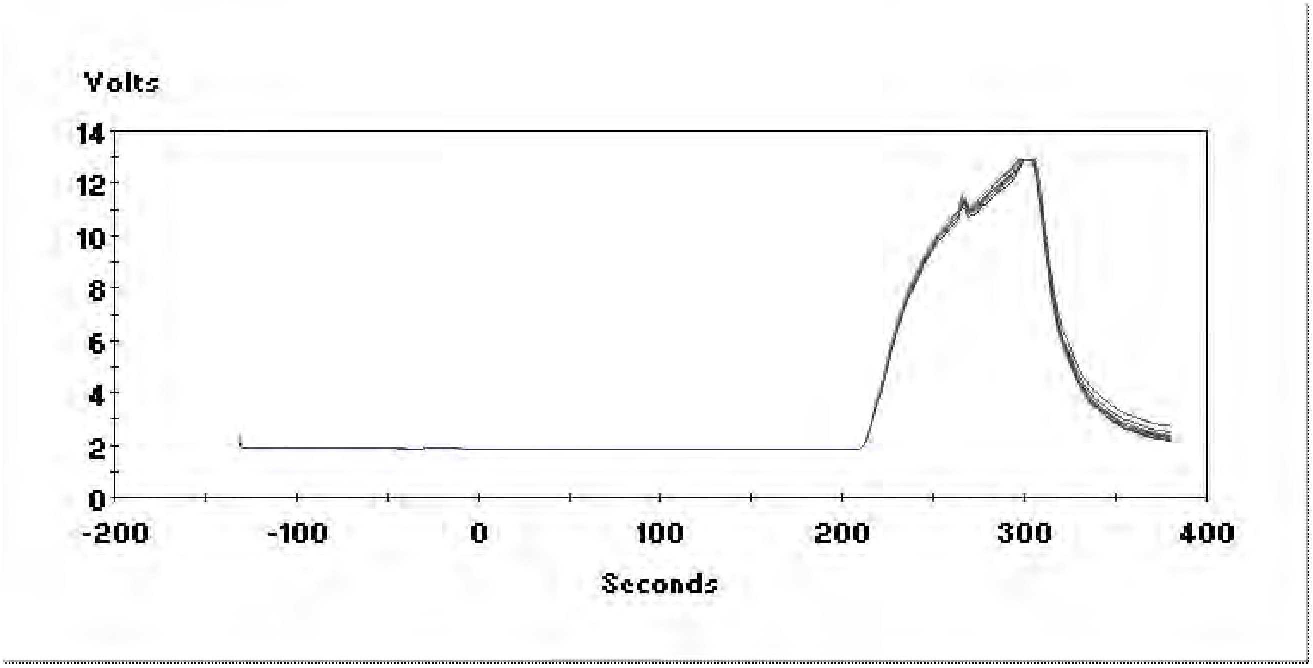

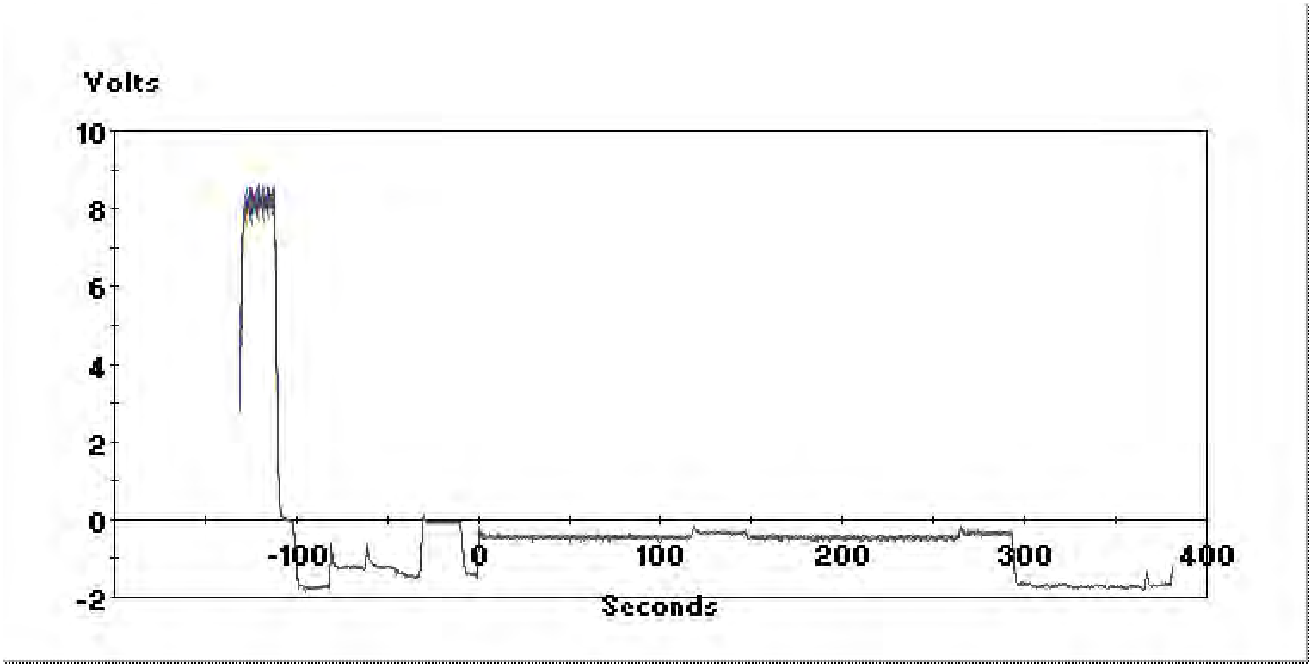

Experiment(x)

**Experiment Name:**	KinDir TeAb-F vs F1 toxin(Hu6F13.4) 090124	**Start Time:**	Sun Sep 1 14:01:11 2024
**Experiment Type:**	Kinetics, Direct	**End Time:**	Sun Sep 1 16:09:09 2024
**Binding Site Concentration:**	100.00pM	**Buffer:**	PBS/BSA
**Kd:**	15.50pM	**Label:**	Hu6F10&Hu6F15.4-647
**Titrant:**	100.00pM	**Label Conc:**	0

Comments(x)

beads: Hu6F13.4 IgG1 coated 08/28/24

sample volume: 500 ul

detection: Hu6F10&Hu6F15.4-647 (1:800)

CBP: 100 pM [final] BoNT/F1

titrant: 100 pM [final] TeAb-F IgG 09/01/24

beads: Hu6F13.4 IgG1 coated 08/28/24

sample volume: 500 ul

detection: Hu6F10&Hu6F15.4-647 (1:800)

CBP: 100 pM [final] BoNT/F1

titrant: 100 pM [final] TeAb-F IgG 09/01/24

Timing(x)

Bead Handling (Custom Beads)

Draw Source	Time (sec)	Volume (uL)	Rate (mL/min)	Stir
Backflush	20	0	0.0000	
Buffer	20	500	1.5000	✓
Particle Reservoir 1	23	380	1.0000	✓
Buffer	30	500	1.0000	
Waste	2	8	0.2500	
Buffer	20	0	0.0000	
Buffer	9	150	1.0000	

Sample Timing

Draw Source	Time (sec)	Volume (uL)	Rate (mL/min)	Time Stamp
Rack 2: Tube 1	120	500	0.2500	
Buffer	30	125	0.2500	
Rack 1: Tube 21	120	500	0.2500	
Buffer	30	125	0.2500	
Buffer	90	1500	1.0000	

Analysis(x)

Baseline / Endpoints:

to (sec) from beginning
to (sec) from end

Binding

Ignore	Signal (V)	Time
	0.8089	299.5
	0.1928	995.5
	0.1932	1692.5
	0.2094	2389
	0.1851	3086
	0.1829	3783
	0.1765	4480.5
	0.1836	5177.5
	0.1776	5875.5
	0.1748	6573
	0.1800	7271.5

**kon:**	5.286e+07/Ms
**koff:**	8.193e-04/s
**Sig 100%:**	3.75
**NSB:**	-1.53
**%Error:**	0.20
**Kd:**	15.50pM
**CBP:**	100.00pM
**Titrant:**	100.00pM

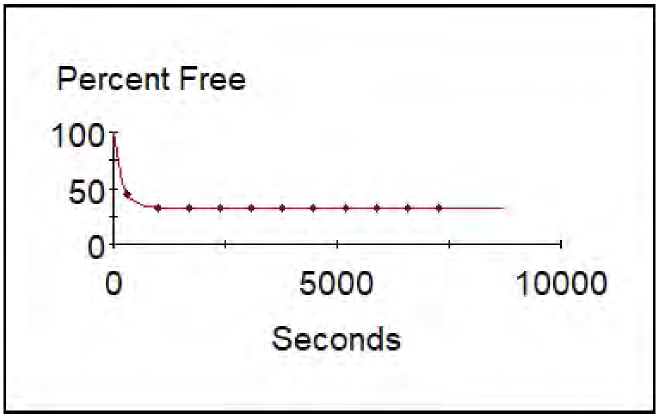

**kon:**	5.286e+07/Ms
**95% confidence interval**	
**kon High:**	1.103e+08/Ms
**kon Low:**	4.765e+07/Ms

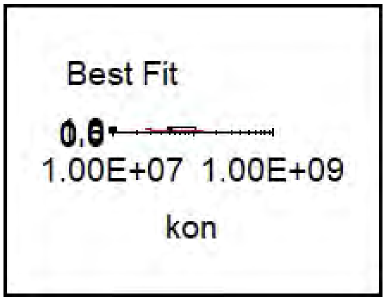

Data Traces(x)

**Cycles:**	11
**Incubation delay (min):**	0
**Mix Time:**	Sun Sep 1 14:01:09 2024

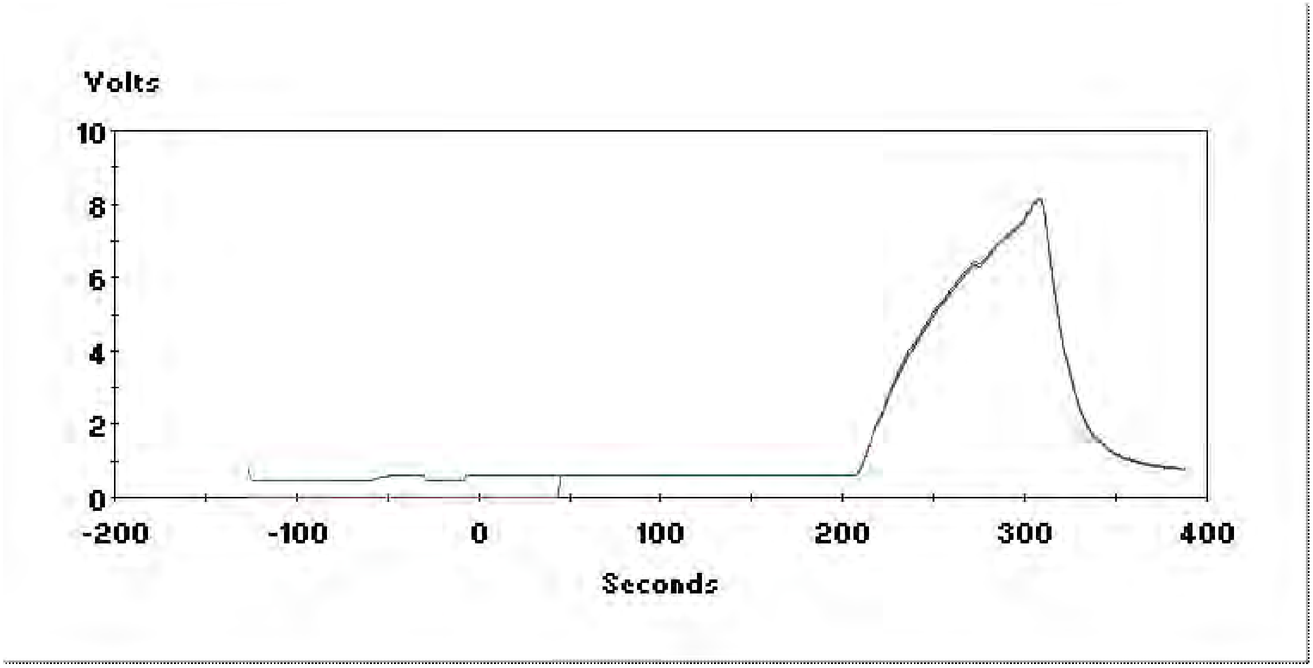

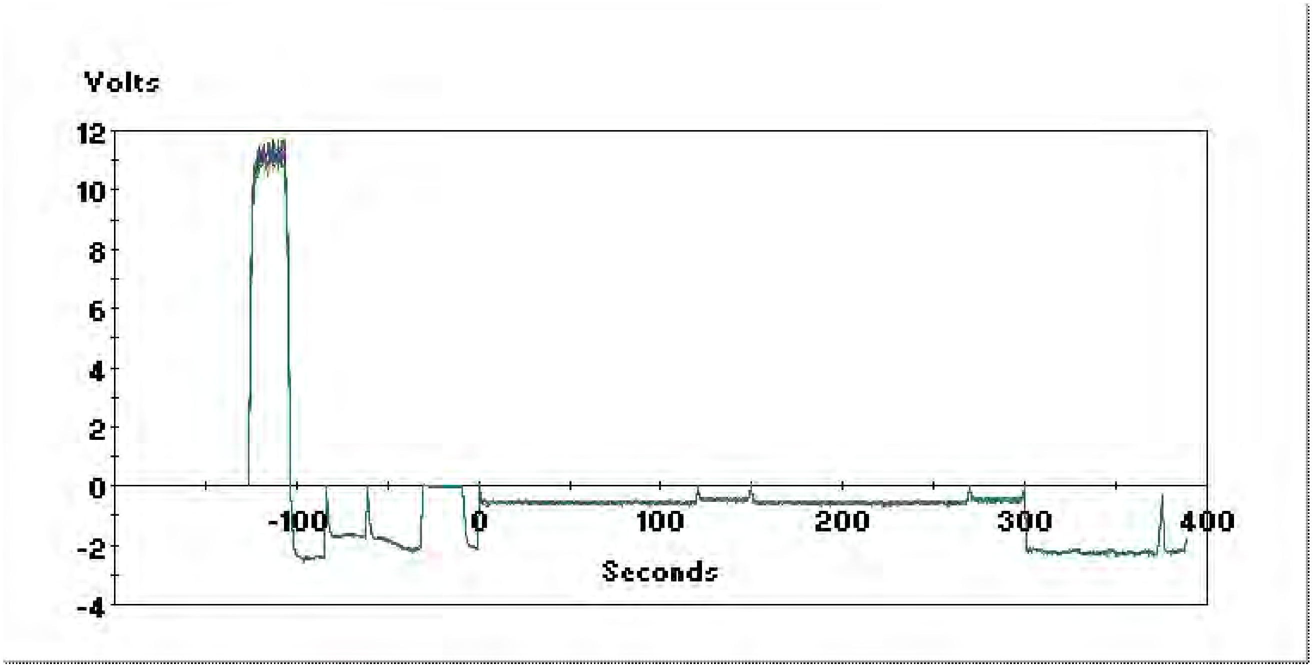

Experiment(x)

**Experiment Name:**	KinDir TeAb-F vs NXF13 domain 082624	**Start Time:**	Mon Aug 26 19:30:35 2024
**Experiment Type:**	Kinetics, Direct	**End Time:**	Mon Aug 26 21:38:40 2024
**Binding Site Concentration:**	300.00pM	**Buffer:**	PBS/BSA
**Kd:**	1.79nM	**Label:**	Anti-His-647
**Titrant:**	200.00pM	**Label Conc:**	0

Comments(x)

beads: Hu6F13.4 IgG1 coated 08/20/24

sample volume: 500 ul

detection: Anti-His -647 (1:500)

CBP: 300 pM [final] NXF13 Domain

titrant: 200 pM [final] TeAb-F IgG 08/26/24

beads: Hu6F13.4 IgG1 coated 08/20/24

sample volume: 500 ul

detection: Anti-His -647 (1:500)

CBP: 300 pM [final] NXF13 Domain

titrant: 200 pM [final] TeAb-F IgG 08/26/24

Timing(x)

Bead Handling (Custom Beads)

Draw Source	Time (sec)	Volume (uL)	Rate (mL/min)	Stir
Backflush	20	0	0.0000	
Buffer	20	500	1.5000	✓
Particle Reservoir 1	24	400	1.0000	✓
Buffer	30	500	1.0000	
Waste	2	8	0.2500	
Buffer	20	0	0.0000	
Buffer	9	150	1.0000	

Sample Timing

Draw Source	Time (sec)	Volume (uL)	Rate (mL/min)	Time Stamp
Rack 2: Tube 1	120	500	0.2500	
Buffer	30	125	0.2500	
Rack 2: Tube 60	120	500	0.2500	
Buffer	30	125	0.2500	
Buffer	90	1500	1.0000	

Analysis (x)

Baseline / Endpoints:

to (sec) from beginning

to (sec) from end

Binding

Ignore	Signal (V)	Time
	0.8361	305
	0.5508	1001.5
	0.5261	1699.5
✓	0.8750	2396.5
	0.4702	3094.5
	0.4505	3792
	0.4611	4490
	0.4685	5188
✓	0.6088	5886.5
	0.4451	6584.5
	0.4216	7283.5

**kon:**	7.290e+05/Ms
**koff:**	1.305e-03/s
**Sig 100%:**	1.08
**NSB:**	-5.97
**%Error:**	0.24
**Kd:**	1.79nM
**CBP:**	300.00pM
**Titrant:**	200.00pM

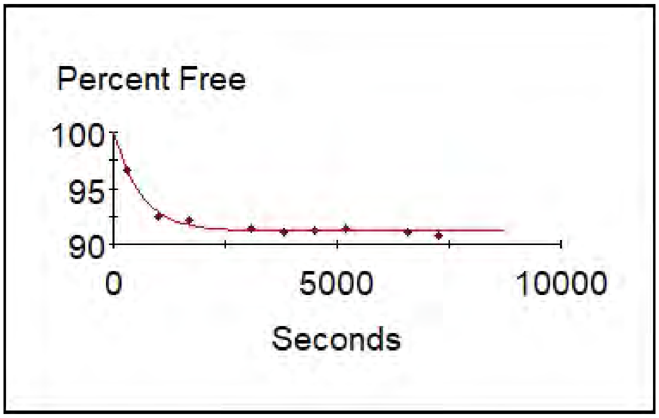

kon: 7.290e+05/Ms

95% confidence interval

kon High: 9.625e+05/Ms

kon Low: 5.785e+05/Ms

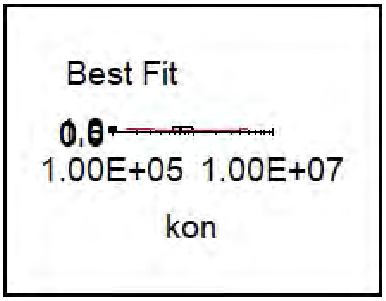

Data Traces(x)

**Cycles:**	11
**Incubation delay (min):**	0
**Mix Time:**	Mon Aug 26 19:30:28 2024

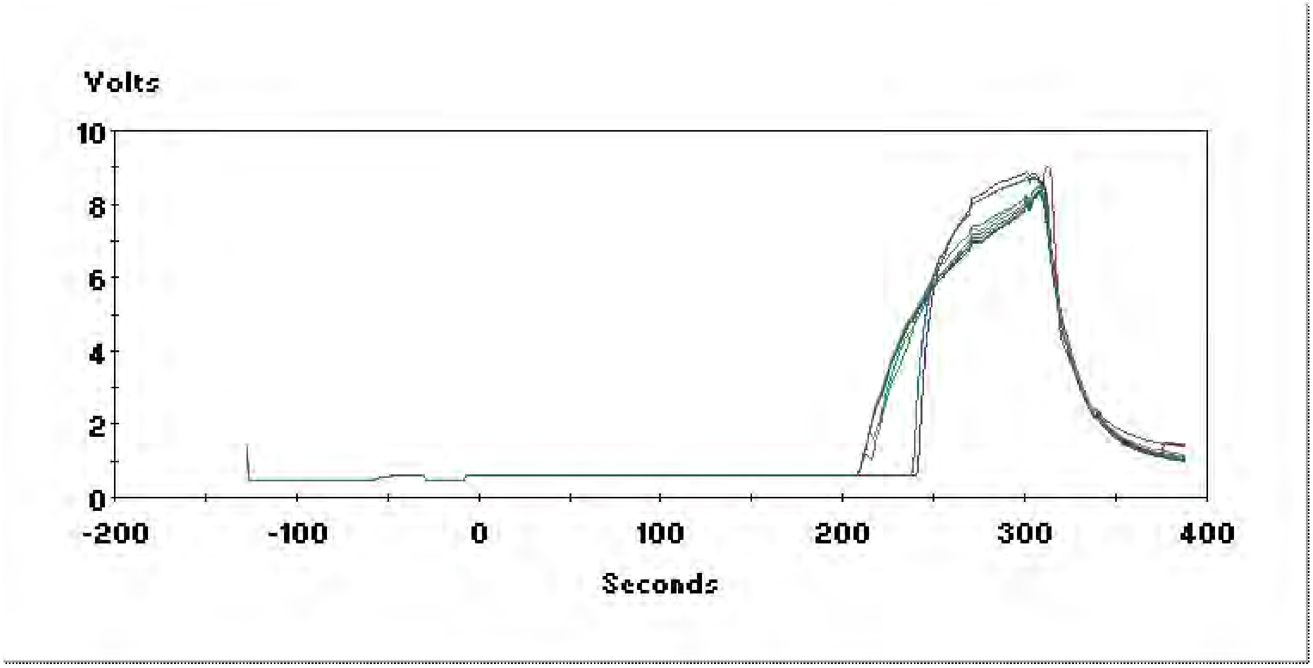

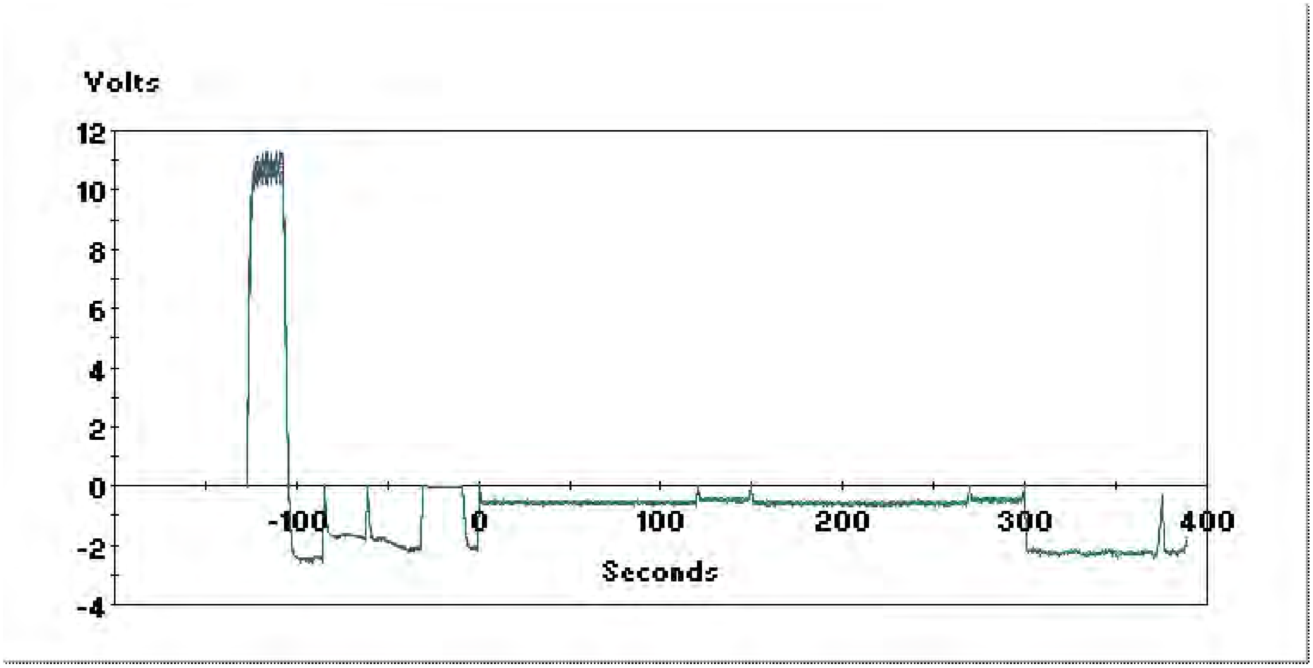

Supplement: Supplementary file 1 [file toxins-17-00281-s001.zip › Fig S3.pdf]
